# ﻿A revision of the parasitoid wasp genus *Alphomelon* Mason with the description of 30 new species (Hymenoptera, Braconidae)

**DOI:** 10.3897/zookeys.1175.105068

**Published:** 2023-08-16

**Authors:** Jose L. Fernandez-Triana, Eduardo M. Shimbori, James B. Whitfield, Angelica M. Penteado-Dias, Scott R. Shaw, Caroline Boudreault, Jayme Sones, Kate Perez, Allison Brown, Ramya Manjunath, John M. Burns, P. D. N. Hebert, M. Alex Smith, Winnifred Hallwachs, Daniel H. Janzen

**Affiliations:** 1 Canadian National Collection of Insects, Arachnids and Nematodes, 960 Carling Ave., Ottawa, K1A0C6, Canada; 2 Colección Nacional de Insectos, Instituto de Biología, Universidad Nacional Autónoma de México, Tercer Circuito, S/N, Ciudad Universitaria, Coyoacán, C.P. 04510, Ciudad de México, Mexico; 3 University of Illinois, Urbana-Champaign, USA; 4 Universidade Federal de São Carlos, São Carlos, Brazil; 5 College of Agriculture and Natural Resources, University of Wyoming, Laramie, USA; 6 Canadian Centre for DNA Barcoding, University of Guelph, Guelph, Canada; 7 Department of Entomology, National Museum of Natural History, Smithsonian Institution, Washington D.C., USA; 8 University of Guelph, Guelph, Canada; 9 Department of Biology, University of Pennsylvania, Philadelphia, USA

**Keywords:** Biology, DNA barcoding, Microgastrinae, morphology, Nearctic, Neotropical

## Abstract

The parasitoid wasp genus *Alphomelon* Mason, 1981 is revised, based on a combination of basic morphology (dichotomous key and brief diagnostic descriptions), DNA barcoding, biology (host data and wasp cocoons), and distribution data. A total of 49 species is considered; the genus is almost entirely Neotropical (48 species recorded from that region), but three species reach the Nearctic, with one of them extending as far north as 45° N in Canada. *Alphomelon* parasitizes exclusively Hesperiinae caterpillars (Lepidoptera: Hesperiidae), mostly feeding on monocots in the families Arecaceae, Bromeliaceae, Cannaceae, Commelinaceae, Heliconiaceae, and Poaceae. Most wasp species parasitize either on one or very few (2–4) host species, usually within one or two hesperiine genera; but some species can parasitize several hosts from up to nine different hesperiine genera. Among species with available data for their cocoons, roughly half weave solitary cocoons (16) and half are gregarious (17); cocoons tend to be surrounded by a rather distinctive, coarse silk (especially in solitary species, but also distinguishable in some gregarious species). Neither morphology nor DNA barcoding alone was sufficient on its own to delimit all species properly; by integrating all available evidence (even if incomplete, as available data for every species is different) a foundation is provided for future studies incorporating more specimens, especially from South America. The following 30 **new species** are described: *cruzi*, *itatiaiensis*, and *palomae*, authored by Shimbori & Fernandez-Triana; and *adrianguadamuzi*, *amazonas*, *andydeansi*, *calixtomoragai*, *carolinacanoae*, *christerhanssoni*, *diniamartinezae*, *duvalierbricenoi*, *eldaarayae*, *eliethcantillanoae*, *gloriasihezarae*, *guillermopereirai*, *hazelcambroneroae*, *josecortesi*, *keineraragoni*, *luciarosae*, *manuelriosi*, *mikesharkeyi*, *osvaldoespinozai*, *paramelanoscelis*, *paranigriceps*, *petronariosae*, *ricardocaleroi*, *rigoi*, *rostermoragai*, *sergioriosi*, and *yanayacu*, authored by Fernandez-Triana & Shimbori.

## ﻿Introduction

The genus *Alphomelon* (Hymenoptera: Braconidae, Microgastrinae) was described by Mason (1981) to accommodate three New World species, previously described under different genera, but all having in common a white spot on the gena, near the mandible base. Deans et al. (2003) revised the genus, including the description of 13 new Neotropical species, and provided the only available key to all known species at the time. That same year, Shimabukuro and Penteado-Dias (2003) added two new species from Brazil. Even today, *Alphomelon* is still restricted to the New World, and at present includes 19 described species mostly from the Neotropics but with a few extending north into the Nearctic. The overall species richness is estimated to exceed 50 species (Fernandez-Triana et al. 2020).

This study contributes to the ongoing insect biodiversity inventory by rearing and Malaise trapping of the 120,000 terrestrial ha Área de Conservación Guanacaste (ACG) in northwestern Costa Rica, a complex of dry forest, cloud forest, and rain forest, and many intergrades (Janzen and Hallwachs 2016). Here, we revise *Alphomelon* with an emphasis on the ACG species but also describe additional species from other Neotropical areas.

We provide an updated key, illustrations for all known species, and comments on natural history and DNA barcoding data. We also discuss the challenges of describing new species by combining morphological, molecular, and biological data while integrating previously described species based on less comprehensive data.

## ﻿Materials and methods

### ﻿Taxon sampling

As part of studies on the Microgastrinae fauna of ACG, we examined a significant number of *Alphomelon* specimens from that area but also many unidentified specimens of the genus from many Neotropical countries stored in the Canadian National Collection of insects, arachnids and nematodes, Ottawa, Canada (**CNC**), Coleção Entomológica do Departamento de Ecologia e Biologia Evolutiva da UFSCar, São Carlos, Brazil (**DCBU**), and the Illinois Natural History Survey, Urbana-Champaign, USA (**INHS**). We also analyzed images and DNA sequences from some specimens in the Centre for Biodiversity Genomics, University of Guelph, Canada (**CBG**), images of material (holotypes and paratypes) in the Coleção Taxonômica do Departamento de Ecologia e Biologia Evolutiva (DCBU) da Universidade Federal de São Carlos (**UFSCAR**), Brazil, and photographed some specimens in the American Entomological Institute Collection (**AEIC**), Logan, Utah, USA. In total, we studied more than one thousand specimens. This material allowed us to deal with the entire fauna on hand at this time, but we anticipate tens more new species once portions of the Neotropics have been inventoried with the intensity already invested in ACG.

### ﻿Morphology and taxonomic characters

In this paper we diagnose and describe all the species using a combination of basic morphology (dichotomous key and brief diagnostic description), DNA barcodes (when available), biology, and distribution data, following suggestions outlined in Fernandez-Triana (2022).

Morphological terms and measurements mostly follow Huber and Sharkey (1993), Whitfield (1997), Karlsson and Ronquist (2012) and Fernandez-Triana et al. (2014). The abbreviations **T1**, **T2**, and **T3** are used for metasomal mediotergites 1, 2, and 3 respectively; and **L** and **W** refer to length and width, respectively. Following recommendations for the simplified and abbreviated use of morphological data in turbo taxonomy approaches (Fernandez-Triana 2022), the diagnostic descriptions for all the new species were based on a relatively small set of morphological characters (12), which can be quickly assessed and scored (Table 1).

**Table 1.** Morphological characters and their states, as used in the diagnostic description of the new species of *Alphomelon*.

Character	Character states (separated by semicolons)
White patch on gena	neither extending onto clypeus nor to occiput (Figs 65C, 85A, 85B); extending to occiput but not to clypeus (Figs 2B, 16B, 32B, 41B, 64B); extending to occiput and onto clypeus (Figs 18B, 19A, 31C, 37B, 68B)
Tegula/humeral complex color	white/yellow; yellow/yellow; yellow/brown; brown/brown
Mesonotum color	mostly orange-yellow (Figs 57F, 64H, 22F); mostly dark brown to black (Figs 1E, 2E, 3D, 42G)
Metasoma color	mostly orange-yellow (Figs 57E, 64G); with several tergites orange-yellow, some laterotergites and sternites yellow, rest mostly brown (Figs 8D, 18D, 19A, 22A, 22E, 31D, 67B, 67F, 77A, 77F, 77G, 78D); mostly dark brown to black but with some laterotergites and sternites yellow (Figs 15A, 18A, 21B, 21G, 34B, 34F); mostly black or dark brown (Figs 1E, 2E, 7C, 38A, 39A, 42C)
Tarsal claws spine number (hind leg)	0; 1; 2; 3; 4
Pterostigma shape	comparatively more elongate, its length ≥ 3.0× its central height and more triangular with its two lower margins more or less straight (Figs 3C, 6B, 7A, 24A, 48C); comparatively less elongate, its length ≤ 2.5× its central height and usually more rounded with at least one of its lower margins curved (Figs 11C, 18C, 29B)
T1 sculpture	entirely to mostly smooth (Figs 11G, 13E, 13F, 14C, 31D, 31E, 41D, 45E); weakly sculptured along margins (Figs 1D, 1E, 8E, 36F, 38D); strongly sculptured on at least apical half or more (Figs 2D, 2E, 3E, 75F, 76C)
T1 central ridge	absent (Figs 26E, 59B); faintly indicated by shallow depression (Figs 30D, 45E); marked by weak carina (Figs 31D, 31E, 46F, 78D, 78E); clearly marked by two raised carinae (Figs 1E, 3E, 17F, 32E); strongly marked by two raised carinae and strong costulae centrally (Figs 2D, 18D, 33E, 72E, 76C, 76E)
T2 sculpture	entirely to mostly smooth (Figs 1D, 4F, 6F, 8D, 12E, 14C); weakly sculptured along margins (Figs 21G, 22E, 22F, 27E, 29E, 32D); entirely to mostly strongly sculptured (Figs 2D, 3E, 16D, 18D)
Ovipositor sheaths length	shorter than the first segment of metatarsus (Figs 9A, 31A, 44A, 61A); longer than the first segment of metatarsus (Figs 2A, 15A, 16A, 21B, 25A)
Body length (mm)	as detailed in Fernandez-Triana et al. (2014)
Fore wing length (mm)	as detailed in Fernandez-Triana et al. (2014)

Some characters of particular relevance to *Alphomelon* were incorporated from the previous review of the genus by Deans et al. (2003). These characters are: a) the “petiolar ridge” or T1 central ridge (e.g., Figs 2E, 3E, 8E, 17F, 18E), which is a central, longitudinal carina on T1 usually extending from around half of the tergite length and ending close to the posterior margin; the carina can bifurcate posteriorly and the space between the two arms sometimes presents some transverse striations; b) the size and shape of white/pale coloration in the gena, which can extend to the occiput and sometimes onto the clypeus (e.g., Figs 5D, 9D, 12B, 18B, 19A, 31C, 60B); and c) the degree of angulation of the vein cu-a on the hind wing (e.g., Figs 5C, 14C, 19B, 20C; also see Deans et al. 2003).

In the Distribution section for each species, new country or state records are indicated by an asterisk (*). Complete and verbatim label details were provided only for holotypes. Paratypes and other specimens examined were listed only with basic information (country, repository, sex, and voucher codes). All information associated with those specimens can be accessed in the publicly available CNC database (https://www.cnc.agr.gc.ca/taxonomy/TaxonMain.php) and at dx.doi.org/10.5883/DS-ALPHOMEL; the dataset “DS-ALPHOMEL” in BOLD (https://www.boldsystems.org/index.php/MAS_Management_DataConsole?codes=DS-ALPHOMEL) also comprises details for those specimens with available DNA sequences.

### ﻿Molecular data

DNA barcoding was also used to characterize and recognize species. DNA extracts were obtained from single legs using a glass fiber protocol (Ivanova et al. 2006), and total genomic DNA was re-suspended in 30 μl of distilled water. The barcode region, a 658 base pairs (bp) region near the 5’ terminus of the COI gene, was amplified using standard primers following established protocols (e.g., see references in Fernandez-Triana et al. 2014).

The Barcode Index Number (BIN) was considered to approximately characterize species limits, following the BIN concept detailed in Ratnasingham and Hebert (2013).

All information for the sequences associated with each specimen barcoded (including primers and trace files) is available on the Barcode of Life Data System (BOLD) (Ratnasingham and Hebert 2007) under the dataset “DS-ALPHOMEL”. Two Neighbor Joining trees (with all *Alphomelon* sequences longer than 300 and 500 base pairs respectively) are provided in Suppl. material 1.

### ﻿Host data

Host data (Lepidoptera species) as well as wasp cocoon strategy (solitary/gregarious) were mostly taken from the website “Dynamic database for an inventory of the macrocaterpillar fauna, and its food plants and parasitoids in ACG databases” (http://janzen.sas.upenn.edu/caterpillars/database.lasso). Although many hosts are identified to species in that database, for many others the available information only included the Lepidoptera genus with an interim or provisional species name code. For example, “*Neoxeniades* Burns03” and “*Neoxeniades* Burns04” respectively mean species #3 and #4 identified by Burns as members of the genus *Neoxeniades*; these examples being relatively easy to understand. Other host species names have more complicated syntaxis; for example, “*Morys* lydeDHJ02” means species # 2 identified by Daniel H. Janzen within the genus *Morys*, supposedly related to but not the same as the species *Moryslyde* (the relationship shown by adding the identifier initials to the “*lyde*” name but the latter not written in italics to indicate it is a different species). Extreme cases include “hespJanzen01 Janzen60”, which means unidentified genus #1 in the Hesperiidae family identified by Janzen, with an unidentified species # 60, also identified by Janzen; in this case it should be interpreted as an unidentified genus and species of Hesperiidae. These conventions to record Lepidoptera hosts from ACG have been used in many scientific papers published during the past 10+ years.

### ﻿Digital imaging

The majority of the photos were taken by CB and JFT with a Keyence VHX-1000 Digital Microscope (Keyence Corporation, Japan), using a lens with a range of 10–130×; multiple images were taken of the structures through the focal plane and then combined to produce a single in-focus image using the software associated with the Keyence System. Some photographs by CB were taken with a Canon EOS-7D Mark 2 (G) (Canon Inc., Japan) using a super-macro lens Canon MP-65 with a Yongnuo professional flash speedlight flashlight installed on a modified microscope stand; multiple images (in raw format .CR2) were taken of a structure through the focal plane, converted to .dng with Adobe DNG converter, then corrected (brightness and contrast) in Adobe Bridge CS4, converted to .tiff images with Adobe Photoshop CS4 and finally combined to produce a single in-focus image using Zerene Stacker (http://zerenesystems.com/cms/stacker). Final images produced by CB and JFT were corrected using GIMP 2.10.12. Photos by EMS were taken with a 3MP Leica video camera and a Leica M205C stereomicroscope (Wetzlar®, Germany) running Leica Application Suite (LAS) software and focus-stacked using the same software. Images by SRS were produced using Leica Application Suite (Leica Microsystems) and image stacking software, Combine ZM and Zerene Stacker v. 1.04. Color digital photos taken by AMPD were captured through an MC170 HD video camera attached to a Leica M205C stereomicroscope and the Leica Application Suite v. 4.12. Images of wasp cocoons and larvae were taken by parataxonomists at ACG. All plates were prepared using Microsoft PowerPoint 2010 and saved as .TIF files.

Images of eight holotypes (of previously described species), deposited in the Natural History Museum (Smithsonian Institution, Washington DC, USA) were accessed through the Primary Type Specimens Catalog of the Department of Entomology Collections (https://collections.nmnh.si.edu/search/ento/). The downloaded images were later combined into plates. Those images were classified in that website as CC0, therefore making them available under the Creative Commons license CC0 1.0 license granting the right to share for personal and educational purposes under the fair use doctrine; in any case, we acknowledge the source of those images here.

## ﻿Results

With the 30 new species described below, *Alphomelon* now contains 49 species (Table 2). This more than doubles the previous total of 19 known species (Fernandez-Triana et al. 2020), in spite of a major taxonomic revision of the genus having been published relatively recently (Deans et al. 2003). Improved and more comprehensive collecting for the past few years, coupled with DNA barcoding, explain this significant increase in species richness. And the total reported here is far from being close to the expected species richness. We have seen in entomological collections many more specimens from South America that remain unstudied and appear to be undescribed. It is clear that the recent estimate of 50 species as the overall diversity of the genus (Fernandez-Triana et al. 2020) was a severe underestimate, and we now estimate the Neotropical species richness of *Alphomelon* to be close to 100 species, or twice what is presented in this paper.

**Table 2.** List of all *Alphomelon* species with their hosts, wasp cocoon, DNA data (BINs), and distribution by country. ACG- Area de Conservación Guanacaste, Costa Rica. New country or state records are indicated by *. The column BIN code details the BIN associated with a species (when available), or specifies when only partial barcodes or no molecular data are available. For countries where data was available (Canada, United States and Brazil) finer species distributions (by provinces, states, territories) is provided; abbreviations for Canada and the United States follow http://www.canadapost.ca/tools/pg/manual/PGaddress-e.asp; for Brazil states follow https://www.iso.org/obp/ui/#iso:code:3166:BR.

Species	Host	Wasp cocoons	BIN code	Distribution
*Alphomelonadrianguadamuzi*	*Niconiadesincomptus*,*Parphorusdecora*	Solitary	BOLD:AAR3562	Costa Rica (ACG)
*A.amazonas*	No data	No data	No data	Colombia
*A.andydeansi*	*Anthoptusepictetus*, *A.insignis*, *A.* Burns33, *Congachydaea*, *Corticeacorticea*, *Cymaenesodiliatrebius*,*C.* Burns01, *Cyneairma*, *Justinianorda*, *Morys* lydeDHJ02,*Nycteliusnyctelius*, *Parphorusdecora*, *Psoralis* Janzen38, *Synaptesalenus*, *Vehiliusvetula*, *Vettiusaurelius*	Solitary	BOLD:ADJ6568	Costa Rica (ACG)
*A.arecaphile*	*Carystoidesbasoches*,*Synalecynaxa*	Gregarious	BOLD:AAB1086	Brazil (PA), Costa Rica (ACG)
*A.brachymacher*	No data	No data	Partial barcode	Brazil (ES, MT, PA, SC), Colombia, Costa Rica, Ecuador, Peru
*A.brasiliensis*	No data	No data	No data	Brazil (MG, SP, RS).
*A.bromeliphile*	*Neoxeniadesluda*	Gregarious	BOLD:AAB5598	Costa Rica (ACG), Mexico
*A.calixtomoragai*	*Vettiusaurelius*	Solitary	BOLD:ADA5721	Costa Rica (ACG), Mexico
*A.carolinacanoae*	*Carystoidesescalantei*	Solitary	BOLD:ACE5969	Costa Rica (ACG)
*A.christerhanssoni*	*Aidesbrino*, *Carystinaaurifer*, *Dubiellabelpa*	Gregarious	BOLD:AAB0787	Costa Rica (ACG)
*A.citroloma*	No data	No data	Partial barcode	Argentina, Belize*, Bolivia, Brazil (PE, RJ, RO), Costa Rica, Ecuador, Panama, Paraguay, Peru*, Trinidad & Tobago, Venezuela
*A.conforme*	Unidentified hesperiine	Gregarious	No data	Brazil (RJ), Costa Rica, Venezuela
*A.crocostethus*	Unidentified hesperiine	Solitary	BOLD:AAZ9859	Argentina*, Bolivia, Brazil (DF*, ES, MG, PE*, RJ, RN*, SP*), Colombia, Dominican Republic*, Jamaica, Peru*, Puerto Rico
*A.cruzi*	No data	No data	No data	Brazil (MG)
*A.diniamartinezae*	*Niconiadesincomptus*	Gregarious	BOLD:AAE2209	Costa Rica (ACG)
*A.disputabile*	*Cymaenestrebius*, *Lerema* spp.	Solitary	BOLD:AAF3301	United States (KS, TX); Argentina, Belize, Bolivia, Brazil (ES, MT, PA, RJ, SC), Costa Rica, Cuba, Dominica, Ecuador, Grenada, Guatemala, Mexico, Panama, Paraguay, Peru*, Puerto Rico, Saint Vincent, Trinidad & Tobago, Venezuela.
*A.duvalierbricenoi*	*Methionopsisina*	Gregarious	BOLD:AAB4029	Costa Rica (ACG)
*A.eldaarayae*	*Neoxeniades* Burns03, *N.* Burns04	Gregarious	BOLD:AAE2229	Costa Rica (ACG)
*A.eliethcantillanoae*	*Nisoniadescastolus*	Solitary	BOLD:AAC7653	Costa Rica (ACG)
*A.gloriasihezarae*	*Cymaenesodiliatrebius*, *Morysmicythus*	Solitary	BOLD:AAE5720	Costa Rica (ACG), Mexico
*A.guillermopereirai*	*Mnasitheus* Janzen55, *Anthoptusepictetus*, *Anthoptusinsignis*, *Anthoptus* Burns33, *Corticeacorticea*, *Cymaenesodiliatrebius*	Solitary	BOLD:AAB8584 (partially)	Costa Rica (ACG)
*A.hazelcambroneroa*e	*Calpodesfusta*, *C.severus*, *Cyneaanthracinus*, *C.cynea*, *C.irma*, *C.megalops*, *C.* Burns02, *C.* Burns04, *C.* Burns05, *C.* Burns06, *C.* Burns11, *Rhinthonmolion*, *R.osca*	Gregarious	BOLD:AAA6775	Costa Rica (ACG)
*A.itatiaiensis*	No data	No data	No data	Brazil (RJ)
*A.josecortesi*	*Verticasubrufescens*	Gregarious	BOLD:AAB6733	Costa Rica (ACG)
*A.keineraragoni*	*Neoxeniadespluviasilva*, *N.* Burns03	Gregarious	BOLD:ABU7420	Costa Rica (ACG)
*A.luciarosae*	*Ebususebusus*	Gregarious	BOLD:ACJ4259	Costa Rica (ACG)
*A.manuelriosi*	*Corticealysias*, *Cymaenesodiliatrebius*, *Parphorusdecora*, *P.storax*	Solitary	BOLD:ABX0806	Costa Rica (ACG)
*A.melanoscelis*	Unidentified hesperiine	No data	BOLD:AAB8584 (partially)	Argentina*, Brazil (AL, MT), Costa Rica, Mexico, Venezuela
*A.mikesharkeyi*	*Parphorusdecora*, *Quasimellanaservilius*, *Quasimellana* Burns01	Gregarious	BOLD:AAJ2210	Costa Rica (ACG)
*A.nanosoma*	*Carystoidesbasoches*, *C.escalantei*, *C.hondura*, *C.orbius*, *C.* Burns01, *Cobalopsis* sp.(?)	Gregarious	BOLD:AAB9792	Brazil (MT), Costa Rica (ACG), Ecuador, Mexico, Panama, Trinidad & Tobago
*A.nigriceps*	*Calpodesethlius*	Solitary	Partial barcode	Argentina, Belize, Brazil (RO), Colombia, Cuba, Curacao*, Dominica, Grenada, Netherlands Antilles, Peru, Saint Lucia, Saint Vincent, Trinidad & Tobago, Venezuela
*A.osvaldoespinozai*	*Enosisimmaculata*, *Eutychideochus*, *Niconiadesgladys*, *N.incomptus*, *Oxynthescorusca*, *Parphorusdecora*, *Vettiuspica*, “hespJanzen01 Janzen60”	Solitary	BOLD:AAJ2207	Costa Rica (ACG)
*A.palomae*	No data	No data	No data	Brazil (RJ, SP)
*A.paramelanoscelis*	No data	No data	Partial barcode	Brazil (SC), Colombia
*A.paranigriceps*	No data	No data	No data	United States, (FL, GA, NC, SC, TX)
*A.paurogenum*	No data	No data	No data	Argentina, Chile
*A.petronariosae*	*Anthoptusepictetus*, *Congachydaea*, *Leremaliris*, *Moryslyde*, *M.micythus*, *Vehiliusvetula*, *Vehilius* Janzen03	Solitary	BOLD:ADA7564	Costa Rica (ACG)
*A.pyrrhogluteum*	No data	No data	No data	Argentina
*A.rhyssocercus*	No data	No data	No data	Argentina, Costa Rica, Ecuador, Panama, Peru, Trinidad & Tobago, Venezuela
*A.ricardocaleroi*	*Cyneacynea*, *C.megalops*, *C.* Burns06	Gregarious	BOLD:AAB7535	Costa Rica (ACG)
*A.rigoi*	No data	No data	BOLD:AAB8584 (partially)	Belize, Venezuela
*A.rostermoragai*	*Tigasissimplex*, *Vettiusaurelius*	Solitary	BOLD:ACB1223	Costa Rica (ACG)
*A.rugosus*	No data	No data	No data	Brazil (DF, RS, SP)
*A.sergioriosi*	*Calpodesethlius*, *Enosisangularis*, *Pericharesadela*, *Trombaxanthura*	Gregarious	BOLD:AAD2561	Costa Rica (ACG), Mexico
*A.simpsonorum*	Unidentified hesperiine	Solitary	Partial barcode	Brazil (PR, SC), Costa Rica, Paraguay
*A.talidicida*	*Talidessergestus*, *T.sinois*, *T.* Burns01, *T.* Burns02, *T.* Burns03, *T.* Burns04, *Thracidesphidon*	Gregarious	BOLD:AAA7259	Belize, Brazil (MT, PA, PE, SP), Colombia, Costa Rica (ACG), Ecuador, Guyana, Mexico, Panama, Peru, Trinidad & Tobago, Venezuela
*A.winniewertzae*	*Euphyes* spp.	Solitary	Partial barcode	Canada (ON, QC), United States (AR, DC, FL, KS, MA, MI, NC, OH, TN, TX, VA), Mexico (?)
*A.xestopyga*	*Calpodesesperi*, *C.ethlius*, *C.fusta*, *C.severus*, *Cobalopsisnero*, *Cyneairma*, *Cymaenesodiliatrebius*, *C.* Burns01, *Joannajoanna*, *Justinianorda*, *Moryslyde*, *M.micythus*, *M.valeriusvalda*, *M.* lydeDHJ01, *M.* lydeDHJ02, *Niconiadesincomptus*, *Parphorusdecora*, *Quintacannae*, *R.osca*, *Synaptesalenus*, *S.silius*, *Vettiusaurelius*	Gregarious	BOLD:AAA1634	Costa Rica (ACG)
*A.yanayacu*	No data	No data	No data	Ecuador

### ﻿Distribution

The genus is almost entirely Neotropical (48 species recorded from this region), but three species reach the Nearctic, with one of them extending as far north as 45° N in Canada (southern Ontario and southern Quebec). Based on the data from this paper (Table 2), one species (*Alphomelondisputabile*) is widely distributed in the New World (North to South America), one species (*A.winniewertzae*) is only found in North America, 12 are distributed in Central and South America, ten are restricted to South America, and 24 are only found in Central America (20 of them only known so far from ACG in Costa Rica). However, the latter seemingly restricted distribution is undoubtedly due to intensive inventory having only been conducted in the ACG ecosystems.

The highest species richness per country is found in Costa Rica with 38 recorded species, 32 of them in ACG, in great part because of the rearing of tens of thousands of wild-caught caterpillars there for the past 40 years. There are 17 species recorded from Brazil, nine from Mexico, eight from Argentina and Venezuela, seven from Ecuador and Peru, six from Colombia, and Trinidad and Tobago, and five from Belize and Panama. Only the total for Costa Rica can be considered reasonably close to the actual species richness, with most of the figures for South American countries being significantly lower than expected due to undersampling. When other parts of Costa Rica are treated as has been ACG since 1978, ACG might no longer seem to be exceptional.

### ﻿Biology

Our current knowledge of the host biology of the genus (Table 2) can be considered fairly satisfactory, as two-thirds of the species (33 spp, or 67%) have some host information (an additional three species have also been reared but from unidentified hesperiid hosts). Based mostly on ACG data (but also see Deans et al. (2003) for some host data from other areas), *Alphomelon* parasitizes exclusively Hesperiidae caterpillars in the subfamily Hesperiinae, mostly feeding on monocots in the families Arecaceae, Bromeliaceae, Cannaceae, Commelinaceae, Heliconiaceae and Poaceae. Only one species (*Alphomeloneliethcantillanoae*) has been reared from a host-specific caterpillar feeding on an herbaceous dicot (Asteraceae) growing among grasses. Most of the species parasitize either one or few (2–4) host species, usually within one or two hesperiid genera; however, seven (20% of all species with host data) have been recorded from several hosts from three or more (up to nine) different hesperiid genera. Among species with available data for their cocoons, roughly half are solitary (16) and half are gregarious (17). Cocoons of *Alphomelon* tend to be rather distinctive (Figs 91–103), with many species having a messy texture of coarse silk surrounding the cocoon (especially in solitary cocoons but also distinguishable in some gregarious cocoons).

### ﻿Molecular data

There is a relatively good library of DNA barcode sequences of *Alphomelon* in BOLD, with 1,409 sequences representing 37 public BINs as of June 2023 (http://www.boldsystems.org/index.php/Taxbrowser_Taxonpage?taxon=Alphomelon&searchTax=Search+Taxonomy). Of the 49 species dealt with in this paper, 31 (63%) have complete DNA barcodes sequences, another six (12%) have at least partial (= shorter) DNA barcodes and only 12 species (24%) have no molecular data currently available. DNA barcodes (and their BINs) were very useful in most cases (Fig. 104) for discriminating between *Alphomelon* species, with roughly an accuracy of 90%.

Exceptions included four species that are part of a complex related to *Alphomelonmelanoscelis* Deans, 2003, which shared the same BIN (BOLD:AAB8584). Three species of this complex (*A.guillermopereirai* sp. nov., *A.melanocelis*, and *A.paramelanoscelis* sp. nov.) were analyzed further. While they cannot be diagnosed at the BIN level, it was still possible to use diagnostic base pairs (or combinations of base pairs) to differentiate the species within that BIN. There were two diagnostic base pairs (or SNP = Single Nucleotide Polymorphisms) in the available sequences for those three species (Fig. 105). To describe these positions, we used numbers derived by aligning sequences to the cytochrome c oxidase subunit I (COI) region of the full mitochondrial reference genome of *Drosophilamelanogaster* (NC_024511.2). There is a synonymous transition at the third codon at aligned position 201 (A or G) that differentiates *A.melanocelis* from *A.guillermopereirai* and *A.paramelanoscelis* (however, there was a single specimen of *guillermopereirai* (DHJPAR0043086) that did not have the *guillermopereirai*-specific G, but rather the *paramelanoscelis*/*melanocscelis*-specific A). There is a non-synonymous T to A transversion at the first codon position at aligned position 334 that differentiates *A.paramelanoscelis* from both *A.melanocelis* and *A.guillermopereirai*. The combination of these two diagnostic characters in the barcode region can differentiate these three species that fall within the same BIN. It should be noted that interpreting DNA barcode data in this fashion is particularly sensitive to how well one understands the intraspecific variation in the barcode region (i.e., how many samples per species were included) (Fisher and Smith 2008). When there is good intraspecific representation, there is more confidence in the diagnoses, as with any set of character states.

### ﻿Diagnosis of the genus *Alphomelon* Mason, 1981

*Alphomelon* has the propodeum with complete areola and strong carinae; T1 very broad and usually with some sort of longitudinal carina centrally (petiolar ridge); hypopygium inflexible; ovipositor sheaths entirely setose and usually at least 0.5 as long as metatibia; and gena with a pale spot that is relatively large and very distinctive (Mason 1981; Deans et al. 2003). The latter feature gives the name to the genus (from Greek *Alphos* = white spot, *melon* = cheek) and in practice is the most distinctive character to recognize a member of this genus. Although that character is found sporadically (and rarely) in several other Microgastrinae genera, *Alphomelon* seems to be the only genus where it is present in all its known species. Species of *Alphomelon* can be distinguished from other Microgastrinae with a pale spot on the gena by their ovipositor sheaths being relatively long (much shorter in *Cotesia* Cameron, *Glyptapanteles* Ashmead, and *Protapanteles* Ashmead), mesoscutum anteriorly without strong notauli (strong notauli in *Prasmodon* Nixon), propodeum fully areolate (with strong median, longitudinal carina in *Pseudapanteles* Ashmead, complete or partial median longitudinal carina in *Sathon* Mason), metacoxa relatively large and metatibial spurs at least 0.5 length of first segment of metatarsus (metacoxa relatively small and metatibial spurs short, < 0.5 length of first segment of metatarsus in *Notogaster* Fernandez-Triana & Ward), and hypopygium inflexible and without pleats (almost always flexible and with several pleats in *Apanteles* Foerster and *Dolichogenidea* Viereck).

### ﻿Describing new species by combining morphological, molecular, and biological data while integrating previously described species based on less comprehensive data

The present paper was prepared after a relatively recent taxonomic revision of the genus was published 20 years ago. In that paper, Deans et al. (2003) provided a comprehensive treatment, mostly based on morphology but also incorporating some host data, with a total of 16 *Alphomelon* species extensively described (based on morphological characters) and illustrated (mostly drawings, with some black and white photos as well as some scanning electronic microscope photographs), as well as a dichotomous key. That paper provided a foundation for future work, but it was done right before the widespread use of DNA barcoding; thus, no species had associated molecular data. Likewise, two Brazilian species were described that same year (Shimabukuro and Penteado-Dias 2003), with no more than morphological data.

By contrast, the majority of the 30 new species described below have molecular (DNA barcodes), biological (host data), and morphological data available. Therefore, we faced the challenge to integrate all information to cover both sets of species.

In this paper we treat, diagnose, and describe all species using a combination of basic morphology (dichotomous key and a brief diagnostic description), DNA barcodes (when available), biology, and distribution data, following suggestions outlined in Fernandez-Triana (2022).

For the new dichotomous key, we used Deans et al. (2003) as a foundation and then added the new species, while also correcting some minor mistakes from the original key. But there were challenges and limitations when integrating legacy (= historical) and newly described species with disparate amounts of data. For example, some of the morphological characters used by Deans et al. (2003) were found to be either too variable (e.g., number of spines in tarsal claws), or difficult/subjective to assess (e.g., the extent of the white patch on gena, or degree or curvature of vein cu-a on the hind wing). Furthermore, some species (e.g., *A.citroloma*, *A.nanosoma*, *A.xestopyga*) each comprise complexes of morphologically cryptic species, still needing further study, and where morphology alone was insufficient.

DNA barcoding offered arguably the easiest and more standardized way to distinguish species. However, we did not succeed in obtaining DNA barcodes for all species, particularly those described before 2004 (see Table 2), with approximately one-third of all known species of *Alphomelon* not having any molecular data available. And while DNA barcoding seemed to be useful to discriminate among some very similar species (e.g., see couplets 36–39 in the key below), in other cases DNA barcoding alone failed to distinguish species (e.g., the *melanoscelis* complex).

The other sources of information were also limited. Host data and cocoon shape/structure were mostly known for ACG species, with very few records from other areas. And relying on species distribution only offered an incomplete picture, as most of South American countries remain to be studied with the same detail as ACG (i.e., with a sufficient number of specimens available as to draw solid inferences on species distribution).

Thus, there was no silver bullet to deal with these problems. Neither morphology nor DNA barcoding was sufficient on its own to delimit species properly. By integrating all available evidence, even if incomplete and uneven (as available data for every species is different) we hope to provide here a foundation for future and more comprehensive studies of *Alphomelon*. Incorporating more specimens, especially from South America, as well as more sequences will likely change some of the species concepts presented below.

### ﻿Key to female New World species of *Alphomelon*

Modified from Deans et al. (2003), incorporating all new species and additional morphological characters. When available, non-morphological information to help in recognizing species is added at the end of a couplet between brackets: a) Distribution (country), b) Biology (parasitoid cocoons solitary/gregarious; and caterpillar host records), c) DNA (BIN or partial barcodes). Males often can only be identified by DNA barcodes (when and where possible, by comparing sequences that are 500+ base pairs long).

**Table:** 

1	Mesosoma entirely to mostly orange-yellow (at most with part of propodeum, propleuron, mesosternum, and metapleuron dark brown to black) (Figs 22A, E, 23A, E, 57A, F, 64A, H); wings strongly to slightly infumated (Figs 22D, 57D, 64D); tarsal claws with one spine; white patch on usually gena reduced, not extending to post-genal part of occiput	**2**
–	Mesosoma entirely dark brown to black; wings variably colored but usually not infumated; tarsal claws variable; white patch on gena variable	**4**
2(1)	Entire body pale yellow or yellow-orange, except for black head and (sometimes) posterior 0.1–0.2 of metatibia and most of metatarsus brown to dark brown (Fig. 57A); wings only slightly infumated [Distribution: Argentina, Belize, Brazil, Colombia, Cuba, Curacao, Dominica, Grenada, Netherlands Antilles, Peru, Saint Lucia, Saint Vincent, Trinidad & Tobago, Venezuela. Biology. Solitary, reared from *Calpodesethlius*. DNA: Partial barcode]	***Alphomelonnigriceps* (Ashmead, 1900)**
–	Body with much darker coloration, usually with extensive areas dark brown to black (Figs 22A, 23A), if rarely body mostly dark orange-yellow then at least with brown coloration on humeral complex, propleuron (mostly), metapleuron (partially to entirely) and propodeum; wings strongly infumated	**3**
3(2)	T1 petiolar ridge comparatively very large, occupying posterior 0.7 of T1 length and running up to posterior margin of tergite, ridge divided posteriorly in two arms rather widely separated (Fig. 64G); T2 mostly strongly sculptured; head mostly reddish brown, especially face and frons; body mostly dark orange-yellow; wings very strongly infumated [Distribution: United States]	***Alphomelonparanigriceps* Fernandez-Triana & Shimbori, sp. nov.**
–	T1 petiolar ridge comparatively shorter, occupying posterior 0.5 or less of T1 length, and ending clearly before posterior margin of tergite, ridge divided posteriorly in two arms which are usually not as widely separate (Figs 22F, 23F); T2 mostly smooth; head mostly black; body color variable but very rarely mostly dark orange-yellow; wings infumated but not as strongly as above [Distribution: Argentina, Bolivia, Brazil, Colombia, Dominican Republic, Jamaica, Peru, Puerto Rico. Biology: Solitary, reared from unidentified hesperiid. DNA: BINBOLD:AAZ9859]	***Alphomeloncrocostethus* Deans, 2003**
4(1)	Metasoma paler colored, dorsally with some tergites orange or brown-yellow (Figs 18A, D, 31B, D, 67B, F, 77F, G) *and/or* with most laterotergites and sternites yellow (Fig. 34A, B)	**5**
–	Metasoma darker colored, mostly dark brown to black, if rarely most of T2 and small areas of T1 or T3 pale orange-brown then rest of metasoma dorsally and laterotergites and sternites dark brown to black, if rarely first three laterotergites and sternites yellow then rest of metasoma dark brown to black	**10**
5(4)	Metasoma mostly yellow or orange-yellow, except for small central area dark-brown on terga 6–8 and small black area on hypopygium (Fig. 67B, E, F) [Distribution: Argentina]	***Alphomelonpyrrhogluteum* Deans, 2003**
–	Metasoma dorsally mostly brown to black (at most with T1–T3 yellow or orange-yellow, usually less), laterally and ventrally with variable coloration on laterotergites, sternites and hypopygium, but at least with some areas dark brown to black	**6**
6(5)	Comparatively paler colored species, T1–T3 orange-yellow (Figs 31A, B, D, 77A, F, G); coxae entirely to mostly yellow, yellow-orange or pale yellow-brown, with metacoxa at least yellow-orange on posterior 0.5; central ridge on T1 weakly defined or almost absent	**7**
–	Comparatively darker colored species, T1–T3 brown or black (Figs 11F, G, 19B, 20E, 34F); coxae brown, dark brown or black, with metacoxa entirely dark brown to black; central ridge on T1 strongly defined (Figs 11G, 18D, E, 20E, 34F, G)	**8**
7(6)	White patch on gena not extending to clypeus, which is entirely brown; face entirely dark brown (Figs 77B, 78A); ovipositor sheaths comparatively longer, 1.20× as long as length of first segment of metatarsus and > 0.50× length of metatibia (Fig. 77A); T1 with petiolar ridge marked by weak carinae (Figs 77G, 78D, E) [Distribution: Brazil, Costa Rica, Paraguay. Biology: Solitary, reared from unidentified hesperiid. DNA: Partial barcodes]	***Alphomelonsimpsonorum* Deans, 2003**
–	White patch on gena extending to clypeus which is mostly white with only central 0.3 pale brown (Fig. 31C); face with pale orange-brown patch centrally, clearly paler than rest of brown face; ovipositor sheaths comparatively shorter, < 1.00× length of first segment of metatarsus and < 0.50× length of metatibia (Fig. 31A); T1 with petiolar ridge almost absent, marked by very weak carina or small depression (Fig. 31D, E) [Distribution: Costa Rica. Biology: Gregarious, reared from *Neoxeniades* Burns03, *N.* Burns04. DNA: BINBOLD:AAE2229]	***Alphomeloneldaarayae* Fernandez-Triana & Shimbori, sp. nov.**
8(6)	Tarsal claws with four spines; T2 mostly sculptured; hypopygium usually mostly yellow (brown to black on posterior 0.3 or less) (Fig. 34A) [Distribution: Costa Rica. Biology: Solitary, reared from *Mnasitheus* Janzen55, *Anthoptusepictetus*, *Anthoptusinsignis*, *Anthoptus* Burns33, *Corticeacorticea*, *Cymaenesodiliatrebius*. DNA: BINBOLD:AAB8584 (partially)]	***Alphomelonguillermopereirai* Fernandez-Triana & Shimbori, sp. nov.**
–	Tarsal claws with two spines; T2 smooth or weakly sculptured mostly near margins; hypopygium entirely to mostly dark brown to black (Figs 11A, 18A)	**9**
9(8)	Metasomal terga brown (Figs 11F, G); T2 smooth; T1 smooth and with petiolar ridge comparatively shorter, ~ 0.3 tergite length; fore wing vein 2RS very close to vein 2M (Fig. 11C), giving the impression that a small areolet (= second submarginal cell) is present, although the posterior end of that supposed areolet (which would correspond to veins r-m and/or 3RS) is not defined [Distribution: Brazil]	***Alphomelonbrasiliensis* Shimabukuro & Penteado-Dias, 2003**
–	Metasomal terga dark brown to black but usually with yellow markings on T3 (Fig. 18D); T2 mostly rugulose (rarely mostly smooth); T1 sculptured and with petiolar ridge comparatively longer, > 0.5 tergite length; fore wing vein 2RS not as close to vein 2M (Fig. 20C), therefore no small areolet (= second submarginal cell) is apparent [Distribution: Argentina, Belize, Bolivia, Brazil, Costa Rica, Ecuador, Panama, Paraguay, Peru, Trinidad & Tobago, Venezuela. DNA: Partial barcodes]	***Alphomeloncitroloma* Deans, 2003**
10(4)	Exserted portion of ovipositor sheaths shorter than first segment of metatarsus (Figs 9A, 13A, 14A, 44A, 45A, E)	**11**
–	Exserted portion of ovipositor sheaths as long or longer than first segment of metatarsus (e.g., Figs 16A, 29A, 30A, 33A, 46A, 50A, 61A, 65B, 73A, 84A, 90A)	**14**
11(10)	T2 comparatively narrow, ca. same width than T1 width (Fig. 45E); T2 subquadrate (its width at posterior margin just < 2.50× its central length, and T2 width posterior margin < 1.20× T2 width at anterior margin); white patch on gena not extending to clypeus (although clypeus has two small pale brown spots) (Fig. 45B); hind wing with vein cu-a more or less straight; tarsal claws with three or four spines (not clearly visible in examined specimens) [Distribution: Brazil, Costa Rica. Biology: Gregarious, reared from *Ebususebusus*. DNA: BINBOLD:ACJ4259]	***Alphomelonluciarosae* Fernandez-Triana & Shimbori, sp. nov.**
–	T2 comparatively broad, clearly broader than T1 width (Figs 8D, 12E, 13E, 14C); T2 transverse (its width at posterior margin just > 3.00× its central length, and T2 width posterior margin > 1.20× T2 width at anterior margin); white patch on gena usually extending to clypeus (Figs 8B, 9B, 12B, 44B); hind wing with vein cu-a strongly curved towards body; tarsal claws with four spines	**12**
12(11)	Face black; antennae dark brown; ovipositor < 0.50× length of first segment of metatarsus; ovipositor sheaths expanded apically (Fig. 8A) [Distribution: Brazil, Colombia, Costa Rica, Ecuador, Peru]	***Alphomelonbrachymacher* Deans, 2003**
–	Face either with pale brown spot centrally or mostly brown with some yellow spots laterally; antennae pale brown; ovipositor longer than 0.50× length of first segment of metatarsus; ovipositor sheaths not expanded apically	**13**
13(12)	Comparatively slightly darker-colored species, face usually mostly black with only central spot brown (Fig. 13B), T2 usually black to dark brown, anteromesoscutum entirely black (Fig. 13E); T2 more transverse (its width at posterior margin > 3.00× its central length) [Distribution: Costa Rica, Mexico. Biology: Gregarious, reared from *Neoxeniadesluda.* DNA: BINBOLD:AAB5598]	***Alphomelonbromeliphile* Deans, 2003**
–	Comparatively slightly paler-colored species, face mostly brown with some yellow spots laterally (Fig. 44B), T2 mostly reddish-brown to yellow-brown (Fig. 44F), anteromesoscutum with posterior margins reddish-brown; T2 less transverse (its width at posterior margin < 3.00× its central length) [Distribution: Costa Rica. Biology: Gregarious, reared from *Neoxeniadespluviasilva* and *Neoxeniades* Burns03. DNA: BINBOLD:ABU7420]	***Alphomelonkeineraragoni* Fernandez-Triana & Shimbori, sp. nov.**
14(10)	Hind legs mostly dark (Figs 42E, 46A, 48A, 49A, 61A, 72A, 73A, 90A), with metacoxa and metafemur black, dark brown or dark reddish-brown; metatibia usually with anterior 0.1–0.2 white but rest mostly dark orange, dark brown or black, if exceptionally metatibia mostly orange, then metafemur entirely dark; body size comparatively large, usually > 4.0 mm (and up to 5.2 mm), very rarely smaller	**15**
–	Hind leg coloration variable but metafemur never entirely dark and metatibia mostly pale colored (orange-yellow or yellow, with at most dark spot on posterior 0.1–0.2); body size variable but rarely > 4.0 mm	**20**
15(14)	Metatibia almost entirely orange-yellow, with only posterior 0.1 brown (Fig. 90A); smaller size, with body length and fore wing length 3.60–3.70 mm [Distribution: Ecuador]	***Alphomelonyanayacu* Fernandez-Triana & Shimbori, sp. nov.**
–	Metatibia entirely to mostly dark (dark orange, dark brown or black); bigger size, almost always > 4.0 mm, usually more	**16**
16(15)	Size comparatively larger, body length and fore wing at least 4.70 mm (4.70–5.20 mm); and tarsal claws with 3 or 4 spines; and ovipositor sheaths significantly longer (1.30–1.40×) than length of first segment of metatarsus; and tegula yellow and humeral complex brown (rarely both tegula and humeral complex brown) [Distribution: Argentina, Brazil, Costa Rica, Mexico, Venezuela. DNA: BINBOLD:AAB8584 (partially)]	***Alphomelonmelanoscelis* Deans, 2003**
–	Size comparatively smaller, body length and fore wing usually 4.20 mm or less (3.60–4.40 mm); *either* with tarsal claws with fewer than three spines; and/or ovipositor sheaths < 1.20× length of first segment of metatarsus; and/or tegula yellow and humeral complex of different coloration than above	**17**
17(16)	T2 smooth (Figs 46F, 60D, 61E, F)	**18**
–	T2 entirely to partially sculptured (Figs 42F, 72E, 73D)	**19**
18(17)	First three laterotergites and sternites paler colored (yellow or white-yellow) (Figs 60A, 61A, C); ovipositor sheaths ca. same length than first segment of metatarsus; pterostigma comparatively less elongate (i.e., its length ≤ 2.50× its central height) and usually more rounded (i.e., at least one of its lower margins is curved); tarsal claws with 3 or 4 spines; paler colored species with metatibia usually mostly orange-brown (only posterior 0.2 dark brown), humeral complex yellow, and coxae brown [Distribution: Brazil, Colombia. DNA barcoding: Partial sequence]	***Alphomelonparamelanoscelis* Fernandez-Triana & Shimbori, sp. nov.**
–	All laterotergites brown to dark brown (Fig. 46A); ovipositor sheaths longer (1.30–1.50×) than first segment of metatarsus; pterostigma comparatively more elongate (i.e., its length ≥ 3.00× its central height) and more triangular-shaped (i.e., its two lower margins more or less straight); tarsal claws with two spines; darker colored species with metatibia mostly dark brown to black, humeral complex brown, and all coxae dark brown to black [Distribution: Costa Rica. Biology: Solitary, reared from *Corticealysias*, *Cymaenesodiliatrebius*, *Parphorusdecora*, and *P.storax*. DNA barcoding: BINBOLD:ABX0806]	***Alphomelonmanuelriosi* Fernandez-Triana & Shimbori, sp. nov.**
19(17)	T2 strongly sculptured (Figs 72E, 73D), pterostigma comparatively less elongate (i.e., its length ≤ 2.50× its central height) and usually more rounded (i.e., at least one of its lower margins is curved) (Figs 72B, 73C); tarsal claws with three spines; ovipositor sheaths 1.10× as long as first segment of metatarsus [Distribution: Belize, Venezuela. DNA barcoding: BINBOLD:AAB8584 (partially)]	***Alphomelonrigoi* Fernandez-Triana & Shimbori, sp. nov.**
–	T2 only partially and much less strongly sculptured (Fig. 42F); pterostigma comparatively more elongate (i.e., its length ≥ 3.00× its central height) and more triangular-shaped (i.e., its two lower margins more or less straight) (Fig. 42D); tarsal claws with 1 or 2 spines; ovipositor sheaths 1.20–1.30× as long as first segment of metatarsus [Distribution: Costa Rica. Biology: Gregarious, reared from *Verticasubrufescens*. DNA barcoding: BINBOLD:AAB6733]	***Alphomelonjosecortesi* Fernandez-Triana & Shimbori, sp. nov.**
20(14)	White patch on gena neither extending onto clypeus nor onto occiput (90%), if extending to occiput then anteromesoscutum and head strongly punctate; tarsal claws with one spine; hind wing with vein cu-a angled posteriorly towards body	**21**
–	White patch on gena extending onto occiput and often onto clypeus (75%); tarsal claws variable; hind wing with vein cu-a variable	**22**
21(20)	Wings usually infumated (Fig. 65D); head, anteromesoscutum, and scutellar disc weakly punctate; posterior 0.2 of metatibia black (Figs 65B, I); labrum black [Distribution: Argentina, Chile]	***Alphomelonpaurogenum* Deans, 2003**
–	Wings hyaline (Fig. 83C); head, anteromesoscutum and scutellar disc strongly punctate; metatibia entirely orange (Figs 83B, 85A); labrum yellow [Distribution: Canada, Mexico, United States. Biology: Solitary, reared from *Euphyes* spp. DNA barcoding: Partial barcodes]	***Alphomelonwinniewertzae* Deans, 2003**
22(20)	Tarsal claws simple (without spines) or with only one spine	**23**
–	Tarsal claws with 2–4 spines	**30**
23(22)	Pterostigma comparatively less elongate (i.e., its length ≤ 2.50× its central height) and usually more rounded (i.e., at least one of its lower margins is curved); hind wing with vein cu-a angled posteriorly towards body (Figs 27C, 28C, 29B, 33C); tegula yellow, humeral complex at least partially brown (often, although not always) (Fig. 27F); fore wing venation mostly brown	**24**
–	Pterostigma comparatively more elongate (i.e., its length ≥ 3.00× its central height) and more triangular-shaped (i.e., its two lower margins more or less straight) (Figs 4C, 5C, 6D, 7A); hind wing with vein cu-a usually straight or at most slightly curved or angled posteriorly towards body; usually (but not always) tegula paler than humeral complex (i.e., tegulae whitish and humeral complex yellow, or tegula pale yellow and humeral complex pale brown); coloration of fore wing venation variable	**25**
24(23)	Metafemur mostly orange-yellow but with posterior 0.2–0.5 brown (Figs 27A, 28A, 29A); T2 from smooth to slightly rugulose (Figs 27D, 28D, 29E) [Distribution: Argentina, Belize, Bolivia, Brazil, Costa Rica, Cuba, Dominica, Ecuador, Grenada, Guatemala, Mexico, Panama, Paraguay, Peru*, Puerto Rico, Saint Vincent, Trinidad & Tobago, United States, Venezuela. Biology: Solitary, reared from *Lerema* spp. and *Cymaenestrebius.* DNA barcoding: BINBOLD:AAF3301]	***Alphomelondisputabile* (Ashmead, 1900)**
–	Metafemur almost entirely orange-yellow with only posterior 0.1 or less with brown spot (Fig. 33A, D); T2 rather strongly sculptured with striae (Fig. 33D) [Distribution: Costa Rica. Biology: Solitary, reared from *Cymaenesodiliatrebius* and *Morysmicythus*. DNA barcoding: BINBOLD:AAE5720]	***Alphomelongloriasihezarae* Fernandez-Triana & Shimbori, sp. nov.**
25(23)	Fore wing venation mostly brown to dark brown; T2 entirely or mostly smooth (Figs 4F, 5E, 6F, 17F, 17G)	**26**
–	Fore wing venation paler with at least veins M+CU, 1CU, 1M, (RS+M) a yellow-white or pale yellow-brown (Figs 2C, 16C, 32C, 76C); T2 entirely sculptured (Figs 2D, 16D, 76B)	**27**
26(25)	T1 mostly rugulose on posterior 0.5 (Figs 4F, 5E, 6F); hind wing with vein cu-a straight [Distribution: Brazil, Costa Rica; Biology: Gregarious, reared from *Carystoidesbasoches* and *Synalecynaxa*. DNA barcoding: BINBOLD:AAB1086]	***Alphomelonarecaphile* Deans, 2003**
–	T1 mostly smooth (Fig. 17F, G); hind wing with vein cu-a curved [Distribution: Costa Rica. Biology: Gregarious, reared from *Aidesbrino*, *Carystinaaurifer* and *Dubiellabelpa*. DNA barcoding: BINBOLD:AAB0787]	***Alphomelonchristerhanssoni* Fernandez-Triana & Shimbori, sp. nov.**
27(25)	Tegula yellow and humeral complex brown; metafemur with dark spot on posterior 0.25 (Fig. 2A); metatibia with dark spot on posterior 0.2; metatibia spines white-yellow; metatarsus almost entirely dark brown (except for anterior 0.3 of first metatarsus segment orange) [Distribution: Colombia]	***Alphomelonamazonas* Fernandez-Triana & Shimbori, sp. nov.**
–	Tegula whitish, humeral complex yellow; metafemur with dark spot on posterior 0.10–0.15; metatibia orange, without any dark spot posteriorly; metatibia spines orange, same color as metatibia; metatarsus either entirely orange or mostly pale brown but with first segment orange on anterior 0.6–0.7 and last segment entirely yellow-orange	**28**
28(27)	T1 parallel-sided and around 2.00× as long as wide on posterior margin (Fig. 16D, E); ovipositor sheaths comparatively longer, 0.75× metatibia length (Fig. 16A); metatarsus mostly light brown but with first segment orange on anterior 0.6–0.7 and last segment entirely yellow-orange (Fig. 16A) [Distribution: Costa Rica. Biology: Solitary, reared from *Carystoidesescalantei*. DNA barcoding: BINBOLD:ACE5969]	***Alphomeloncarolinacanoae* Fernandez-Triana & Shimbori, sp. nov.**
–	T1 broadening towards posterior margin, < 1.50× as long as wide on posterior margin; ovipositor sheaths comparatively shorter, around half metatibia length; metatarsus entirely orange	**29**
29(28)	T1 mostly to entirely smooth (although with strongly defined petiolar ridge); ovipositor sheaths comparatively shorter, 0.50× metatibia length and ca. same length (1.03×) than first segment of metatarsus (Fig. 32A); slightly smaller size, body length 3.9 mm, fore wing length 3.9 mm [Distribution: Costa Rica. Biology: Solitary, reared from *Nisoniadescastolus*. DNA barcoding: BINBOLD:AAC7653]	***Alphomeloneliethcantillanoae* Fernandez-Triana & Shimbori, sp. nov.**
–	T1 coarsely sculptured on posterior 0.3–0.5 (in addition to strongly defined petiolar ridge); ovipositor sheaths comparatively longer, > 0.60× metatibia length and much longer (1.30–1.40×) than first segment of metatarsus (Fig. 76A); slightly larger size, body length almost always > 4.0 mm, fore wing length almost always > 4.1 mm [Distribution: Costa Rica. Biology: Solitary, reared from *Calpodesethlius*, *Enosisangularis*, *Pericharesadela* and *Trombaxanthura*. DNA barcoding: BINBOLD:AAD2561]	***Alphomelonsergioriosi* Fernandez-Triana & Shimbori, sp. nov.**
30(22)	Tegula black to dark brown; T1 with central ridge strongly bifurcating at posterior end, its arms comparatively widely separated [Distribution: Brazil, Costa Rica, Venezuela. Biology: Gregarious, reared from unidentified hesperiid on Cannaceae and Poaceae]	***Alphomelonconforme* (Muesebeck, 1958)**
–	Tegula pale colored (translucent or yellow); T1 with central ridge not strongly bifurcating at posterior end, its arms comparatively narrowly separated	**31**
31(30)	T1 strongly costate centrally (Figs 3C, E, 69B, D, E, 75F); T2 rugose, raised medially	**32**
–	T1 not strongly costate; T2 rugulose or nitid	**34**
32(31)	Flagellomeres black; tegula yellow (Fig. 3D); mesopleuron mostly smooth (at most with shallow and sparse punctures on anterior half); tarsal claws with three spines; males with pterostigma centrally white with brown margins and entire metasoma black [Distribution: Costa Rica. Biology: Solitary, reared from *Anthoptusepictetus*, *A.insignis*, *Anthoptus* Burns33, *Congachydaea*, *Corticeacorticea*, *Cymaenesodiliatrebius*, *Cymaenes* Burns01, *Cyneairma*, *Justinianorda*, *Morys* lydeDHJ02, *Nycteliusnyctelius*, *Parphorusdecora*, *Psoralis* Janzen38, *Synaptesalenus*, *Vehiliusvetula* and *Vettiusaurelius*. DNA barcoding: BINBOLD:ADJ6568]	***Alphomelonandydeansi* Fernandez-Triana & Shimbori, sp. nov.**
–	Flagellomeres brown to dark brown; tegula usually brown; mesopleuron mostly rugose or with deep punctures; tarsal claws with two spines; males (only known for *A.rhyssocercus*) with pterostigma entirely brown and T2 and T3 orange-brown	**33**
33(32)	White patch on gena extending to occiput and onto clypeus (sometimes clypeus yellow laterally, with only central third brown-black) (Fig. 68B); T2 and T3 brown to pale brown, T4+ brown (Fig. 69D); pro- and mesocoxae reddish-brown, metacoxa reddish brown to dark brown (Fig. 69A); mesopleuron with deep punctures [Distribution: Argentina, Costa Rica, Ecuador, Panama, Peru, Trinidad & Tobago, Venezuela]	***Alphomelonrhyssocercus* Deans, 2003**
–	White patch on gena not extending to occiput or clypeus (clypeus entirely black) (Fig. 75B); entire metasoma dorsally black (Fig. 75F); coxae black (Fig. 75A, E); mesopleuron rugose [Distribution: Brazil]	***Alphomelonrugosus* Shimabukuro & Penteado-Dias, 2003**
34(31)	T1 with central ridge present (Figs 1E, 25C, 38D, 58E, 71E, 74D, E, 80D, 82F)	**35**
–	T1 only with inconspicuous depression centrally (Figs 26E, F, 52E, 54E, 55G)	**44**
35(34)	Pterostigma comparatively more elongate (i.e., its length ≥ 3.00× its central height) and more triangular-shaped (i.e., its two lower margins more or less straight) (Figs 1C, 25A, 70B)	**36**
–	Pterostigma comparatively less elongate (i.e., its length ≤ 2.50× its central height) and usually more rounded (i.e., at least one of its lower margins is curved) (Figs 40C, 52C, 80D, 82D)	**41**
36(35)^1^	Metafemur darker, with posterior 0.5 dark brown (Fig. 74A) [Distribution: Brazil, Costa Rica. Biology: Solitary, reared from *Tigasissimplex* and *Vettiusaurelius*. DNA barcoding: BINBOLD:ACB1223]	***Alphomelonrostermoragai* Fernandez-Triana & Shimbori, sp. nov.**
–	Metafemur paler, with only posterior 0.1–0.2 dark brown	**37**
37(36)	T1 comparatively much broader posteriorly than anteriorly (posterior margin 1.50× as long as anterior margin, T1 median length 1.15× its maximum width) (Fig. 25E); and tegula and humeral complex yellow (Fig. 25B) [Distribution: Brazil]	***Alphomeloncruzi* Shimbori** & **Fernandez-Triana, sp. nov.**
–	T1 comparatively much less broad posteriorly (posterior margin < 1.30× as long as anterior margin, T1 median length 1.30–1.60× its maximum width); and tegula different color than humeral complex, if tegula and humeral complex yellow then T1 median length at least 1.50× its maximum width	**38**
38(37)	T1 comparatively slightly wider (its median length 1.30–1.40× its maximum width); tegula different color than humeral complex (Figs 1E, 58E)	**39**
–	T1 comparatively slightly narrower (its median length 1.50–1.60× its maximum width); tegula and humeral complex yellow (Figs 38D, 71E)	**40**
39(38)	Tegula white-yellow, paler colored than humeral complex (which is dark yellow to yellow-brown) (Fig. 58E); all coxae black (Fig. 58A) [Distribution: Costa Rica. Biology: Solitary, reared from *Enosisimmaculata*, *Eutychideochus*, *Niconiadesgladys*, *N.incomptus*, *Oxynthescorusca*, *Parphorusdecora*, *Vettiuspica* and an unidentified hesperiid with provisional name “hespJanzen01 Janzen60”. DNA barcoding: BINBOLD:AAJ2207]	***Alphomelonosvaldoespinozai* Fernandez-Triana & Shimbori, sp. nov.**
–	Tegula pale brown, darker than yellow humeral complex (Fig. 1E); coxae usually reddish brown [Distribution: Costa Rica. Biology. Gregarious, reared from *Niconiadesincomptus* and *Parphorusdecora*. DNA barcoding: BINBOLD:AAR3562]	***Alphomelonadrianguadamuzi* Fernandez-Triana & Shimbori, sp. nov.**
40(38)	Tarsal claws with 2 or 3 very small spines which are difficult to see [Distribution: Costa Rica. Biology: Gregarious, reared from *Cyneacynea*, *C.megalops* and *C.* Burns06. DNA barcoding: BINBOLD:AAB7535]	***Alphomelonricardocaleroi* Fernandez-Triana & Shimbori, sp. nov.**
–	Tarsal claws with 3 or 4 spines which are clearly visible [Distribution: Costa Rica. Biology: Gregarious, reared from *Calpodesfusta*, *C.severus*, *Cyneaanthracinus*, *C.cynea*, *C.irma*, *C.megalops*, *C.* Burns02, *C.* Burns04, *C.* Burns05, *C.* Burns06, *C.* Burns11, *Rhinthonmolion*, and *R.osca*. DNA barcoding: BINBOLD:AAA6775]	***Alphomelonhazelcambroneroae* Fernandez-Triana & Shimbori, sp. nov.**
41(35)	Ovipositor width 0.3 width of first segment of metatarsus; hind wing with vein cu-a strongly angled at midpoint towards body [Distribution: Belize, Brazil, Colombia, Costa Rica, Ecuador, Guyana, Mexico, Panama, Peru, Trinidad & Tobago, Venezuela. Biology: Gregarious, reared from *Talidessergestus*, *T.sinois*, *T.* Burns01, *T.* Burns02, *T.* Burns03, *T.* Burns04 and *Thracidesphidon*. DNA barcoding: BINBOLD:AAA7259]	***Alphomelontalidicida* (Wilkinson, 1931)**
–	Ovipositor width 0.5 width of first segment of metatarsus; hind wing with vein cu-a slightly angled around median section (of vein)	**42**
42(41)	Metafemur entirely yellow (Figs 40A, 41A); laterotergites dark brown; procoxa pale brown to yellow, metacoxa usually with ventro-apical yellow spot (very small to covering most of coxa) [Distribution: Brazil]	***Alphomelonitatiaiensis* Shimbori & Fernandez-Triana, sp. nov.**
–	Metafemur with dark brown spot on posterior 0.1–0.25 *and/or* first laterotergites yellow; all coxae dark brown	**43**
43(42)	Tarsal claws with two spines; first three laterotergites yellow (Fig. 15A); posterior 0.1 of T3 pale yellow-brown, rest of T3 dark brown (Fig. 15F); metafemur with dark brown spot on posterior 0.1; tegula and humeral complex same yellow color (Fig. 15E); pterostigma slightly more elongated (3.00× as long as its maximum height) [Distribution: Costa Rica. Biology: Solitary, reared from *Vettiusaurelius*. DNA barcoding: BINBOLD:ADA5721]	***Alphomeloncalixtomoragai* Fernandez-Triana & Shimbori, sp. nov.**
–	Tarsal claws with one spine; all laterotergites dark brown (Fig. 66A); T3 entirely dark brown (Fig. 66D); metafemur with dark brown spot on posterior 0.25 (Fig. 66A); tegula white, paler than yellow humeral complex (Fig. 66E); pterostigma slightly less elongated (2.80× as long as its maximum height) [Distribution: Costa Rica. Biology: Solitary, reared from *Anthoptusepictetus*, *CongachydaeaLeremaliris*, *Moryslyde*, *M.micythus*, *Vehiliusvetula*, and *Vehilius* Janzen03. DNA barcoding: BINBOLD:ADA7564]	***Alphomelonpetronariosae* Fernandez-Triana & Shimbori, sp. nov.**
44(34)	Metafemur entirely orange-yellow; metasoma dorsally entirely dark brown (Fig. 59A, E) [Distribution: Brazil]	***Alphomelonpalomae* Shimbori & Fernandez-Triana, sp. nov.**
–	Metafemur with dark brown to black spot on posterior 0.1 (spot usually larger) (Figs 26E, 56A); metasoma often with T2 and/or T3 partially paler (pale brown, pale reddish brown, yellow-brown) (Figs 30D, 52E, 53E, 56B)	**45**
45(44)	White patch on gena usually not extending onto clypeus (Figs 26A, 30B); T1 mostly nitid with petiolar ridge represented by slight depression (Figs 26E, F, 30D); usually hind wing vein cu-a evenly curved towards body *and* tarsal claws with two spines and ovipositor width 0.5 width of first segment of metatarsus (if hind wing with vein cu-a strongly curved medially and tarsal claws with three spines and ovipositor width 0.3 width of first segment of metatarsus, then body size smaller, ~ 2.7 mm)	**46**
–	White patch on gena extending onto lateral portions of clypeus; T1 at least somewhat rugulose, slightly punctate and with central ridge represented by raised bump with slight depression; usually hind with wing vein cu-a sharply angled at midpoint and tarsal claws with 3 or 4 spines and ovipositor width 0.3 width of first segment of metatarsus (if hind wing vein cu-a evenly curved towards body and/or tarsal claws with two spines then body size larger, 3.0–4.0 mm)	**48**
46(45)	Metafemur brown on posterior 0.5 (Fig. 26C, D); tarsal claws with three spines; ovipositor width 0.3 width of first segment of metatarsus; hind wing with vein cu-a strongly curved medially [Distribution: Costa Rica. Biology: Gregarious, reared from *Niconiadesincomptus*. DNA barcoding: BINBOLD:AAE2209]	***Alphomelondiniamartinezae* Fernandez-Triana & Shimbori, sp. nov.**
–	Metafemur brown on posterior 0.1–0.2 (Figs 30A, 52A, 53A, 54A); tarsal claws with two spines; ovipositor width 0.5 width of first segment of metatarsus; hind wing with vein cu-a evenly curved towards body	**47**
47(46)	Body size slightly larger (3.3–3.4 mm); T2 paler (orange-yellow or pale reddish-brown) and contrasting with rest of tergites (mostly dark brown to black) (Fig. 30D) [Distribution: Costa Rica. Biology: Gregarious, reared from *Methionopsisina*. DNA barcoding: BINBOLD:AAB4029]	***Alphomelonduvalierbricenoi* Fernandez-Triana & Shimbori, sp. nov.**
–	Body size slightly smaller (<3.0 mm); T2 (pale brown to rarely orange-yellow) usually less contrasting or not contrasting at all with rest of tergites (mostly brown to pale brown or rarely orange-brown) (variation shown in Figs 52E, 53E, 54E, 55F, 56B) [Distribution: Brazil, Costa Rica, Ecuador, Mexico, Panama, Trinidad & Tobago. Biology: Gregarious, reared from *Carystoidesbasoches*, *C.escalantei*, *C.hondura*, *C.orbius*, *Carystoides* Burns01, and *Cobalopsis* sp. (questionable). DNA barcoding: BINBOLD:AAB9792]	***Alphomelonnanosoma* Deans, 2003**
48(45)	Tegula and humeral complex comparatively paler (white-yellow) (Fig. 51C, F); metafemur with comparatively very small dark spot on posterior 0.1 or less (Fig. 51A); fore wing with many veins pale (yellow-brown to yellow-white), including veins M+CU, 1M, (RS+M)a, (RS+M)b and R1 (Fig. 51C); hind wing with vein cu-a curved medially; T2 comparatively narrower when compared with T1 (T2 width at posterior margin < 1.5× T1 width at posterior margin) [Distribution: Costa Rica. Biology: Gregarious, reared from *Parphorusdecora*, *Quasimellanaservilius* and *Quasimellana* Burns01. DNA barcoding: BINBOLD:AAJ2210]	***Alphomelonmikesharkeyi* Fernandez-Triana & Shimbori, sp. nov.**
–	Tegula and humeral complex comparatively darker (yellow) (Figs 87F, 89A); metafemur with comparatively larger dark spot on posterior 0.3 (Figs 86A, 87A, 88A, 89C); fore wing with most veins dark (dark brown to pale brown) (Figs 86D, 87D, 88D, 89A); hind wing with vein cu-a usually sharply angled at midpoint towards body; T2 comparatively wider when compared with T1 (T2 width at posterior margin > 1.5× T1 width at posterior margin) [Distribution: Costa Rica. Biology: Gregarious, reared from *Calpodesesperi*, *C.ethlius*, *C.fusta*, *C.severus*, *Cobalopsisnero*, *Cyneairma*, *Cymaenesodiliatrebius*, *C.trebius*, *C.* Burns01, *Joannajoanna*, *Justinianorda*, *Moryslyde*, *M.micythus*, *M.valeriusvalda*, *M.* lydeDHJ01, *M.* lydeDHJ02, *Parphorusdecora*, *Quintacannae*, *Rhinthonmolion*, *R.osca*, *Synaptesalenus*, *S.silius*, *Vettiusaurelius*. DNA barcoding: BINBOLD:AAA1634]	***Alphomelonxestopyga* Deans, 2003**

### ﻿Taxonomic treatment of species, in alphabetical order

#### ﻿ Alphomelon adrianguadamuzi

Taxon classificationAnimaliaHymenopteraBraconidae

Fernandez-Triana & Shimbori sp. nov.

7CB4C610-41A9-5AB4-87E5-A1667B8A4ADD

https://zoobank.org/703277DC-6B5C-4D32-B66F-882D77BB9D21

Figs 1A–E , 91A

##### Type material.

***Holotype*.** Costa Rica • Female, CNC; Alajuela, Area de Conservación Guanacaste, Brasilia, Gallinazo, 11°01'05.70"N, 85°22'19.16"W, 360m; 9.I.2012; ex. *Parphorusdecora*; coll. Minor Carmona; Voucher code: DHJPAR0047257; Host voucher code: 12-SRNP-65054.

##### Distribution.

Costa Rica (ACG).

##### Biology.

Solitary, reared from *Niconiadesincomptus* and *Parphorusdecora*.

##### DNA barcoding.

BINBOLD:AAR3562.

##### Etymology.

Named in honor of Sr. Adrián Guadamuz in honor of his decades of teamwork in the ACG parataxonomist team.

##### Diagnostic description.

White patch on gena: extending to occiput but not to clypeus. Tegula/humeral complex color: brown/yellow. Mesonotum color: mostly dark brown to black. Metasoma color: mostly black or dark brown. Tarsal claws spines: 3. Pterostigma shape: comparatively less elongate, its length ≤ 2.5× its central height and usually more rounded with at least one of its lower margins curved. T1 sculpture: weakly sculptured along margins. T1 central ridge: clearly marked by two raised carinae. T2 sculpture: entirely to mostly smooth. Ovipositor sheaths length: longer than first segment of metatarsus. Body length: 3.6 mm. Fore wing length: 3.6 mm.

**Figure 1.**
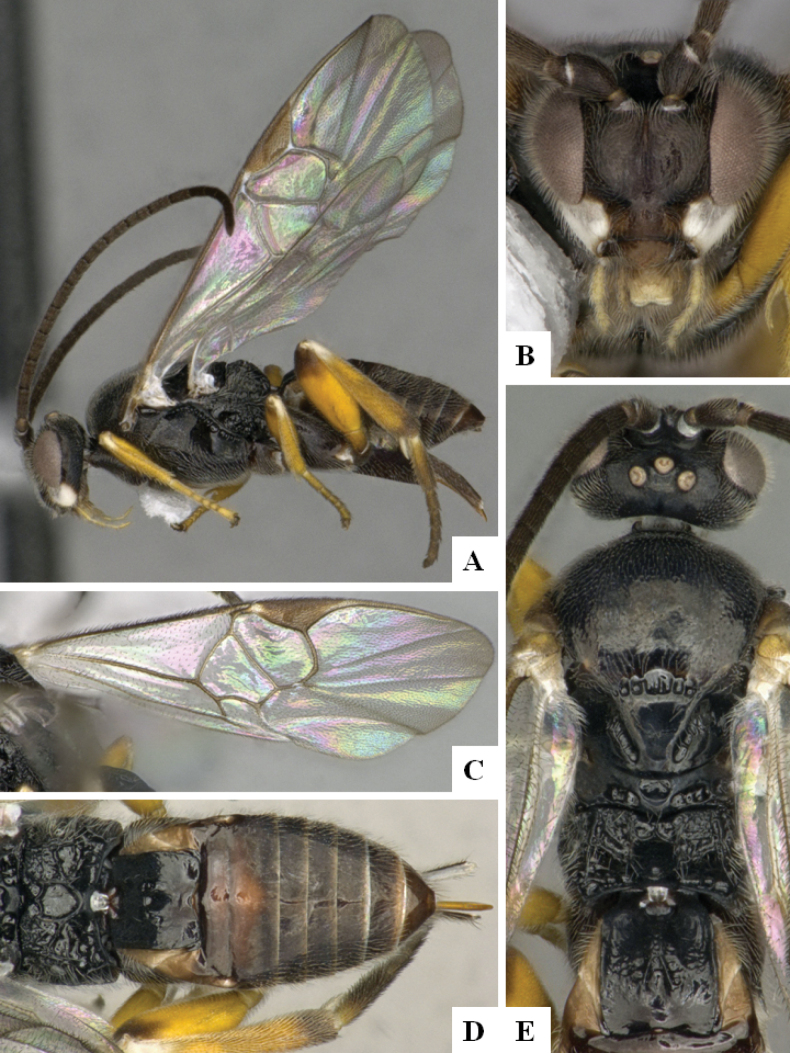
*Alphomelonadrianguadamuzi* Fernandez-Triana & Shimbori holotype female DHJPAR0047257 **A** habitus, lateral **B** head, frontal **C** fore wing **D** propodeum and metasoma, dorsal **E** head and mesosoma, dorsal.

#### ﻿ Alphomelon amazonas

Taxon classificationAnimaliaHymenopteraBraconidae

Fernandez-Triana & Shimbori sp. nov.

D3C55EC4-E5CB-502F-833E-3C5E628DB072

https://zoobank.org/A239BCD6-BD74-4C0F-9E1E-1CAF9D3E2D4A

Fig. 2A–E

##### Type material.

***Holotype*.** Colombia • Female, CNC; Amazonas, Leticia, 04°12'34.20"S, 69°56'49.53"W; 19–26.II.1974; Malaise Trap; Voucher code: CNC1066010.

##### Distribution.

Colombia.

##### Biology.

No data.

##### DNA barcoding.

Not available.

##### Etymology.

Named after the Colombian department Amazonas, the capital of which is the type locality, Leticia. The species name also indirectly refers to the Amazon River (in Spanish also spelled as Amazonas), which borders the city of Leticia, as an appreciation of the extraordinary diversity found in that area.

##### Diagnostic description.

White patch on gena: extending to occiput but not to clypeus. Tegula/humeral complex color: yellow/brown. Mesonotum color: mostly dark brown to black. Metasoma color: mostly black or dark brown. Tarsal claws spines: 1. Pterostigma shape: comparatively more elongate, its length ≥ 3.0× its central height and more triangular-shaped with its two lower margins more or less straight. T1 sculpture: strongly sculptured on at least apical half or more. T1 central ridge: strongly marked by two raised carinae and strong costulae centrally. T2 sculpture: entirely to mostly strongly sculptured. Ovipositor sheaths length: longer than first segment of metatarsus. Body length: 4.1 mm. Fore wing length: 3.7 mm.

**Figure 2.**
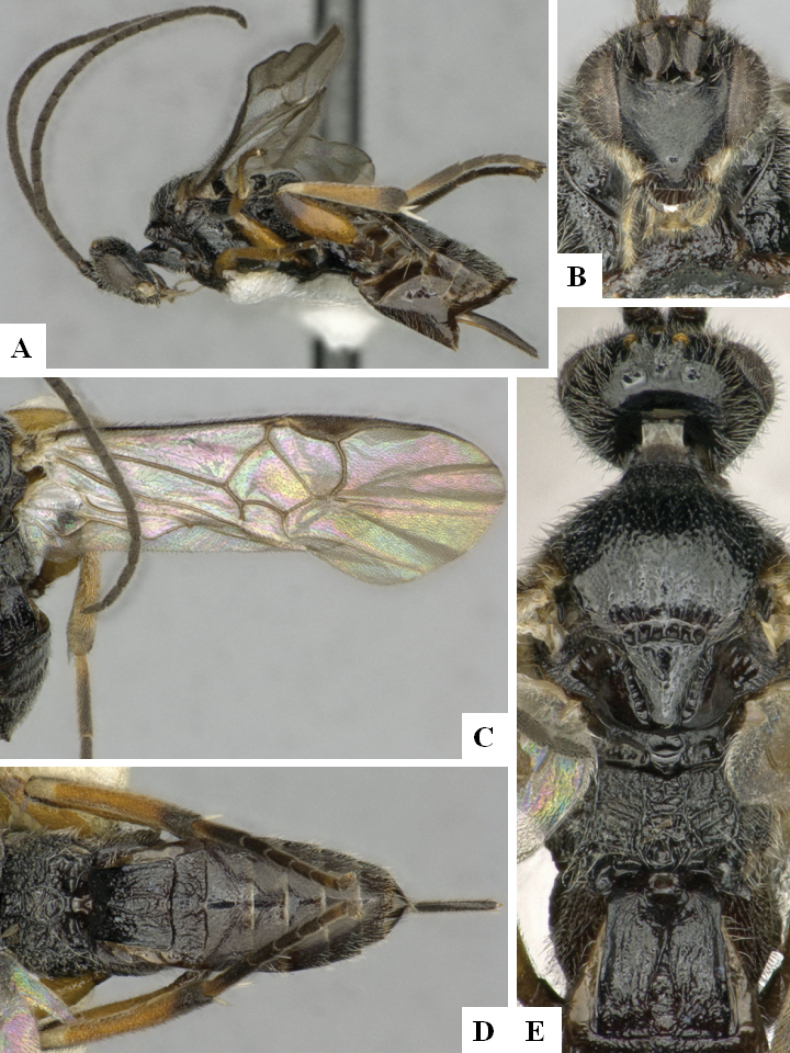
*Alphomelonamazonas* Fernandez-Triana & Shimbori holotype female CNC1066010 **A** habitus, lateral **B** head, frontal **C** fore wing **D** propodeum and metasoma, dorsal **E** mesosoma and T1, dorsal.

#### ﻿ Alphomelon andydeansi

Taxon classificationAnimaliaHymenopteraBraconidae

Fernandez-Triana & Shimbori sp. nov.

34034635-D4F5-53D9-B001-3CF91956098F

https://zoobank.org/DDBF4E97-0082-47AD-8399-DCA888B7BC0E

Figs 3A–F , 91B

##### Type material.

***Holotype*.** Costa Rica • Female, CNC; Guanacaste, Area de Conservación Guanacaste, Sector Santa Rosa, Area Administrativa, 10°50'15.50"N, 85°37'07.36"W, 295m; 25.XII.2008; Malaise Trap; coll. Daniel Janzen & Winifred Hallwachs; Voucher code: DHJPAR0031609.

***Paratypes*.** Costa Rica• 17 females, 3 males, CNC; DHJPAR0031655, DHJPAR0031618, DHJPAR0031647, DHJPAR0031619, DHJPAR0043085, DHJPAR0031612, DHJPAR0031613, DHJPAR0031643, DHJPAR0031611, DHJPAR0031615, DHJPAR0025868, DHJPAR0047217, DHJPAR0031617, DHJPAR0047175, DHJPAR0049243, DHJPAR0049244, DHJPAR0049254, DHJPAR0049260, DHJPAR0049250, DHJPAR0055291.

##### Distribution.

Costa Rica (ACG).

##### Biology.

Solitary, reared from *Anthoptusepictetus*, *A.insignis*, *A.* Burns33, *Congachydaea*, *Corticeacorticea*, *Cymaenesodiliatrebius*, *Cymaenes* Burns01, *Cyneairma*, *Justinianorda*, *Morys* lydeDHJ02, *Nycteliusnyctelius*, *Parphorusdecora*, *Psoralis* Janzen38, *Synaptesalenus*, *Vehiliusvetula* and *Vettiusaurelius*, all feeding on Poaceae.

##### DNA barcoding.

BINBOLD:ADJ6568.

##### Etymology.

Named in honor of Dr. Andrew (Andy) Deans in honor of his effort to work on the morphological taxonomy of ACG, and as a pioneer in the taxonomic study of *Alphomelon*, before the tool of DNA barcoding for identification became widely available.

##### Diagnostic description.

White patch on gena: extending to occiput and onto clypeus. Tegula/humeral complex color: yellow/yellow. Mesonotum color: mostly dark brown to black. Metasoma color: mostly dark brown to black but with some laterotergites and sternites yellow. Tarsal claws spines: 2 or 3. Pterostigma shape: comparatively less elongate, its length ≤ 2.5× its central height and usually more rounded with at least one of its lower margins curved. T1 sculpture: strongly sculptured on at least apical half or more. T1 central ridge: clearly marked by two raised carinae. T2 sculpture: entirely to mostly strongly sculptured. Ovipositor sheaths length: longer than first segment of metatarsus. Body length: 3.5–4.3 mm. Fore wing length: 3.6–4.2 mm.

**Figure 3.**
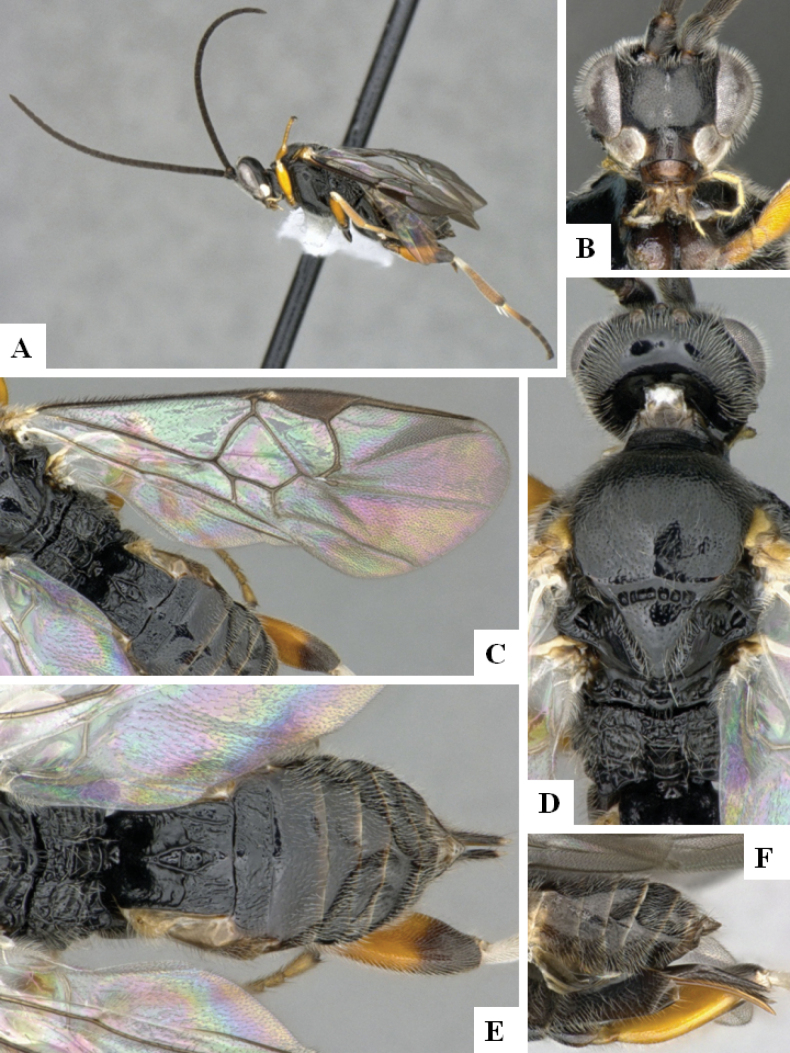
*Alphomelonandydeansi* Fernandez-Triana & Shimbori holotype female DHJPAR0031609 **A** habitus, lateral **B** head, frontal **C** wings **D** mesosoma, dorsal **E** propodeum and metasoma, dorsal **F** ovipositor, lateral.

#### ﻿ Alphomelon arecaphile

Taxon classificationAnimaliaHymenopteraBraconidae

Deans, 2003

367B6121-5F1B-5222-A774-AE91424A4943

**Figure 4.**
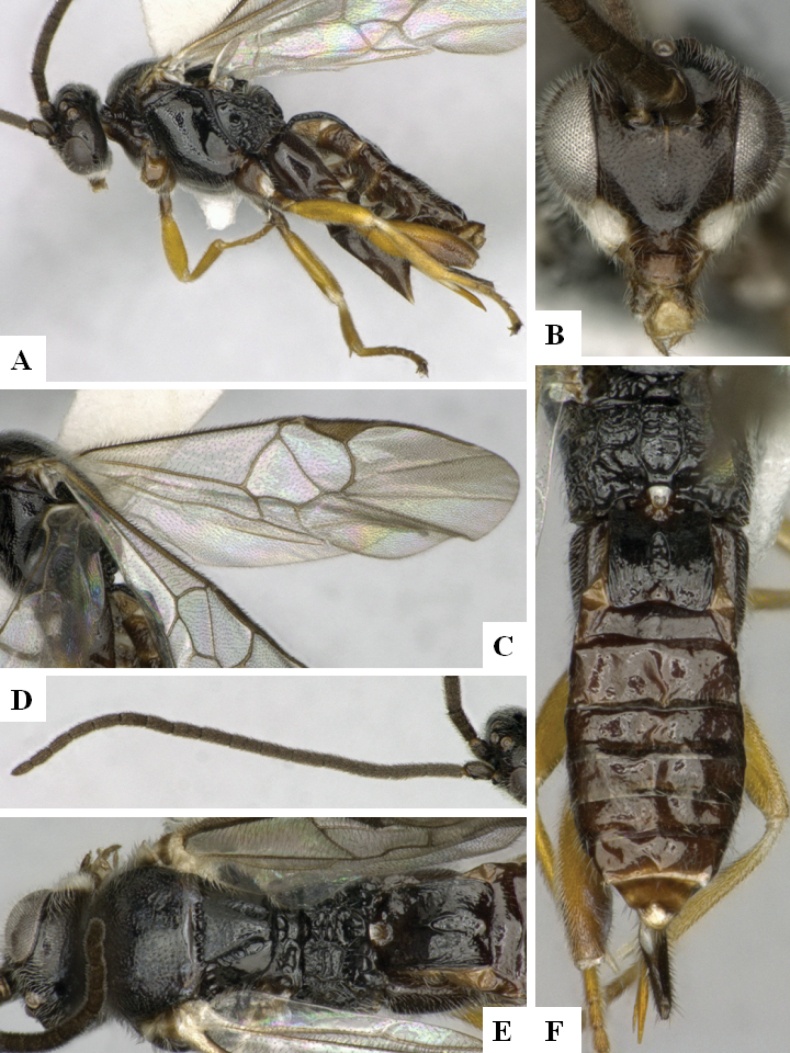
*Alphomelonarecaphile* Deans female CNC280512 **A** habitus, lateral **B** head, frontal **C** wings **D** antenna, lateral **E** mesosoma, dorsal **F** propodeum and metasoma, dorsal.

**Figure 5.**
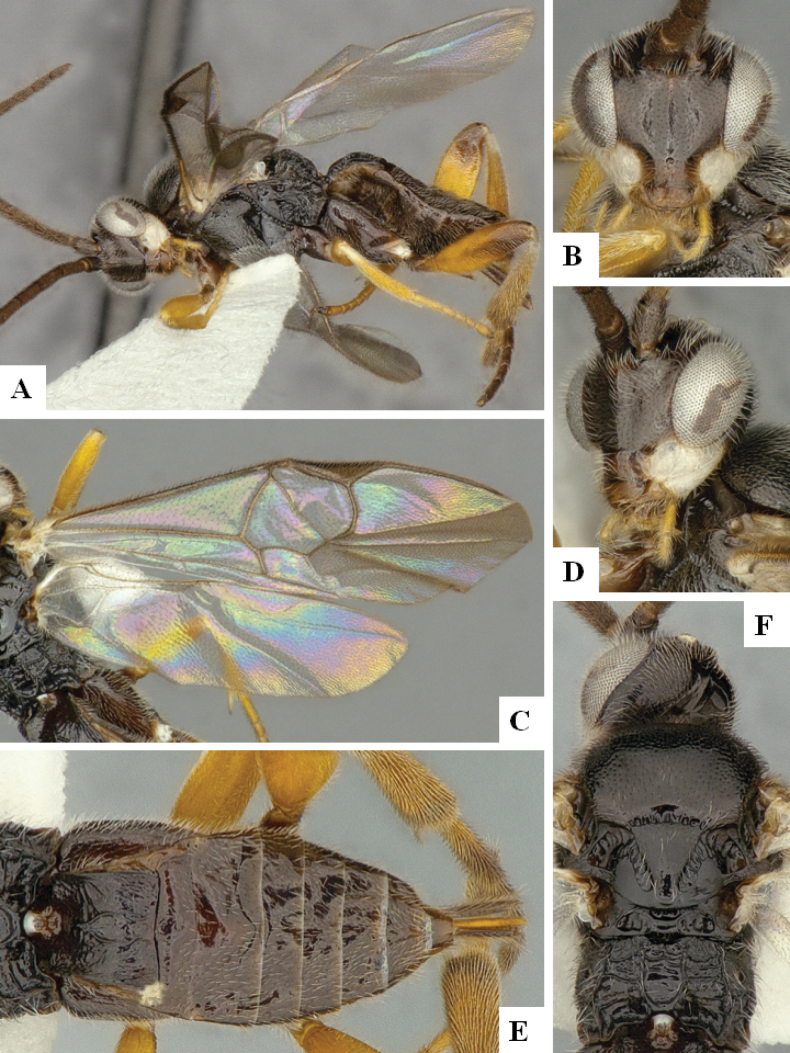
*Alphomelonarecaphile* Deans female CNC704666 **A** habitus, lateral **B** head, frontal **C** wings **D** head, fronto-lateral **E** metasoma, dorsal **F** mesosoma, dorsal.

**Figure 6.**
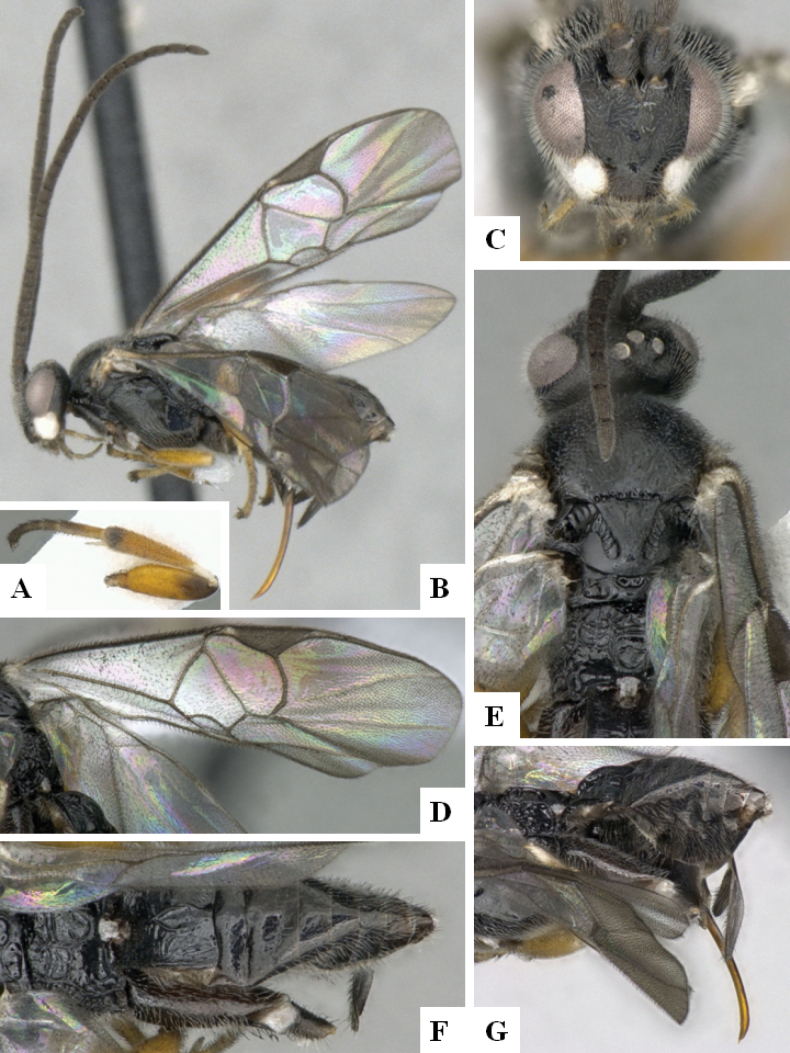
*Alphomelonarecaphile* Deans female DHJPAR0053712 **A** hind leg **B** habitus, lateral **C** head, frontal **D** wings **E** mesosoma, dorsal **F** propodeum and metasoma, dorsal **G** ovipositor, lateral.

**Figure 7.**
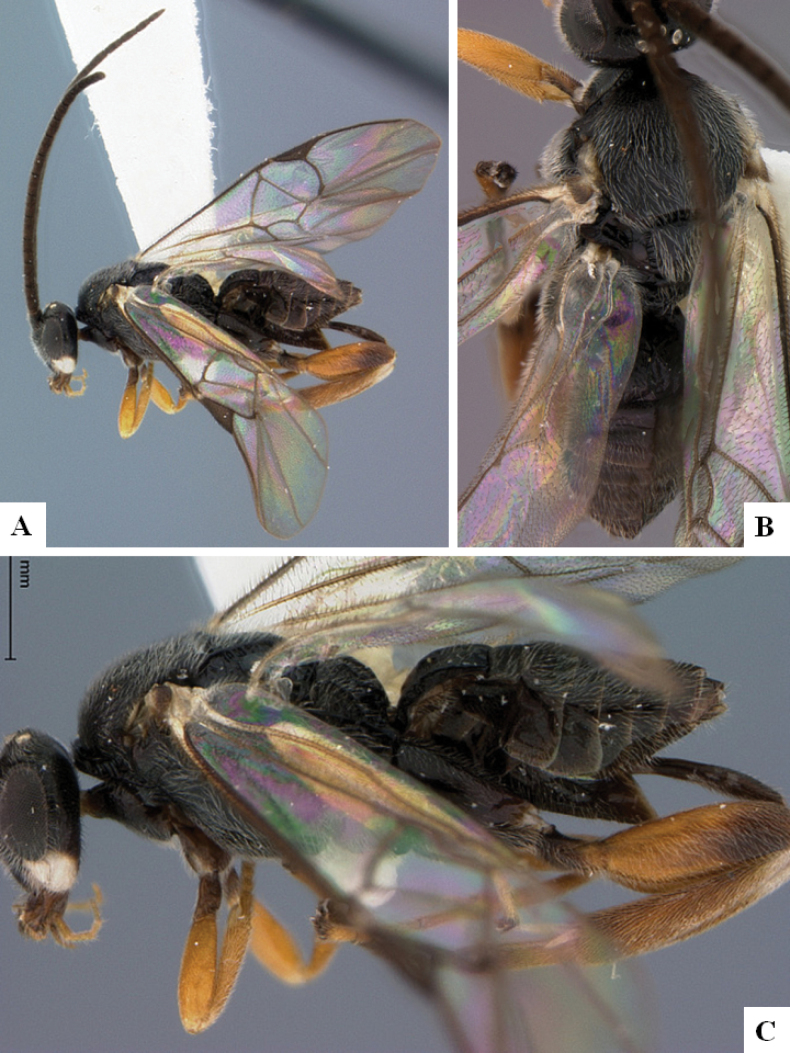
*Alphomelonarecaphile* Deans holotype female USNMENT00828294 **A** habitus, lateral **B** habitus, dorsal **C** close-up of habitus, lateral.

Figs 4A–F , 5A–F , 6A–G , 7A–C , 92A

##### Distribution.

Brazil (PA), Costa Rica (ACG); collected in intermediate altitude rainforest sites.

##### Biology.

Gregarious, reared from *Carystoidesbasoches* and *Synalecynaxa*.

##### DNA barcoding.

BINBOLD:AAB1086.

##### Other specimens examined.

(14 females, 4 males). DHJPAR0050978, CNC1802029, CNC1802030, CNC1802031, CNC1802032, CNC1802033, CNC1802034, CNC1802035, CNC1802036, DHJPAR0053712, DHJPAR0026445, DHJPAR0047100, CNC280512, CNC704668, CNC704667, CNC704664, CNC704665, CNC704666.

##### Notes.

This species was keyed out by Deans et al. (2003) in an incorrect way, as it has one spine on the tarsal claws but it was keyed out through the second half of their couplet 11, which stated “tarsal claws with 2–4 spines” (Deans et al. 2003: 7). Here we have keyed out the species based on the correct assessment of this character. A few specimens may have some veins in the fore wing more pale in color, but otherwise they fit very well with the rest of the characters (including biology, molecular and other morphological features).

#### ﻿ Alphomelon brachymacher

Taxon classificationAnimaliaHymenopteraBraconidae

Deans, 2003

B4E1A65C-7EC8-5DD0-9175-1148556AD802

**Figure 8.**
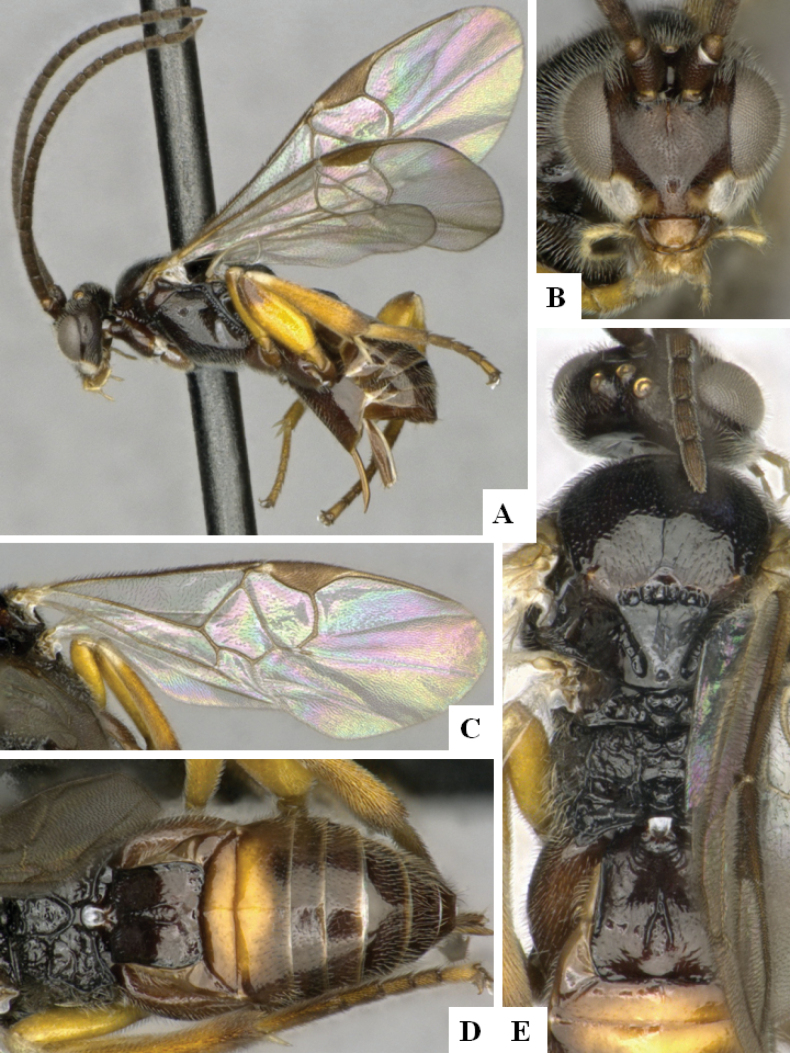
*Alphomelonbrachymacher* Deans female CNCHYM 00006 **A** habitus, lateral **B** head, frontal **C** wings **D** propodeum and metasoma, dorsal **E** mesosoma, dorsal.

**Figure 9.**
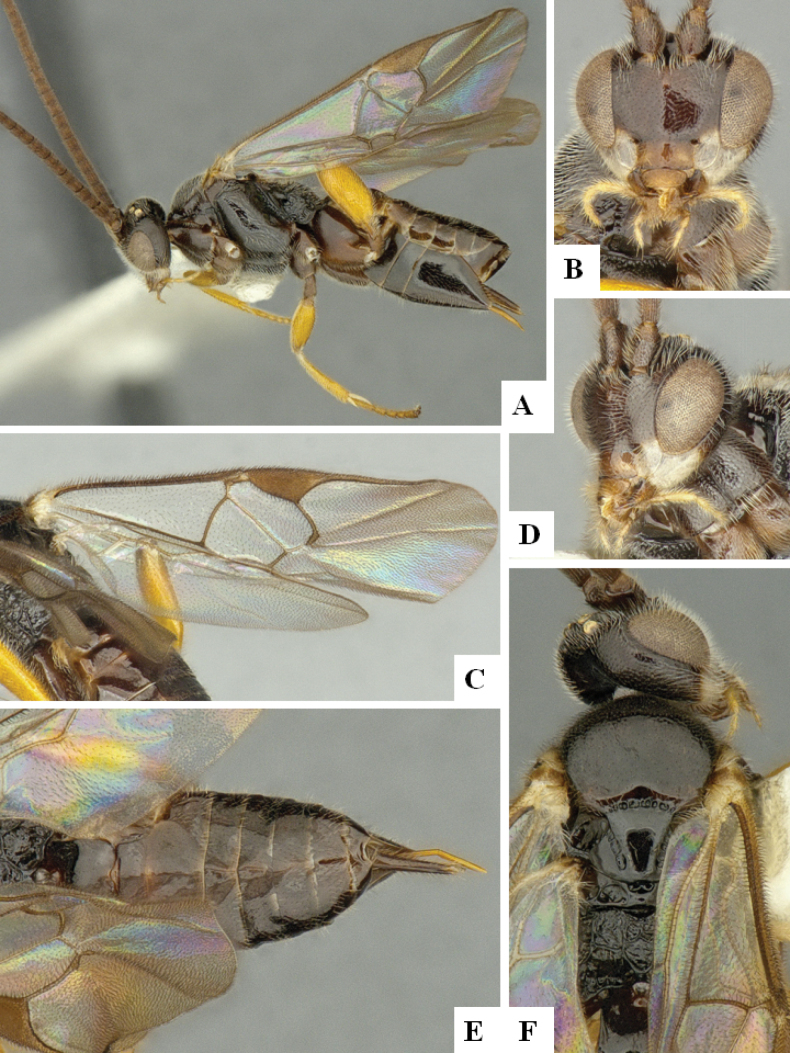
*Alphomelonbrachymacher* Deans female CNCHYM 00010 **A** habitus, lateral **B** head, frontal **C** wings **D** head, fronto-lateral **E** metasoma, dorsal **F** mesosoma, dorsal.

**Figure 10.**
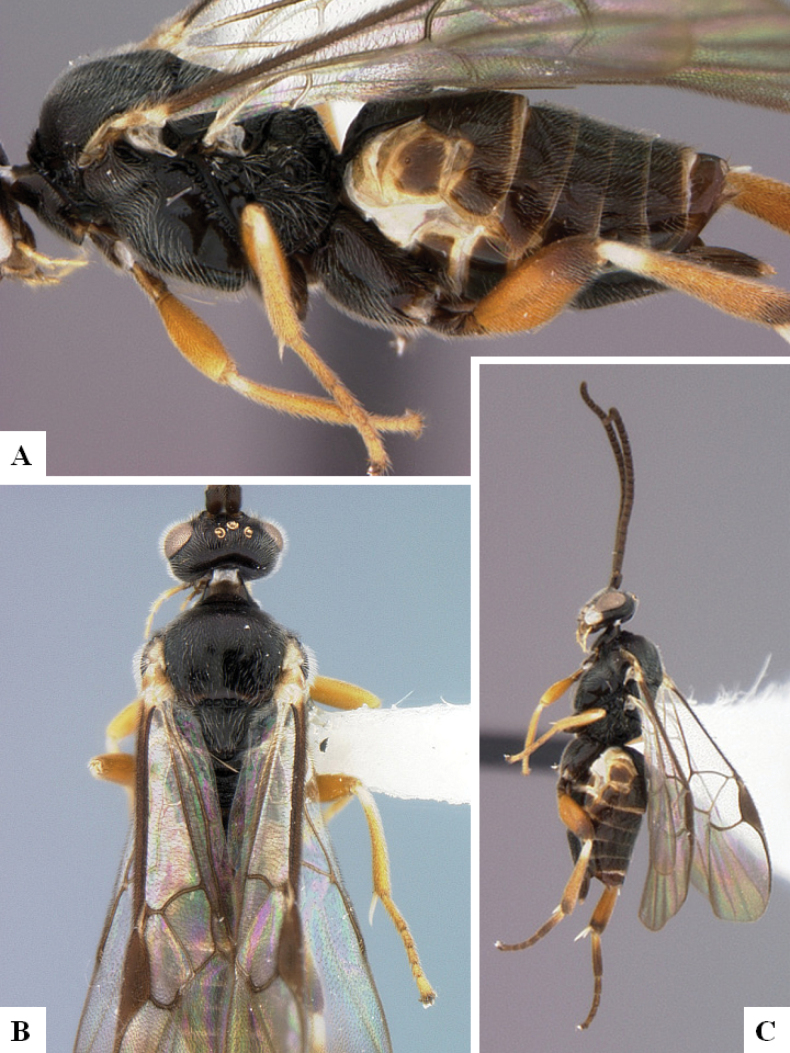
*Alphomelonbrachymacher* Deans holotype female USNMENT00828295 **A** close-up of habitus, lateral **B** head and mesosoma, dorsal **C** habitus, lateral.

Figs 8A–E , 9A–F , 10A–C

##### Distribution.

Brazil (ES, MT, PA, SC), Colombia, Costa Rica, Ecuador, Peru.

##### Biology.

No data.

##### DNA barcoding.

Four partial barcodes (164 bp).

##### Other specimens examined.

(8 females). CNCHYM 00010, CNC280513, CNC704683, CNCHYM 00006, CNCHYM 00007, CNCHYM 00008, CNCHYM 00009, CNC704684.

##### Notes.

Some of the specimens we have seen (e.g., Fig. 8A) have the ovipositor longer and of different coloration than what was discussed by Deans et al. (2003).

#### ﻿ Alphomelon brasiliensis

Taxon classificationAnimaliaHymenopteraBraconidae

Shimabukuro & Penteado-Dias, 2003

786B5E0F-AE31-581A-917C-880D7BD4BA47

Fig. 11A–G

##### Distribution.

Brazil (MG, SP, RS).

##### Biology.

Unknown.

##### DNA barcoding.

Not available.

##### Notes.

**Figure 11.**
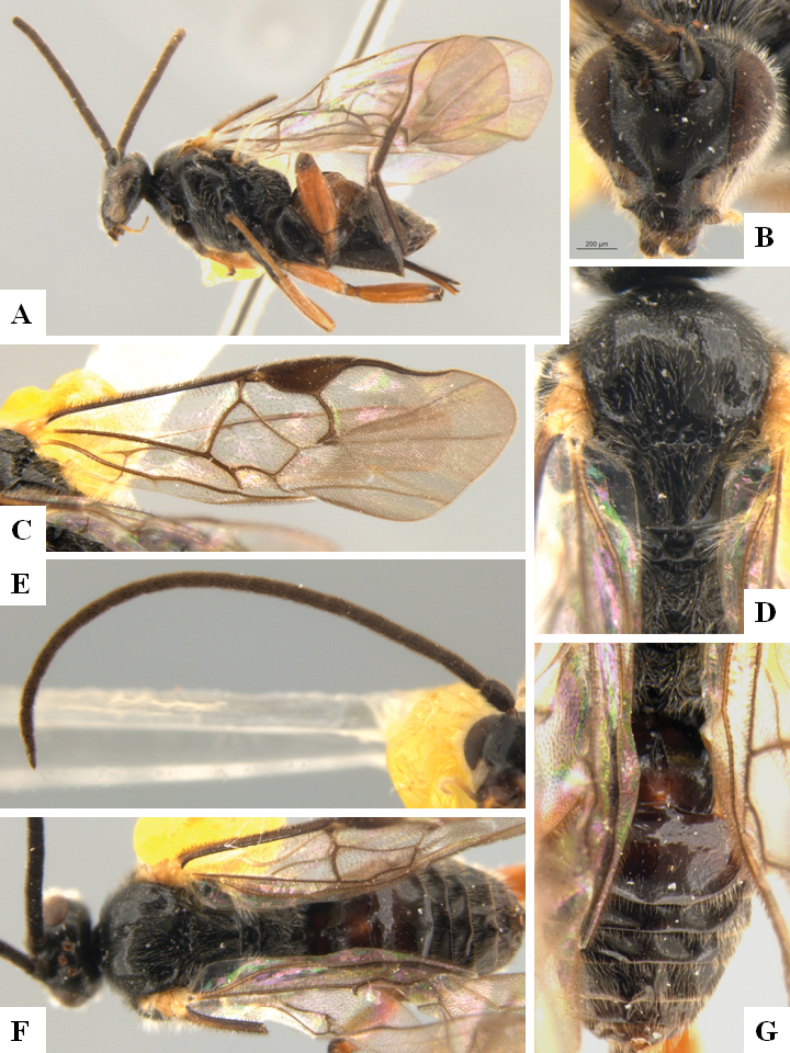
*Alphomelonbrasiliensis* Shimabukuro & Penteado-Dias holotype female DCBU513116 **A** habitus, lateral **B** head, frontal **C** fore wing **D** mesosoma, dorsal **E** antenna, lateral **F** habitus, dorsal **G** metasoma, dorsal.

The fore wing vein 2RS is very close to vein 2M (Fig. 11C), giving the impression that a small areolet (= second submarginal cell) is present; although the posterior end of that supposed areolet (which would correspond to veins r-m and/or 3RS) is not defined. This feature (of having an almost completely defined areolet) is unique among all species of *Alphomelon* that we studied.

#### ﻿ Alphomelon bromeliphile

Taxon classificationAnimaliaHymenopteraBraconidae

Deans, 2003

5FFFA33F-2935-51D8-BCAA-A21AEB9E8EF9

**Figure 12.**
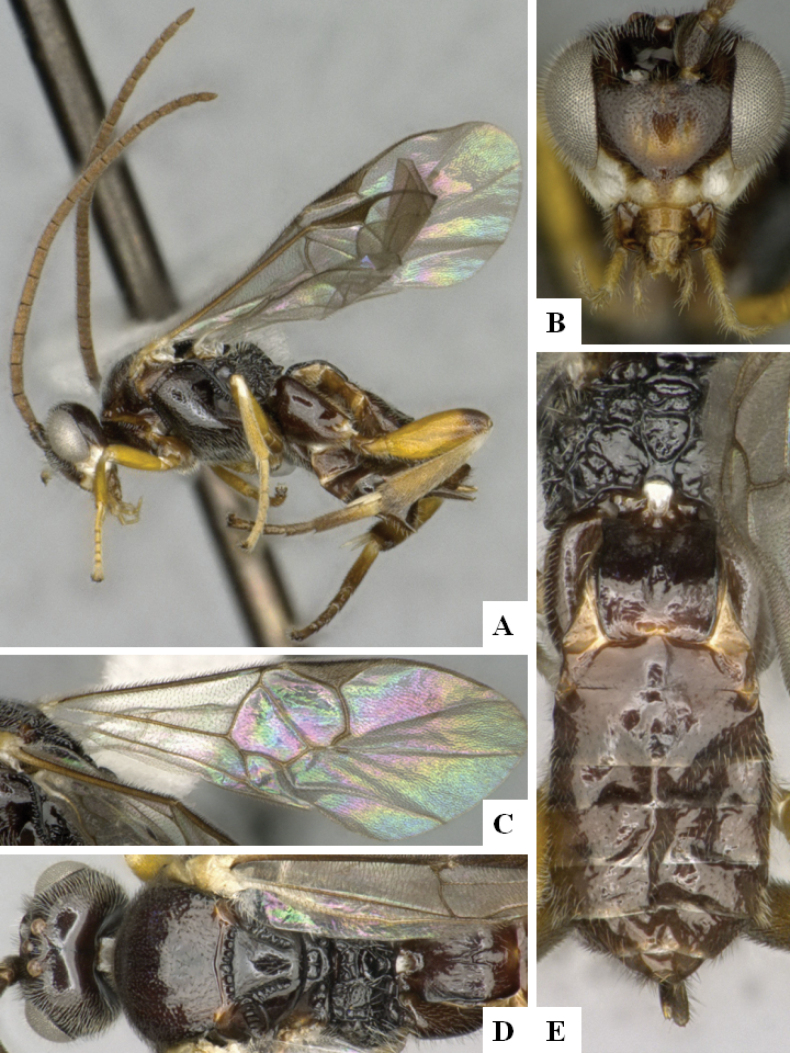
*Alphomelonbromeliphile* Deans female CNC704660 **A** habitus, lateral **B** head, frontal **C** fore wing **D** head and mesosoma, dorsal **E** propodeum and metasoma, dorsal.

**Figure 13.**
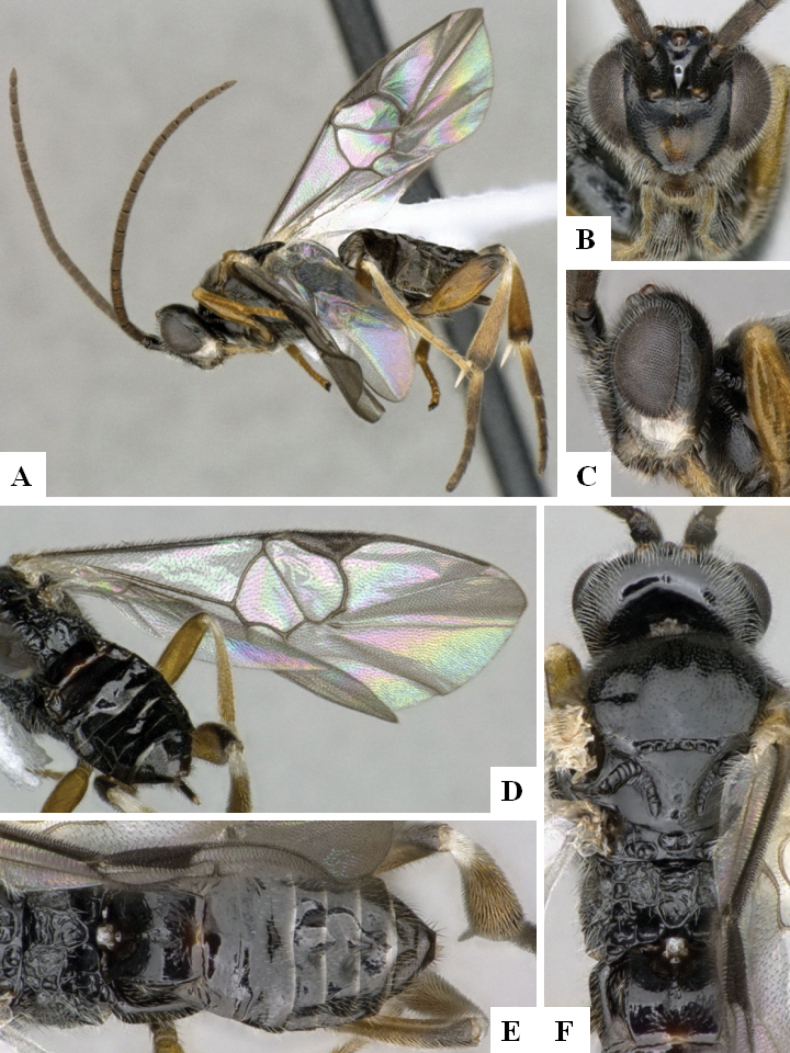
*Alphomelonbromeliphile* Deans female CNC1802037 **A** habitus, lateral **B** head, frontal **C** head, lateral **D** wings **E** Propodeum and metasoma, dorsal **F** mesosoma, dorsal.

**Figure 14.**
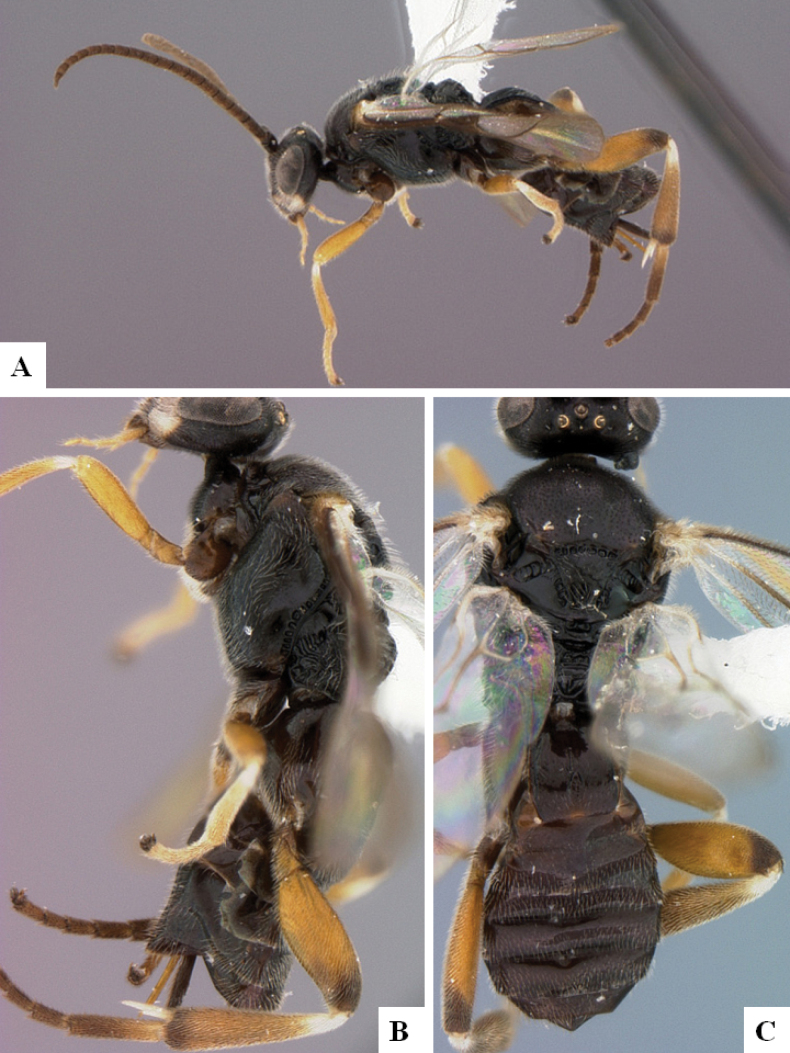
*Alphomelonbromeliphile* Deans holotype female USNMENT00828296 **A** habitus, lateral **B** close-up of habitus, lateral **C** habitus, dorsal.

Figs 12A–E , 13A–F , 14A–C , 92B

##### Distribution.

Costa Rica (ACG), Mexico.

##### Biology.

Gregarious, reared from *Neoxeniadesluda*.

##### DNA barcoding.

BINBOLD:AAB5598.

##### Other specimens examined.

(14 females, 7 males, 2 sex unknown). DHJPAR0051200, CNC1802037, CNC1802038, CNC1802039, CNC1802040, CNC1802041, CNC1802042, CNC1802043, DHJPAR0031621, DHJPAR0031657, DHJPAR0031669, DHJPAR0003979 (additional specimens in a gel capsule associated with that specimen), CNC1802044, CNC1802045, CNC1802046, CNCHYM 00011, CNC704663, CNC704660, CNC704662, CNC704661, CNC704659, CNC704658, CNC280514.

#### ﻿ Alphomelon calixtomoragai

Taxon classificationAnimaliaHymenopteraBraconidae

Fernandez-Triana & Shimbori sp. nov.

DE314F46-CF8E-5E61-A536-C7C09BE50292

https://zoobank.org/2083B0DD-A58D-4741-843D-C409ECEBE2CC

Figs 15A–F , 93A

##### Type material.

***Holotype*.** Costa Rica • Female, CNC; Guanacaste, Area de Conservación Guanacaste, Sector El Hacha, Vuelta Peligrosa, 11°02'07.22"N, 85°32'11.22"W, 280m; 25.IX.2008; ex. *Vettiusaurelius*; coll. Roster Moraga; Voucher code: DHJPAR0034186; Host voucher code: 08-SRNP-23629.

##### Other specimens examined.

Mexico • 1 female, CBG. Voucher code: BIOUG26457-C04.

##### Distribution.

Costa Rica (ACG), Mexico.

##### Biology.

Solitary, reared from *Vettiusaurelius*.

##### DNA barcoding.

BINBOLD:ADA5721.

##### Etymology.

Named in honor of Sr. Calixto Moraga in honor of his decades of teamwork in the ACG parataxonomist team.

##### Diagnostic description.

White patch on gena: extending to occiput and onto clypeus. Tegula/humeral complex color: yellow/yellow. Mesonotum color: mostly dark brown to black. Metasoma color: mostly dark brown to black but with some laterotergites and sternites yellow. Tarsal claws spines: 2. Pterostigma shape: comparatively less elongate, its length ≤ 2.5× its central height and usually more rounded with at least one of its lower margins curved. T1 sculpture: entirely to mostly smooth. T1 central ridge: clearly marked by two raised carinae. T2 sculpture: entirely to mostly smooth. Ovipositor sheaths length: longer than first segment of metatarsus. Body length: 4.3 mm. Fore wing length: 4.6 mm.

**Figure 15.**
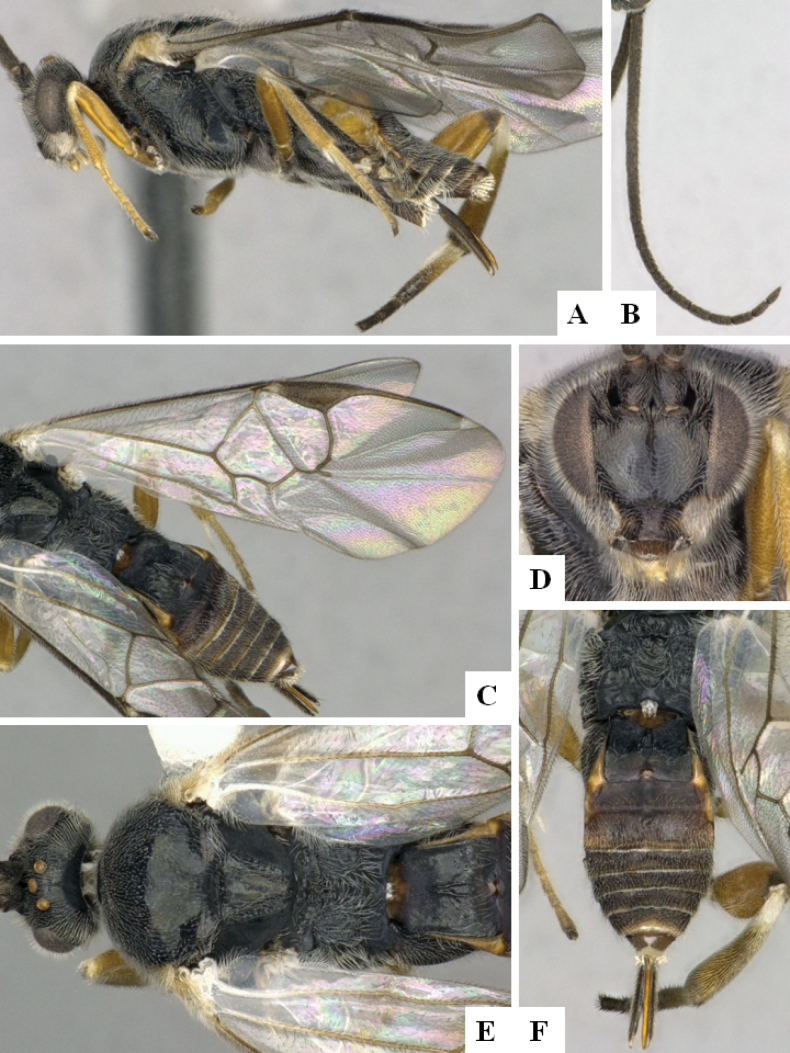
*Alphomeloncalixtomoragai* Fernandez-Triana & Shimbori holotype female DHJPAR0034186 **A** habitus, lateral **B** antenna, lateral **C** fore wing **D** head, frontal **E** head and mesosoma, dorsal **F** propodeum and metasoma, dorsal.

##### Notes.

The specimen from Mexico (which has an almost complete barcode with 564 bp) is associated with the species because its sequence matches perfectly with the specimen from ACG and the image available in BOLD is also similar. The sequences of *A.calixtomoragai* and *A.petronariosae* are comparatively very similar (only 1.28% bp difference) but they have morphological differences (see key) as well as different hosts.

#### ﻿ Alphomelon carolinacanoae

Taxon classificationAnimaliaHymenopteraBraconidae

Fernandez-Triana & Shimbori sp. nov.

9679B72C-EB06-5BCF-A7A0-12995F20ECE3

https://zoobank.org/E8F5245F-6310-4D89-BE95-8AEDE51B45CF

Figs 16A–E , 93B

##### Type material.

***Holotype*.** Costa Rica • Female, CNC; Guanacaste, Area de Conservación Guanacaste, Sector Cacao, Sendero Nayo, 10°55'28.06"N, 85°28'10.31"W, 1090m; 22.X.2013; ex. *Carystoidesescalantei*; coll. Harry Ramirez; Voucher code: DHJPAR0054776; Host voucher code: 13-SRNP-36219.

##### Distribution.

Costa Rica (ACG).

##### Biology.

Solitary, reared from *Carystoidesescalantei*.

##### DNA barcoding.

BINBOLD:ACE5969.

##### Etymology.

Named in honor of Sra. Carolina Cano in honor of her decades of teamwork in the ACG parataxonomist team.

##### Diagnostic description.

White patch on gena: extending to occiput but not to clypeus. Tegula/humeral complex color: white/yellow. Mesonotum color: mostly dark brown to black. Metasoma color: mostly black or dark brown. Tarsal claws spines: 1. Pterostigma shape: comparatively more elongate, its length ≥ 3.0× its central height and more triangular with its two lower margins more or less straight. T1 sculpture: strongly sculptured on at least apical half or more. T1 central ridge: strongly marked by two raised carinae and strong costulae centrally. T2 sculpture: entirely to mostly strongly sculptured. Ovipositor sheaths length: longer than first segment of metatarsus. Body length: 4.3 mm. Fore wing length: 4.5 mm.

**Figure 16.**
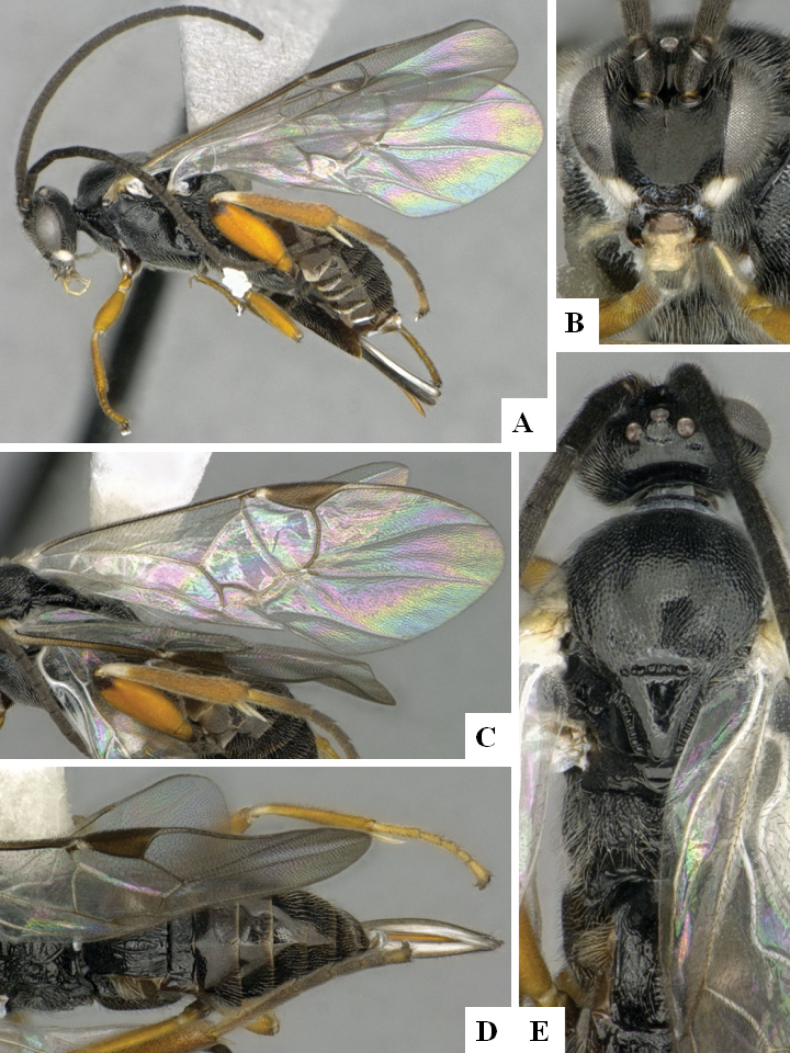
*Alphomeloncarolinacanoae* Fernandez-Triana & Shimbori holotype female DHJPAR0054776 **A** habitus, lateral **B** head, frontal **C** fore wing **D** metasoma, dorsal **E** head and mesosoma, dorsal.

##### Notes.

The single specimen studied had all the anterior and middle legs with one spine on every tarsal claw; one hind leg was missing, but the one present had a different number of spines on its tarsal claws (one claw had two spines whereas the second claw had one spine). Here we consider the species as having tarsal claws with one spine and thus, the species should be run through the first half of couplet 22; if other specimens become available in the future this species may need to be run differently in the key.

#### ﻿ Alphomelon christerhanssoni

Taxon classificationAnimaliaHymenopteraBraconidae

Fernandez-Triana & Shimbori sp. nov.

A9E44B3C-5F76-5C02-848D-343A8C249C24

https://zoobank.org/FD3052CE-87E2-4C87-8AC7-F989B25F0C5E

Figs 17A–G , 94A

##### Type material.

***Holotype*.** Costa Rica • Female, CNC; Alajuela, Area de Conservación Guanacaste, Sector Rincon Rain Forest, Quebrada Escondida, 10°53'57.41"N, 85°16'29.50"W, 420m; 21.III.2013; ex. *Carystinaaurifer*; coll. Pablo Umana Calderon; Voucher code: CNC308752; Host voucher code: 13-SRNP-41090.

***Paratypes*.** Costa Rica • 63 females, 6 males, CNC; DHJPAR0051210, DHJPAR0051778, DHJPAR0038130, DHJPAR0034208, CNC308762, CNC308761, CNC308763, CNC308764, CNC308765, CNC308766, CNC308767, CNC308768, CNC308769, CNC308770, CNC308771, CNC308772, CNC308773, CNC308774, CNC308775, CNC308776, CNC308777, CNC308778, CNC308779, CNC308780, CNC308781 (additional specimens in a gel capsule associated with that specimen), CNC308782, DHJPAR0052972, CNC308753, CNC308754, CNC308755, CNC308756, CNC308757 (additional specimens in a gel capsule associated with that specimen), DHJPAR0041775, DHJPAR0038131, CNC308786, CNC308783 (additional specimens in a gel capsule associated with that specimen), DHJPAR0038970, DHJPAR0030718, DHJPAR0041792, DHJPAR0047129, CNC308784, CNC308785 (additional specimens in a gel capsule associated with that specimen), CNC958829, CNC958831, CNC958830, CNC958832 (additional specimens in a gel capsule associated with that specimen), DHJPAR0039878, DHJPAR0055257, CNC308787, CNC308788, CNC308789, CNC308790, CNC308791, CNC308792, CNC308793, CNC308794, CNC308795, DHJPAR0053105, CNC308800, CNC308801, DHJPAR0020200, CNC308796, CNC308797, DHJPAR0053730, CNC308798, CNC308799, DHJPAR0054626, CNC308802, CNC308803.

**Figure 17.**
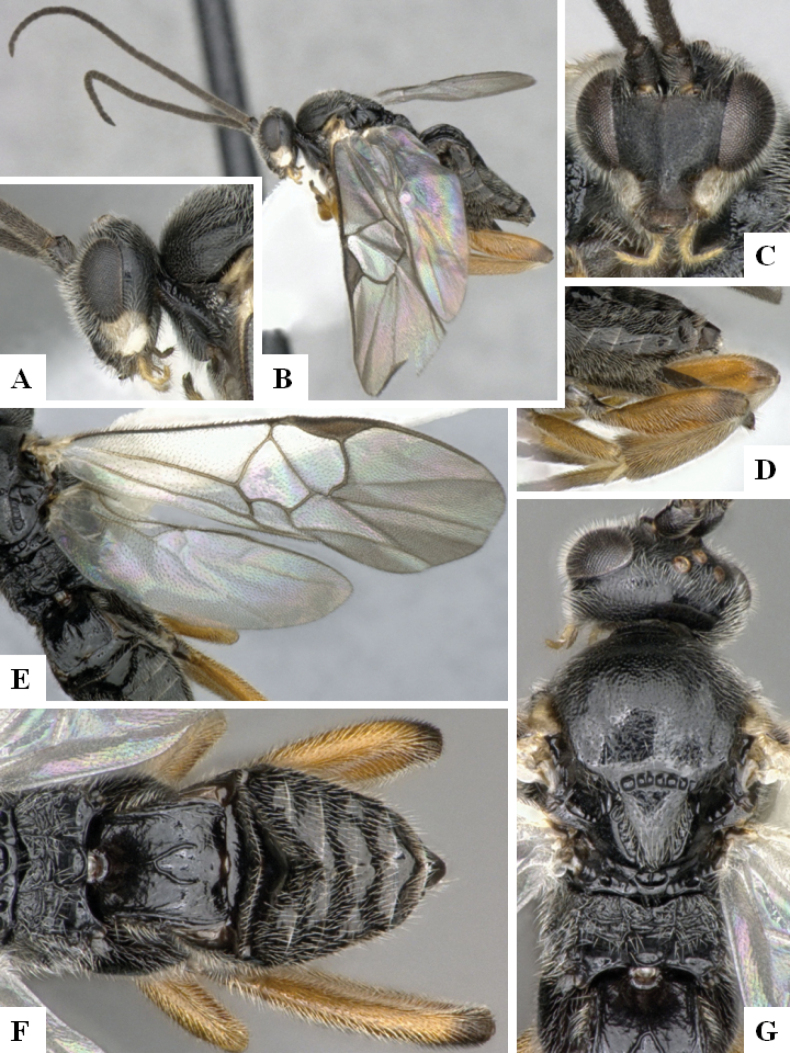
*Alphomelonchristerhanssoni* Fernandez-Triana & Shimbori holotype female CNC308752 **A** head, lateral **B** habitus, lateral **C** head, frontal **D** ovipositor, lateral **E** wings, **F** propodeum and metasoma, dorsal **G** mesosoma, dorsal.

##### Distribution.

Costa Rica (ACG).

##### Biology.

Gregarious, reared from *Aidesbrino*, *Carystinaaurifer*, and *Dubiellabelpa*.

##### DNA barcoding.

BINBOLD:AAB0787.

##### Etymology.

Named after Mr. Christer Hansson in honor of his dedication to the taxonomy of the Eulophidae (Chalcidoidea) of Costa Rica.

##### Diagnostic description.

White patch on gena: extending to occiput and onto clypeus. Tegula/humeral complex color: yellow/ partially yellow and partially brown. Mesonotum color: mostly dark brown to black. Metasoma color: mostly black or dark brown. Tarsal claws spines: 1. Pterostigma shape: comparatively more elongate, its length ≥ 3.0× its central height and more triangular with its two lower margins more or less straight. T1 sculpture: entirely to mostly smooth. T1 central ridge: clearly marked by two raised carinae. T2 sculpture: entirely to mostly smooth. Ovipositor sheaths length: longer than first segment of metatarsus. Body length: 3.6–3.9 mm. Fore wing length: 3.4–3.8 mm.

#### ﻿ Alphomelon citroloma

Taxon classificationAnimaliaHymenopteraBraconidae

Deans, 2003

16B7ECD1-DD5E-545A-AE56-AA72BAFCF96D

**Figure 18.**
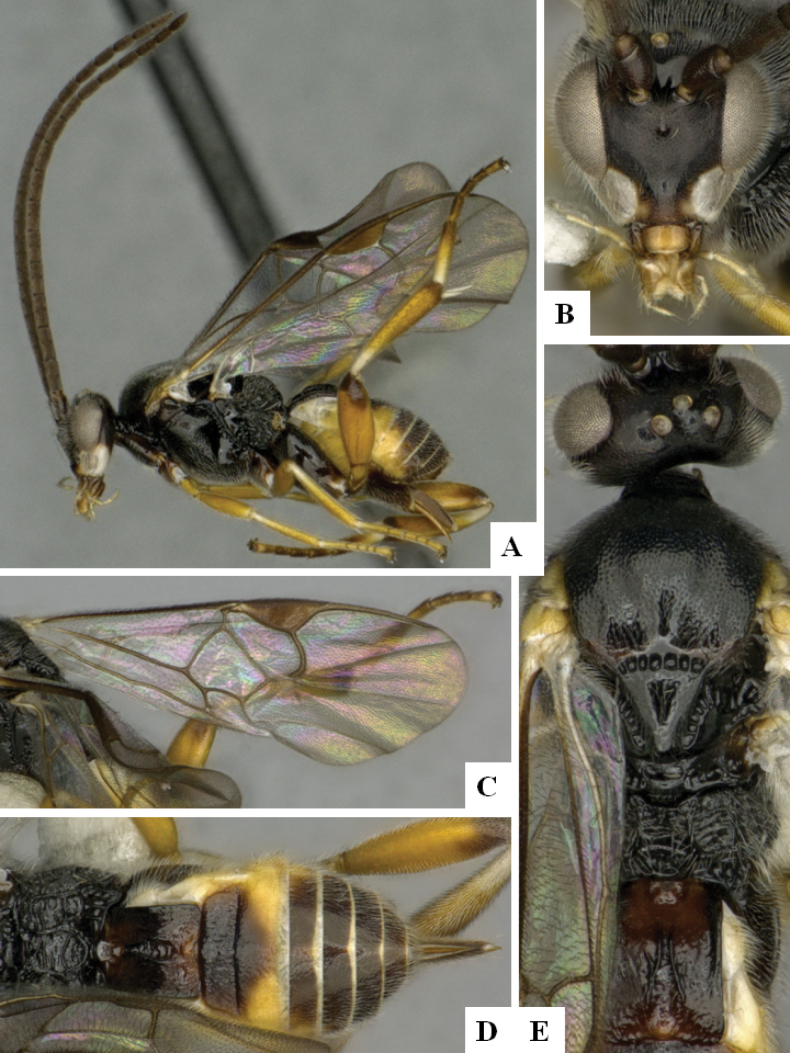
*Alphomeloncitroloma* Deans female CNC704170 **A** habitus, lateral **B** head, frontal **C** fore wing **D** propodeum and metasoma, dorsal **E** mesosoma, dorsal.

**Figure 19.**
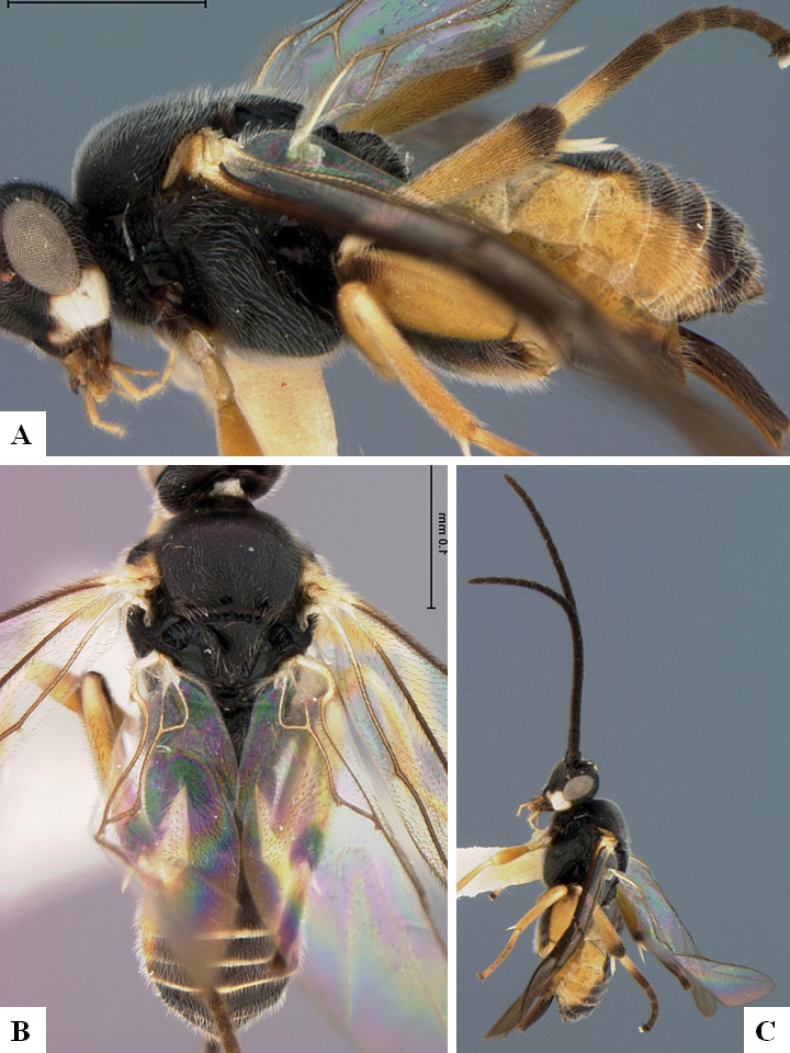
*Alphomeloncitroloma* Deans holotype female USNMENT00828298 **A** close-up of habitus, lateral **B** habitus, dorsal **C** habitus, lateral.

**Figure 20.**
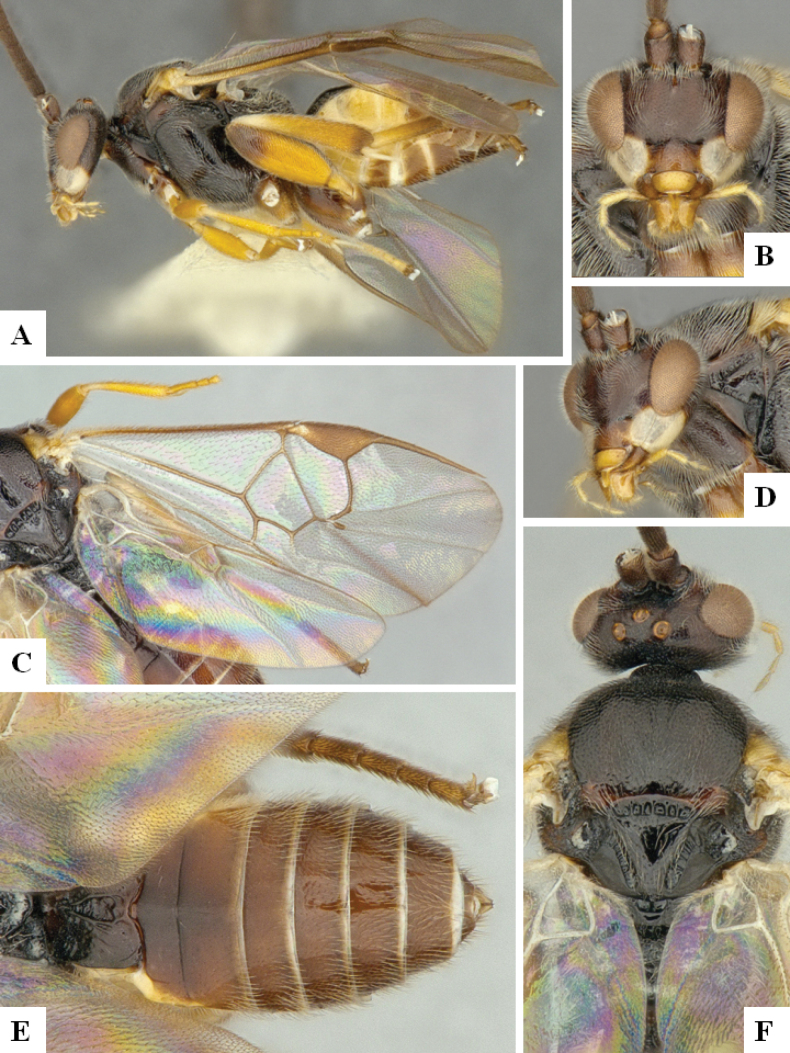
*Alphomeloncitroloma* Deans male CNCHYM 00019 **A** habitus, lateral **B** head, frontal **C** wings **D** head, fronto-lateral **E** metasoma, dorsal **F** mesosoma, dorsal.

Figs 18A–E , 19A–C , 20A–F

##### Distribution.

Argentina, Belize*, Bolivia, Brazil (PE, RJ, RO), Costa Rica, Ecuador, Panama, Paraguay, Peru*, Trinidad & Tobago, Venezuela.

##### Biology.

No data.

##### DNA barcoding.

No BIN but four partial barcodes (109–234 bp) available.

##### Notes.

In the CNC collection many specimens from Central and South America look morphologically similar to *A.citroloma*. However, they differ in coloration, sculpture of T2, and the number of spines on the tarsal claws. We suspect additional cryptic species may be discovered in the future when the South American fauna is better studied and more DNA barcodes become available.

##### Other specimens examined.

(79 females, 72 males, 1 sex unknown): CNC280515, CNCHYM 00019, CNCHYM 00016, CNCHYM 00018, CNCHYM 00012, CNCHYM 00013, CNCHYM 00014, CNCHYM 00015, CNCH0686, CNC704128, CNC704129, CNC704130, CNC704131, CNC704132, CNC704133, CNC704134, CNC704135, CNC704136, CNC704137, CNC704138, CNC704139, CNC704140, CNC704141, CNC704142, CNC704143, CNC704144, CNC704145, CNC704146, CNC704147, CNC704148, CNC704149, CNC704150, CNC704151, CNC704152, CNC704153, CNC704154, CNC704155, CNC704156, CNC704157, CNC704158, CNC704159, CNC704160, CNC704161, CNC704162, CNC704163, CNC704164, CNC704165, CNC704166, CNC704167, CNC704168, CNC704169, CNC704170, CNC704171, CNC704172, CNC704173, CNC704174, CNC704175, CNC704176, CNC704177, CNC704178, CNC704179, CNC704180, CNC704181, CNC704182, CNC704183, CNC704184, CNC704185, CNC704186, CNC704187, CNC704188, CNC704189, CNC704190, CNC704191, CNC704192, CNC704193, CNC704194, CNC704195, CNC704196, CNC704197, CNC704198, CNC704199, CNC704200, CNC704201, CNC704202, CNC704203, CNC704204, CNC704205, CNC704206, CNC704207, CNC704208, CNC704209, CNC704210, CNC704211, CNC704212, CNC704213, CNC704214, CNC704215, CNC704216, CNC704217, CNC704218, CNC704219, CNC704220, CNC704221, CNC704222, CNC704223, CNC704224, CNC704225, CNC704226, CNC704227, CNC704228, CNC704229, CNC704230, CNC704231, CNC704232, CNC704233, CNC704234, CNC704235, CNC704236, CNC704237, CNC704238, CNC704239, CNC704240, CNC704241, CNC704242, CNC704243, CNC704244, CNC704245, CNC704246, CNC704247, CNC704248, CNC704249, CNC704250, CNC704251, CNC704252, CNC704253, CNC704670, CNC704671, CNC704672, CNC704673, CNC704674, CNC704675, CNC704676, CNC704677, CNC704678, CNC704679, CNC704680, CNC704681, CNC704682, CNC704685, CNC704686, CNC704687, CNC1065904.

#### ﻿ Alphomelon conforme

Taxon classificationAnimaliaHymenopteraBraconidae

(Muesebeck, 1958)

DD36FCA1-82C2-58AF-A3ED-0AB3722791B9

Fig. 21A–H

##### Distribution.

Brazil (RJ), Costa Rica, Venezuela; specimens collected in rainforest sites.

##### Biology.

Gregarious, reared from unidentified hesperiine feeding on Cannaceae in Venezuela and unidentified hesperiine feeding on Poaceae in Costa Rica.

**Figure 21.**
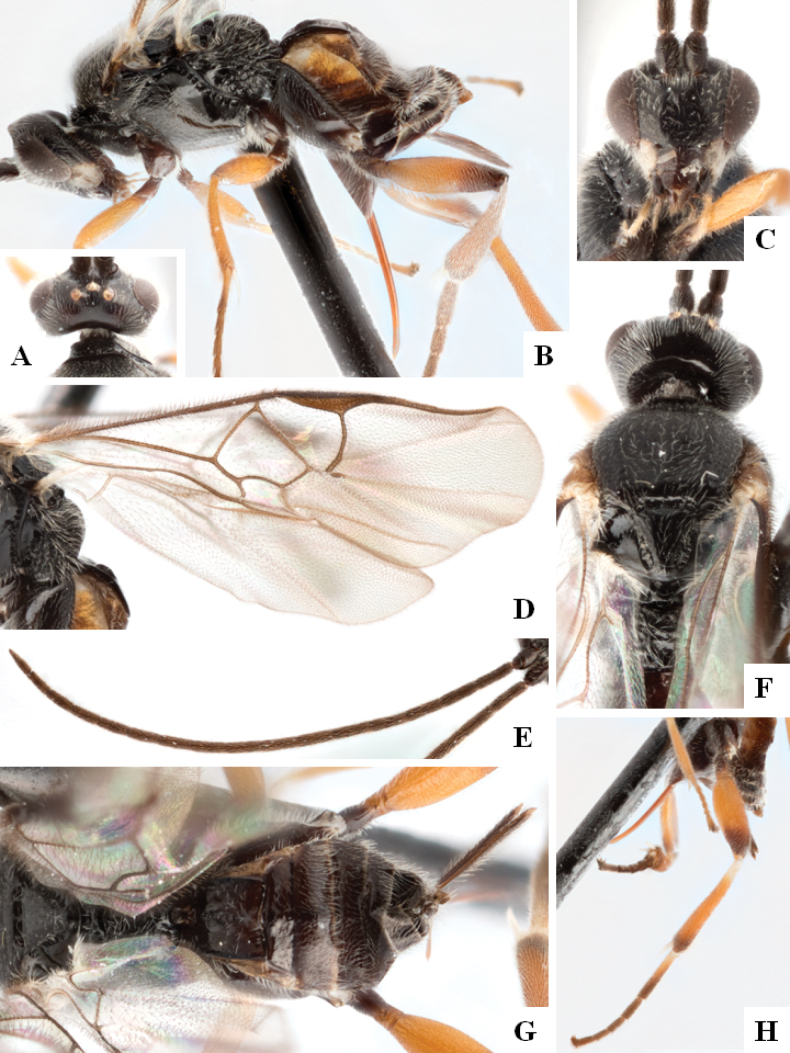
*Alphomelonconforme* (Muesebeck) female from AEIC**A** head, dorsal **B** habitus, lateral **C** head, frontal **D** wings **E** antenna, ventral **F** mesosoma, dorsal **G** metasoma, dorsal **H** hind leg.

##### DNA barcoding.

Not available.

#### ﻿ Alphomelon crocostethus

Taxon classificationAnimaliaHymenopteraBraconidae

Deans, 2003

3B5F1F0D-3B2B-517C-B68D-18B3D0A4FE94

**Figure 22.**
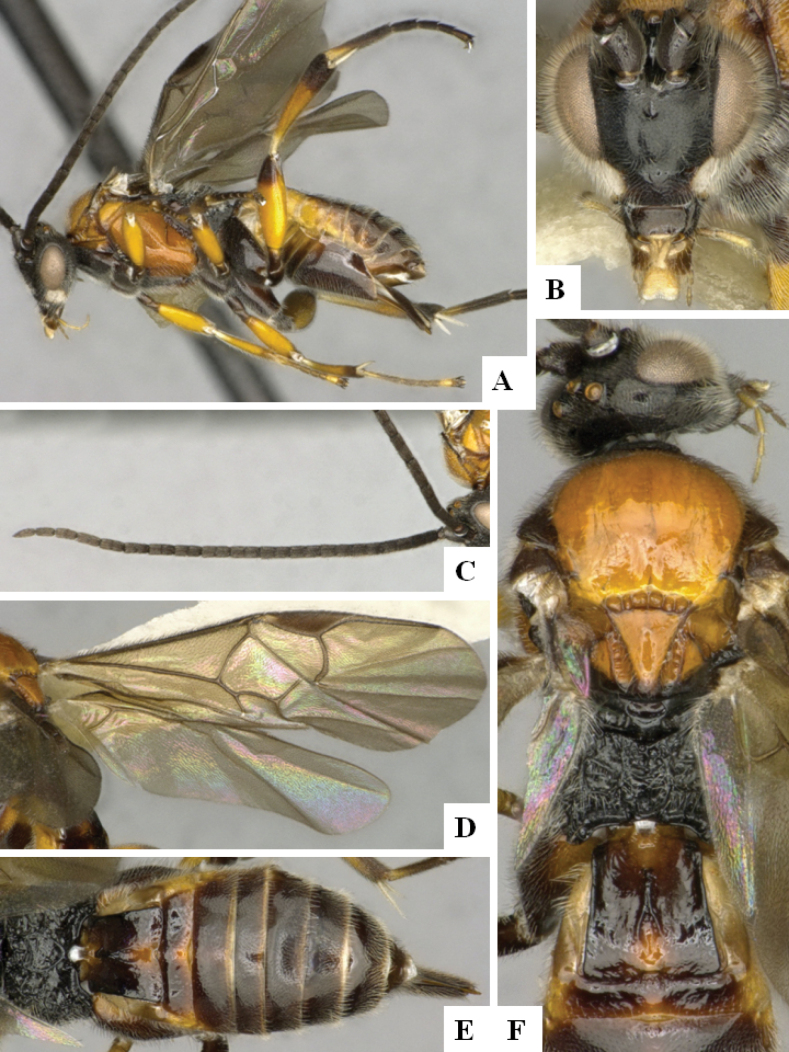
*Alphomeloncrocostethus* Deans female CNC704257 **A** habitus, lateral **B** head, frontal **C** antenna, lateral **D** wings **E** propodeum and metasoma, dorsal **F** mesosoma, dorsal.

**Figure 23.**
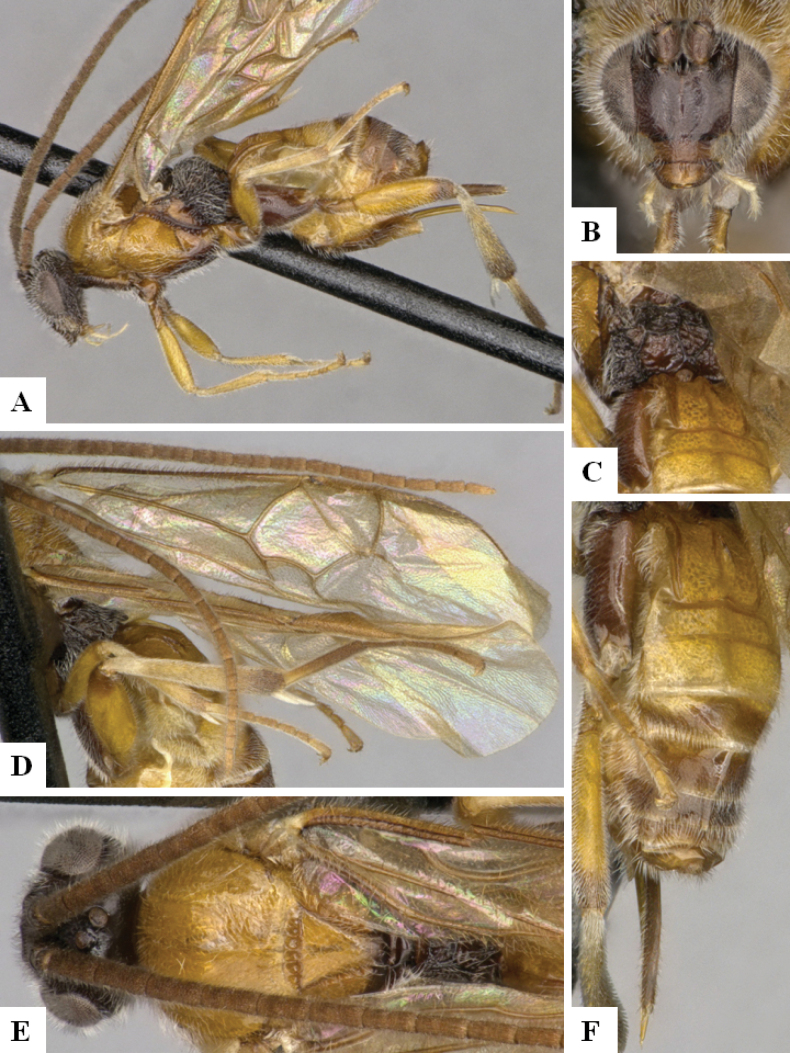
*Alphomeloncrocostethus* Deans female CNC1065929 **A** habitus, lateral **B** head, frontal **C** propodeum, dorso-lateral **D** wings **E** head and mesosoma, dorsal **F** metasoma, dorso-lateral.

**Figure 24.**
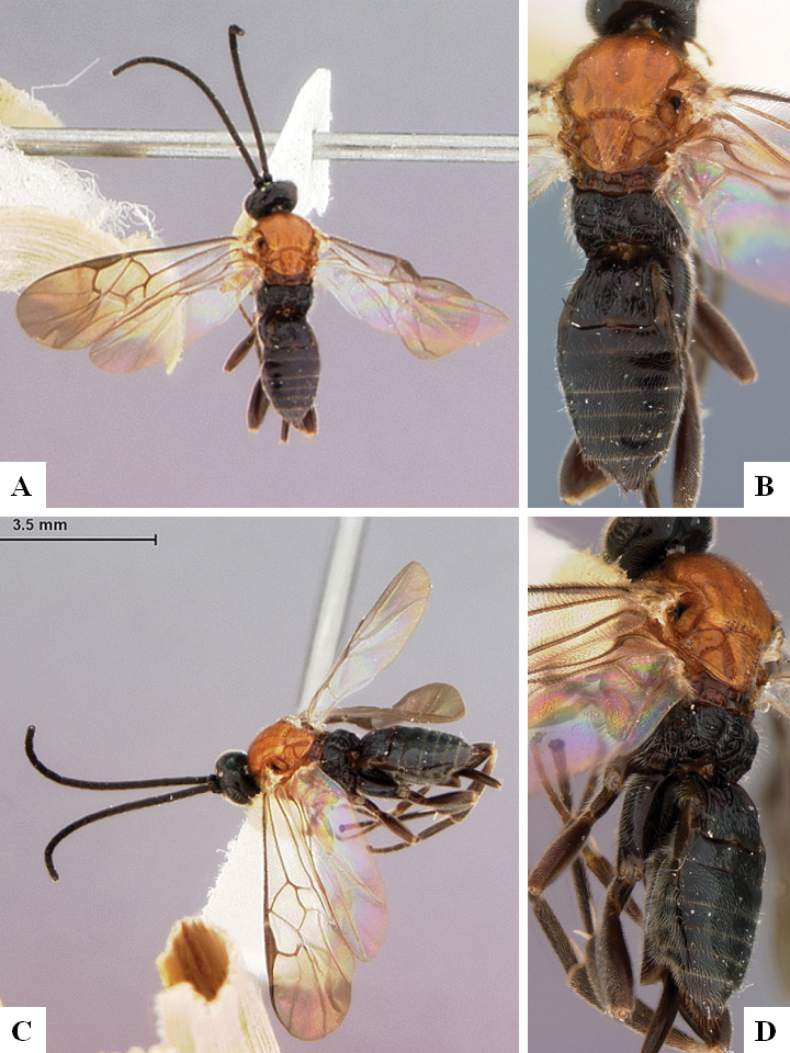
*Alphomeloncrocostethus* Deans holotype female USNMENT00828299 **A** habitus, dorsal **B** close-up of habitus, dorsal **C** habitus, lateral **D** close-up of habitus, lateral.

Figs 22A–F , 23A–F , 24A–D

##### Distribution.

Argentina*, Bolivia, Brazil (DF*, ES, MG, PE*, RJ, RN*, SP*), Colombia, Dominican Republic*, Jamaica, Peru*, Puerto Rico.

##### Biology.

Solitary, reared from unidentified hesperiid on sugar cane *Saccharumofficinarum* (Deans et al. 2003).

##### DNA barcoding.

BINBOLD:AAZ9859.

##### Notes.

The specimens we examined are very variable and it is likely that there are several species within what is currently considered *A.crocostethus*. The Caribbean specimens have darker legs, mesosoma and metasoma; T1 with stronger sculpture (near posterior margins); and T1 petiolar ridge tends to be larger and wider. The South American specimens vary considerably, but usually have paler colored legs, mesosoma, and metasoma; T1 with weaker sculpture; and T1 petiolar ridge shorter, narrower, and usually less well defined. However, there are exceptions, sometimes from the same locality (such as in Brazil, where most of the material we have examined was collected). Besides morphology, DNA barcoding supports more than one species, although only four specimens (three with partial barcodes) are available. Until more specimens and sequences from South America are available for study, we think is best to keep a single species, but future work will certainly reveal more within what appears to be a species complex.

##### Other specimens examined.

(13 females): CNC280516, CNCHYM 00021, CNCHYM 00022, CNCHYM 00023, CNCHYM 00020, CNC704254, CNC704255, CNC704256, CNC704257, CNC704258, CNC1065929, CNC1179774, CNC1179953.

#### ﻿ Alphomelon cruzi

Taxon classificationAnimaliaHymenopteraBraconidae

Shimbori & Fernandez-Triana sp. nov.

CBB82AF1-78FC-5BE2-A1EE-3EB8ACF890FC

https://zoobank.org/784074FB-BF74-4A7D-8ABE-341B10A207C8

Fig. 25A–E

##### Type material.

***Holotype*.** Brazil • Female “Sete Lagoas, MG, Brasil / EMBRAPA Milho e Sorgo / Mata João Dias / S19o25'27,1” W44o8'59” / Armadilha Malaise / 11.IV – 21.IV.2011 / I. Cruz col.” (DCBU 82866)

##### Distribution.

Brazil.

##### Biology.

No data.

##### DNA barcoding.

None available.

##### Etymology.

Named after Sr. Ivan Cruz, collector of the type specimen.

##### Diagnostic description.

White patch on gena: extending to occiput but not to clypeus. Tegula/humeral complex color: yellow/yellow. Mesonotum color: mostly dark brown to black. Metasoma color: mostly black or dark brown. Tarsal claws spines: 2. Pterostigma shape: comparatively more elongate, its length ≥ 3.0× its central height and more triangular with its two lower margins more or less straight. T1 sculpture: entirely to mostly smooth. T1 central ridge: clearly marked by two raised carinae. T2 sculpture: entirely to mostly smooth. Ovipositor sheaths length: longer than first segment of metatarsus. Body length: 4.1 mm. Fore wing length: 4.4 mm.

**Figure 25.**
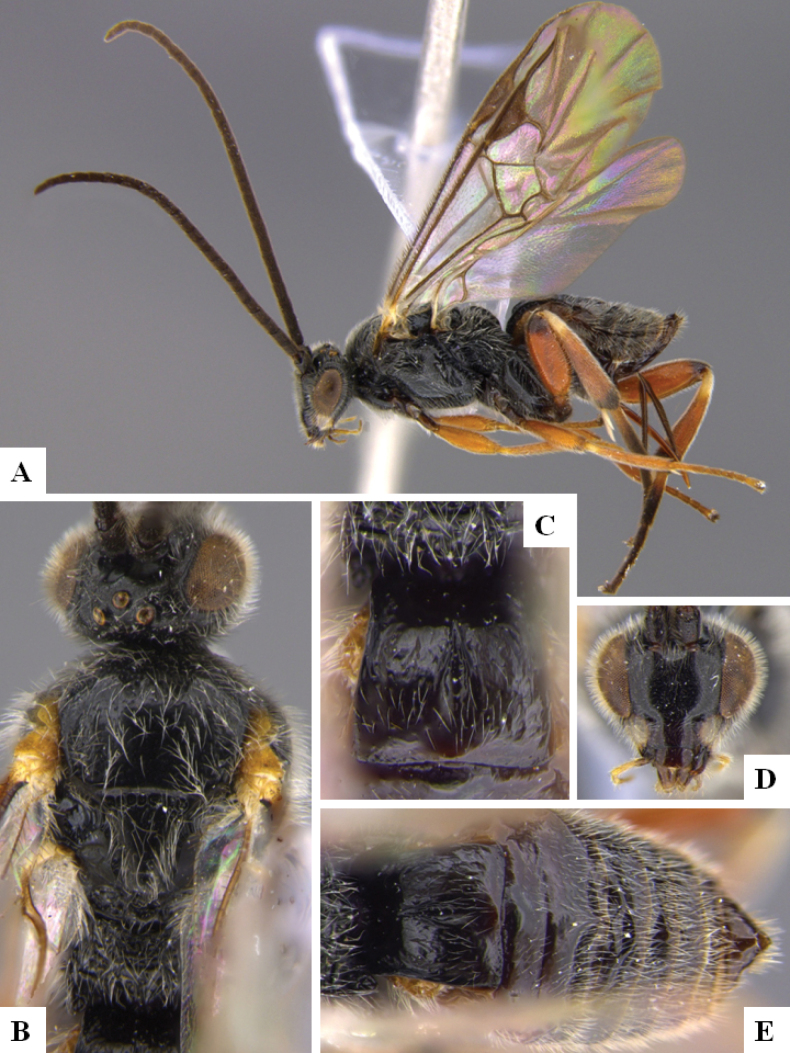
*Alphomeloncruzi* Shimbori & Fernandez-Triana holotype female DCBU 82866 **A** habitus, lateral **B** mesosoma, dorsal **C** T1, dorsal **D** head, frontal **E** metasoma, dorsal.

#### ﻿ Alphomelon diniamartinezae

Taxon classificationAnimaliaHymenopteraBraconidae

Fernandez-Triana & Shimbori sp. nov.

3469B30A-F6A8-510B-AD09-F83DD55C3CA6

https://zoobank.org/DEEBC1C0-880A-4541-B5F6-451D04AB1D3D

Figs 26A–F , 94B

##### Type material.

***Holotype*.** Costa Rica • Female, CNC; Alajuela, Area de Conservación Guanacaste, Sector Rincon Rain Forest, Laureles, 10°55'59.48"N, 85°15'12.06"W, 95m; 21.X.2006; ex. *Niconiadesincomptus*; coll. Anabelle Cordoba; Voucher code: DHJPAR0012804; Host voucher code: 06-SRNP-43998.

***Paratypes*.** Costa Rica • 2 females, 1 male, CNC; CNC1180084, CNC1180085, CNC1180086.

##### Distribution.

Costa Rica (ACG).

##### Biology.

Gregarious, reared from *Niconiadesincomptus*.

##### DNA barcoding.

BINBOLD:AAE2209.

##### Etymology.

Named in honor of Srta. Dinia Martínez in honor of her decades of teamwork in the ACG parataxonomist team.

##### Diagnostic description.

White patch on gena: extending to occiput and onto clypeus. Tegula/humeral complex color: yellow/yellow. Mesonotum color: mostly dark brown to black. Metasoma color: mostly black or dark brown. Tarsal claws spines: 3. Pterostigma shape: comparatively less elongate, its length ≤ 2.5× its central height and usually more rounded with at least one of its lower margins curved. T1 sculpture: entirely to mostly smooth. T1 central ridge: absent. T2 sculpture: entirely to mostly smooth. Ovipositor sheaths length: longer than first segment of metatarsus. Body length: 2.5–2.8mm. Fore wing length: 2.6–2.8 mm.

**Figure 26.**
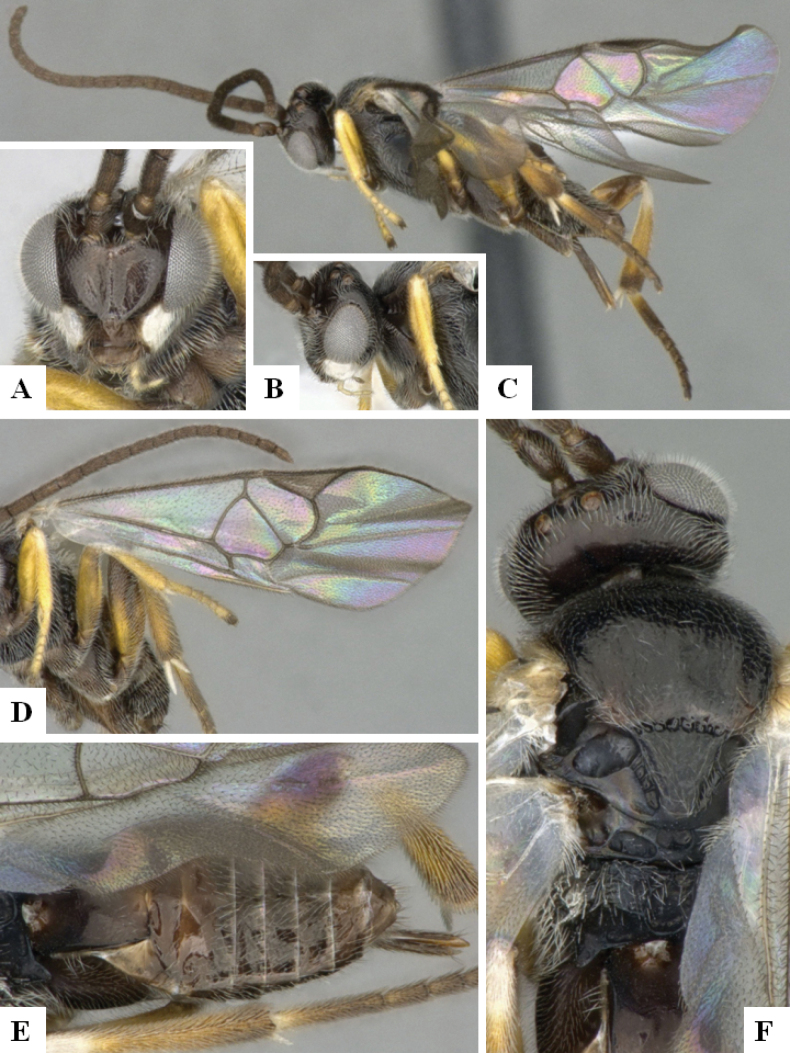
*Alphomelondiniamartinezae* Fernandez-Triana & Shimbori holotype female DHJPAR0012804 **A** head, frontal **B** head, lateral **C** habitus, lateral **D** fore wing **E** metasoma, dorsal **F** mesosoma, dorsal.

#### ﻿ Alphomelon disputabile

Taxon classificationAnimaliaHymenopteraBraconidae

(Ashmead, 1900)

C66E79A1-9E90-510A-B381-8206C08C29C7

**Figure 27.**
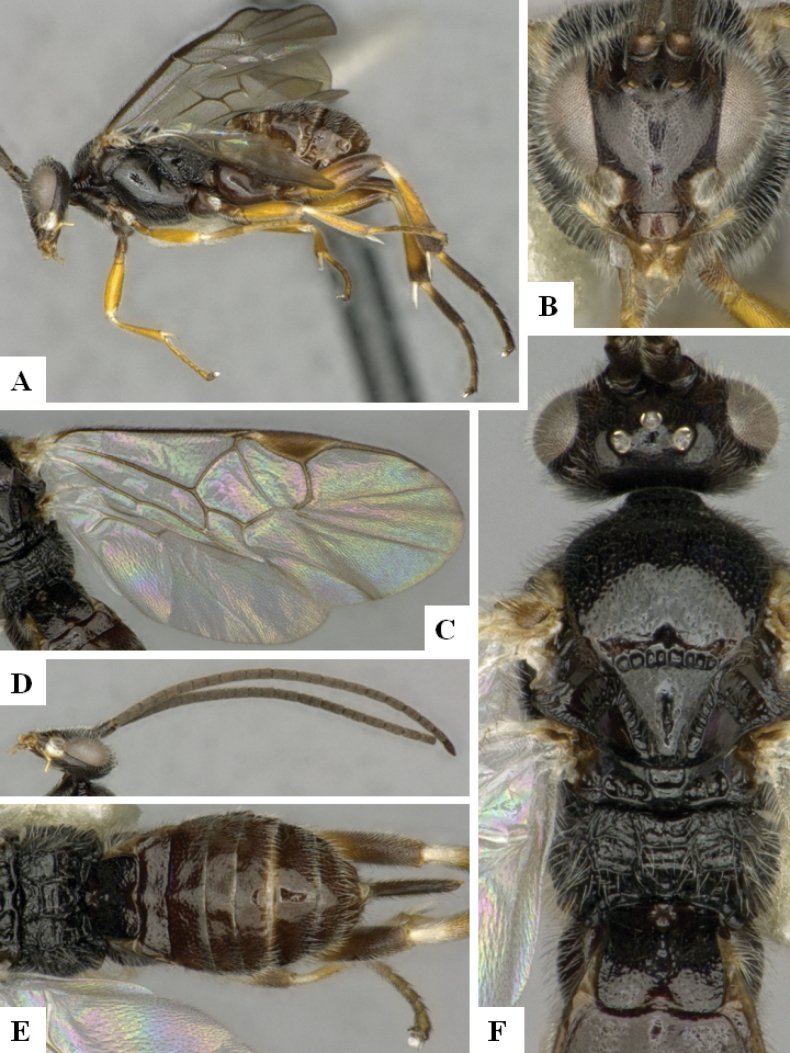
*Alphomelondisputabile* (Ashmead) female CNC704298 **A** habitus, lateral **B** head, frontal **C** wings **D** antennae, lateral **E** propodeum and metasoma, dorsal **F** head and mesosoma, dorsal.

**Figure 28.**
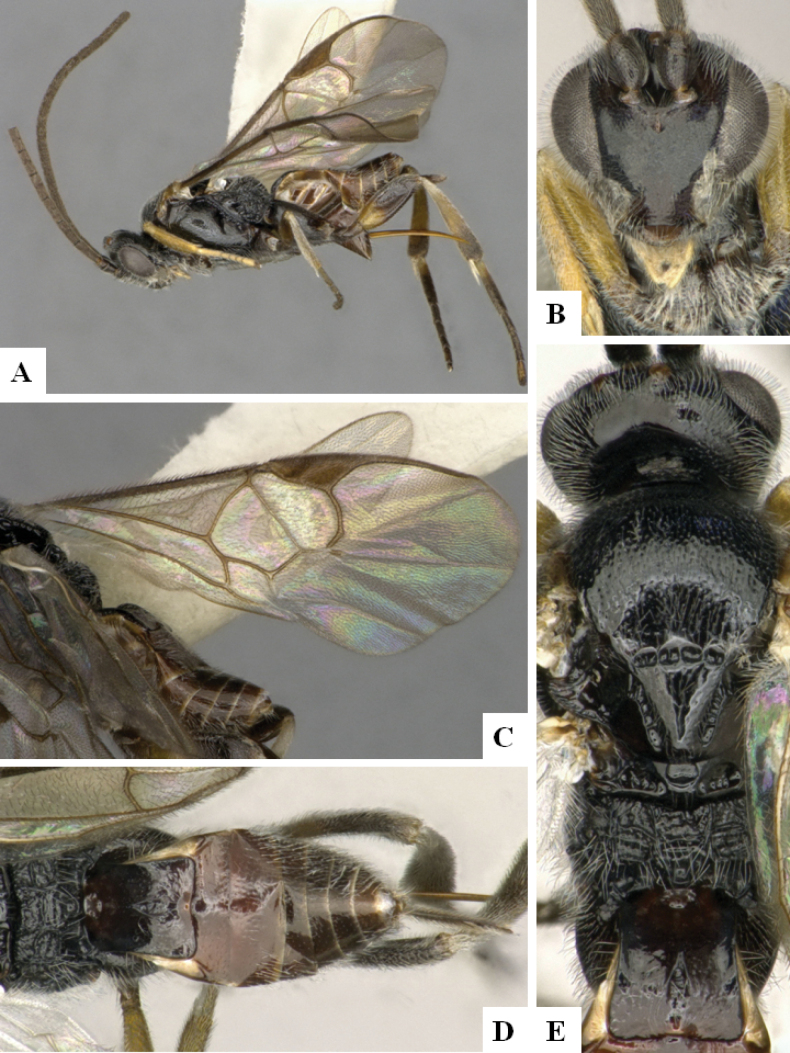
*Alphomelondisputabile* (Ashmead) female CNC1065931 **A** habitus, lateral **B** head, frontal **C** wings **D** propodeum and metasoma, dorsal **E** mesosoma, dorsal.

**Figure 29.**
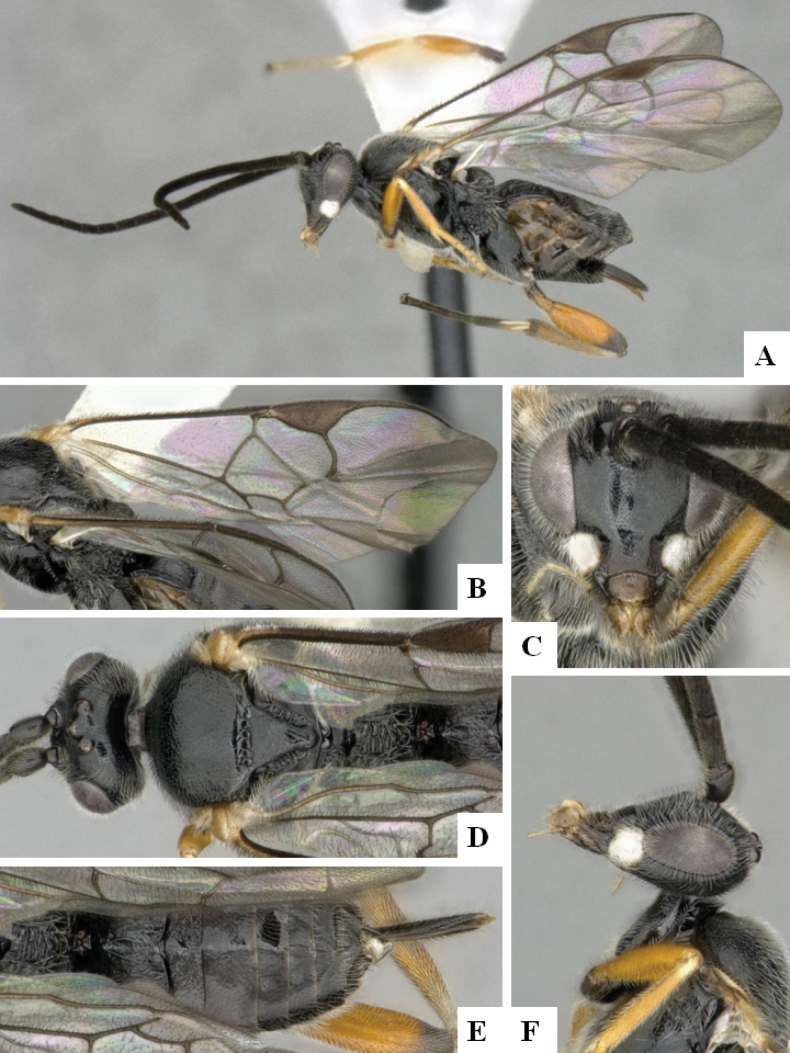
*Alphomelondisputabile* (Ashmead) female DHJPAR0031614 **A** habitus, lateral **B** wings **C** head, frontal **D** head and mesosoma, dorsal **E** propodeum and metasoma, dorsal **F** head, lateral.

Figs 27A–F , 28A–E , 29A–F

##### Distribution.

Argentina, Belize, Bolivia, Brazil (ES, MT, PA, RJ, SC), Costa Rica, Cuba, Dominica, Ecuador, Grenada, Guatemala, Mexico, Panama, Paraguay, Peru*, Puerto Rico, Saint Vincent, Trinidad & Tobago, United States (KS, TX), Venezuela.

##### Biology.

Solitary, reared from *Cymaenestrebius* and *Lerema* spp. on Poaceae.

##### DNA barcoding.

BINBOLD:AAF3301.

##### Notes.

Some specimens may have tarsal claws with two spines.

##### Other specimen examined.

(70 females, 43 males, 1 sex unknown): DHJPAR0031614, CNCHYM 00005, CNCHYM 00033, CNCHYM 00031, CNCHYM 00030, CNCHYM 00024, CNCHYM 00026, CNCHYM 00027, CNCHYM 00028, CNCHYM 00029, CNCHYM 00032, CNC704259, CNC704260, CNC704261, CNC704262, CNC704263, CNC704264, CNC704265, CNC704266, CNC704267, CNC704268, CNC704269, CNC704270, CNC704271, CNC704272, CNC704273, CNC704274, CNC704275, CNC704276, CNC704277, CNC704278, CNC704279, CNC704280, CNC704281, CNC704282, CNC704283, CNC704284, CNC704285, CNC704286, CNC704290, CNC704291, CNC704292, CNC704293, CNC704294, CNC704295, CNC704296, CNC704297, CNC704298, CNC704299, CNC704300, CNC704301, CNC704302, CNC704303, CNC704304, CNC704305, CNC704306, CNC704307, CNC704308, CNC704309, CNC704310, CNC704311, CNC704312, CNC704313, CNC704314, CNC704315, CNC704316, CNC704317, CNC704318, CNC704319, CNC704320, CNC704321, CNC704322, CNC704323, CNC704324, CNC704325, CNC704326, CNC704327, CNC704328, CNC704329, CNC704330, CNC704331, CNC704332, CNC704333, CNC704334, CNC704335, CNC704336, CNC704337, CNC704338, CNC704339, CNC704340, CNC704341, CNC704342, CNC704343, CNC704344, CNC704345, CNC704346, CNC704347, CNC704348, CNC704349, CNC704350, CNC704351, CNC704352, CNC704353, CNC704354, CNC704355, CNC704356, CNC704357, CNC704358, CNC704359, CNC704360, CNC734950, CNC1065892, CNC1065931, CNC1065995.

#### ﻿ Alphomelon duvalierbricenoi

Taxon classificationAnimaliaHymenopteraBraconidae

Fernandez-Triana & Shimbori sp. nov.

A42DF4A5-FAC2-52BA-B832-D20F842573FF

https://zoobank.org/394F9781-BF68-48BF-9BF2-4E5CA30167C9

Figs 30A–E , 95A

##### Type material.

***Holotype*.** Costa Rica • Female, CNC; Guanacaste, Area de Conservación Guanacaste, Sector Mundo Nuevo, Vado Miramonte, 10°46'18.30"N, 85°26'02.40"W, 305m; 27.III.2007; ex. *Methionopsisina*; coll. Jose Alberto Sanchez; Voucher code: DHJPAR0030806; Host voucher code: 07-SRNP-55789.

***Paratypes*.** Costa Rica • 4 females, 1 male, CNC; CNC1180109, CNC1180011 (additional specimens in a gel capsule associated with that specimen), CNC1180117, CNC1180124, DHJPAR00002291.

##### Distribution.

Costa Rica (ACG).

##### Biology.

Gregarious, reared from *Methionopsisina*.

##### DNA barcoding.

BINBOLD:AAB4029.

##### Etymology.

Named in honor of Sr. Duvalier Briceño in honor of his decades of teamwork in the ACG parataxonomist team.

##### Diagnostic description.

White patch on gena: extending to occiput and onto clypeus. Tegula/humeral complex color: white/yellow.

Mesonotum color: mostly dark brown to black. Metasoma color: mostly dark brown to black but with some laterotergites and sternites yellow. Tarsal claws spines: 2. Pterostigma shape: comparatively less elongate, its length ≤ 2.5× its central height and usually more rounded with at least one of its lower margins curved. T1 sculpture: entirely to mostly smooth. T1 central ridge: faintly indicated by shallow depression/marked by weak carina. T2 sculpture: entirely to mostly smooth. Ovipositor sheaths length: longer than first segment of metatarsus. Body length: 3.1–3.4 mm. Fore wing length: 3.1–3.5 mm.

**Figure 30.**
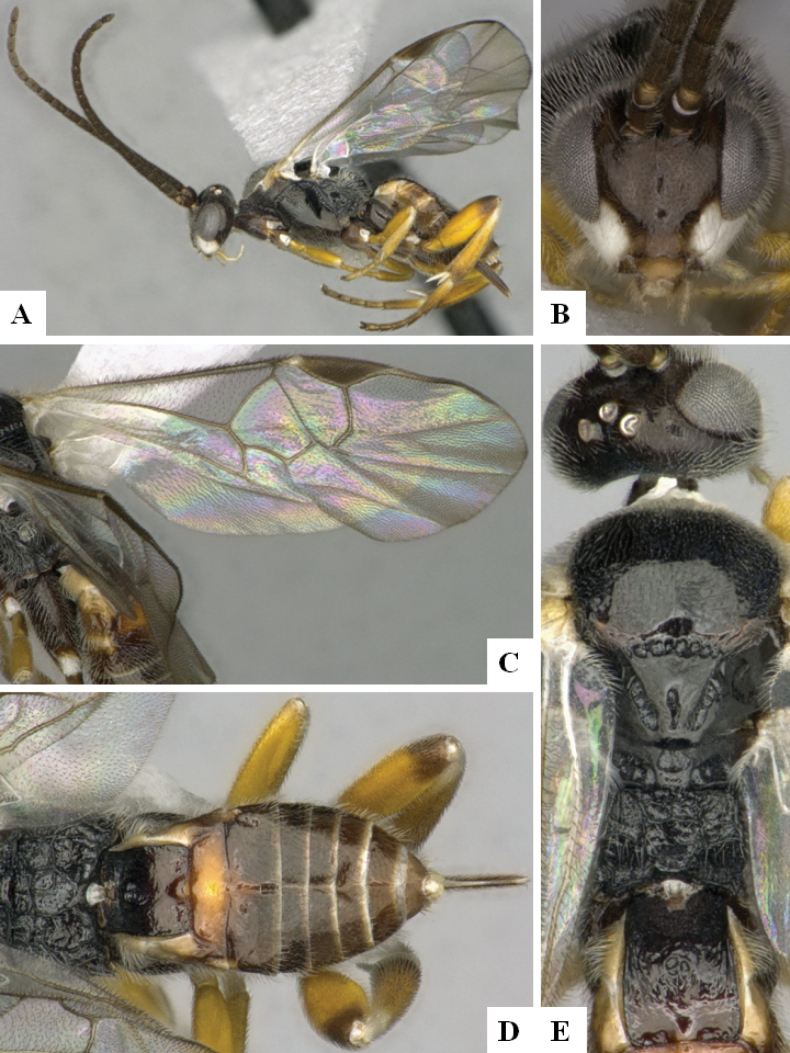
*Alphomelonduvalierbricenoi* Fernandez-Triana & Shimbori holotype female CNC1180104 **A** habitus, lateral **B** head, frontal **C** wings **D** propodeum and metasoma, dorsal **E** mesosoma, dorsal.

#### ﻿ Alphomelon eldaarayae

Taxon classificationAnimaliaHymenopteraBraconidae

Fernandez-Triana & Shimbori sp. nov.

498487E2-3F19-5778-88BD-D5573A8A4A1F

https://zoobank.org/A03807BB-1149-4331-9902-EF4378AD2383

Figs 31A–E , 95B

##### Type material.

***Holotype*.** Costa Rica • Female, CNC; Guanacaste, Area de Conservacion Guanacaste, Sector Pitilla, Sendero Naciente, 10°59'13.38"N, 85°25'41.38"W, 700m; 18.IV.2007; ex: *Neoxeniades* sp. Burns04; coll. Petrona Rios; Voucher code: CNC1179987; Host voucher code: 07-SRNP-31939.

***Paratypes*.** Costa Rica • 8 females, CNC. Voucher codes: DHJPAR0042931, DHJPAR0042940, DHJPAR0049085, CNC1179988, CNC1179989 (additional specimens in a gel capsule associated with that specimen), CNC958826, CNC958827, CNC958828 (additional specimens in a gel capsule associated with that specimen).

**Figure 31.**
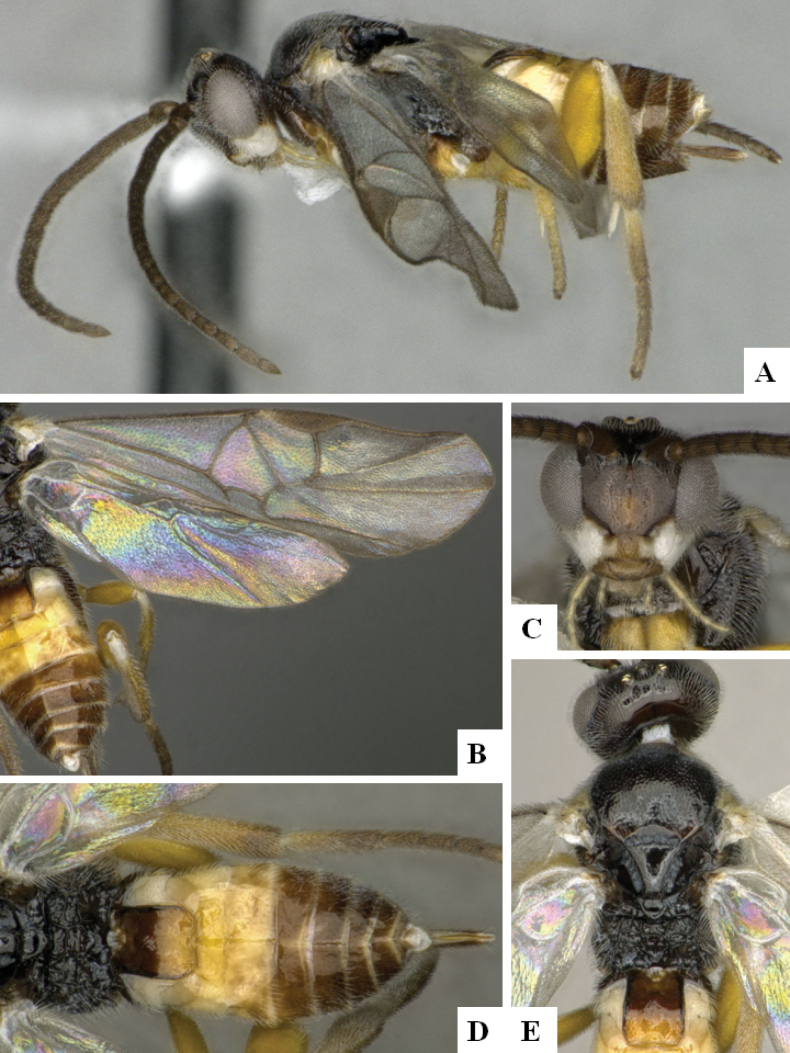
*Alphomeloneldaarayae* Fernandez-Triana & Shimbori holotype female CNC1179987 **A** habitus, lateral **B** wings **C** head, frontal **D** propodeum and metasoma, dorsal **E** mesosoma, dorsal.

##### Distribution.

Costa Rica (ACG).

##### Biology.

Gregarious, reared from *Neoxeniades* Burns03, *Neoxeniades* Burns04.

##### DNA barcoding.

BINBOLD:AAE2229.

##### Etymology.

Named in honor of Sra. Elda Araya in honor of her decades of teamwork in the ACG parataxonomist team.

##### Diagnostic description.

White patch on gena: extending to occiput and onto clypeus. Tegula/humeral complex color: yellow/yellow. Mesonotum color: mostly dark brown to black. Metasoma color: with several tergites orange-yellow, some laterotergites and sternites yellow, rest mostly brown. Tarsal claws spines: 3. Pterostigma shape: comparatively less elongate, its length ≤ 2.5× its central height and usually more rounded with at least one of its lower margins curved. T1 sculpture: entirely to mostly smooth. T1 central ridge: marked by weak carina. T2 sculpture: entirely to mostly smooth. Ovipositor sheaths length: shorter than first segment of metatarsus. Body length: 3.1–3.3 mm. Fore wing length: 3.0–3.5 mm.

#### ﻿ Alphomelon eliethcantillanoae

Taxon classificationAnimaliaHymenopteraBraconidae

Fernandez-Triana & Shimbori sp. nov.

8B2A322B-23E5-5851-8ECF-F97C7C3FDFB7

https://zoobank.org/E0172A4B-A32D-4500-BCAD-60D76DBDD1D8

Fig. 32A–E

##### Type material.

***Holotype*.** Costa Rica • Female, CNC; Guanacaste, Area de Conservación Guanacaste, Sector Pitilla, Cano, 10°59'43.44"N, 85°23'59.28"W, 490m; 9.X.2008; ex. *Nisoniadescastolus*; coll. Ronald Siezar; Voucher code: DHJPAR0031004; Host voucher code: 08-SRNP-72663.

##### Distribution.

Costa Rica (ACG).

##### Biology.

Solitary, reared from *Nisoniadescastolus* on *Lepidaploatortuosa* (Asteraceae).

##### DNA barcoding.

BINBOLD:AAC7653.

##### Etymology.

Named in honor of Sra. Elieth Cantillano in honor of her decades of teamwork in the ACG parataxonomist team.

##### Diagnostic description.

White patch on gena: extending to occiput but not to clypeus. Tegula/humeral complex color: yellow/yellow. Mesonotum color: mostly dark brown to black. Metasoma color: mostly black or dark brown. Tarsal claws spines: 1. Pterostigma shape: comparatively more elongate, its length ≥ 3.0× its central height and more triangular with its two lower margins more or less straight. T1 sculpture: entirely to mostly smooth. T1 central ridge: clearly marked by two raised carinae. T2 sculpture: weakly sculptured along margins. Ovipositor sheaths length: longer than first segment of metatarsus. Body length: 3.9 mm. Fore wing length: 4.0 mm.

**Figure 32.**
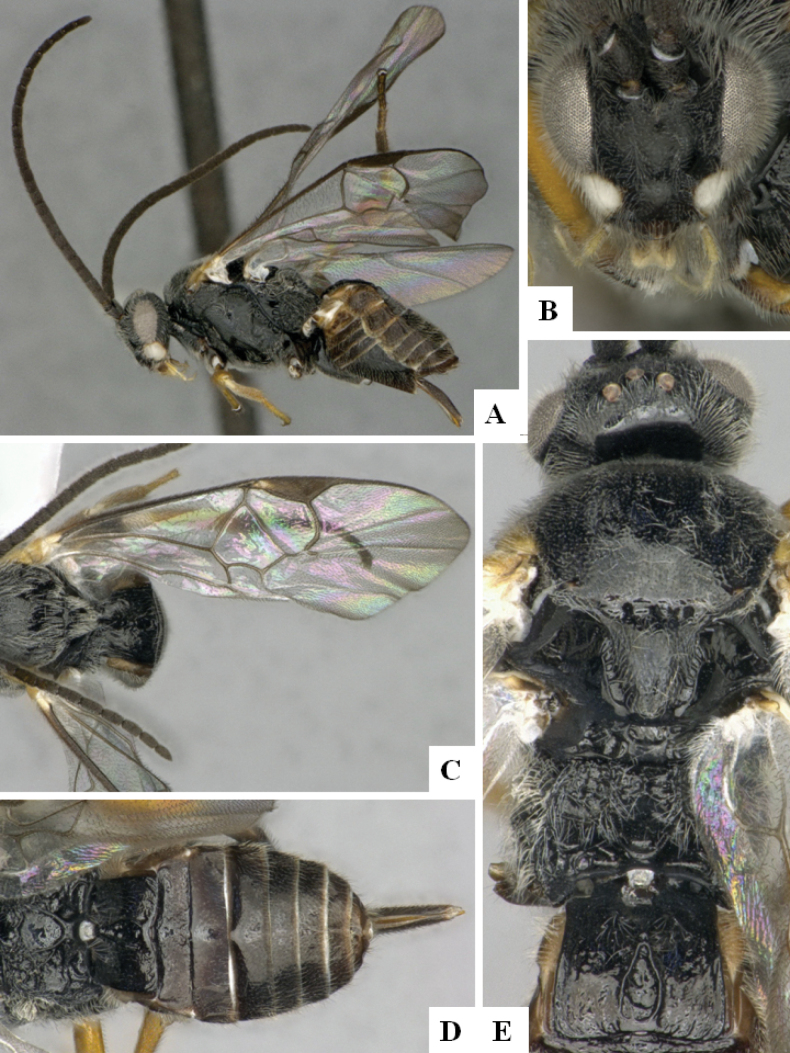
*Alphomeloneliethcantillanoae* Fernandez-Triana & Shimbori holotype female DHJPAR0031004 **A** habitus, lateral **B** head, frontal **C** fore wing **D** propodeum and metasoma, dorsal **E** mesosoma, dorsal.

#### ﻿ Alphomelon gloriasihezarae

Taxon classificationAnimaliaHymenopteraBraconidae

Fernandez-Triana & Shimbori sp. nov.

056BD236-B4D7-58CD-A89E-9F40D75A7635

https://zoobank.org/BFA2916D-563B-4FD4-8A30-7C1CEEE961E2

Figs 33A–E , 96A

##### Type material.

***Holotype*.** Costa Rica • Female, CNC; Alajuela, Area de Conservación Guanacaste, Sector San Cristobal, Estacion San Gerardo, 10°52'48.32"N, 85°23'19.93"W, 575m; 15.IV.2008; Malaise Trap; coll. Daniel Janzen & Winifred Hallwachs; Voucher code: DHJPAR0026269.

***Paratypes*.** Costa Rica • 1 female, 1 male, CNC; DHJPAR0012536, DHJPAR0039877.

##### Other specimens examined.

Mexico • 1 female, 1 male, CBG. Voucher codes: 07TAPACH-01674, 07TAPACH-01693.

##### Distribution.

Costa Rica (ACG), Mexico.

##### Biology.

Solitary, reared from *Cymaenesodiliatrebius*, *Morysmicythus*.

##### DNA barcoding.

BINBOLD:AAE5720.

##### Etymology.

Named in honor of Sra. Gloria Sihezar in honor of her decades of teamwork in the ACG parataxonomist team.

##### Diagnostic description.

White patch on gena: extending to occiput but not to clypeus. Tegula/humeral complex color: yellow/yellow. Mesonotum color: mostly dark brown to black. Metasoma color: mostly black or dark brown. Tarsal claws spines: 1. Pterostigma shape: comparatively less elongate, its length ≤ 2.5× its central height and usually more rounded with at least one of its lower margins curved. T1 sculpture: strongly sculptured on at least apical half or more. T1 central ridge: strongly marked by two raised carinae and strong costulae centrally. Ovipositor sheaths length: longer than first segment of metatarsus. Body length: 3.5–3.6 mm. Fore wing length: 3.5–3.7 mm.

**Figure 33.**
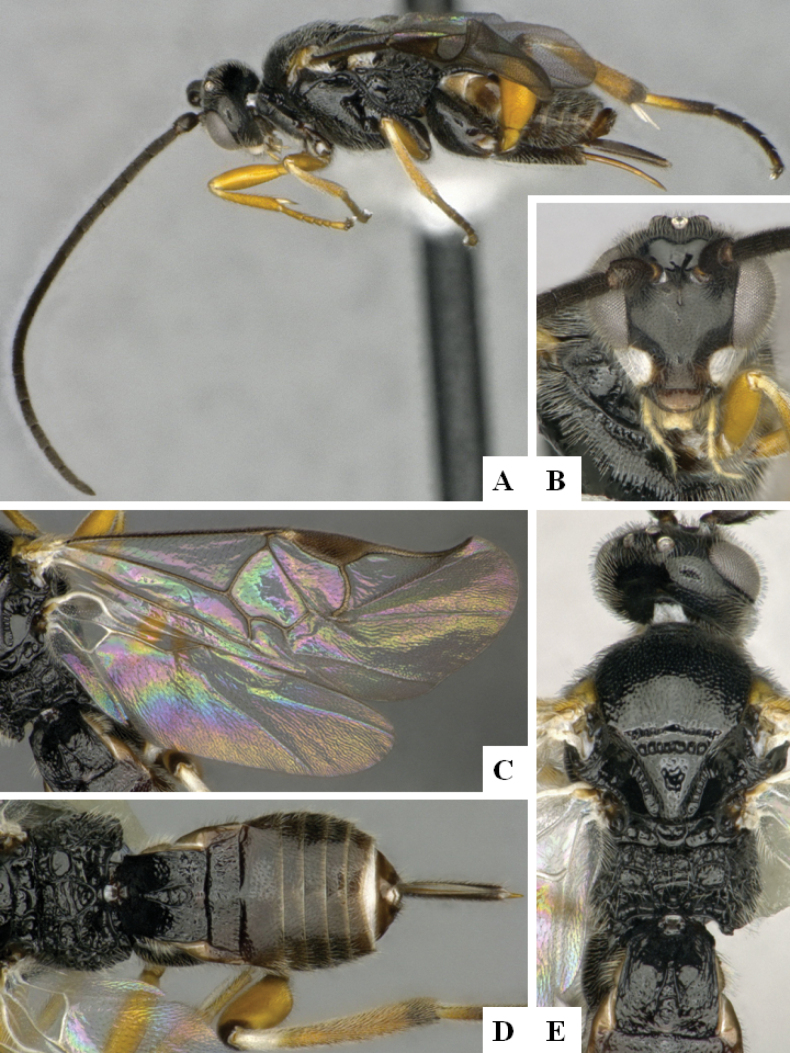
*Alphomelongloriasihezarae* Fernandez-Triana & Shimbori holotype female DHJPAR0026269 **A** habitus, lateral **B** head, frontal **C** wings **D** propodeum and metasoma, dorsal **E** mesosoma, dorsal.

##### Notes.

The specimens from Mexico (at least one sequence being a full barcode) are associated with the species because their sequences match perfectly with ACG specimens and the images available in BOLD are also similar.

#### ﻿ Alphomelon guillermopereirai

Taxon classificationAnimaliaHymenopteraBraconidae

Fernandez-Triana & Shimbori sp. nov.

6C2E549D-0A59-526B-9DBA-FEC240E9EFB1

https://zoobank.org/3AFF3D0E-CF69-406F-96A9-F44D53C505C1

**Figure 34.**
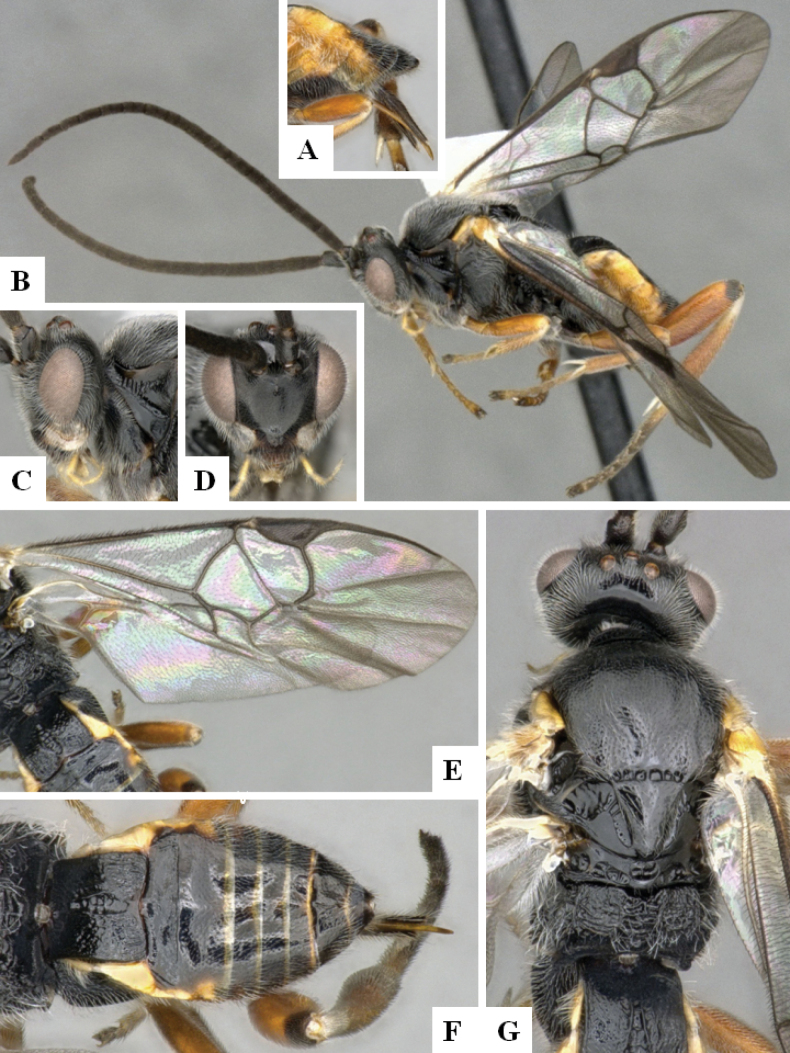
*Alphomelonguillermopereirai* Fernandez-Triana & Shimbori holotype female DHJPAR0049916 **A** ovipositor, lateral **B** habitus, lateral **C** head, lateral **D** head, frontal **E** wings **F** metasoma, dorsal **G** mesosoma, dorsal

**Figure 35.**
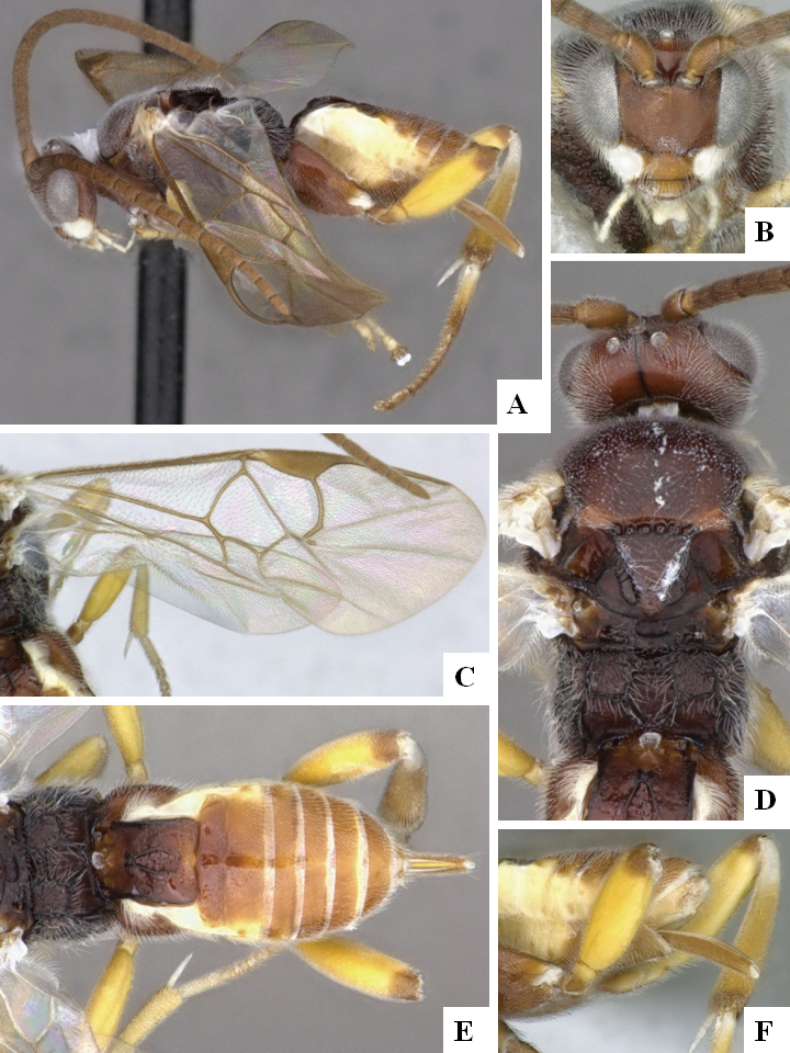
*Alphomelonguillermopereirai* Fernandez-Triana & Shimbori paratype female DHJPAR0054794 **A** habitus, lateral **B** head, frontal **C** wings **D** mesosoma, dorsal **E** propodeum and metasoma, dorsal **F** ovipositor, lateral.

**Figure 36.**
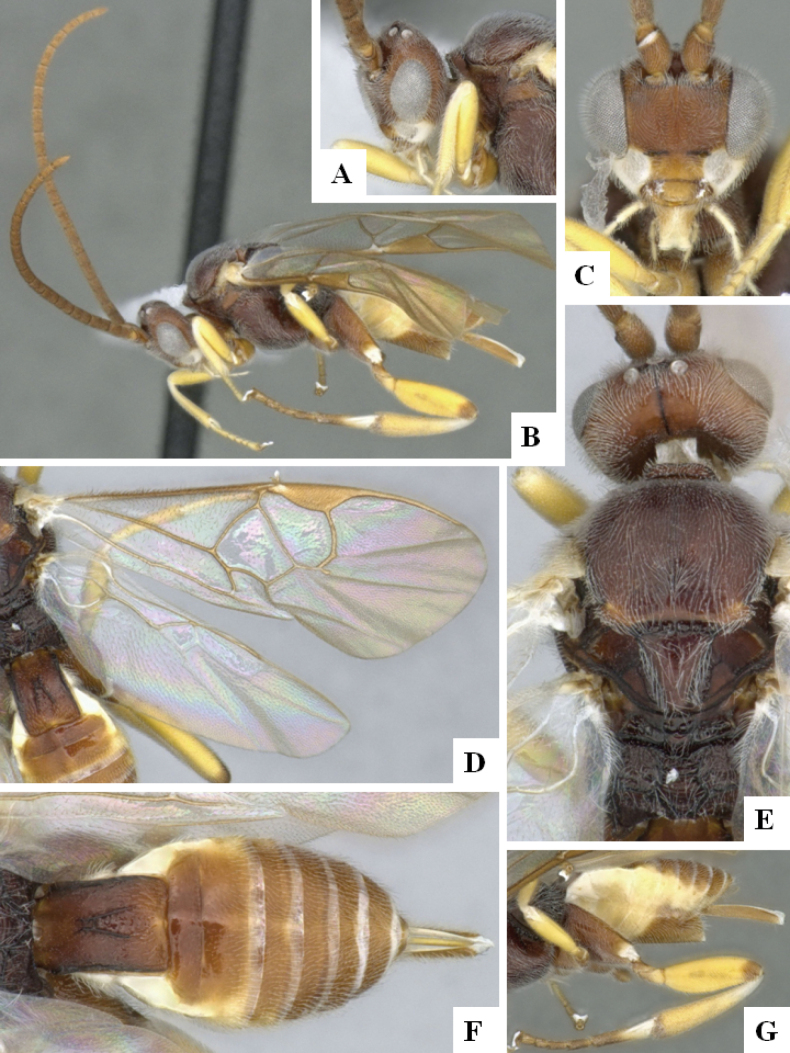
*Alphomelonguillermopereirai* Fernandez-Triana & Shimbori paratype female DHJPAR0054802 **A** head, lateral **B** habitus, lateral **C** head, frontal **D** wings **E** mesosoma, dorsal **F** metasoma, dorsal **G** ovipositor, lateral.

Figs 34A–G , 35A–F , 36A–G , 96B

##### Type material.

***Holotype*.** Costa Rica • Female, CNC; Alajuela, Area de Conservación Guanacaste, Sector Rincon Rain Forest, Jacobo, 10°56'26.88"N, 85°19'3.72"W, 461m; 22.VI.2012; ex: undetermined hesperiid with provisional name “hespJanzen01 Janzen55”; coll. Edwin Apu; Voucher code: DHJPAR0049916; Host voucher code: 12-SRNP-81025.

***Paratypes*.** Costa Rica • 12 females, 3 males, CNC. Voucher codes: DHJPAR0043075, DHJPAR0043077, DHJPAR0043086, DHJPAR0043087, DHJPAR0047235, DHJPAR0053881, DHJPAR0054794, DHJPAR0054802, DHJPAR0055561, DHJPAR0055293, DHJPAR0055325, CNC308812, CNC704669, CNC1065996, CNCHYM 00017.

##### Other specimens examined.

Costa Rica • 2 Females, CNC. Voucher codes: DHJPAR0026274, DHJPAR0049249.

##### Distribution.

Costa Rica (ACG and Peñas Blancas).

##### Biology.

Solitary, reared from *Mnasitheus* Janzen55, *Anthoptusepictetus*, *Anthoptusinsignis*, *Anthoptus* Burns33, *Corticeacorticea*, *Cymaenesodiliatrebius*.

##### DNA barcoding.

BINBOLD:AAB8584 (but see Notes below).

##### Etymology.

Named in honor of Sr. Guillermo Pereira honor of his decades of teamwork in the ACG parataxonomist team.

##### Diagnostic description.

White patch on gena: extending to occiput but not to clypeus. Tegula/humeral complex color: yellow/yellow. Mesonotum color: mostly dark brown to black. Metasoma color: with several tergites orange-yellow, some laterotergites and sternites yellow, rest mostly brown. Tarsal claws spines: 4. Pterostigma shape: comparatively less elongate, its length ≤ 2.5× its central height and usually more rounded with at least one of its lower margins curved. T1 sculpture: weakly sculptured along margins. T1 central ridge: marked by two raised carinae and strong costulae centrally. T2 sculpture: weakly sculptured along margins. Ovipositor sheaths length: longer than first segment of metatarsus. Body length: 4.1–4.5 mm. Fore wing length: 4.2–4.5 mm.

##### Notes.

The associated BIN includes at least three (possibly more) species, see further explanations and details under the treatment of *Alphomelonmelanoscelis*. Two ACG specimens (DHJPAR0026274 and DHJPAR0049249) have a darker coloration (they look similar to *A.andydeansi* and thus they would end in a different place of the key) and also have different DNA barcodes. They are excluded from the type series.

#### ﻿ Alphomelon hazelcambroneroae

Taxon classificationAnimaliaHymenopteraBraconidae

Fernandez-Triana & Shimbori sp. nov.

B3583096-3978-58F4-A3AA-8C6720B7740E

https://zoobank.org/2E74C6CB-79A4-4331-BF7C-1C9D927D6C30

**Figure 37.**
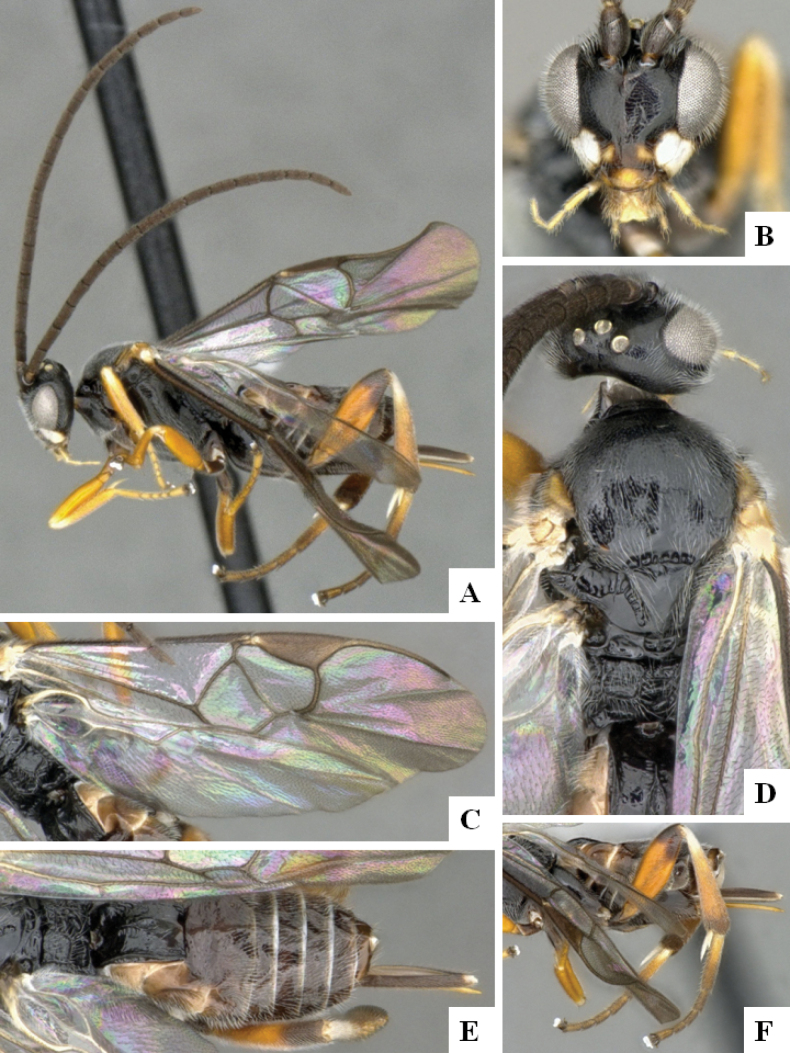
*Alphomelonhazelcambroneroae* Fernandez-Triana & Shimbori holotype female CNC308813 **A** habitus, lateral **B** head, frontal **C** wings **D** mesosoma, dorsal **E** metasoma, dorsal **F** metasoma, lateral.

**Figure 38.**
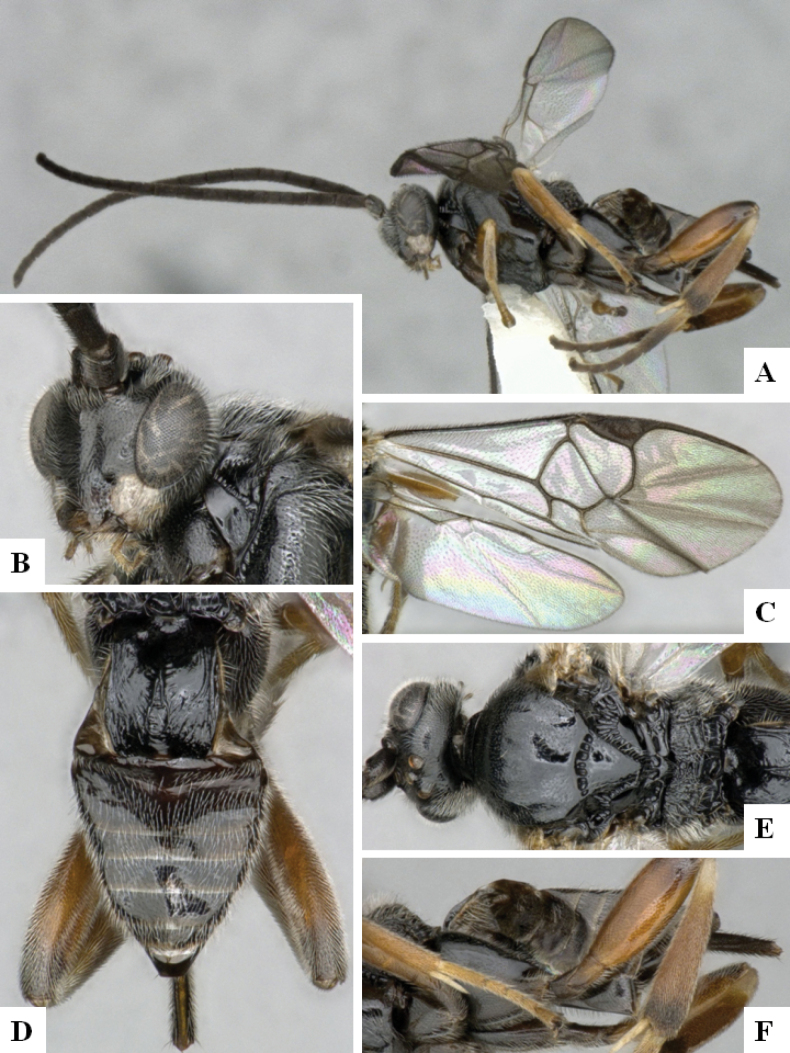
*Alphomelonhazelcambroneroae* Fernandez-Triana & Shimbori paratype female CNC308824 **A** habitus, lateral **B** head, fronto-lateral **C** wings **D** metasoma, dorsal **E** mesosoma, dorsal **F** metasoma, lateral.

**Figure 39.**
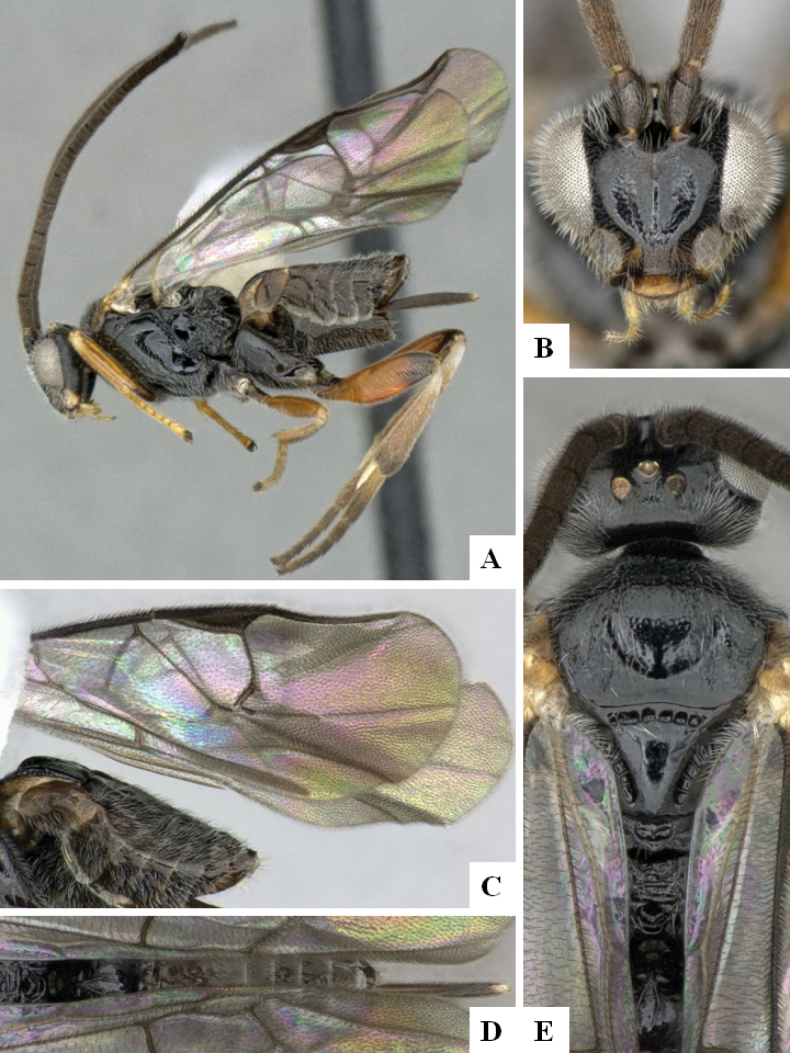
*Alphomelonhazelcambroneroae* Fernandez-Triana & Shimbori paratype female DHJPAR0051805 **A** habitus, lateral **B** head, frontal **C** wings **D** metasoma, dorsal **E** head and mesosoma, dorsal.

Figs 37A–F , 38A–F , 39A–E , 97A

##### Type material.

***Holotype*.** Costa Rica • Female, CNC; Guanacaste, Area de Conservación Guanacaste, Sector Pitilla, Sendero Mismo, 10°59'15.29"N, 85°25'10.81"W, 680m; 11.II.2013, ex. *Cyneamegalops*; coll. Manuel Rios; Voucher code: CNC308813; Host voucher code: 13-SRNP-30331.

***Paratypes*.** Costa Rica • 30 females, 3 males, CNC; DHJPAR0051805, DHJPAR0054746, DHJPAR0030704, DHJPAR0030714, CNC308819, CNC308820, CNC308821, CNC308822, CNC308823, CNC308824, CNC308825, CNC308826, CNC308817, CNC308818, CNC308814, CNC308815, CNC308816 (additional specimens in a gel capsule associated with that specimen), DHJPAR0053717, DHJPAR0050981, CNC308827, CNC308828, CNC308829, CNC308830, CNC308831 (additional specimens in a gel capsule associated with that specimen), DHJPAR0050090, CNC308832, CNC308833, CNC308834 (additional specimens in a gel capsule associated with that specimen), DHJPAR0026885, DHJPAR0048159, DHJPAR0053774, CNC308835, CNC308836.

##### Distribution.

Costa Rica (ACG).

##### Biology.

Gregarious, reared from *Calpodesfusta*, *C.severus*, *Cyneaanthracinus*, *C.cynea*, *C.irma*, *C.megalops*, *Cynea* Burns02, *Cynea* Burns04, *Cynea* Burns05, *Cynea* Burns06, *CyneaCynea* Burns11, *Rhinthonmolion*, and *R.osca*.

##### DNA barcoding.

BINBOLD:AAA6775.

##### Etymology.

Named after Sra. Hazel Cambronero in honor of her decades of teamwork in the ACG parataxonomist team.

##### Diagnostic description.

White patch on gena: extending to occiput and onto clypeus. Tegula/humeral complex color: yellow/yellow. Mesonotum color: mostly dark brown to black. Metasoma color: mostly black or dark brown. Tarsal claws spines: 3 or 4. Pterostigma shape: comparatively more elongate, its length ≥ 3.0× its central height and more triangular with its two lower margins more or less straight. T1 sculpture: weakly sculptured along margins (but some specimens, including holotype, with T1 mostly smooth). T1 central ridge: clearly marked by two raised carinae. T2 sculpture: entirely to mostly smooth. Ovipositor sheaths length: longer than first segment of metatarsus. Body length: 3.7–4.4 mm. Fore wing length: 3.9–4.4 mm.

#### ﻿ Alphomelon itatiaiensis

Taxon classificationAnimaliaHymenopteraBraconidae

Shimbori & Fernandez-Triana sp. nov.

1319FBA5-D15D-5A14-9B41-0BFAD7DA0ECB

https://zoobank.org/799B3BD2-A218-472F-8DC3-AABA9CA91A69

Figs 40A–G , 41A–E

##### Type material.

***Holotype*.** Brazil • Female (DCBU 100574): “Itatiaia, RJ, Brasil / PARNA de Itatiaia / Projeto Pensa Rio - Hympar / S 22o25'42,6” W 44o37'42,2” / Armadilha Malaise 03 (1.442 m) / 21.XI.2013 / R.F. Monteiro col.”

***Paratypes*.** 1 female, same as holotype (DCBU 100575); 1 female, same as holotype, except “... 22°26'1,4"S 44°36'49,5"W / Armadilha malaise 01 (1070 m) / 27.II.2014 …” (DCBU 100571); 1 female, same as holotype except “... 22°25'20,2"S 44°38'10"W Armadilha Malaise 04 (1.642m) / 14.IX.2013 …” (DCBU 100578); 1 female: “Itatiaia, RJ, Brasil / PARNA de Itatiaia / 22°26'16,5"S 44°36'41,4"W / Armadilha Malaise 02 (987 m) / 30.I.2012 / R.F. Monteiro col.” (DCBU 100577).

##### Distribution.

Brazil.

##### Biology.

No data.

##### DNA barcoding.

Not available.

##### Etymology.

Named after the locality of collection of the holotype and paratype specimens.

##### Diagnostic description.

White patch on gena: extending to occiput but not to clypeus. Tegula/humeral complex color: yellow/yellow. Mesonotum color: mostly dark brown to black. Metasoma color: mostly black or dark brown. Tarsal claws spines: 2 or 3. Pterostigma shape: comparatively less elongate, its length ~ 2.5× its central height and usually more rounded with at least one of its lower margins curved. T1 sculpture: entirely to mostly smooth. T1 central ridge: clearly marked [with 1 or 2 strong costula centrally; costula absent in smaller specimens]. T2 sculpture: entirely to mostly smooth. Ovipositor sheaths length: slightly longer than first segment of metatarsus. Body length: 3.7–4.8 mm. Fore wing length: 3.8–4.8 mm.

**Figure 40.**
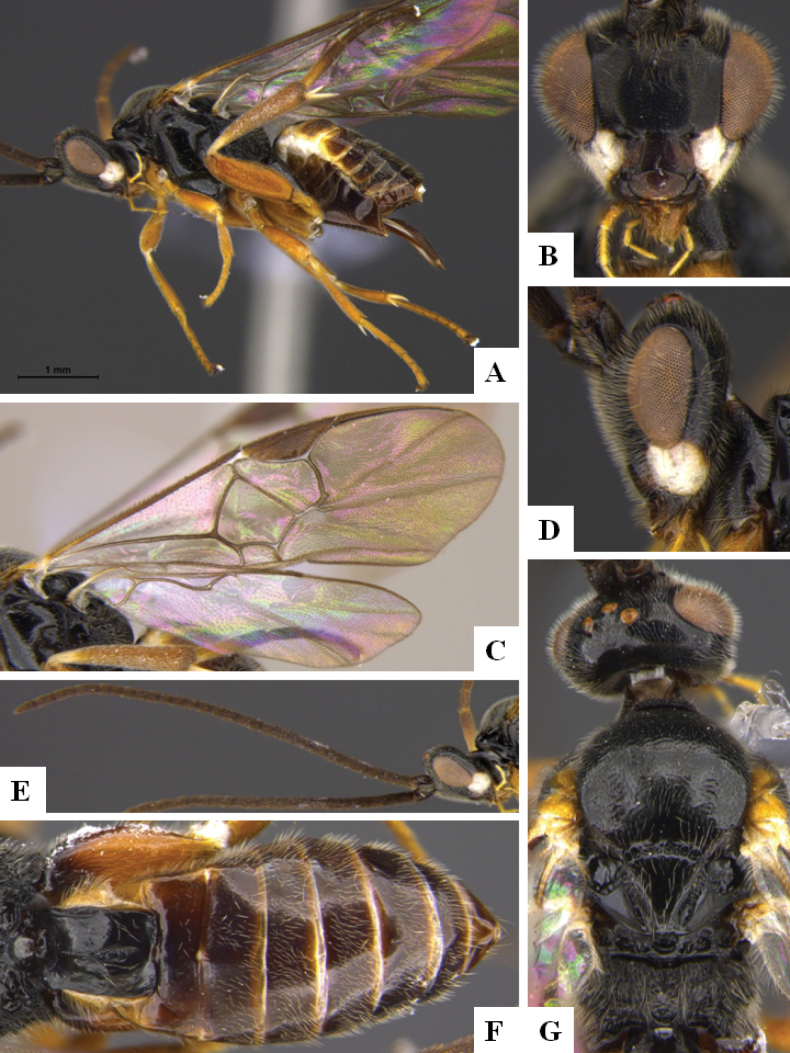
*Alphomelonitatiaiensis* Shimbori & Fernandez-Triana holotype female DCBU 100574 (**A to E**) and paratype female DCBU 100575 (**F, G**) **A** habitus, lateral **B** head, frontal **C** wings **D** head, lateral **E** antenna, lateral **F** metasoma, dorsal **G** mesosoma, dorsal.

**Figure 41.**
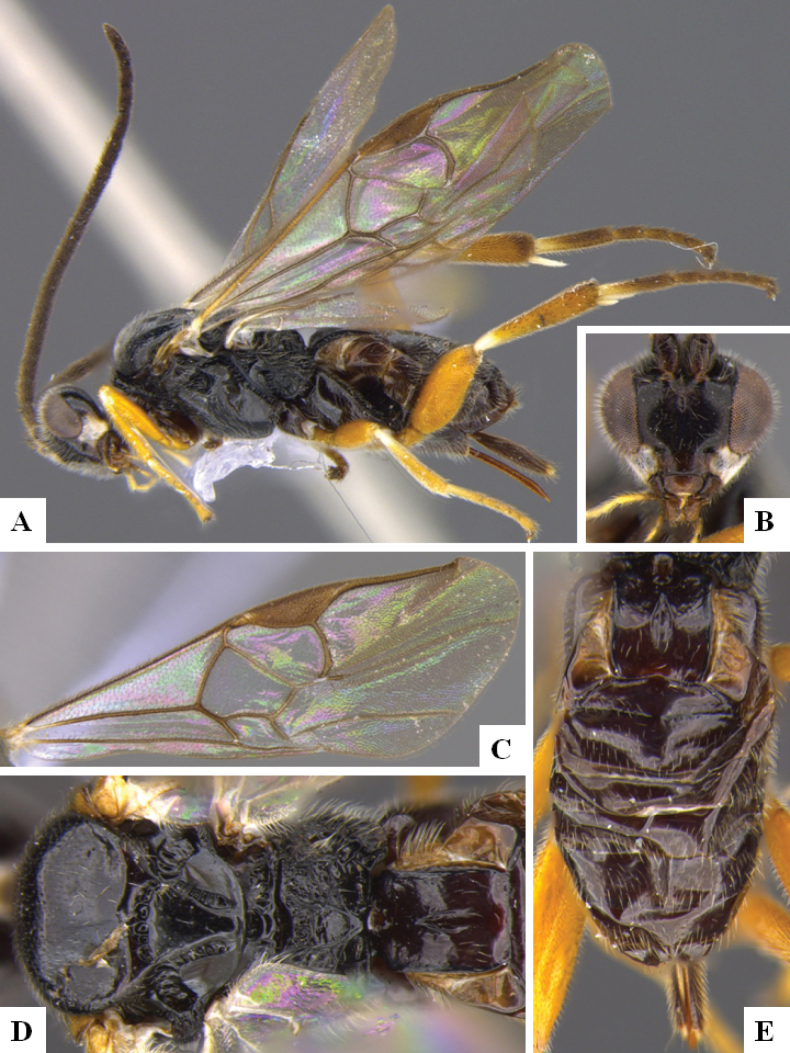
*Alphomelonitatiaiensis* Shimbori & Fernandez-Triana paratype female DCBU 100571 (**A, B**) and paratype female DCBU 100577 (**C–E**) **A** habitus, lateral **B** head, frontal **C** fore wing **D** mesosoma, dorsal **E** metasoma, dorsal.

#### ﻿ Alphomelon josecortesi

Taxon classificationAnimaliaHymenopteraBraconidae

Fernandez-Triana & Shimbori sp. nov.

5EC3961B-B2B0-5894-995D-F1E431EFAA26

https://zoobank.org/D3242314-8BDA-4EF2-ABD7-A75AD54D7890

**Figure 42.**
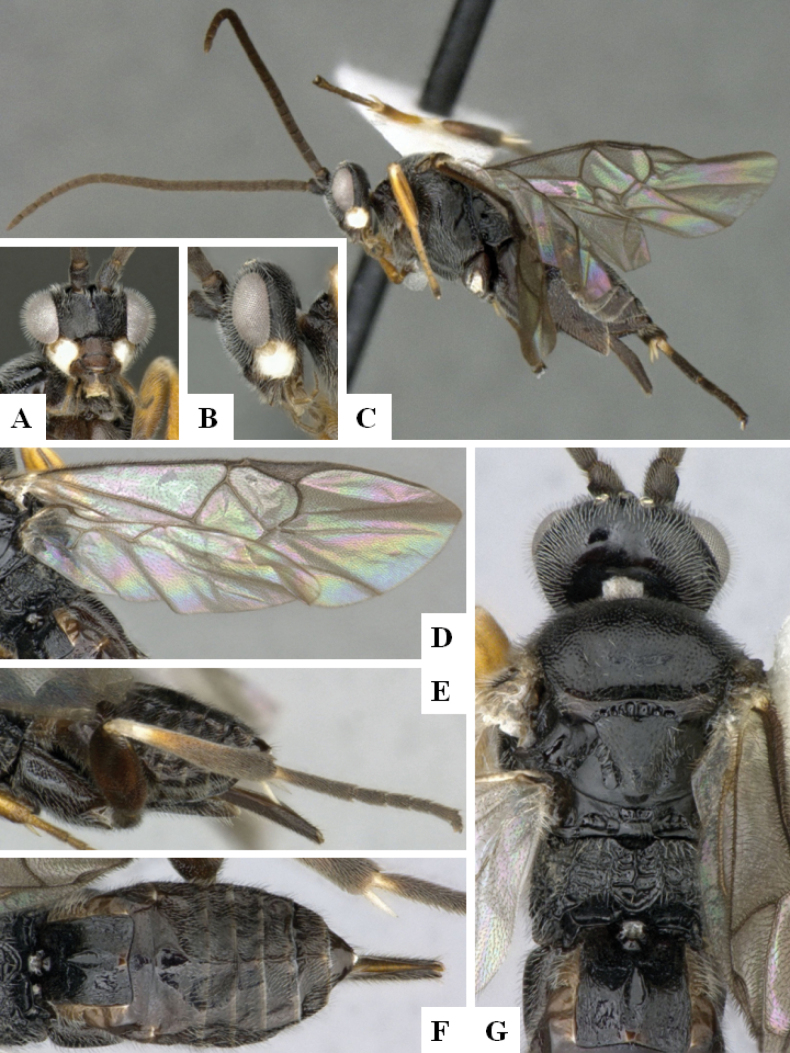
*Alphomelonjosecortesi* Fernandez-Triana & Shimbori holotype female DHJPAR0051219 **A** head, frontal **B** head, lateral **C** habitus, lateral **D** wings **E** metasoma, lateral **F** metasoma, dorsal **G** mesosoma, dorsal.

**Figure 43.**
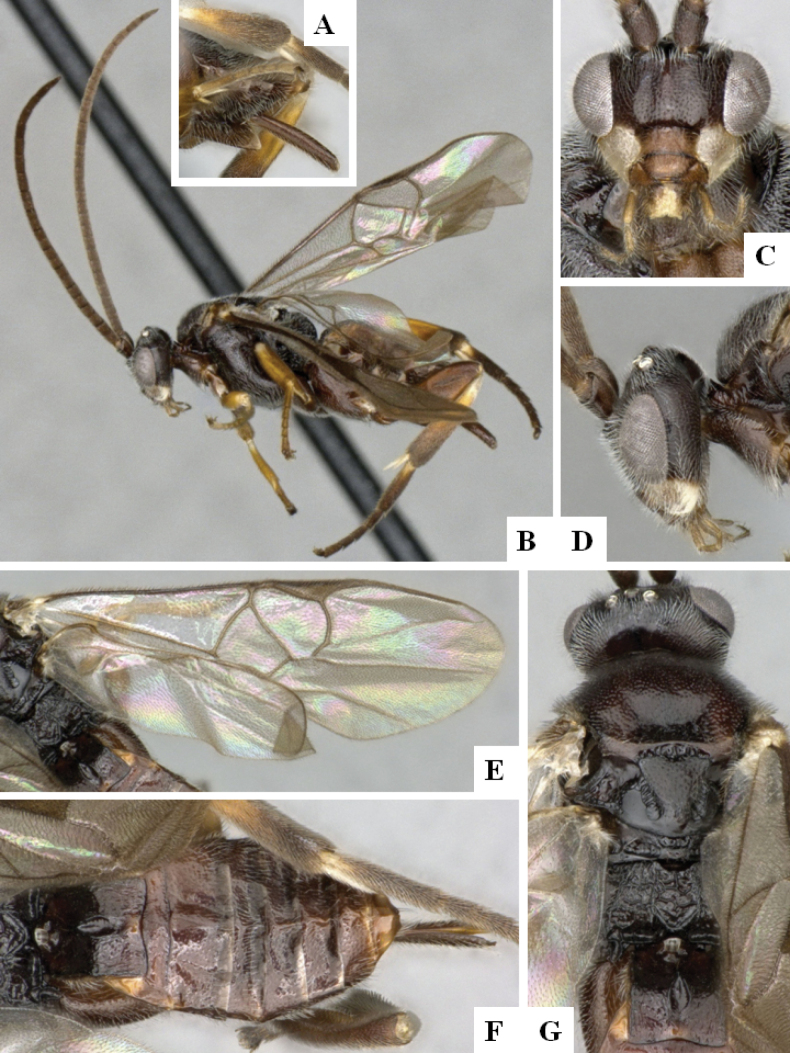
*Alphomelonjosecortesi* Fernandez-Triana & Shimbori paratype female CNC1802002 **A** ovipositor, lateral **B** habitus, lateral **C** head, frontal **D** head, lateral **E** wings **F** metasoma, dorsal **G** mesosoma, dorsal.

Figs 42A–G , 43A–G , 48A–F , 97B , 99B

##### Type material.

***Holotype*.** Costa Rica • Female, CNC; Guanacaste, Area de Conservación Guanacaste, Sector Pitilla, Sendero Orosilito, 10°58'59.88"N, 85°26'10.32"W, 900m; 14.I.2013; ex. *Verticasubrufescens*; coll. Freddy Quesada; Voucher code: DHJPAR0051219; Host voucher code: 13-SRNP-30133.

***Paratypes*.** Costa Rica • 37 females, 3 males, CNC; CNC1801997, CNC1801998, CNC1801999, CNC1802000, CNC1802001, CNC1802002, CNC1802003, DHJPAR0041756, DHJPAR0052937, CNC1802004, CNC1802005, CNC1802006, CNC1802007, CNC1802008, CNC1802009, DHJPAR0051213, CNC1802010, CNC1802011, CNC1802012, CNC1802013, CNC1802014, CNC1802015, CNC1802016, CNC1802017, CNC1802018, CNC1802019, CNC1802020, DHJPAR0055445, CNC1802021, CNC1802022, CNC1802023, CNC1802024, CNC1802025, CNC1802026, CNC1802027, CNC1802028, CNC958823, CNC958824, CNC958825 (additional specimens in a gel capsule associated with that specimen), DHJPAR0034230.

##### Distribution.

Costa Rica (ACG).

##### Biology.

Gregarious, reared from *Verticasubrufescens* on at least 34 palm species (Arecaceae).

##### DNA barcoding.

BINBOLD:AAB6733.

##### Etymology.

Named in honor of Sr. José Cortés in honor of his decades of teamwork in the ACG parataxonomist team.

##### Diagnostic description.

White patch on gena: extending to occiput and onto clypeus. Tegula/humeral complex color: yellow/brown or brown/brown. Mesonotum color: mostly dark brown to black. Metasoma color: mostly black or dark brown. Tarsal claws spines: 3 or 4. Pterostigma shape: comparatively more elongate, its length ≥ 3.0× its central height and more triangular with its two lower margins more or less straight. T1 sculpture: strongly sculptured on at least apical half or more. T1 central ridge: clearly marked by two raised carinae. T2 sculpture: entirely to mostly smooth. Ovipositor sheaths length: longer than first segment of metatarsus. Body length: 4.1–4.2 mm. Fore wing length: 4.0–4.3 mm.

#### ﻿ Alphomelon keineraragoni

Taxon classificationAnimaliaHymenopteraBraconidae

Fernandez-Triana & Shimbori sp. nov.

C6302362-B02A-58C5-8F56-9CDB2A409285

https://zoobank.org/D05A016F-080A-4430-BB69-3EC3E86C6F8E

Figs 44A–G , 98A

##### Type material.

***Holotype*.** Costa Rica • Female, CNC; Alajuela, Area de Conservación Guanacaste, Sector Rincon Rain Forest, Jacobo, 10°56'26.88"N, 85°19'3.72"W, 461m; 1.V.2010; ex: *Neoxeniadespluviasilva*; coll. Noe Castillo; Voucher code: DHJPAR0041820; Host voucher code: 10-SRNP-69422.

***Paratypes*.** Costa Rica • 11 females, 1 male, CNC. Voucher codes: DHJPAR0038957, DHJPAR0038962, DHJPAR0047035, DHJPAR0047120, CNC308804, CNC308805, CNC308806 (additional specimens in a gel capsule associated with that specimen), CNC308807, CNC308808, CNC308809 (additional specimens in a gel capsule associated with that specimen), CNC308810, CNC308811.

##### Distribution.

Costa Rica (ACG).

##### Biology.

Gregarious, reared from *Neoxeniadespluviasilva* and *Neoxeniades* Burns03.

##### DNA barcoding.

BINBOLD: ABU7420.

##### Etymology.

Named in honor of Sr. Keiner Aragón in honor of his decades of teamwork in the ACG inventory team.

##### Diagnostic description.

White patch on gena: extending to occiput and onto clypeus. Tegula/humeral complex color: yellow/yellow. Mesonotum color: mostly dark brown to black. Metasoma color: mostly black or dark brown. Tarsal claws spines: 4. Pterostigma shape: comparatively more elongate, its length ≥ 3.0× its central height and more triangular with its two lower margins more or less straight. T1 sculpture: entirely to mostly smooth/weakly sculptured along margins/ strongly sculptured on at least apical half or more. T1 central ridge: marked by weak carina. T2 sculpture: entirely to mostly smooth. Ovipositor sheaths length: shorter than first segment of metatarsus. Body length: 2.9–3.7 mm. Fore wing length: 3.2–3.8 mm.

**Figure 44.**
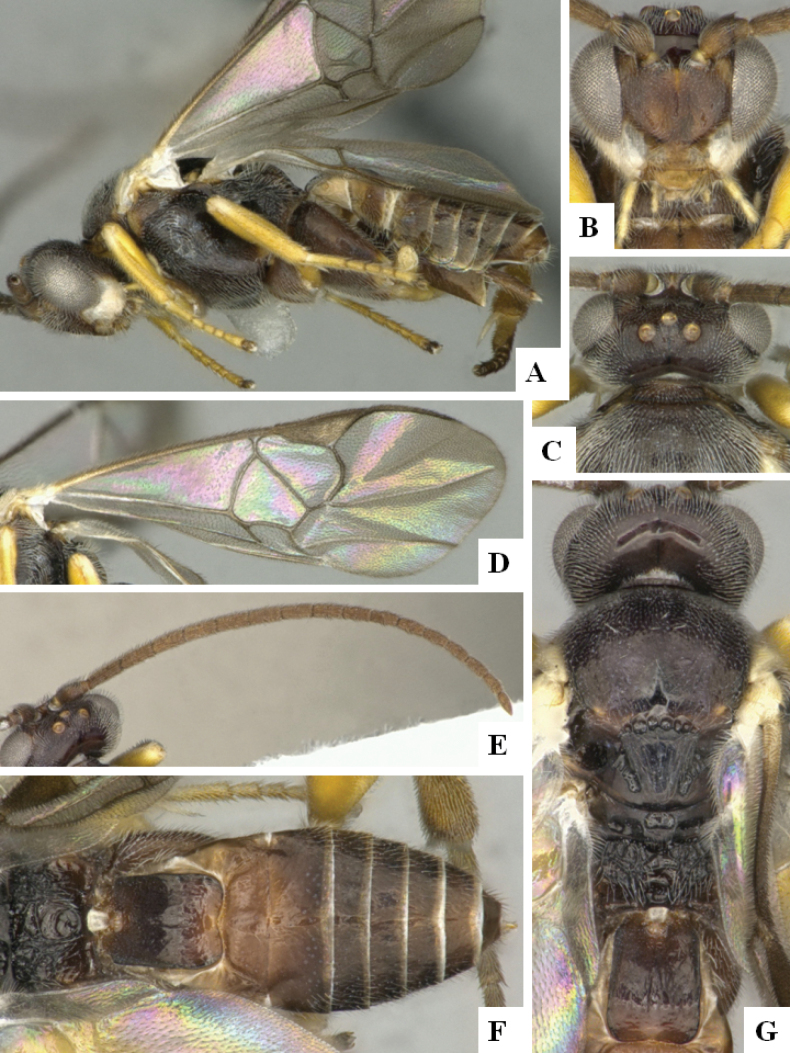
*Alphomelonkeineraragoni* Fernandez-Triana & Shimbori holotype female DHJPAR0041820 **A** habitus, lateral **B** head, frontal **C** head, dorsal **D** fore wing **E** antenna, lateral **F** propodeum and metasoma, dorsal **G** mesosoma, dorsal.

##### Notes.

*Alphomelonbromeliphile* and *A.keineraragoni* are very similar morphologically. However, *bromeliphile* parasitizes caterpillars of *Neoxeniadesluda* feeding on epiphytic Bromeliaceae in rain forest, whereas *A.keineraragoni* parasitizes different *Neoxeniades* species (*N.pluviasilva* and *N.* Burns03) feeding on terrestrial Bromeliaceae in dry forest. They also have distinctive BINs, which are 2.72% bp different.

#### ﻿ Alphomelon luciarosae

Taxon classificationAnimaliaHymenopteraBraconidae

Fernandez-Triana & Shimbori sp. nov.

799E2A06-DD2E-5C40-AF57-F291FC46AAE0

https://zoobank.org/7AB920E4-02BE-40D6-9843-68176A91782B

Figs 45A–G , 98B

##### Type material.

***Holotype*.** Costa Rica • Female, CNC; Alajuela, Area de Conservación Guanacaste, Brasilia, Piedrona, 11°0'58.32"N, 85°21'32.40"W, 340m; 4.XII.2012; ex: *Ebususebusus*; coll. Duvalier Briceño; Voucher code: DHJPAR0051292; Host voucher code: 12-SRNP-65952.

##### Distribution.

Costa Rica (ACG).

##### Biology.

Gregarious, reared from *Ebususebusus*.

##### DNA barcoding.

BINBOLD:ACJ4259.

##### Etymology.

Named in honor of Sra. Lucía Rosa in honor of her decades of teamwork in the ACG parataxonomist team.

##### Diagnostic description.

White patch on gena: extending to occiput but not to clypeus. Tegula/humeral complex color: yellow/yellow. Mesonotum color: mostly dark brown to black. Metasoma color: mostly black or dark brown. Tarsal claws spines: 3. Pterostigma shape: comparatively more elongate, its length ≥ 3.0× its central height and more triangular with its two lower margins more or less straight. T1 sculpture: entirely to mostly smooth. T1 central ridge: faintly indicated by shallow depression. T2 sculpture: entirely to mostly smooth. Ovipositor sheaths length: shorter than first segment of metatarsus. Body length: 3.8 mm. Fore wing length: 3.8 mm.

**Figure 45.**
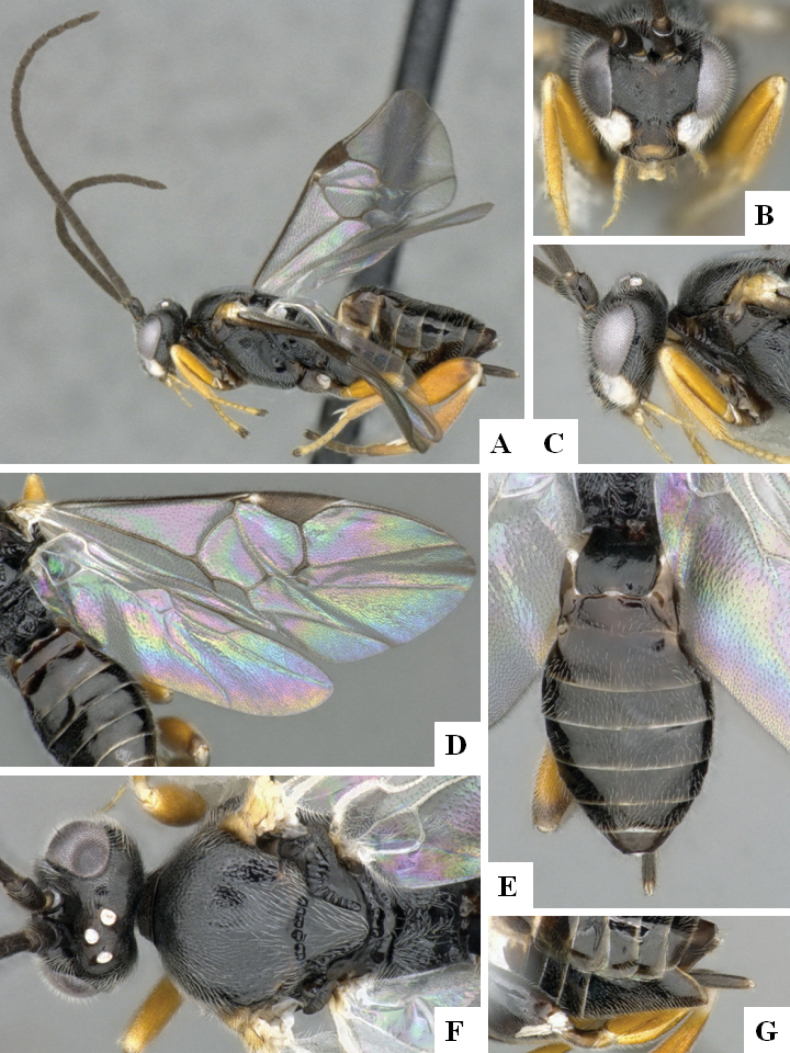
*Alphomelonluciarosae* Fernandez-Triana & Shimbori holotype female DHJPAR0051292 **A** habitus, lateral **B** head, frontal **C** head, lateral **D** wings **E** metasoma, dorsal **F** mesosoma, dorsal **G** ovipositor, lateral.

#### ﻿ Alphomelon manuelriosi

Taxon classificationAnimaliaHymenopteraBraconidae

Fernandez-Triana & Shimbori sp. nov.

85161723-453C-5C94-BDD3-F676894B8CCC

https://zoobank.org/8C18B545-6439-4956-9CD0-DC06E9CA85E8

Figs 46A–G , 99A

##### Type material.

***Holotype*.** Costa Rica • Female, CNC; Alajuela, Area de Conservación Guanacaste, Sector Rincon Rain Forest, Palomo, 10°57'42.84"N, 85°16'49.44"W, 96m; 2.X.2013; ex: *Cymaenesodiliatrebius*; coll. Keiner Aragón; Voucher code: DHJPAR0053843; Host voucher code: 13-SRNP-76649.

***Paratypes*.** Costa Rica • 1 female, 3 males, CNC. Voucher codes: DHJPAR0049470, DHJPAR0051849, DHJPAR0053809, DHJPAR0053818.

##### Distribution.

Costa Rica (ACG).

##### Biology.

Solitary, reared from *Corticealysias*, *Cymaenesodiliatrebius*, *Parphorusdecora*, and *Parphorusstorax*.

##### DNA barcoding.

BINBOLD:ABX0806.

##### Etymology.

Named in honor of Sr. Manuel Ríos in honor of his decades of teamwork in the ACG parataxonomist team.

##### Diagnostic description.

White patch on gena: extending to occiput and onto clypeus. Tegula/humeral complex color: dark brown/brown. Mesonotum color: mostly dark brown to black. Metasoma color: mostly black or dark brown. Tarsal claws spines: 2. Pterostigma shape: comparatively more elongate, its length ≥ 3.0× its central height and more triangular with its two lower margins more or less straight. T1 sculpture: entirely to mostly smooth. T1 central ridge: marked by weak carina. T2 sculpture: entirely to mostly smooth. Ovipositor sheaths length: longer than first segment of metatarsus. Body length: 3.5–4.2 mm. Fore wing length: 3.7–4.3 mm.

**Figure 46.**
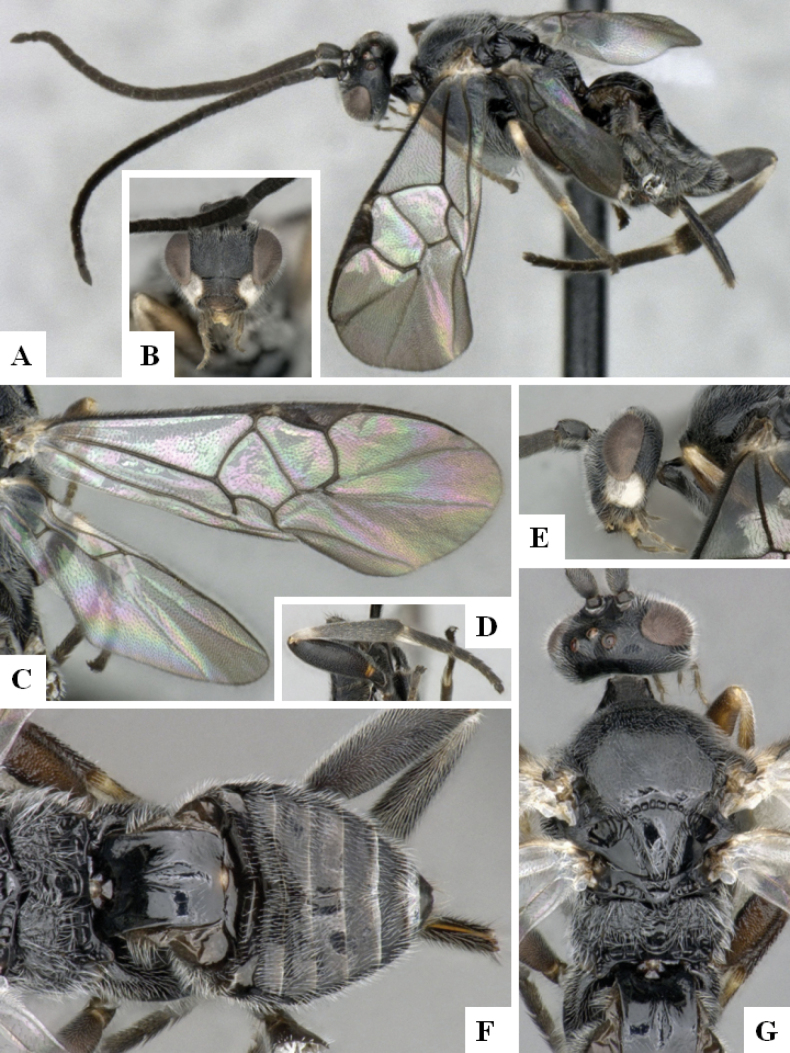
*Alphomelonmanuelriosi* Fernandez-Triana & Shimbori holotype female DHJPAR0053843 **A** habitus, lateral **B** head, frontal **C** wings **D** hind leg, lateral **E** head, lateral **F** propodeum and metasoma, dorsal **G** mesosoma, dorsal.

**Figure 47.**
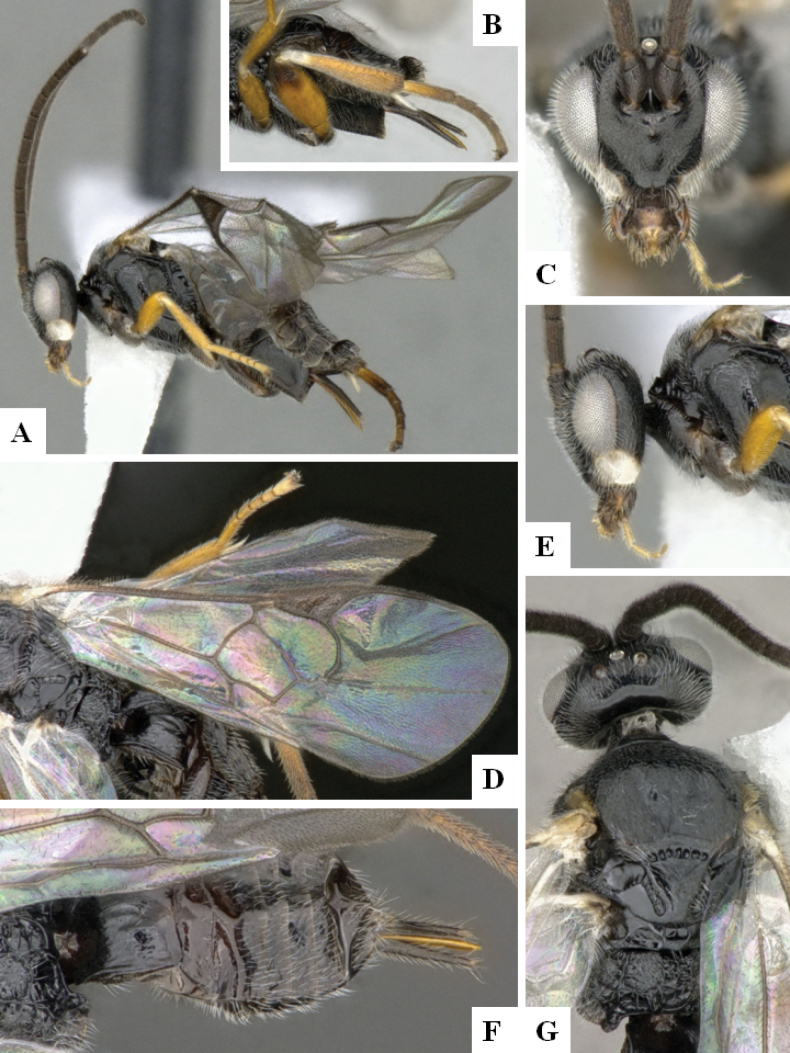
*Alphomelonxestopyga* Deans female DHJPAR0031632 **A** habitus, lateral **B** metasoma, lateral **C** head, frontal **D** wings **E** head, lateral **F** metasoma, dorsal **G** mesosoma, dorsal.

**Figure 48.**
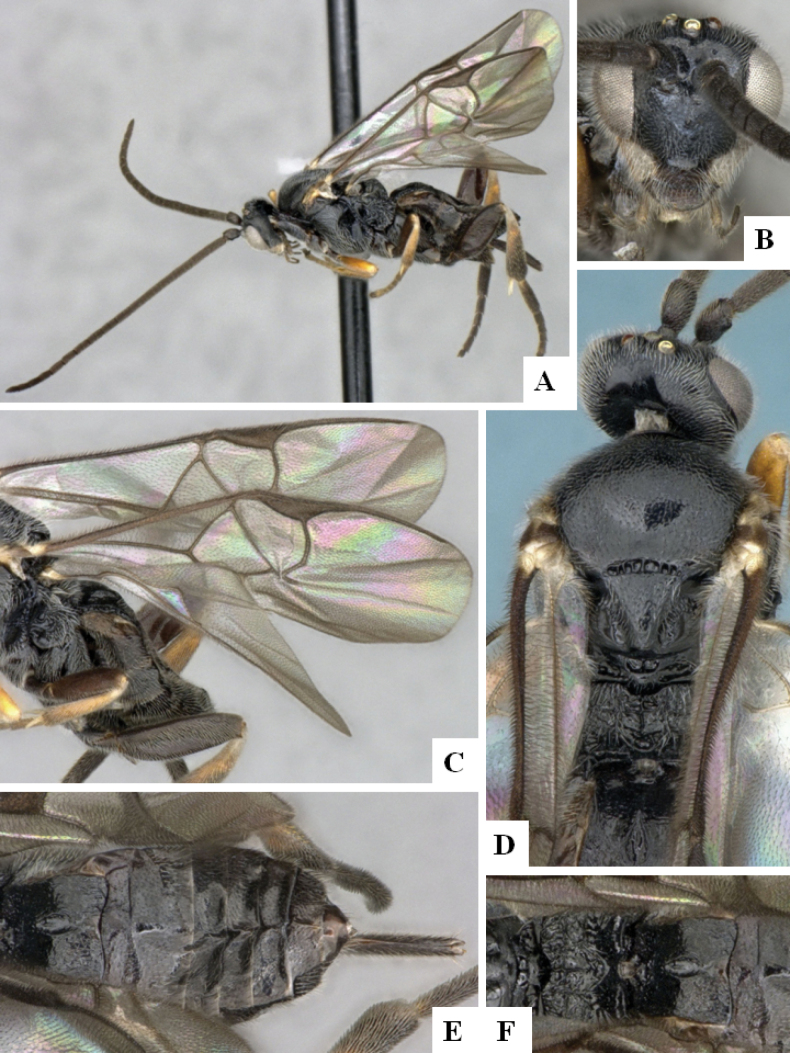
*Alphomelonjosecortesi* Fernandez-Triana & Shimbori paratype female DHJPAR0051213. **A** habitus, lateral **B** head, frontal **C** wings **D** mesosoma, dorsal **E** metasoma, dorsal **F** propodeum and T1, dorsal.

#### ﻿ Alphomelon melanoscelis

Taxon classificationAnimaliaHymenopteraBraconidae

Deans, 2003

277C6CFB-1F10-5FC3-B19F-528AFEC381CE

Figs 49A–D , 50A–E

##### Distribution.

Argentina*, Brazil (AL, MT), Costa Rica, Mexico, Venezuela; collected in dry forest and rainforest sites.

##### Biology.

Reared from unidentified hesperiines feeding on grasses (Poaceae) and sedges (Cyperaceae) in Costa Rica (Deans et al. 2003).

##### DNA barcoding.

BINBOLD:AAB8584 (but see Notes below).

##### Other specimens examined.

See notes below.

##### Notes.

BINBOLD:AAB8584 comprises what we consider to be several species, namely a) a few specimens of the “true” *A.melanoscelis* (in the restricted sense as it is understood in this paper), from Central and South America; b) a single specimen of *A.paramelanoscelis* and two specimens of *A.rigoi*, two new South American species described below; c) Costa Rican (ACG) specimens of *A.guillermopereirai*, a completely different species morphologically and which represents the majority of specimens currently associated with that BIN in BOLD. This BIN has a relatively large barcode variation, with the maximum distance between sequences within the BIN being 1.93% bp, a larger difference than the distance to the closest BIN in BOLD (BOLD:ADJ6568) which is only 1.08% bp. That second BIN represents *A.andydeansi*, a species with comparatively significant morphological differences (especially paler color of hind legs) and different host caterpillars.

Based strictly on the specimens of these species that are deposited in the CNC and that we could examine and study, the *A.melanoscelis* specimens cluster separate from specimens of *A.guillermopereirai* (although the barcode differences between the two species are < 1% bp). This is an example of BINs failing to properly represent the species limits (as compared versus other criteria to delimit species such as morphology and biology). BINs containing more than one species have been commonplace with ACG speciose genera (e.g., Hebert et al. 2004, Burns et al. 2008, Fernandez-Triana 2019) although the discussion of the topic among the scientific community is ongoing and far from being resolved (e.g., Brower 2010; Meier et al. 2021).

**Figure 49.**
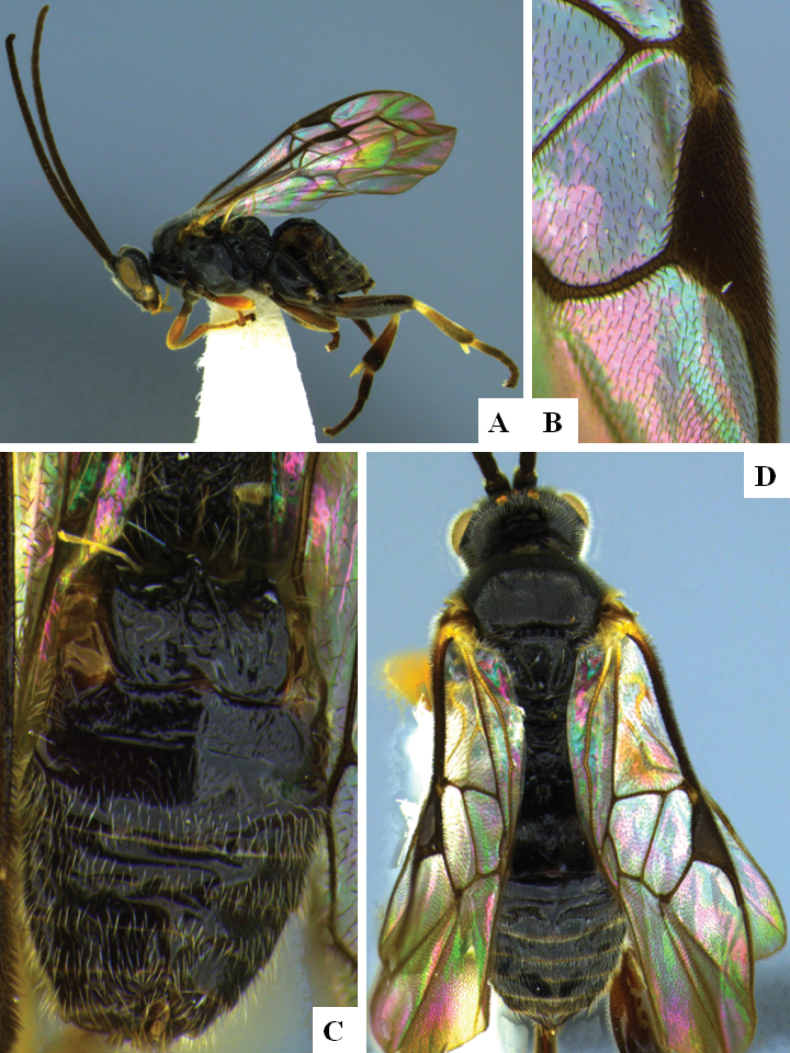
*Alphomelonmelanoscelis* Deans holotype female **A** habitus, lateral **B** fore wing pterostigma **C** metasoma, dorsal **D** habitus, dorsal.

In addition to the holotype (from Costa Rica, Heredia, which we examined, and it is illustrated here in Figs 49A–D) and the paratypes from Costa Rica, Brazil, Mexico and Venezuela mentioned in the original description of the species by Deans et al. (2003) (of those paratypes we could only examine a Brazilian specimen deposited in the CNC with voucher code CNCHYM 00036 and a sequence 164 bp long), we provided here a restricted concept of *melanoscelis*. The following specimens (not included in the original description) are associated with the species: a) additional specimens from Brazil (CNCHYM 00035, CNC2805178, and CNC704367); b) a new country record from Argentina which is based on a sequence in BOLD (specimen BIOUG24734-D06 with 585 bp); c) at least four ACG specimens (currently in BOLD as *A.guillermopereirai*): DHJPAR0026277, DHJPAR0027666, DHJPAR0047176, and DHJPAR0049083; d) ACG specimen DHJPAR0020621, which had been associated to *A.andydeansi* based on sequence matching, is actually *A.melanoscelis*, based on morphological examination (this could represent an accidental labeling mistake or lab contamination). Other ACG specimens that in the future could be transferred to *A.melanoscelis* (based on molecular data) are DHJPAR0058243, DHJPAR0058276, and DHJPAR0056870, but we have not been able to study those specimens and thus cannot conclude on its status at present.

Specimens from Belize, which were included in the original description of *A.melanoscelis* not as paratypes but just as “other material examined” (Deans et al. 2003: 25) are now considered to represent a separate species, *A.rigoi*. One of those Belize specimens rendered an almost complete barcode (573 bp), which allowed us to associate with this species additional specimens in the CNC from Venezuela (with voucher code WMIC 0349, not far from the locality of the Venezuelan paratype of *A.melanoscelis*) and Colombia (see Notes under *A.rigoi* below).

**Figure 50.**
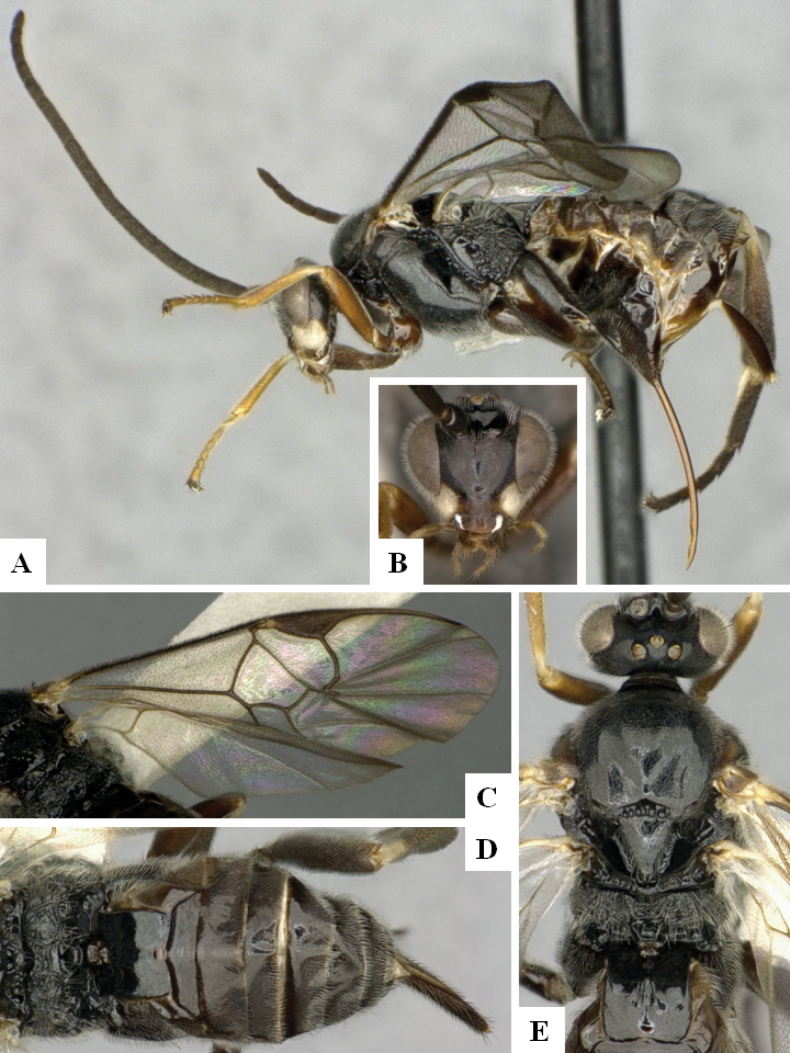
*Alphomelonmelanoscelis* Deans paratype female CNCHYM 00036 **A** habitus, lateral **B** head, frontal **C** wings **D** propodeum and metasoma, dorsal **E** mesosoma, dorsal.

A Mexican specimen in BOLD (in the same BIN and with voucher code 07TAPACH-01773) shows a reddish-brown metafemur and may represent a different species, not considered in this paper because we could not access that specimen.

#### ﻿ Alphomelon mikesharkeyi

Taxon classificationAnimaliaHymenopteraBraconidae

Fernandez-Triana & Shimbori sp. nov.

5A92F5EF-DE33-56BF-8553-4FD76006F793

https://zoobank.org/FEA1C5E8-A3EB-4667-9107-E7D78CBCC80A

Figs 51A–F , 100A

##### Type material.

***Holotype*.** Costa Rica • Female, CNC; Guanacaste, Area de Conservación Guanacaste, Sector Pitilla, Medrano, 11°00'57.67"N, 85°22'49.91"W, 380m; 10.III.2012; ex. *Parphorusdecora*; coll. Minor Carmona; Voucher code: DHJPAR0049235; Host voucher code: 12-SRNP-65197.

***Paratypes*.** Costa Rica • 6 females, 1 male, CNC; CNC308750, CNC308751 (additional specimens in a gel capsule associated with that specimen), DHJPAR0038114, CNC958833, CNC958834, CNC958835 (additional specimens in a gel capsule associated with that specimen), DHJPAR0002292 (additional specimens in a gel capsule associated with that specimen).

**Figure 51.**
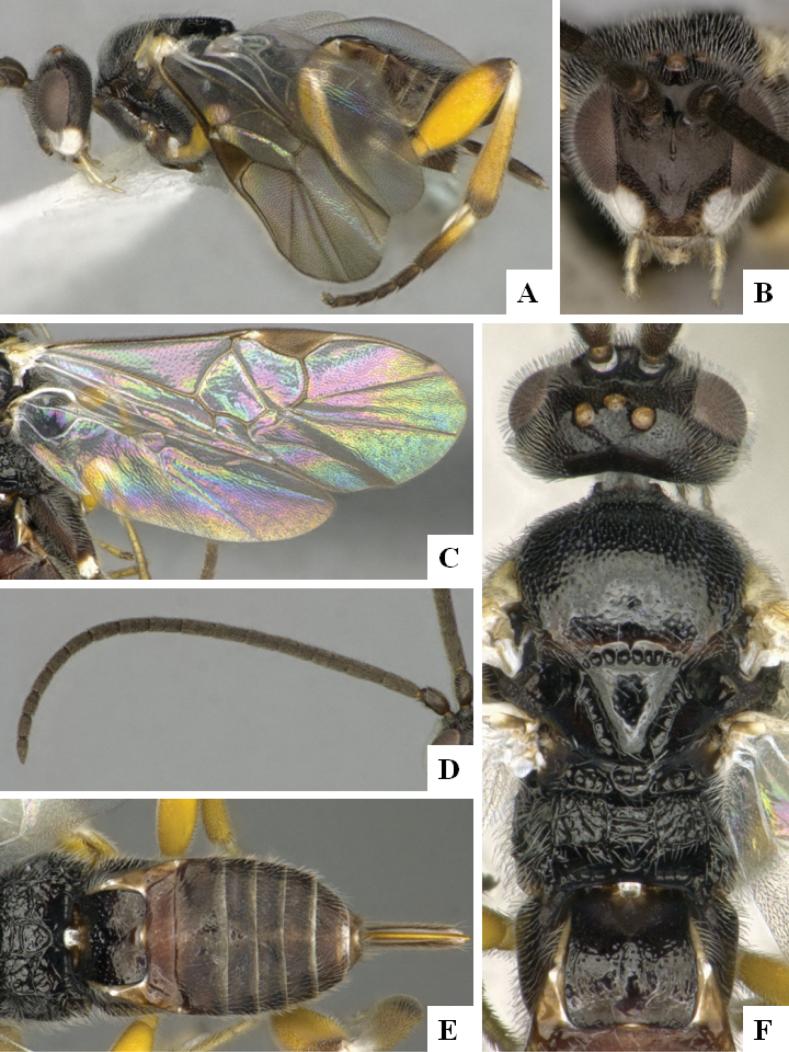
*Alphomelonmikesharkeyi* Fernandez-Triana & Shimbori holotype female DHJPAR0049235 **A** habitus, lateral **B** head, frontal **C** wings **D** antenna, lateral **E** propodeum and metasoma, dorsal **F** head and mesosoma, dorsal.

##### Distribution.

Costa Rica (ACG).

##### Biology.

Gregarious, reared from *Parphorusdecora*, *Quasimellanaservilius*, and *Quasimellana* Burns01.

##### DNA barcoding.

BINBOLD:AAJ2210.

##### Etymology.

Named in honor of Dr. Michael (Mike) Joseph Sharkey, for his intense interest in conducting the taxonomy of ACG and Costa Rican Braconidae making use of DNA barcode information for identifications.

##### Diagnostic description.

White patch on gena: extending to occiput and onto clypeus. Tegula/humeral complex color: white/yellow or. Mesonotum color: mostly dark brown to black. Metasoma color: mostly black or dark brown. Tarsal claws spines: 2 or 3. Pterostigma shape: comparatively less elongate, its length ≤ 2.5× its central height and usually more rounded with at least one of its lower margins curved. T1 sculpture: weakly sculptured along margins. T1 central ridge: marked by weak carina. T2 sculpture: entirely to mostly smooth. Ovipositor sheaths length: shorter than first segment of metatarsus/longer than first segment of metatarsus. Body length: 3.3–3.6 mm. Fore wing length: 3.4–3.5 mm.

#### ﻿ Alphomelon nanosoma

Taxon classificationAnimaliaHymenopteraBraconidae

Deans, 2003

5C08B086-8F16-5D39-A60F-D428240924B3

**Figure 52.**
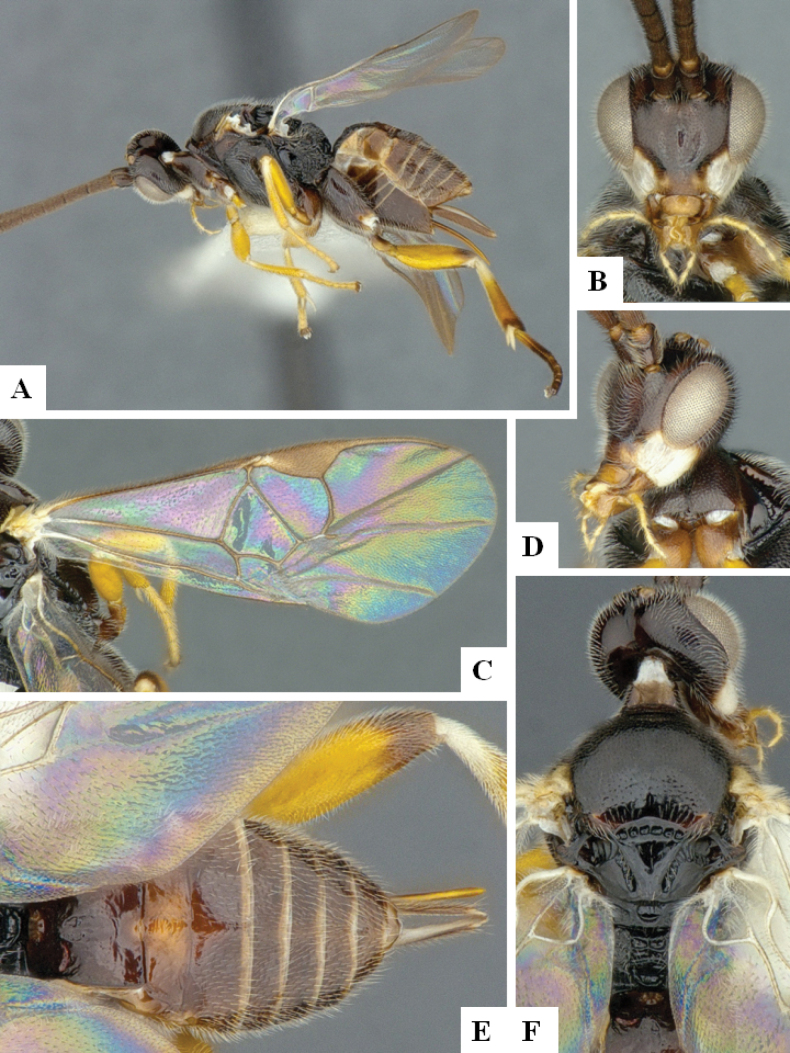
*Alphomelonnanosoma* Deans female CNC704374 **A** habitus, lateral **B** head, frontal **C** fore wing **D** head, fronto-lateral **E** metasoma, dorsal **F** mesosoma, dorsal.

**Figure 53.**
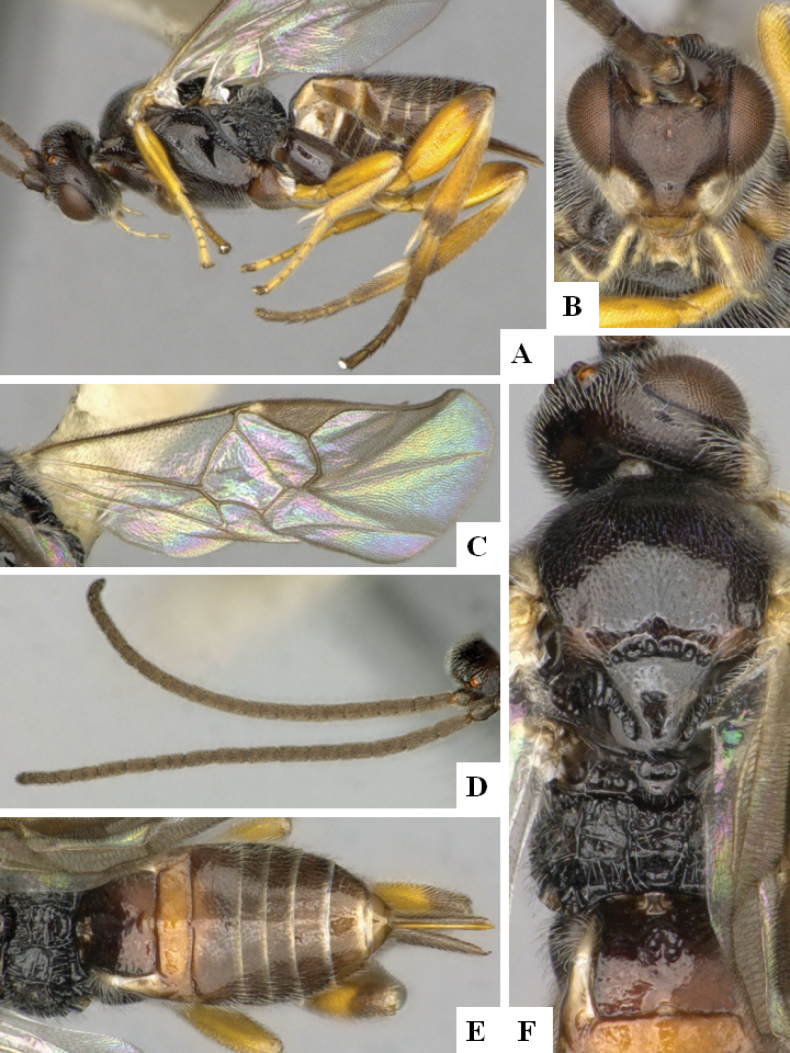
*Alphomelonnanosoma* Deans female CNC704379 **A** habitus, lateral **B** head, frontal **C** fore wing **D** antennae, dorso-lateral **E** propodeum and metasoma, dorsal **F** mesosoma, lateral.

**Figure 54.**
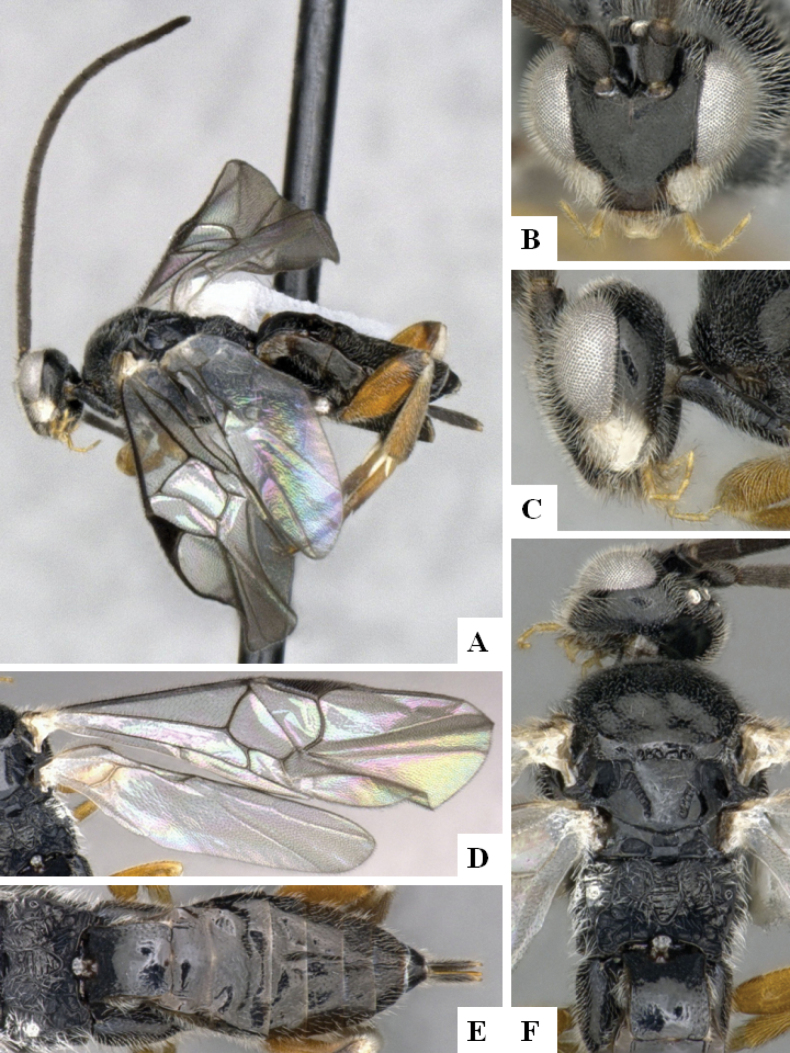
*Alphomelonnanosoma* Deans female CNC1802093 **A** habitus, lateral **B** head, frontal **C** head, lateral **D** wings **E** propodeum and metasoma, dorsal **F** mesosoma, dorsal.

**Figure 55.**
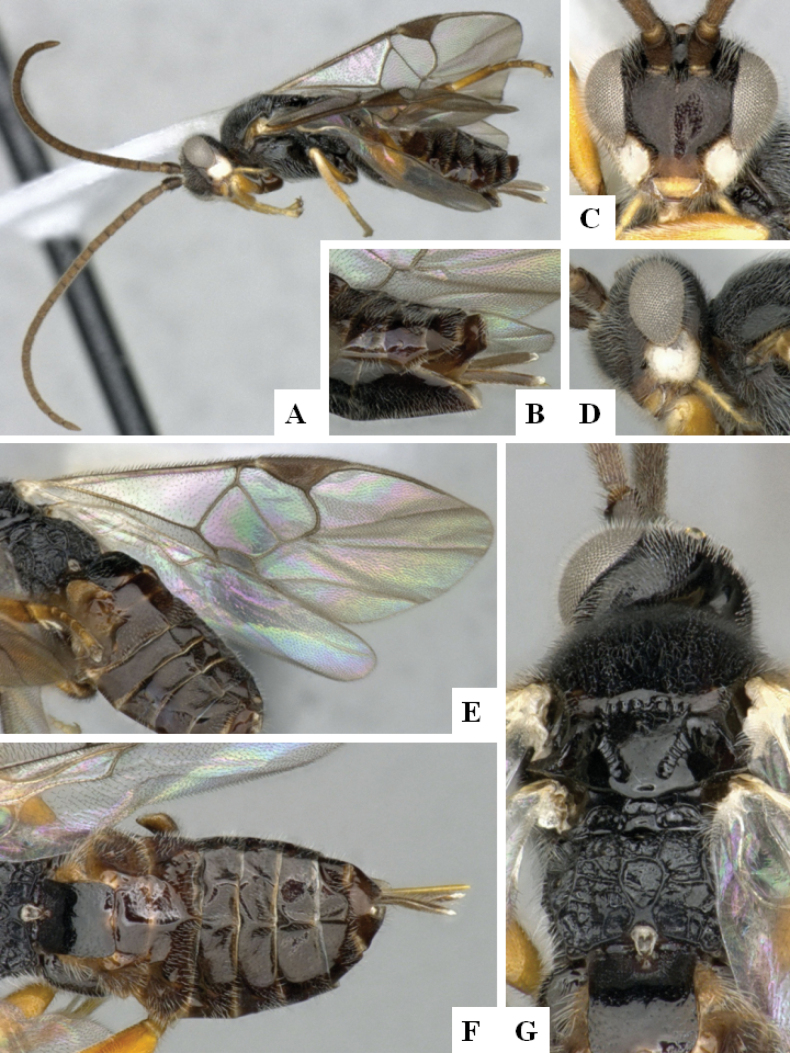
*Alphomelonnanosoma* Deans female DHJPAR0052916 **A** habitus, lateral **B** ovipositor, lateral **C** head, frontal **D** head, lateral **E** wings **F** metasoma, dorsal **G** mesosoma, dorsal.

**Figure 56.**
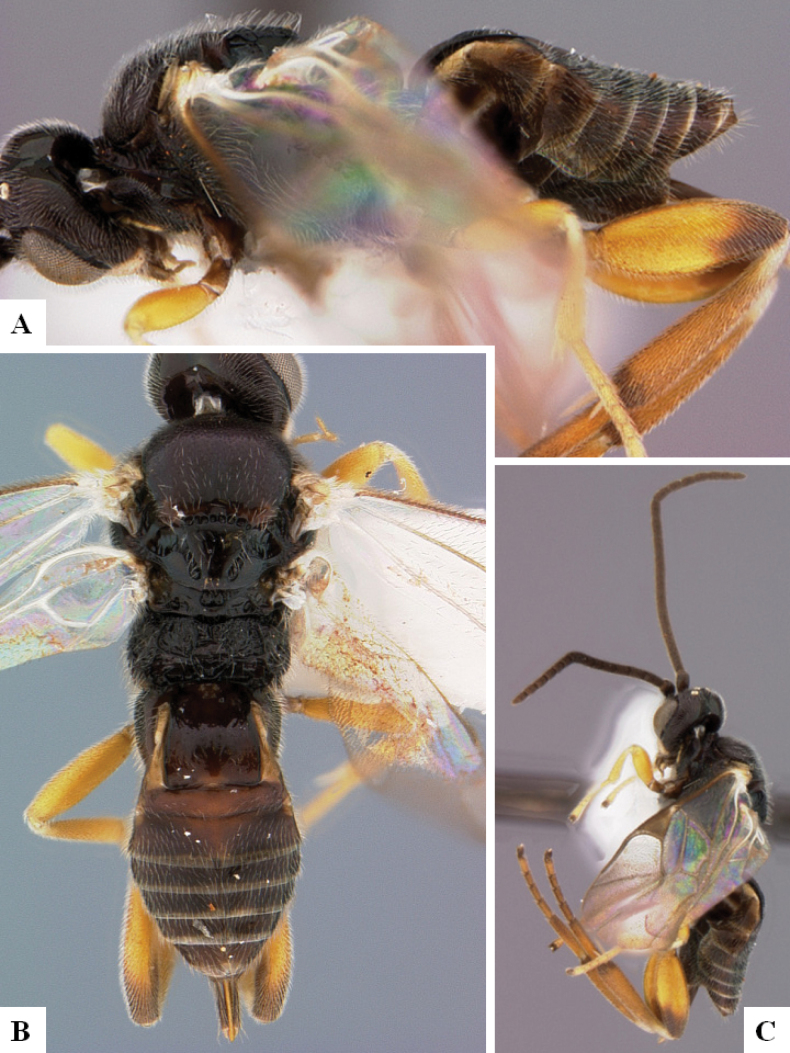
*Alphomelonnanosoma* Deans holotype female USNMENT00828300 **A** close-up of habitus, lateral **B** habitus, dorsal **C** habitus, lateral.

Figs 52A–F , 53A–F , 54A–F , 55A–G , 56A–C , 100B

##### Distribution.

Brazil (MT), Costa Rica (ACG), Ecuador, Mexico, Panama, Trinidad & Tobago.

##### Biology.

Gregarious, reared from *Carystoidesbasoches*, *C.escalantei*, *C.hondura*, *C.orbius*, *Carystoides* Burns01, *Carystoides* escalanteiDHJ02 on at least 10 palm species (Arecaceae). A previous host record, *Cobalopsis* sp. on *Oryzalatifolia* (Deans et al. 2003) could not be confirmed.

##### DNA barcoding.

BINBOLD:AAB9792.

##### Other specimens examined.

(83 females, 17 males, 1 sex unknown): CNC1802049, CNC1802050, CNC1802051, CNC1802052, CNC1802053, CNC1802054, CNC1802055, CNC1802056, CNC1802057, CNC1802058, CNC1802059, CNC1802060, CNC1802061, CNC1802062 (additional specimens in a gel capsule associated with that specimen), CNC1802063, CNC1802064, CNC1802065, DHJPAR0052916, CNC1802066, CNC1802067, CNC1802068, CNC1802069, CNC1802070, CNC1802071, CNC1802072, CNC1802073, CNC1802074, CNC1802075, CNC1802076, CNC1802077, CNC1802078, CNC1802079, DHJPAR0051812, CNC1802080, CNC1802081, CNC1802082, CNC1802083, CNC1802084, CNC1802085 (additional specimens in a gel capsule associated with that specimen), DHJPAR0054602, CNC1802086, CNC1802087, CNC1802088, CNC1802089, CNC1802090, CNC1802091, CNC1802092, CNC1802093, CNC1802094, CNC1802095, CNC1802096, DHJPAR0054600, CNC1802097, CNC1802098, CNC1802099, CNC1802100, CNC1802101 (additional specimens in a gel capsule associated with that specimen), CNC280519, CNCHYM 00042, CNCHYM 00038, CNCHYM 00039, CNCHYM 00040, CNCHYM 00041, CNC704368, CNC704369, CNC704370, CNC704371, CNC704372, CNC704373, CNC704374, CNC704375, CNC704376, CNC704377, CNC704378, CNC704379, CNC704380, CNC704381, CNC704382, CNC704383, CNC704384, CNC704385, CNC704386, CNC704387, CNC721027, CNC721028, CNC721029, CNC721030, CNC721031, CNC721032, CNC721033, CNC721034, CNC721035, CNC721036, CNC721037, CNC721038, CNC721039, CNC721040, CNC721041, CNC721042, CNC721043, CNC721044.

##### Notes.

Based on the material studied, *A.nanosoma* could include a complex of species (all comparatively of smaller size than most *Alphomelon* species). Pending the availability of more DNA barcodes from more specimens, especially from South America, we keep for now all known specimens within the same species.

#### ﻿ Alphomelon nigriceps

Taxon classificationAnimaliaHymenopteraBraconidae

(Ashmead, 1900)

927AD5F6-DFC9-56FB-9E11-ADC0F7EC13C4

Fig. 57A–F

##### Other specimens examined.

(4 females, 2 sex unknown): CNC721046, CNCHYM 00046, CNCHYM 00044, CNCHYM 00043, CNC721045, CNC734964.

##### Distribution.

Argentina, Belize, Brazil (RO), Colombia, Cuba, Curacao*, Dominica, Grenada, Netherlands Antilles, Peru, Saint Lucia, Saint Vincent, Trinidad & Tobago, Venezuela.

##### Biology.

Solitary, reared from *Calpodesethlius* and unidentified hesperiid on corn *Zeamays* (Deans et al. 2003).

##### DNA barcoding.

Two partial barcodes (234 and 458 bp).

##### Notes.

Based on CNC specimens we have studied, the Southern American specimens are darker than the ones from Central America and could represent a different species. In fact, and based on the sequences available to us, one Caribbean specimen (voucher code CNCHYM 00044, from Curacao, and with a partial barcode of 458 bp) is different from a South American specimen (voucher code CNCHYM 00045, from Argentina and with partial barcode of 573 bp). That information seems to indicate that at least two (and possibly three) different species are currently mixed within the name *A.nigriceps*; however, in this paper we prefer to keep all specimens as one species until additional material and sequences become available for study.

**Figure 57.**
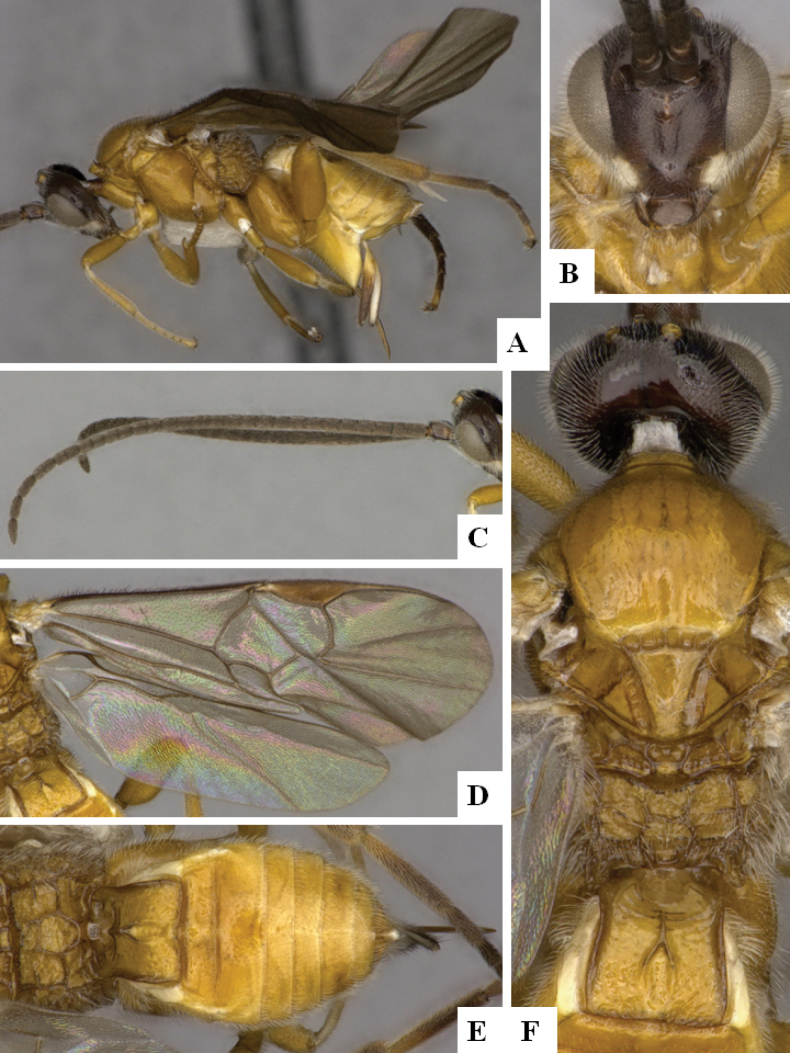
*Alphomelonnigriceps* (Ashmead) female CNC721046 **A** habitus, lateral **B** head, frontal **C** antennae, lateral **D** wings **E** propodeum and metasoma, dorsal **F** mesosoma, dorsal.

#### ﻿ Alphomelon osvaldoespinozai

Taxon classificationAnimaliaHymenopteraBraconidae

Fernandez-Triana & Shimbori sp. nov.

80288B33-2FCA-5659-AE3A-1AF00100D211

https://zoobank.org/6B41A1F1-B010-4CD7-B9DF-5C42D6469BCA

Figs 58A–E , 101A

##### Type material.

***Holotype*.** Costa Rica • Female, CNC; Alajuela, Area de Conservación Guanacaste, Sector Rincon Rain Forest, Garzasol, 10°53'47.98"N, 85°17'24.11"W, 400m; 21.III.2007; ex. *Vettiuspica*; coll. Jose Perez; Voucher code: DHJPAR0030952; Host voucher code: 07-SRNP-40735.

***Paratypes*.** Costa Rica • 3 females, 2 males, CNC; CNC1179920 (additional specimens in a gel capsule associated with that specimen), DHJPAR0030884, DHJPAR0031005, DHJPAR0038181, CNC1180004.

##### Distribution.

Costa Rica (ACG).

##### Biology.

Solitary, reared from *Enosisimmaculata*, *Eutychideochus*, *Oxynthescorusca*, *Parphorusdecora*, *Psoralis* Janzen38, *Vettiuspica*, and an unidentified hesperiid with provisional name “hespJanzen01 Janzen60”.

##### DNA barcoding.

BINBOLD:AAJ2207.

##### Etymology.

Named in honor of Sr. Osvaldo Espinoza in honor of his decades of teamwork in the ACG parataxonomist team.

##### Diagnostic description.

White patch on gena: extending to occiput and onto clypeus. Tegula/humeral complex color: white/yellow. Mesonotum color: mostly dark brown to black. Metasoma color: mostly black or dark brown. Tarsal claws spines: 3. Pterostigma shape: comparatively more elongate, its length ≥ 3.0× its central height and more triangular with its two lower margins more or less straight. T1 sculpture: entirely to mostly smooth. T1 central ridge: marked by weak carina. T2 sculpture: entirely to mostly smooth. Ovipositor sheaths length: longer than first segment of metatarsus. Body length: 3.8–4.7 mm. Fore wing length: 3.9–4.6 mm.

**Figure 58.**
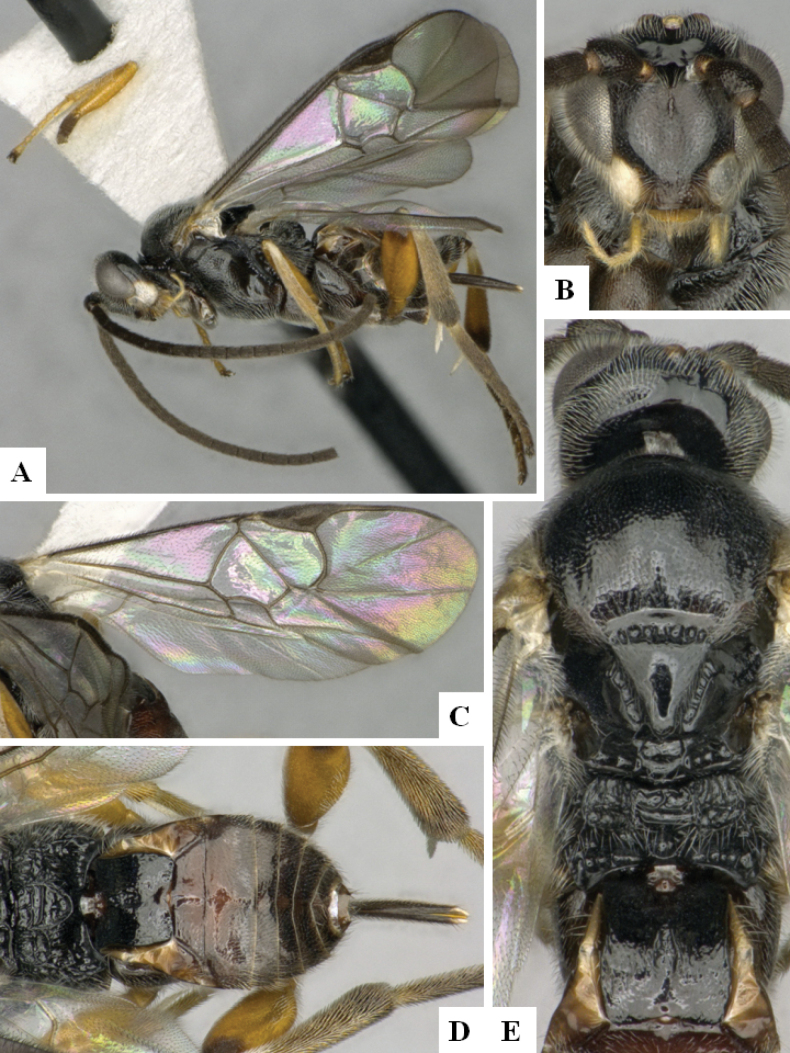
*Alphomelonosvaldoespinozai* Fernandez-Triana & Shimbori holotype female DHJPAR0030952 **A** habitus, lateral **B** head, frontal **C** wings **D** propodeum and metasoma, dorsal **E** mesosoma, dorsal.

#### ﻿ Alphomelon palomae

Taxon classificationAnimaliaHymenopteraBraconidae

Shimbori & Fernandez-Triana sp. nov.

3AF14BA1-B344-54AB-96D6-BB6DDFE496AD

https://zoobank.org/7F77AE85-5C18-47EA-88DC-6FE0EAA205B3

Fig. 59A–F

##### Type material.

***Holotype*.** Brazil • Female “Teresópolis, RJ, Brasil / PARANA da Serra dos Órgãos 22°28'36"S 42°59'31"W Armadilha Malaise 04a (563 m) X.2015 / R.F. Monteiro e eq. col.” (DCBU 467753)

***Paratypes*.** Brazil. 3 females, same as holotype except: 1F “22°28'37"S 42°59'45"W / Malaise 5b (691m) / I.2015” (DCBU 370748); 1F “22°29'40"S 42°59'54"W / Armadilha Malaise 3a (360m) / XI.2014” (DCBU 356385); 1F “22°31'02"S 43°00'22"W Armadilha Malaise 02b (248m) VII.2015” (DCBU 375530). 2 females “Iguape, SP, Brasil / ESEC Juréia - Itatins / 24°31'12"S 47°12'5,8"W / Armadilha Malaise 4 / 19.v.2010 / N.W. Perioto e eq. cols” (DCBU 49960, 49963).

##### Distribution.

Brazil.

##### Biology.

No data.

##### DNA barcoding.

Not available.

##### Etymology.

Named in honor of our colleague Dra. Paloma Helena Fernandes Shimabukuro, for her contribution to the taxonomy of the Brazilian species of *Alphomelon*.

##### Diagnostic description.

White patch on gena: extending to occiput and onto clypeus. Tegula/humeral complex color: yellow/yellow. Mesonotum color: mostly dark brown to black. Metasoma color: mostly black or dark brown. Tarsal claws spines: 1. Pterostigma shape: comparatively less elongate, its length ≤ 2.5× its central height and usually more rounded with at least one of its lower margins curved. T1 sculpture: entirely smooth. T1 central ridge: absent or faintly indicated by shallow depression. T2 sculpture: entirely to mostly smooth. Ovipositor sheaths length: longer than first segment of metatarsus. Body length: 3.7–4.0 mm. Fore wing length: 3.7–4.0 mm.

**Figure 59.**
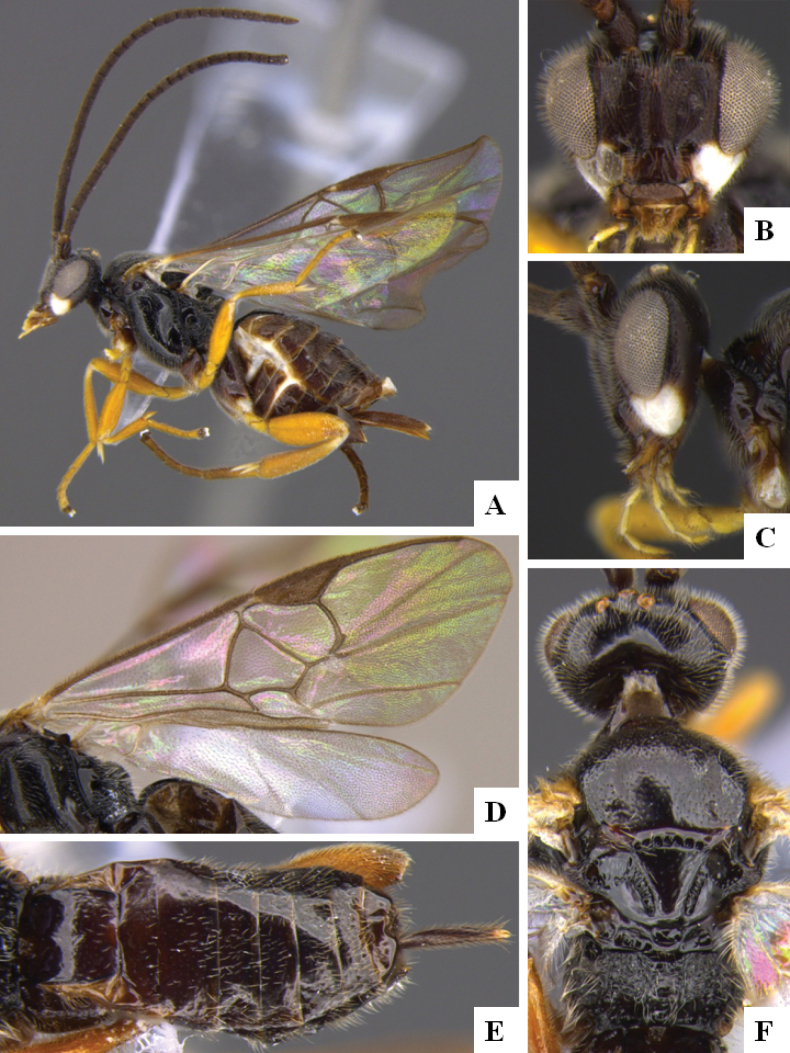
*Alphomelonpalomae* Shimbori & Fernandez-Triana holotype female DCBU 467753 **A** habitus, lateral **B** head, frontal **C** head, lateral **D** wings **E** metasoma, dorsal **F** mesosoma, dorsal.

#### ﻿ Alphomelon paramelanoscelis

Taxon classificationAnimaliaHymenopteraBraconidae

Fernandez-Triana & Shimbori sp. nov.

2FFE4218-61EE-5BA6-818E-C2F065626B28

https://zoobank.org/5B3781B1-6F4B-4A52-B0FB-76FEE47E62B4

**Figure 60.**
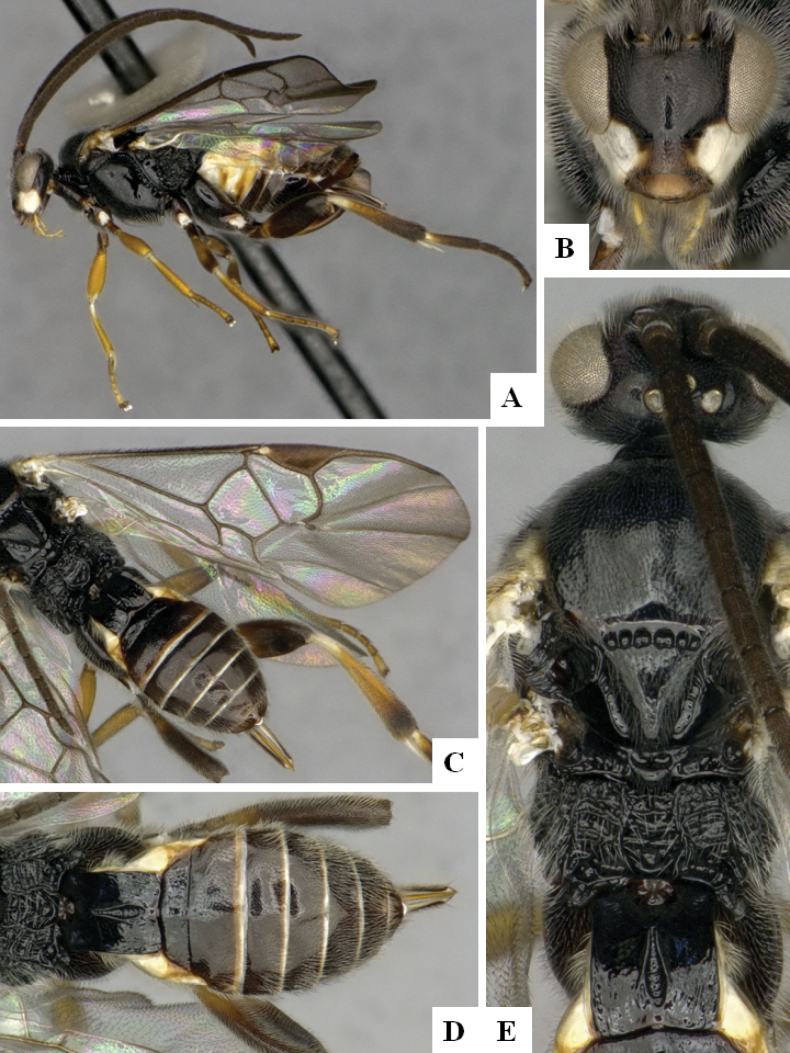
*Alphomelonparamelanoscelis* Fernandez-Triana & Shimbori female CNC704366 **A** habitus, lateral **B** head, frontal **C** wings **D** propodeum and metasoma, dorsal **E** mesosoma, dorsal.

**Figure 61.**
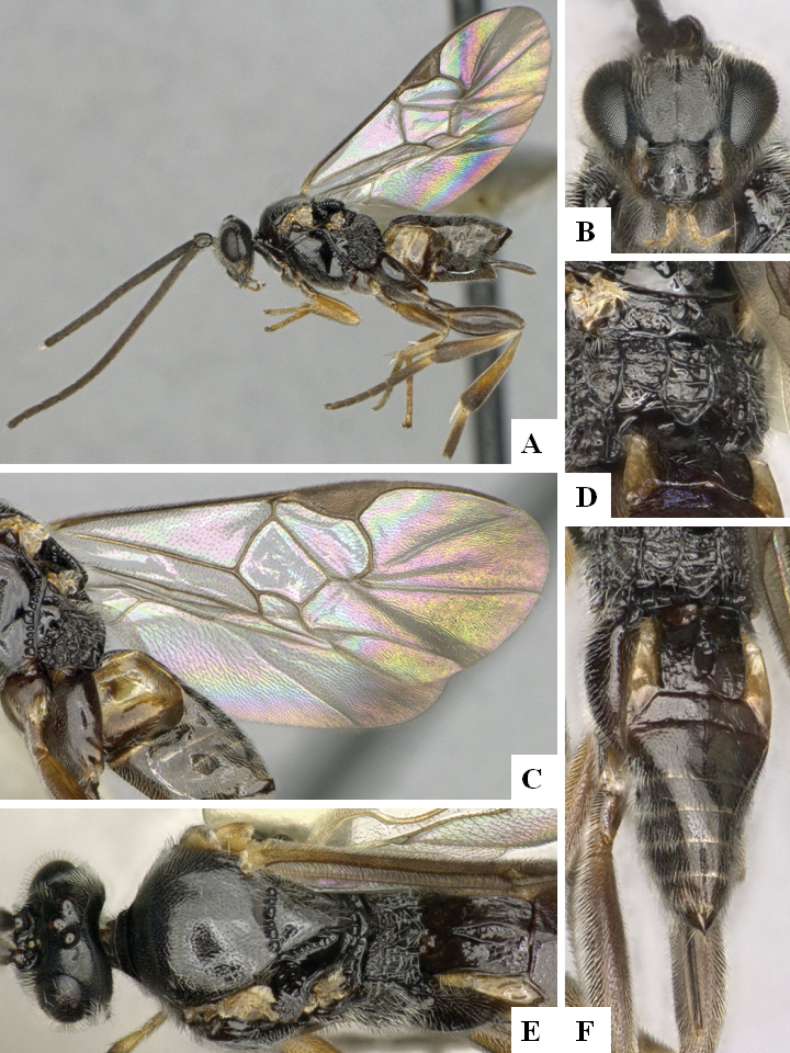
*Alphomelonparamelanoscelis* Fernandez-Triana & Shimbori holotype female CNC704362 **A** habitus, lateral **B** head, frontal **C** wings **D** propodeum, dorsal **E** head and mesosoma, dorsal **F** metasoma, dorsal.

**Figure 62.**
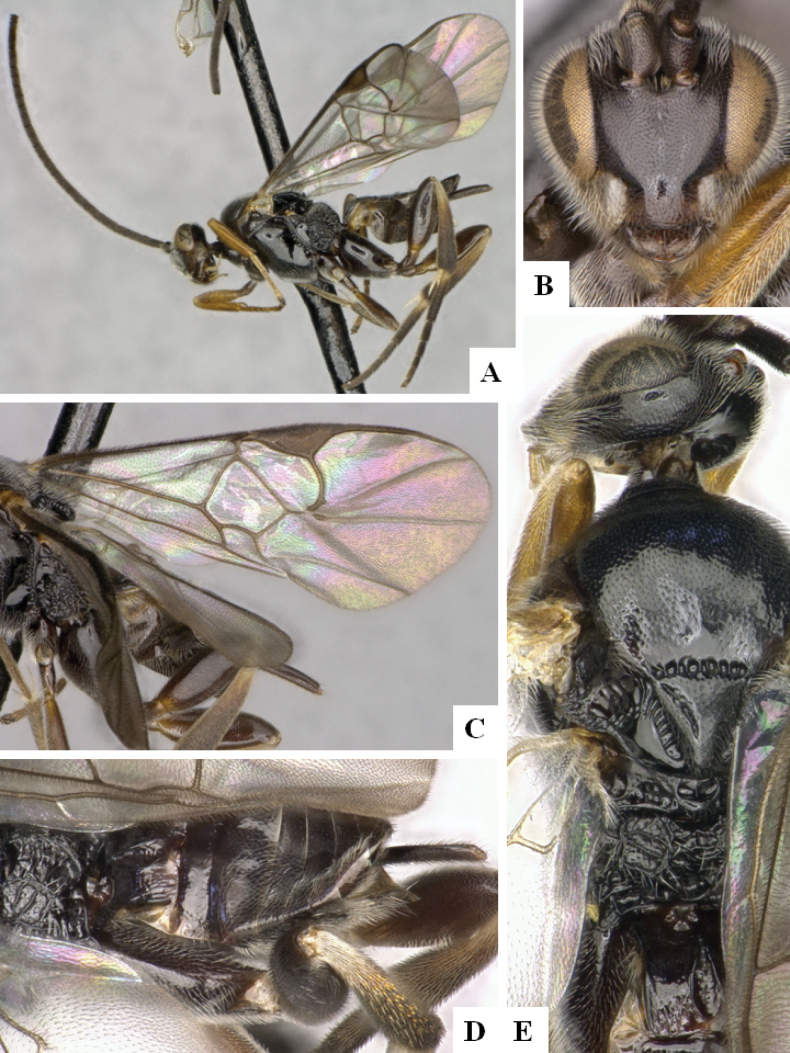
*Alphomelonparamelanoscelis* Fernandez-Triana & Shimbori paratype female CNC704363 **A** habitus, lateral **B** head, frontal **C** fore wing **D** propodeum and metasoma, dorso-lateral **E** mesosoma, dorsal.

**Figure 63.**
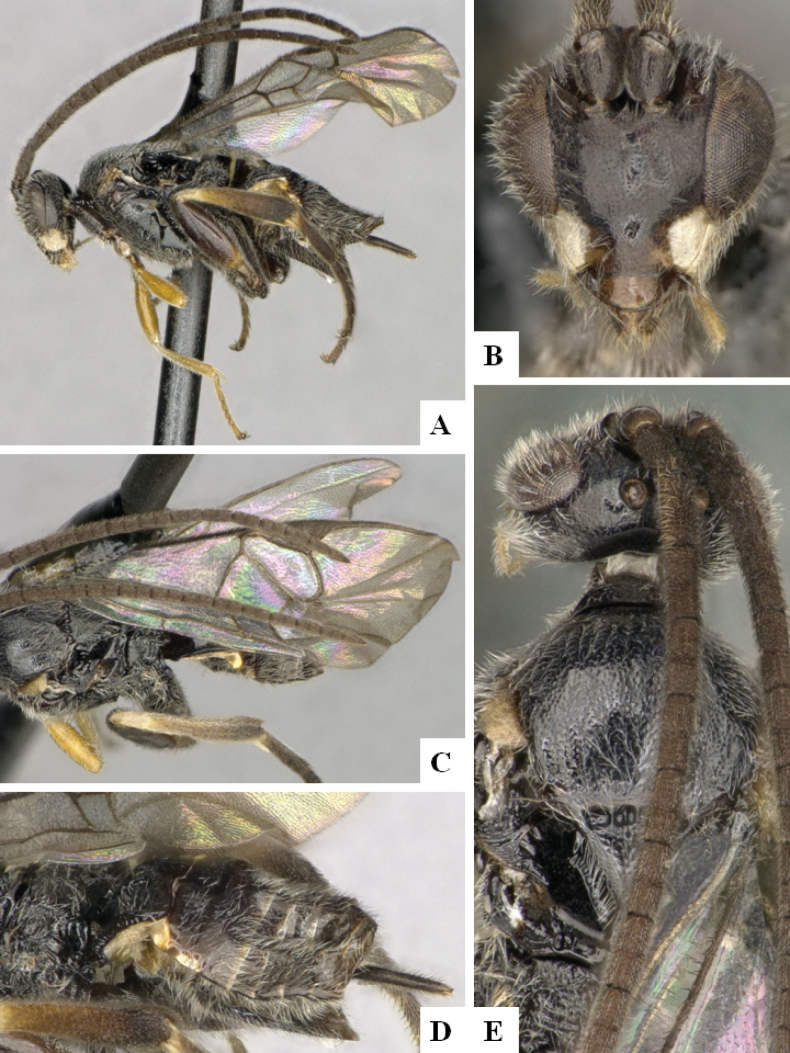
*Alphomelonparamelanoscelis* Fernandez-Triana & Shimbori paratype female CNCHYM 00034 **A** habitus, lateral **B** head, frontal **C** wings **D** propodeum and metasoma, dorso-lateral **E** mesosoma, dorsal.

Figs 60A–E , 61A–F , 62A–E , 63A–E

##### Type material.

***Holotype*.** Brazil • Female, CNC; Santa Catarina, Nova Teutonia, 27°11'0"S, 52°23'0"W, 300–500 m; 9.II.1962; coll. Fritz Plaumann; Voucher code: CNC704362.

***Paratypes*.** Brazil • 3 females, CNC. Voucher codes: CNC704361, CNC704363, CNC704366; COLOMBIA • 1 female, CNC. Voucher code: CNCHYM 00034.

##### Distribution.

Brazil, Colombia.

##### Biology.

No data.

##### DNA barcoding.

No BIN but one partial sequence (442 bp) from Colombia (see further details about problems with the closest BIN associated with this species under the treatment of *Alphomelonmelanoscelis*).

##### Etymology.

Named after *Alphomelonmelanoscelis*, a species it resembles. In fact, some specimens of *A.paramelanoscelis* were considered as part of *A.melanoscelis* by previous authors (Deans et al. 2003).

##### Diagnostic description.

White patch on gena: extending to occiput and onto clypeus. Tegula/humeral complex color: yellow/yellow. Mesonotum color: mostly dark brown to black. Metasoma color: mostly dark brown to black but with some laterotergites and sternites yellow. Tarsal claws spines: 3 or 4. Pterostigma shape: comparatively less elongate, its length ≤ 2.5× its central height and usually more rounded with at least one of its lower margins curved. T1 sculpture: entirely to mostly smooth. T1 central ridge: marked by two raised carinae. T2 sculpture: entirely to mostly smooth. Ovipositor sheaths length: shorter than first segment of metatarsus. Body length: 4.1–4.7 mm. Fore wing length: 4.0–4.7 mm.

##### Notes.

The paratype from southern Colombia (Leticia) shares all characteristics with the Brazilian specimens except that metatibia is dark brown on posterior 0.5 and its is smaller (body size 4.1 mm and fore wing length 4.0 mm).

#### ﻿ Alphomelon paranigriceps

Taxon classificationAnimaliaHymenopteraBraconidae

Fernandez-Triana & Shimbori sp. nov.

E9FAAD48-4339-5CD2-81CC-6577DF885817

https://zoobank.org/536C1A8C-BC86-41E1-B41E-7DD8A6B3E747

Fig. 64A–H

##### Type material.

***Holotype*.** United States • Female, CNC; Georgia, McIntosh county, Sapelo Island, Shrub Sand Dunes; 18.vii–22.viii.1987; Malaise Trap; BRC Hymenoptera Team; Voucher code: CNC280520.

##### Other specimens examined.

United States • 2 females, USNM, Florida (Key Largo); North Carolina (Moore County, Southern Pines); 1 female, TAMU, Texas (Hunt county, Clymer Prairie); unspecified specimen from South Carolina (Deans et al. 2003).

##### Distribution.

United States.

##### Biology.

No data.

##### DNA barcoding.

Not available.

##### Etymology.

Named after *Alphomelonnigriceps*, a species it resembles. Some specimens of *A.paranigriceps* were considered as part of *A.nigriceps* by previous authors (e.g., Deans et al. 2003; Fernandez-Triana et al. 2020).

##### Diagnostic description.

White patch on gena: extending to occiput but not to clypeus. Tegula/humeral complex color: yellow/yellow. Mesonotum color: mostly orange-yellow. Metasoma color: mostly orange-yellow. Tarsal claws spines: 1. Pterostigma shape: comparatively less elongate, its length ≤ 2.5× its central height and usually more rounded with at least one of its lower margins curved. T1 sculpture: strongly sculptured on at least apical half or more. T1 central ridge: strongly marked by two raised carinae. T2 sculpture: entirely to mostly strongly sculptured. Ovipositor sheaths length: as long as first segment of metatarsus. Body length: 5.0 mm. Fore wing length: 5.2 mm.

##### Notes.

We consider as belonging to this species all specimens from the USA (FL, NC, SC, and TX) previously reported by Marsh (1965) and Deans et al. (2003) as *A.nigriceps*. This would agree with the comments made by Deans et al. (2003: 28) about the USA specimens having different (darker) colouration than tropical specimens. Because we could not examine that material we cannot include those specimens as paratypes of the new species, but we assume in this paper that the “true” *A.nigriceps* is limited to the Caribbean islands and South America, whereas *A.paranigriceps* includes all specimens from continental USA.

**Figure 64.**
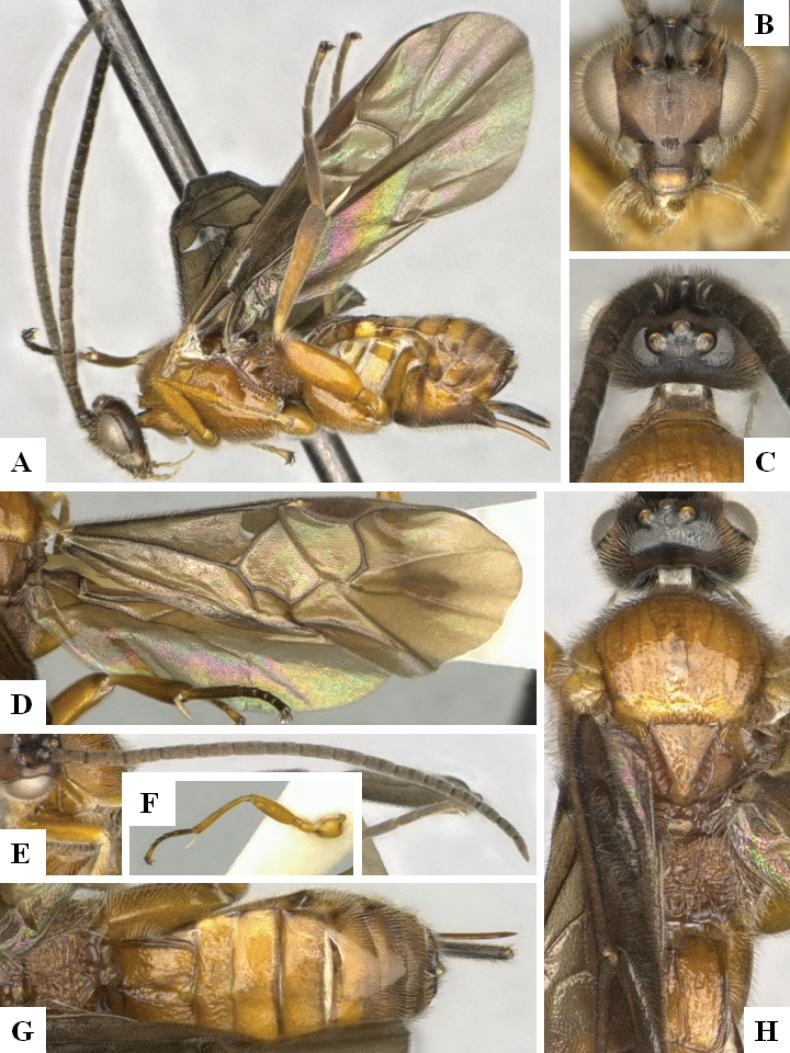
*Alphomelonparanigriceps* Fernandez-Triana & Shimbori holotype female CNC280520 **A** habitus, lateral **B** head, frontal **C** head, dorsal **D** wings **E** antenna, ventral **F** mid leg, lateral **G** propodeum and metasoma, dorsal **H** mesosoma, dorsal.

#### ﻿ Alphomelon paurogenum

Taxon classificationAnimaliaHymenopteraBraconidae

Deans, 2003

59B4BBFB-166C-5F20-85FB-786B4D6CF527

Fig. 65A–I

##### Distribution.

Argentina, Chile.

##### Biology.

No data.

##### DNA barcoding.

Not available.

**Figure 65.**
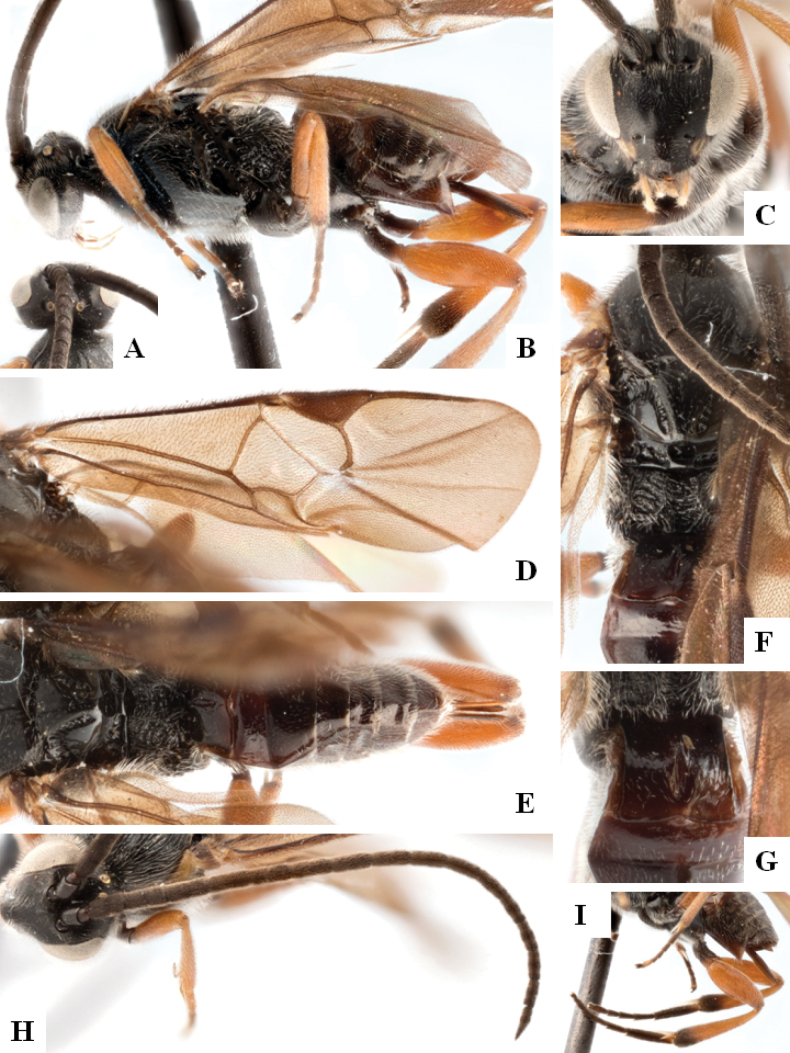
*Alphomelonpaurogenum* Deans paratype female from AEIC**A** head, dorsal **B** habitus, lateral **C** head, frontal **D** fore wing **E** metasoma, dorsal **F** propodeum, dorsal **G** T1, dorsal **H** antenna, ventral **I** Hind legs, lateral.

#### ﻿ Alphomelon petronariosae

Taxon classificationAnimaliaHymenopteraBraconidae

Fernandez-Triana & Shimbori sp. nov.

33AB4AB9-A6C7-507F-AEC0-90103ABF1D9E

https://zoobank.org/D33B0E1A-E82C-44E9-AD68-E080CC2B5091

Figs 66A–E , 101B

##### Type material.

***Holotype*.** Costa Rica • Female, CNC; Guanacaste, Area de Conservación Guanacaste, Sector Pitilla, Pasmompa, 11°01'09.34"N, 85°24'35.89"W, 440m; 17.IX.2011; ex. *Leremaliris*; coll. Freddy Quesada; Voucher code: DHJPAR0048219; Host voucher code: 11-SRNP-32632.

##### Distribution.

Costa Rica (ACG).

##### Biology.

Solitary, reared from *Anthoptusepictetus*, *Congachydaea*, *Leremaliris*, *Moryslyde*, *M.micythus*, *Vehiliusvetula*, and *Vehilius* Janzen03.

##### DNA barcoding.

BIN BOLD:ADA7564

##### Etymology.

Named in honor of Sra. Petrona Ríos in honor of her decades of teamwork in the ACG parataxonomist team.

##### Diagnostic description.

White patch on gena: extending to occiput and onto clypeus. Tegula/humeral complex color: white/yellow. Mesonotum color: mostly dark brown to black. Metasoma color: mostly black or dark brown. Tarsal claws spines: 1. Pterostigma shape: comparatively less elongate, its length ≤ 2.8× its central height and usually more rounded with at least one of its lower margins curved. T1 sculpture: entirely to mostly smooth.

T1 central ridge: clearly marked by two raised carinae. T2 sculpture: entirely to mostly smooth. Ovipositor sheaths length: longer than first segment of metatarsus. Body length: 4.4 mm. Fore wing length: 4.2 mm.

##### Notes.

The barcodes available for this species are comparatively very similar to those of *A.calixtomoragai* (only 1.28% bp different) but the two species have morphological differences (see key) as well as different hosts.

**Figure 66.**
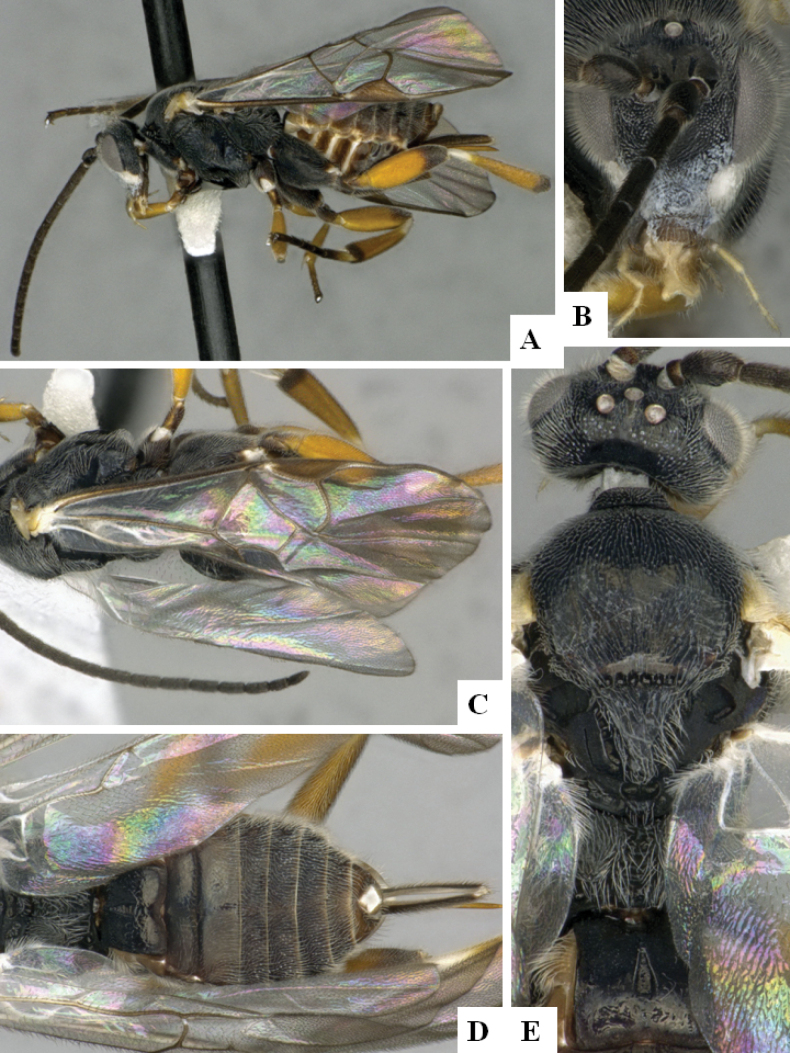
*Alphomelonpetronariosae* Fernandez-Triana & Shimbori holotype female DHJPAR0048219 **A** habitus, lateral **B** head, frontal **C** wings **D** metasoma, dorsal **E** mesosoma, dorsal.

#### ﻿ Alphomelon pyrrhogluteum

Taxon classificationAnimaliaHymenopteraBraconidae

Deans, 2003

6566CC81-E0D7-5C49-B550-5E9B2133858D

Fig. 67A–G

##### Distribution.

Argentina.

##### Biology.

Unknown.

##### DNA barcoding.

Not available.

**Figure 67.**
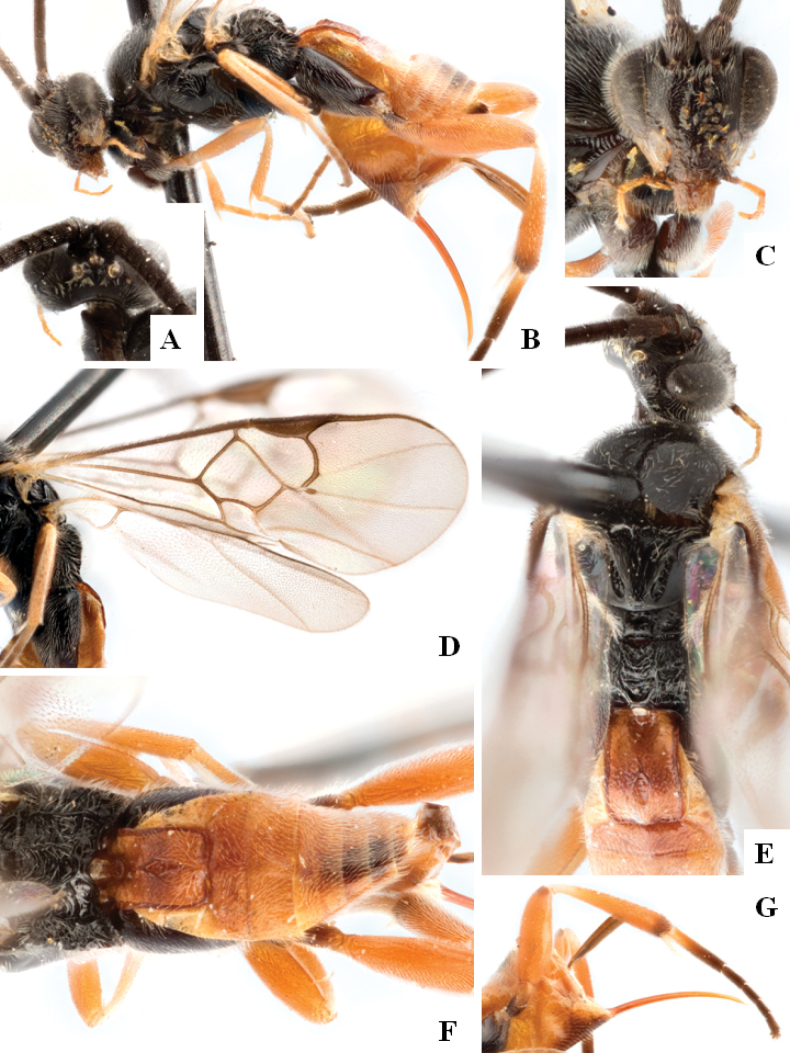
*Alphomelonpyrrhogluteum* Deans paratype female from AEIC**A** head, dorsal **B** habitus, lateral **C** head, frontal **D** wings **E** mesosoma, dorsal **F** metasoma, dorsal **G** hind leg, lateral.

#### ﻿ Alphomelon rhyssocercus

Taxon classificationAnimaliaHymenopteraBraconidae

Deans, 2003

7A1FFAA6-E2EF-566A-96AF-B3F0777A2D17

Figs 68A–G , 69A–E

##### Distribution.

Argentina, Costa Rica, Ecuador, Panama, Peru, Trinidad & Tobago, Venezuela; specimens collected in rainforest sites.

##### Biology.

Unknown.

##### DNA barcoding.

Not available.

##### Other specimens examined.

(9 females, 10 males, 2 sex unknown): CNCHYM 00049, CNC721047, CNC721048, CNC721051, CNC721057, CNC721056, CNC721055, CNC721054, CNC721061, CNC721059, CNC721049, CNC280521, CNC721053, CNC721058, CNC721052, CNCHYM 00047, CNCHYM 00048, CNCHYM 00050, CNC721050, CNC721060, CNC1196961.

**Figure 68.**
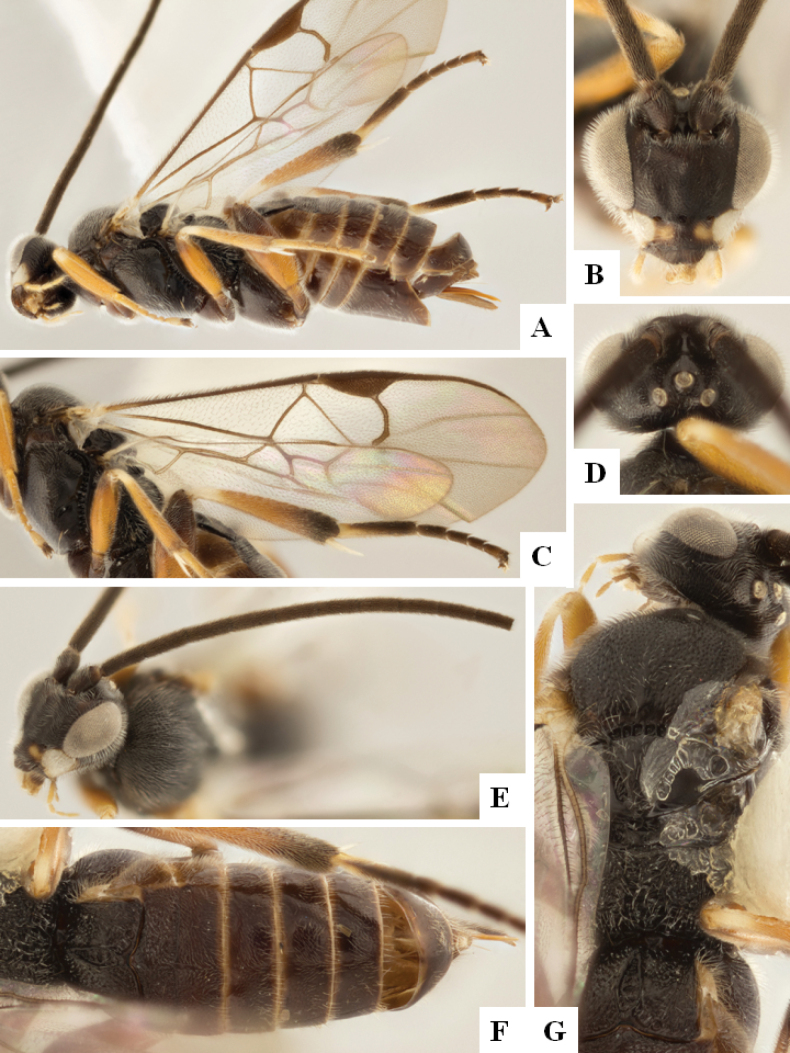
*Alphomelonrhyssocercus* Deans holotype female **A** habitus, lateral **B** head, frontal **C** wings **D** head, dorsal **E** antenna, lateral **F** metasoma, dorsal **G** mesosoma, dorsal.

**Figure 69.**
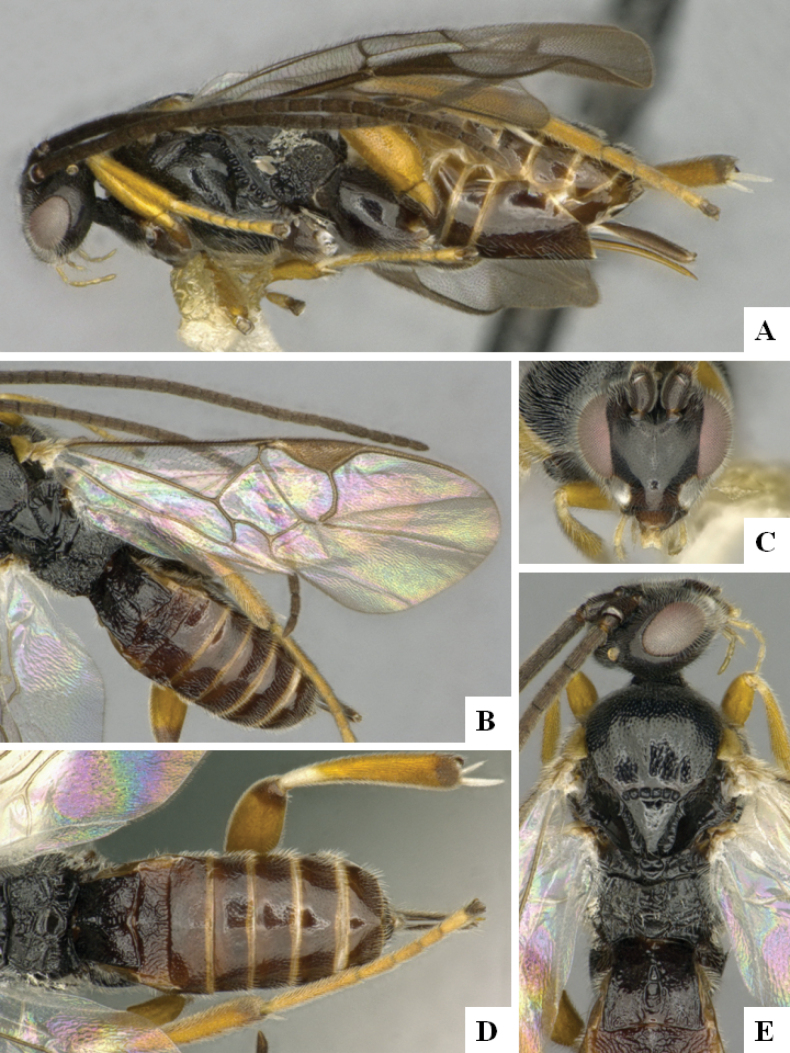
*Alphomelonrhyssocercus* Deans paratype female CNCHYM 00049 **A** habitus, lateral **B** fore wing **C** head, frontal **D** propodeum and metasoma, dorsal **E** mesosoma, dorsal.

#### ﻿ Alphomelon ricardocaleroi

Taxon classificationAnimaliaHymenopteraBraconidae

Fernandez-Triana & Shimbori sp. nov.

C244DD1B-2042-5561-AC19-F21172263A34

https://zoobank.org/3893D222-5444-4C00-B61A-26E806CD1A39

**Figure 70.**
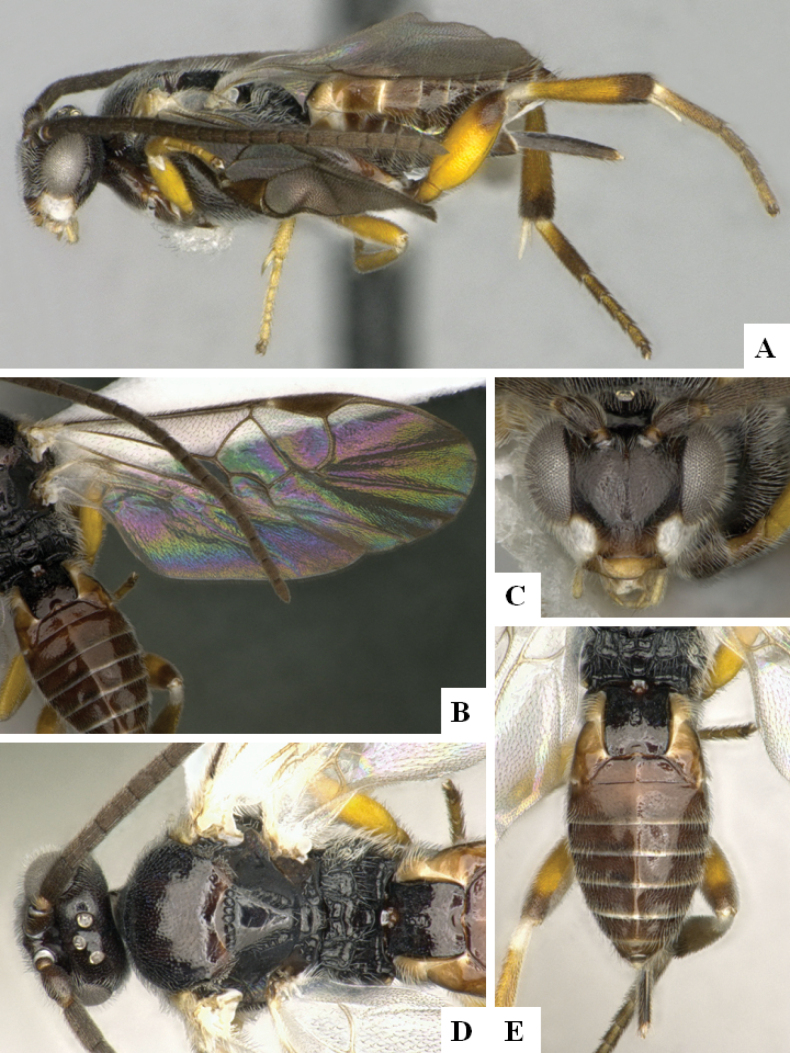
*Alphomelonricardocaleroi* Fernandez-Triana & Shimbori holotype female DHJPAR0020280 **A** habitus, lateral **B** wings **C** head, frontal **D** mesosoma, dorsal **E** metasoma, dorsal.

**Figure 71.**
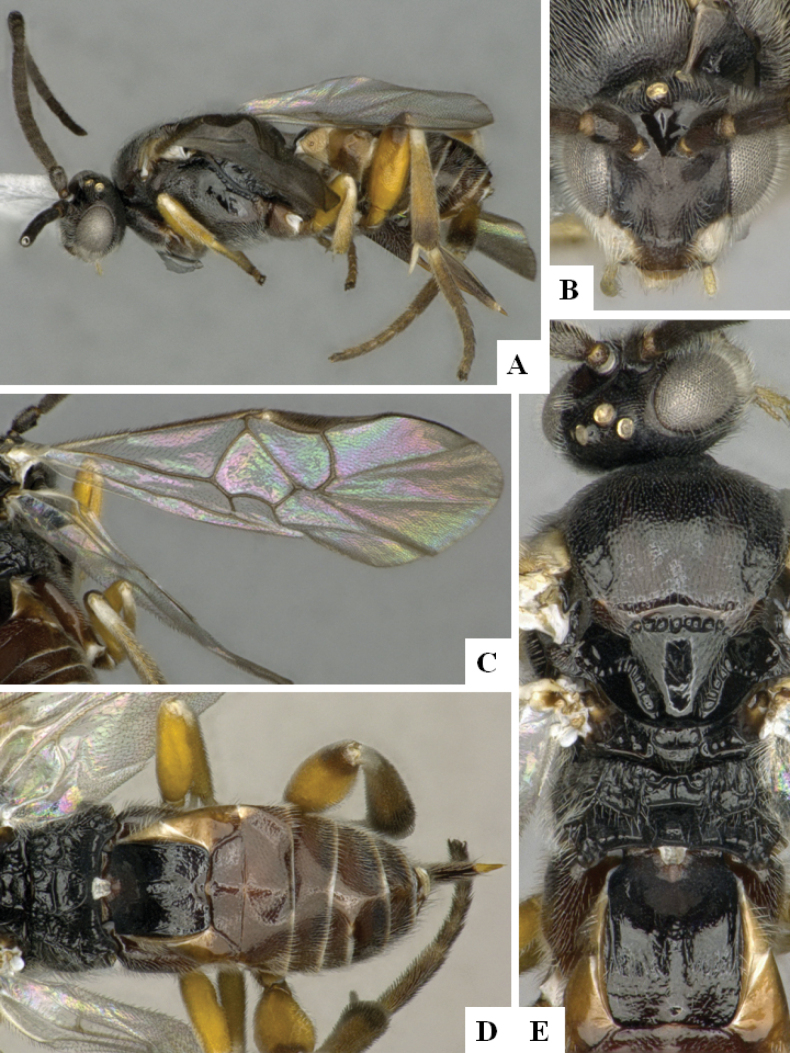
*Alphomelonricardocaleroi* Fernandez-Triana & Shimbori paratype female DHJPAR0040440 **A** habitus, lateral **B** head, frontal **C** fore wing **D** propodeum and metasoma, dorsal **E** mesosoma, dorsal.

Figs 70A–E , 71A–E , 102A

##### Type material.

***Holotype*.** Costa Rica • Female, CNC; Alajuela, Area de Conservación Guanacaste, Sector Rincon Rain Forest, Vochysia, 10°51'59.98"N, 85°14'43.01"W, 320m; 16.VI.2007; ex. *Cynea* sp. Burns06; coll. Jose Perez; Voucher code: DHJPAR0020280; Host voucher code: 07-SRNP-41811.

***Paratypes*.** Costa Rica • 12 females, CNC; CNC1180130, CNC1180125, CNC1180148 (additional specimens in a gel capsule associated with that specimen), DHJPAR0020276, DHJPAR0040440, CNC1180144, CNC1180154, CNC1180169, DHJPAR0054695, CNC1196552, CNC1196559, CNC1196599 (additional specimens in a gel capsule associated with that specimen).

##### Distribution.

Costa Rica (ACG).

##### Biology.

Gregarious, reared from *Cyneacynea*, *C.megalops*, and *C.* Burns06.

##### DNA barcoding.

BINBOLD:AAB7535.

##### Etymology.

Named in honor of Sr. Ricardo Calero in honor of his decades of teamwork in the ACG inventory parataxonomist team.

##### Diagnostic description.

White patch on gena: extending to occiput and onto clypeus. Tegula/humeral complex color: yellow/yellow. Mesonotum color: mostly dark brown to black. Metasoma color: mostly black or dark brown. Tarsal claws spines: 2 or 3. Pterostigma shape: comparatively more elongate, its length ≥ 3.0× its central height and more triangular with its two lower margins more or less straight. T1 sculpture: weakly sculptured along margins. T1 central ridge: marked by weak carina. T2 sculpture: entirely to mostly smooth. Ovipositor sheaths length: longer than first segment of metatarsus. Body length: 3.7–3.9 mm. Fore wing length: 3.4–3.7 mm.

##### Notes.

Some paratype specimens have ovipositor sheaths longer than holotype.

#### ﻿ Alphomelon rigoi

Taxon classificationAnimaliaHymenopteraBraconidae

Fernandez-Triana & Shimbori sp. nov.

DF58017D-E3BB-56C1-8265-5362EC455C79

https://zoobank.org/B505E0F8-ABE3-4C06-A047-9F9AF4C044F1

Figs 72A–E , 73A–E

##### Type material.

***Holotype*.** Belize • Female, CNC; Belize [Label has country as “British Honduras”], Middlesex, 17°2'22.70"N, 88°31'13.30"W, 125m; 12.IV.1965; coll. E. C. Welling; Voucher code: CNCHYM 00037.

***Paratypes*.** Belize • 2 females, CNC. Voucher codes: CNC704364, CNC704365.

##### Other specimens examined.

Venezuela • 1 female, CNC. Voucher codes: WMIC 0349

##### Distribution.

Belize, Venezuela.

##### Biology.

No data.

##### DNA barcoding.

BINBOLD:AAB8584, based on an almost complete sequence (573 bp), but see Notes below.

##### Etymology.

JFT dedicates this species to his dear friend Rigoberto (Rigo) Pérez de Alejo Fortún, in appreciation for the many adventures and experiences they have shared over the years.

##### Diagnostic description.

White patch on gena: extending to occiput and onto clypeus. Tegula/humeral complex color: yellow/brown. Mesonotum color: mostly dark brown to black. Metasoma color: mostly black or dark brown. Tarsal claws spines: 3. Pterostigma shape: comparatively less elongate, its length ≤ 2.5× its central height and usually more rounded with at least one of its lower margins curved. T1 sculpture: strongly sculptured on at least apical half or more. T1 central ridge: strongly marked by two raised carinae and strong costulae centrally. T2 sculpture: strongly sculptured. Ovipositor sheaths length: shorter than first segment of metatarsus. Body length: 4.2–4.4 mm. Fore wing length: 4.2–4.6 mm.

**Figure 72.**
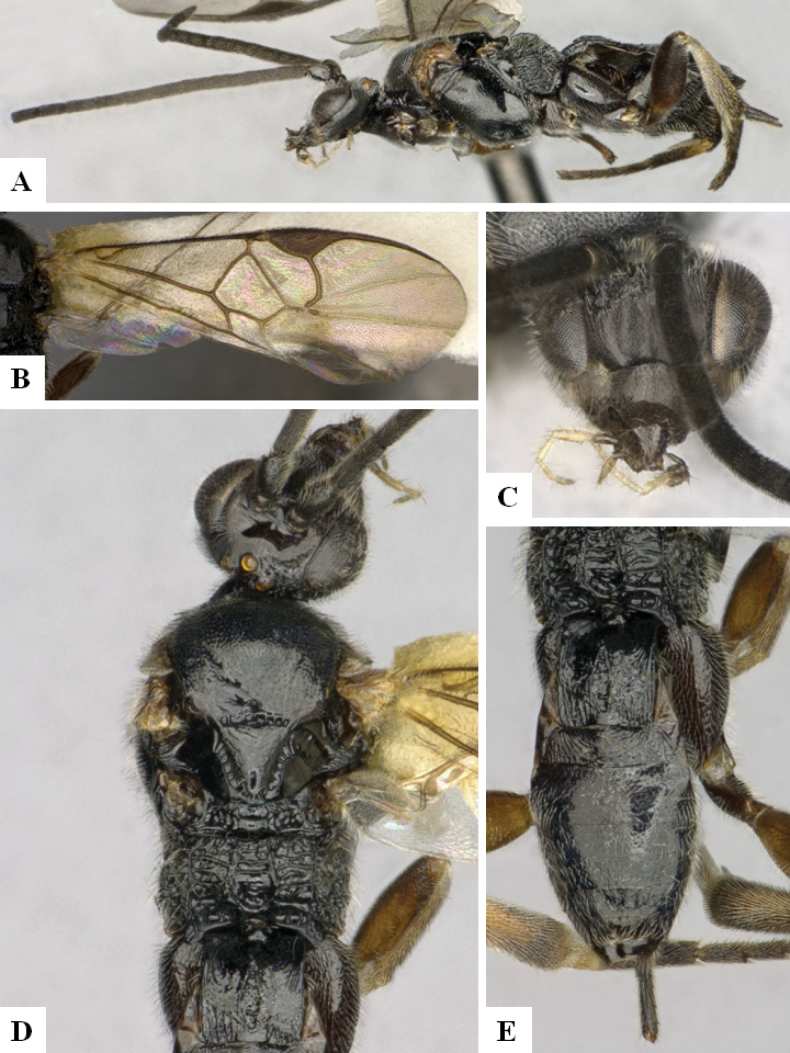
*Alphomelonrigoi* Fernandez-Triana & Shimbori holotype female CNCHYM 00037 **A** habitus, lateral **B** fore wing **C** head, frontal **D** mesosoma, dorsal **E** metasoma, dorsal.

##### Notes.

The associated BIN includes at least three (possibly more) species, see further explanations and details under the treatment of *Alphomelonmelanoscelis.* One female from Venezuela (WMIC 0349) is kept provisionally as this species but is excluded from the type series because it probably represents a different, undescribed species.

**Figure 73.**
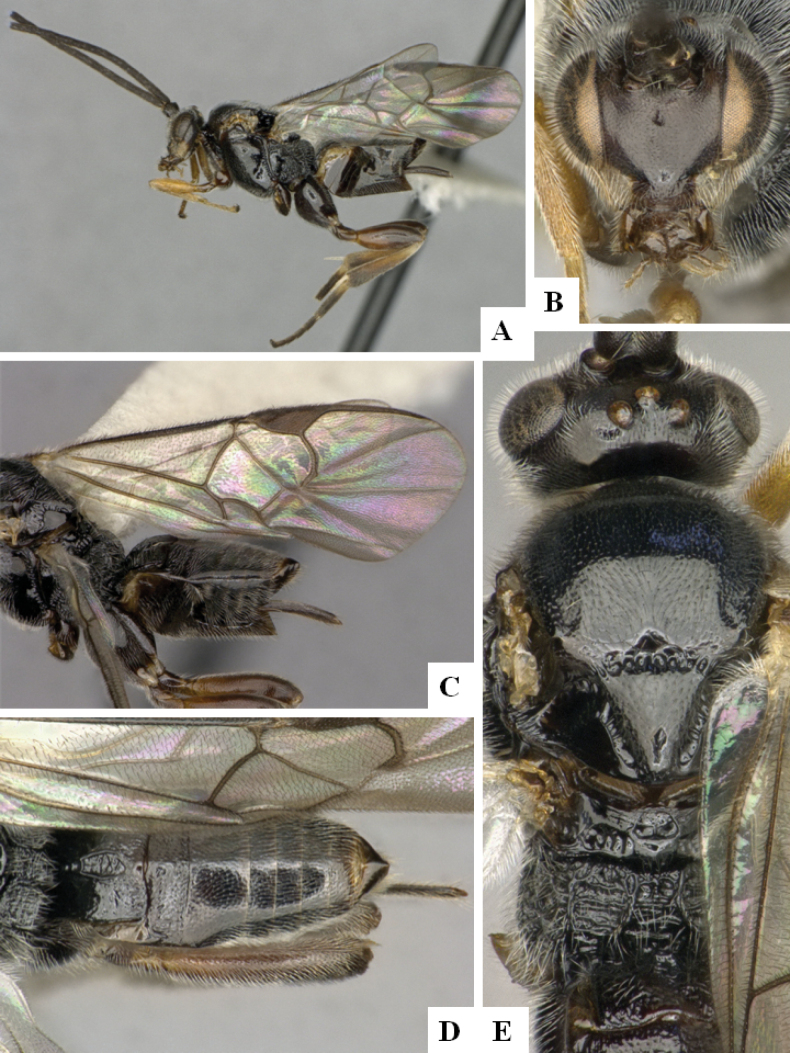
*Alphomelonrigoi* Fernandez-Triana & Shimbori paratype female CNC704365 **A** habitus, lateral **B** head, frontal **C** wings **D** metasoma, dorsal **E** mesosoma, dorsal.

#### ﻿ Alphomelon rostermoragai

Taxon classificationAnimaliaHymenopteraBraconidae

Fernandez-Triana & Shimbori sp. nov.

2E7C9461-0B2D-5F1C-A88A-517A8D611E95

https://zoobank.org/3ED44A5B-B7B5-4312-98CD-84320011E29D

Fig. 74A–E

##### Type material.

***Holotype*.** Costa Rica • Female, CNC; Guanacaste, Area de Conservación Guanacaste, Sector El Hacha, Estacion Los Almendros, 11°01'56.14"N, 85°31'39.94"W, 290m; 28.XII.2011; ex. *Vettiusaurelius*; coll. Elieth Cantillano; Voucher code: DHJPAR0049074; Host voucher code: 11-SRNP-23685.

##### Distribution.

Costa Rica (ACG).

##### Biology.

Solitary, reared from *Tigasissimplex* and *Vettiusaurelius*.

##### DNA barcoding.

BINBOLD:ACB1223.

##### Etymology.

Named in honor of Sr. Roster Moraga in honor of his decades of teamwork in the ACG parataxonomist team.

##### Diagnostic description.

White patch on gena: extending to occiput and onto clypeus. Tegula/humeral complex color: white/yellow. Mesonotum color: mostly dark brown to black. Metasoma color: mostly black or dark brown. Tarsal claws spines: 3. Pterostigma shape: comparatively more elongate, its length ≥ 3.0× its central height and more triangular with its two lower margins more or less straight. T1 sculpture: entirely to mostly smooth. T1 central ridge: clearly marked by two raised carinae. T2 sculpture: entirely to mostly smooth. Ovipositor sheaths length: longer than first segment of metatarsus. Body length: 3.6 mm. Fore wing length: 3.7 mm.

**Figure 74.**
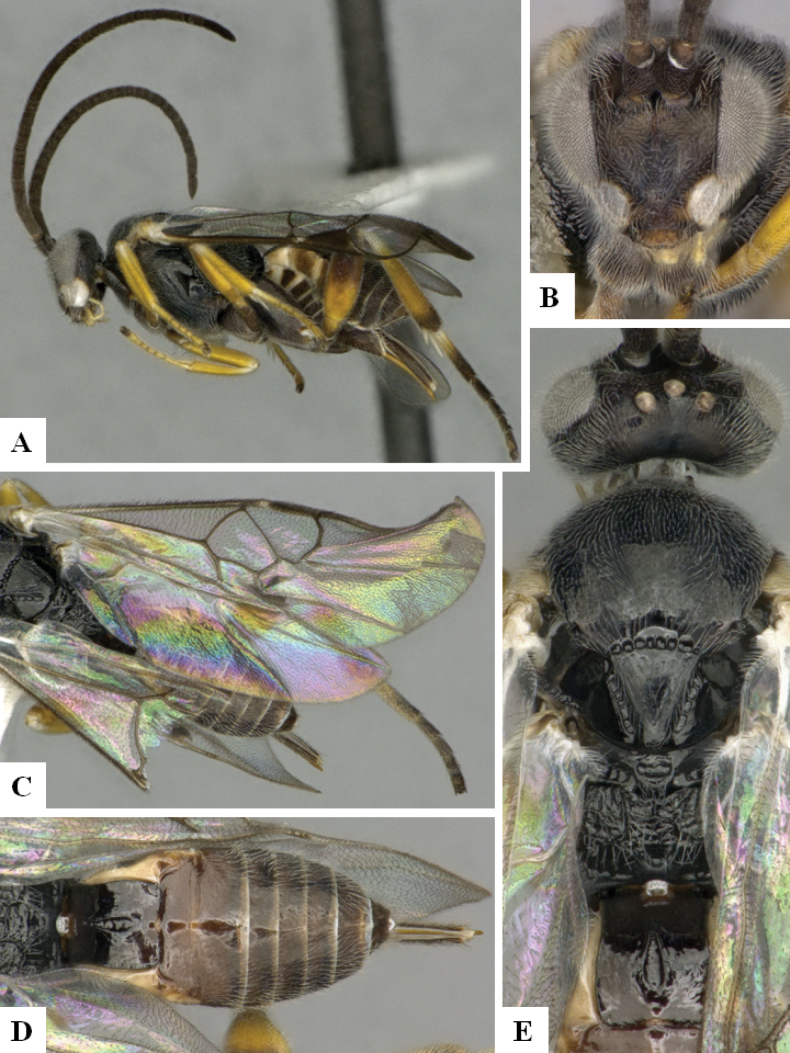
*Alphomelonrostermoragai* Fernandez-Triana & Shimbori holotype female DHJPAR0049074 **A** habitus, lateral **B** head, frontal **C** wings **D** metasoma, dorsal **E** mesosoma, dorsal.

#### ﻿ Alphomelon rugosus

Taxon classificationAnimaliaHymenopteraBraconidae

Shimabukuro & Penteado-Dias, 2003

E173E3A9-2BBF-5FF9-91AD-0043E3D734F9

Fig. 75A–F

##### Distribution.

Brazil (DF, RS, SP).

##### Biology.

Unknown.

##### DNA barcoding.

Not available.

##### Notes.

*Alphomelonrugosus* is morphologically similar to *A.rhyssocercus*, and could end up being the same species when more specimens can be examined and DNA barcodes obtained. For the time being, we consider it as a valid, distinct species, based on the differences outlined in the key.

**Figure 75.**
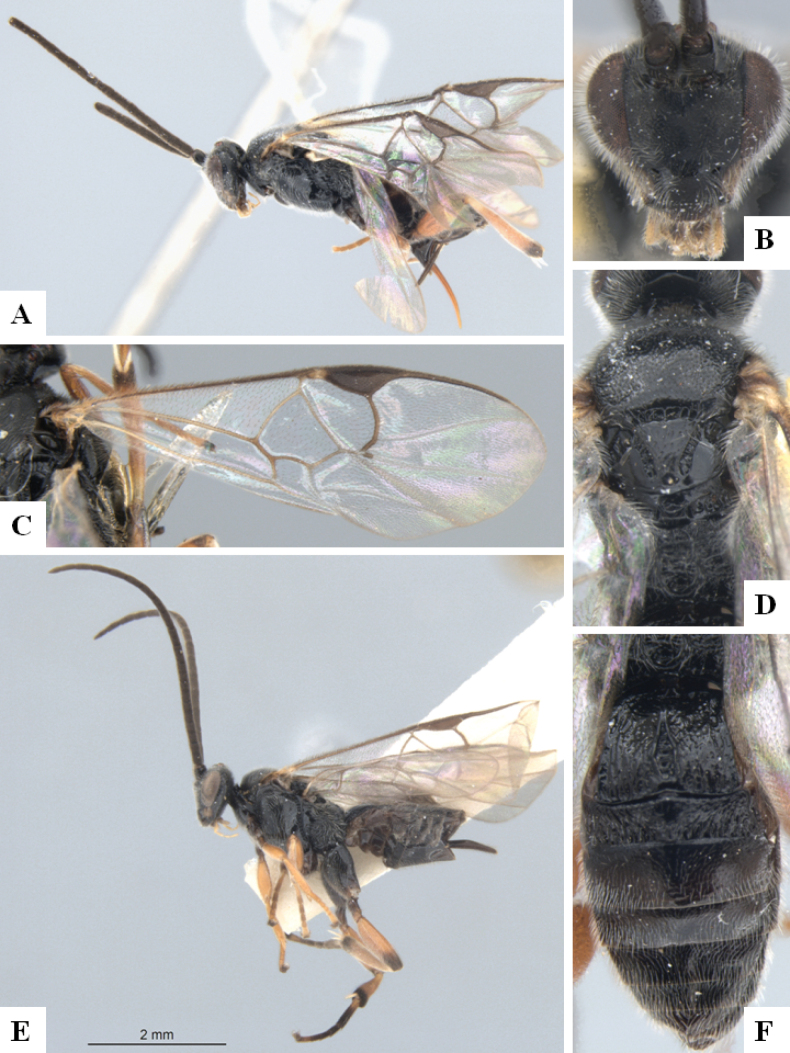
*Alphomelonrugosus* Shimabukuro & Penteado-Dias holotype female DCBU513130 (**A, B, D–F**), paratype female canhim86 (**C**) and paratype female IBGE (**E**) **A** habitus, lateral **B** head, frontal **C** fore wing. **D** mesosoma, dorsal **E** habitus, lateral **F** metasoma, dorsal.

#### ﻿ Alphomelon sergioriosi

Taxon classificationAnimaliaHymenopteraBraconidae

Fernandez-Triana & Shimbori sp. nov.

A63A4791-1650-5D65-918E-CECF5AF8E2A5

https://zoobank.org/7C8FCE40-332B-40AB-9D20-A06D19C55399

Figs 76A–F , 102B

##### Type material.

***Holotype*.** Costa Rica • Female, CNC; Guanacaste, Area de Conservación Guanacaste, Sector Santa Rosa, Cuesta Canyon Tigre, 10°49'01.31"N, 85°38'37.18"W, 270m; 31.VII.1992; ex. *Calpodesethlius*; coll. Parataxonomists; Voucher code: DHJPAR0013671; Host voucher code: 92-SRNP-4136.

***Paratypes*.** Costa Rica • 7 females, 1 male, CNC; DHJPAR0013669, DHJPAR0053837, DHJPAR0053814, DHJPAR0053819, DHJPAR0053835, DHJPAR0031007, DHJPAR0013665, DHJPAR0053842.

##### Other specimens examined.

Mexico • 1 Female, CBG. Voucher code: 07TAPACH-01765.

##### Distribution.

Costa Rica (ACG), Mexico.

##### Biology.

Gregarious, reared from *Calpodesethlius*, *Enosisangularis*, *Pericharesadela*, and *Trombaxanthura*.

##### DNA barcoding.

BINBOLD:AAD2561.

##### Etymology.

Named in honor of Sr. Sergio Ríos in honor of his decades of teamwork in the ACG parataxonomist team.

##### Diagnostic description.

White patch on gena: extending to occiput but not to clypeus. Tegula/humeral complex color: white/yellow. Mesonotum color: mostly dark brown to black. Metasoma color: mostly black or dark brown. Tarsal claws spines: 1. Pterostigma shape: comparatively more elongate, its length ≥ 3.0× its central height and more triangular-with its two lower margins more or less straight. T1 sculpture: strongly sculptured on at least apical half or more. T1 central ridge: strongly marked by two raised carinae and strong costulae centrally. T2 sculpture: entirely to mostly strongly sculptured. Ovipositor sheaths length: longer than first segment of metatarsus. Body length: 3.8–4.2 mm. Fore wing length: 4.1–4.2 mm.

**Figure 76.**
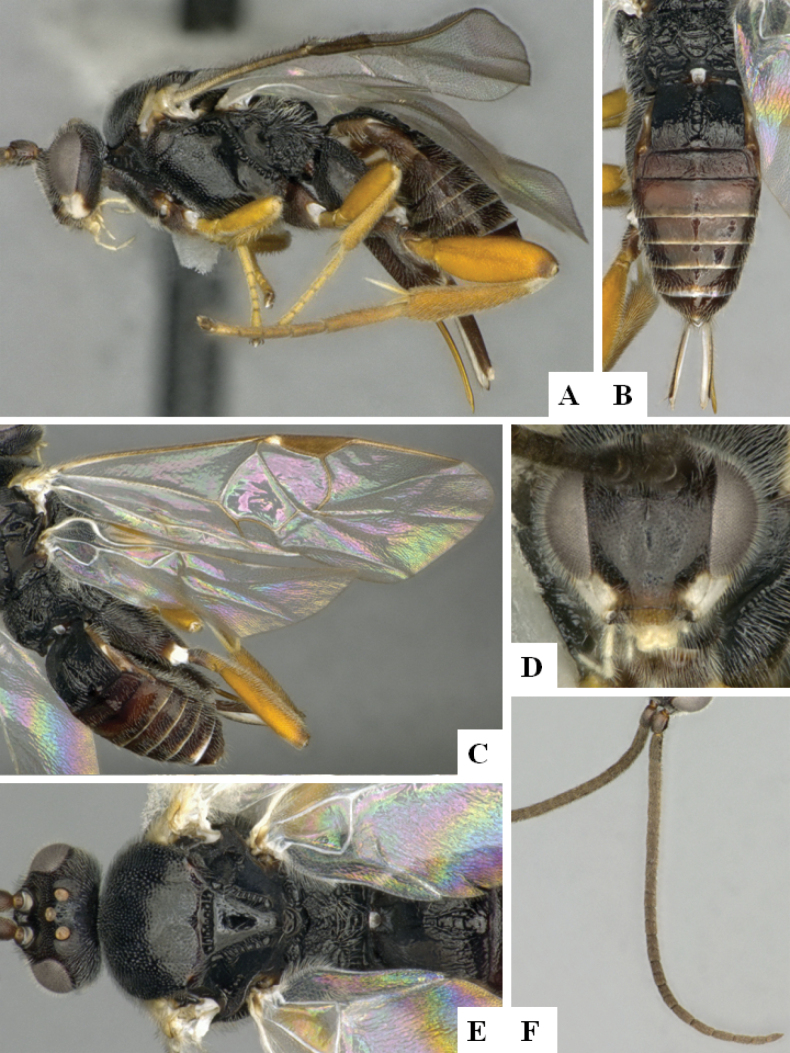
*Alphomelonsergioriosi* Fernandez-Triana, Shimbori holotype female DHJPAR0013671 **A** habitus, lateral **B** propodeum and metasoma, dorsal **C** wings **D** head, frontal **E** head and mesosoma, dorsal **F** antenna, dorsal.

##### Notes.

The specimen from Mexico (having a full barcode) is associated with the species because its sequence matches perfectly with ACG specimens and the image available in BOLD is also similar.

#### ﻿ Alphomelon simpsonorum

Taxon classificationAnimaliaHymenopteraBraconidae

Deans, 2003

CFCF3139-273C-508D-849B-C3725A30CCA3

Figs 77A–G , 78A–E

##### Distribution.

Brazil (PR, SC), Costa Rica, Paraguay; all known records are from rainforest sites.

##### Biology.

Solitary, reared from unidentified Hesperiinae feeding on Poaceae in Costa Rica (no ACG) (Deans et a. 2003).

##### DNA barcoding.

Two partial barcodes (164 and 234 bp).

##### Other specimens examined.

(4 females): CNC1197001, CNCHYM 00051, CNCHYM 00052, CNC704289.

**Figure 77.**
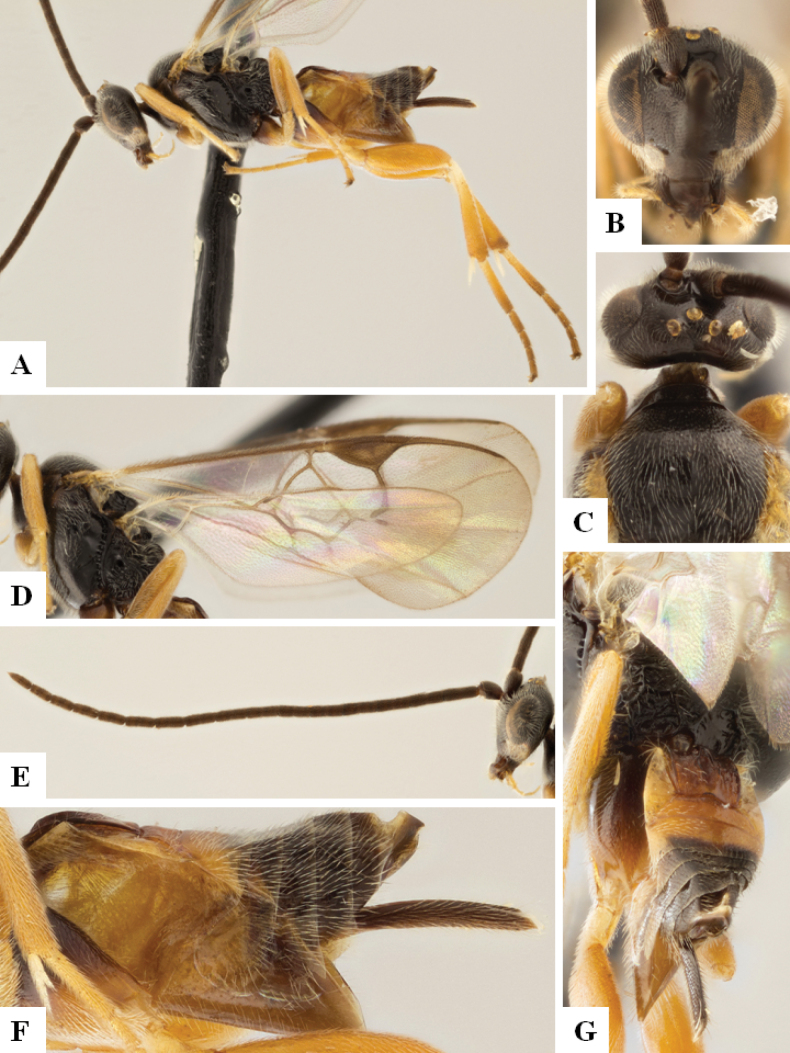
*Alphomelonsimpsonorum* Deans holotype female **A** habitus, lateral **B** head, frontal **C** Wings **D** head and mesosoma, dorsal **E** antenna, lateral **F** metasoma, lateral **G** propodeum and metasoma, dorso-lateral.

**Figure 78.**
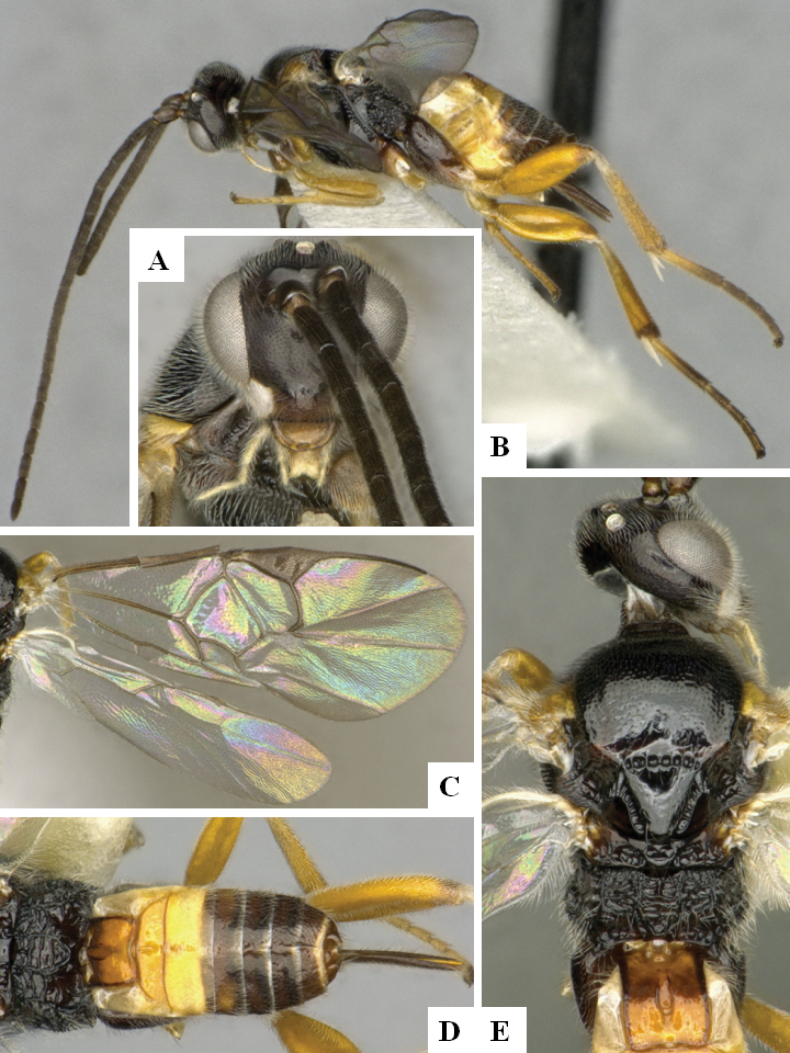
*Alphomelonsimpsonorum* Deans paratype female CNCHYM 00051 **A** head, frontal **B** habitus, lateral **C** wings **D** propodeum and metasoma, dorsal **E** mesosoma, dorsal.

#### ﻿ Alphomelon talidicida

Taxon classificationAnimaliaHymenopteraBraconidae

(Wilkinson, 1931)

CCAB419A-2DB5-53DD-B9B6-D35CEC4FABC3

**Figure 79.**
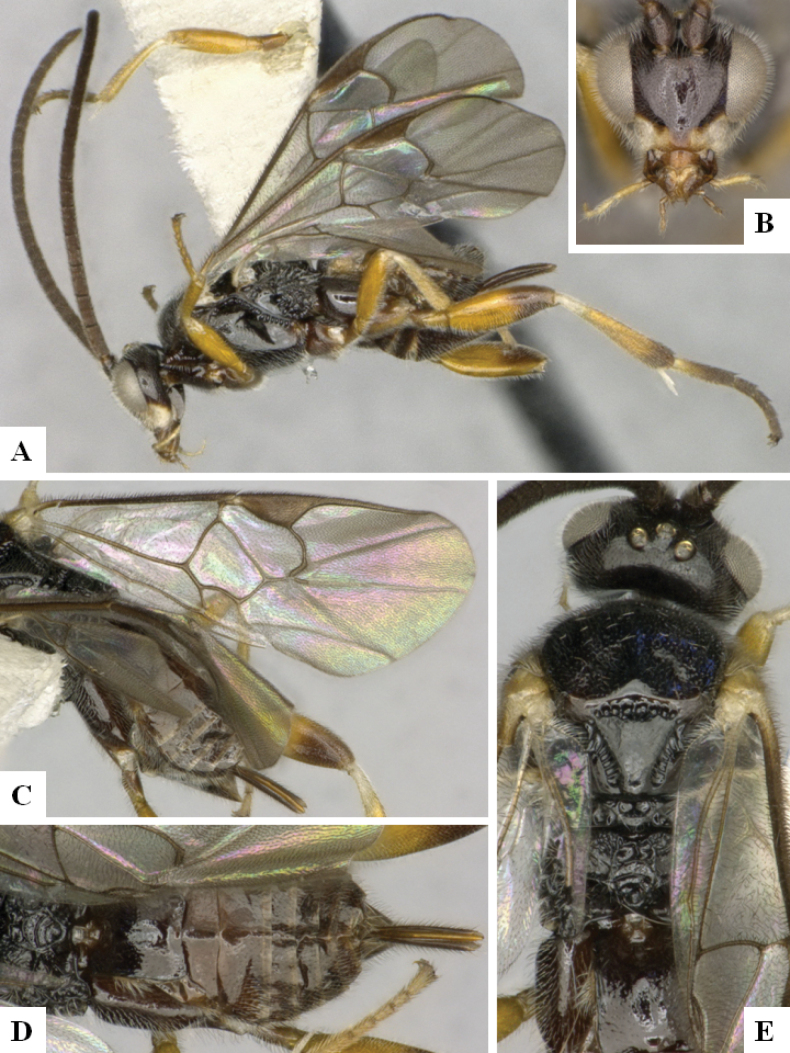
*Alphomelontalidicida* (Wilkinson) female CNC280523 **A** habitus, lateral **B** head, frontal **C** fore wing **D** metasoma, dorsal **E** mesosoma, dorsal.

**Figure 80.**
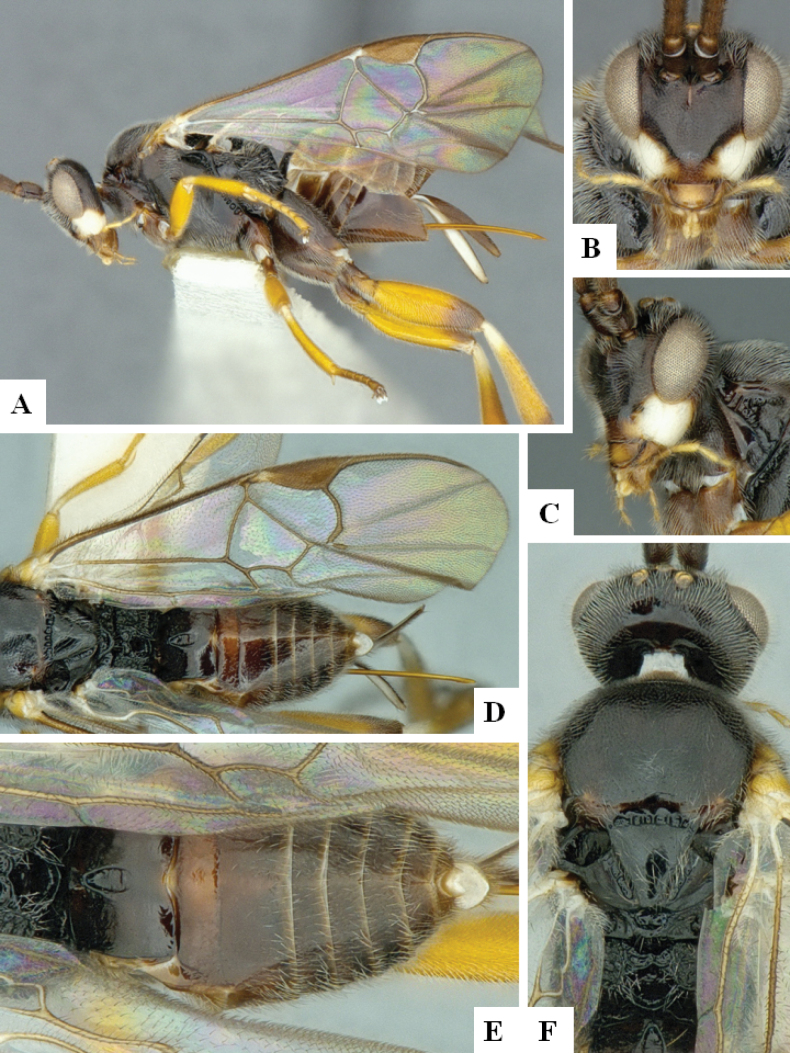
*Alphomelontalidicida* (Wilkinson) female CNC721067 **A** habitus, lateral **B** head, frontal **C** head, fronto-lateral **D** fore wing **E** metasoma, dorsal **F** mesosoma, dorsal.

**Figure 81.**
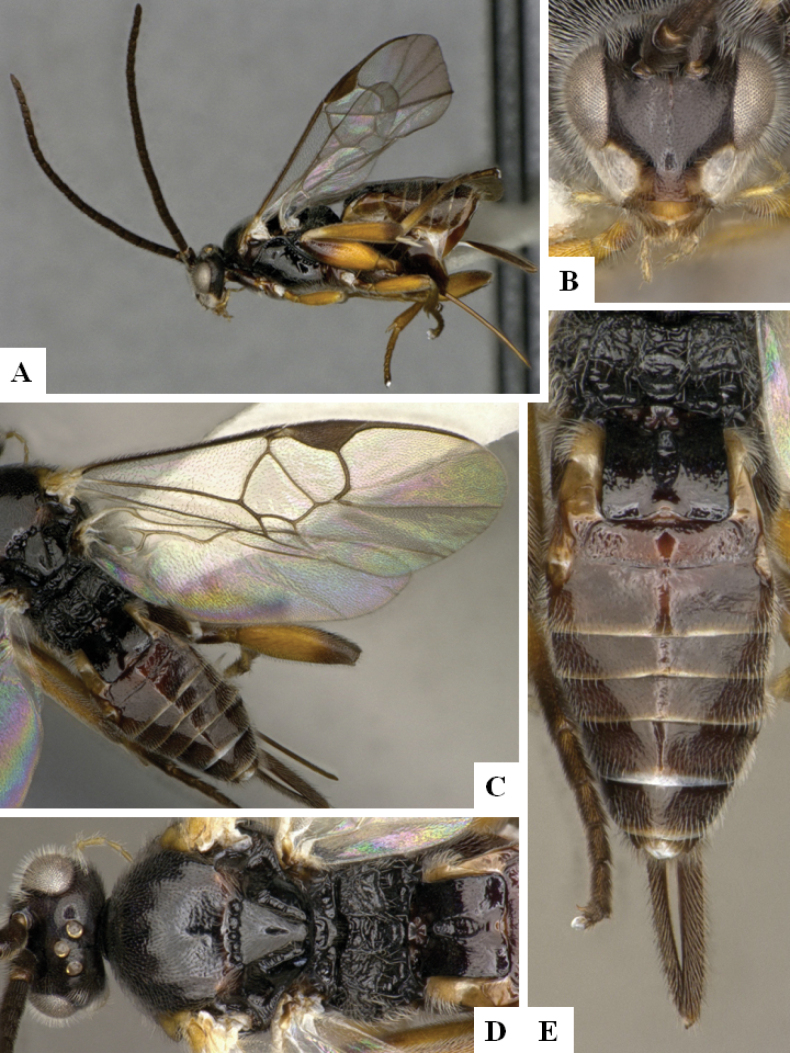
*Alphomelontalidicida* (Wilkinson) female CNC721079 **A** habitus, lateral **B** head, frontal **C** wings **D** head and mesosoma, dorsal **E** propodeum and metasoma, dorsal.

**Figure 82.**
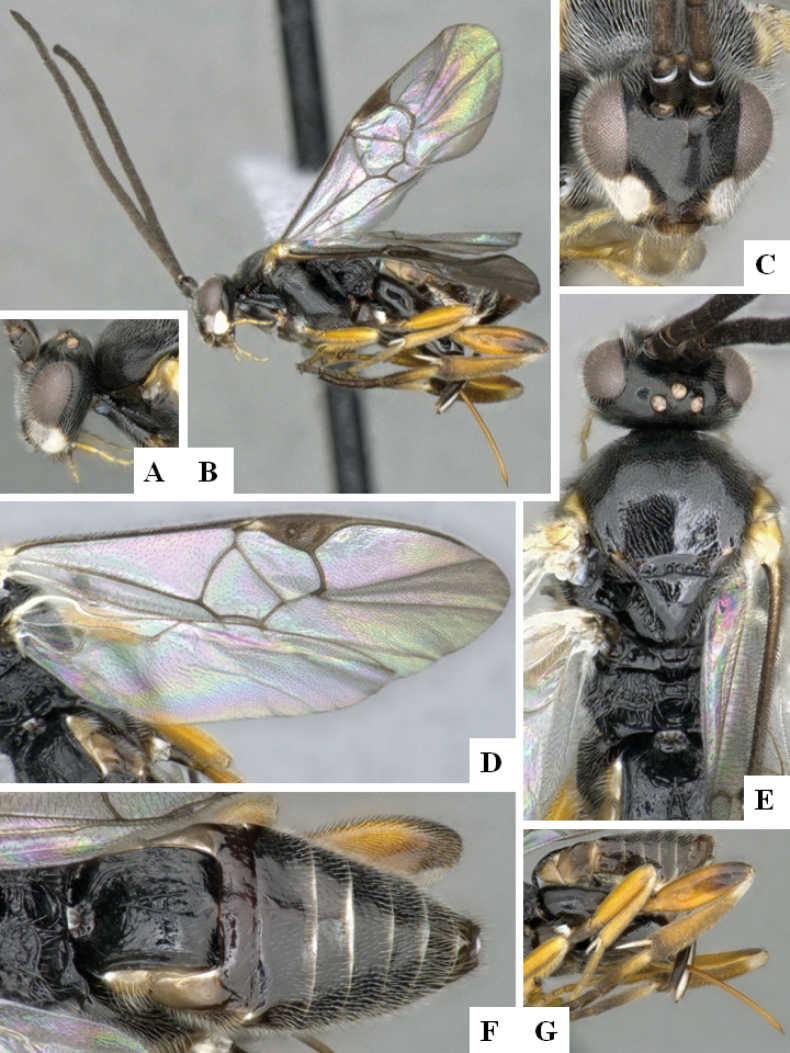
*Alphomelontalidicida* (Wilkinson) female CNC1802118 **A** head, lateral **B** habitus, lateral **C** head, frontal **D** wings **E** head and mesosoma, dorsal **F** metasoma, dorsal **G** metasoma, lateral.

Figs 79A–E , 80A–F , 81A–E , 82A–G , 103A

##### Distribution.

Belize, Brazil (MT, PA, PE, SP), Colombia, Costa Rica (ACG), Ecuador, Guyana, Mexico, Panama, Peru, Trinidad & Tobago, Venezuela.

##### Biology.

Gregarious, reared from *Talidessergestus*, *T.sinois*, *Talides* Burns01, *Talides* Burns02, *Talides* Burns03, *Talides* Burns04 and *Thracidesphidon*, on at least seven species of *Heliconia* (Heliconiaceae).

##### DNA barcoding.

BINBOLD:AAA7259.

##### Other specimens examined.

(94 females, 16 males): DHJPAR0050959, CNC1802102, CNC1802103, CNC1802104, CNC1802105, CNC1802106, CNC1802107, CNC1802108 (additional specimens in a gel capsule associated with that specimen), DHJPAR0050091, CNC1802109, CNC1802110, CNC1802111, CNC1802112, CNC1802113, CNC1802114, CNC1802115, CNC1802116, CNC1802117, DHJPAR0050979, CNC1802118, CNC1802119, CNC1802120, CNC1802121, CNC1802122 (additional specimens in a gel capsule associated with that specimen), DHJPAR0050958, CNC1802123, CNC1802124, CNC1802125, CNC1802126, CNC5213175, CNC5213176, CNC5213177, CNC5213178, CNC5213179, CNC5213180, DHJPAR0051199, CNC5213181, CNC5213182, CNC5213183, CNC5213184 (additional specimens in a gel capsule associated with that specimen), DHJPAR0054700, CNC5213185, CNC5213186, CNC5213187, CNC5213188, CNC5213189, CNC5213190 (additional specimens in a gel capsule associated with that specimen), DHJPAR0055247, DHJPAR0049213, CNC5213191, CNC5213192 (additional specimens in a gel capsule associated with that specimen), DHJPAR0053777, CNC5213193, CNC5213194, CNC5213195, CNC5213196, CNC5213197, CNC5213198, CNC5213199, CNC5213200, CNC5213201, CNC5213202, CNC5213203, CNC5213204, CNC5213105, DHJPAR0051203, CNC5213206, CNC5213207, CNC5213208, CNC5213209, CNC5213210, CNC5213211, CNC5213212 (additional specimens in a gel capsule associated with that specimen), CNC721062, CNC721063, CNC721064, CNC721065, CNC721066, CNC721067, CNC721068, CNC721069, CNC721071, CNC721072, CNC721073, CNC721074, CNC721075, CNC721076, CNC721077, CNC280522, CNC721078, CNC721087, CNC721086, CNC721085, CNC721083, CNC721082, CNC721080, CNC721088, CNC721089, CNC721090, CNC721091, CNC721092, CNCHYM 00053, CNCHYM 00054, CNCHYM 00055, CNCHYM 00056, CNC721079, CNC280523, CNC721081, CNC721084, CNC721070.

#### ﻿ Alphomelon winniewertzae

Taxon classificationAnimaliaHymenopteraBraconidae

Deans, 2003

8F3EABED-328F-541A-96FB-6825105B7237

**Figure 83.**
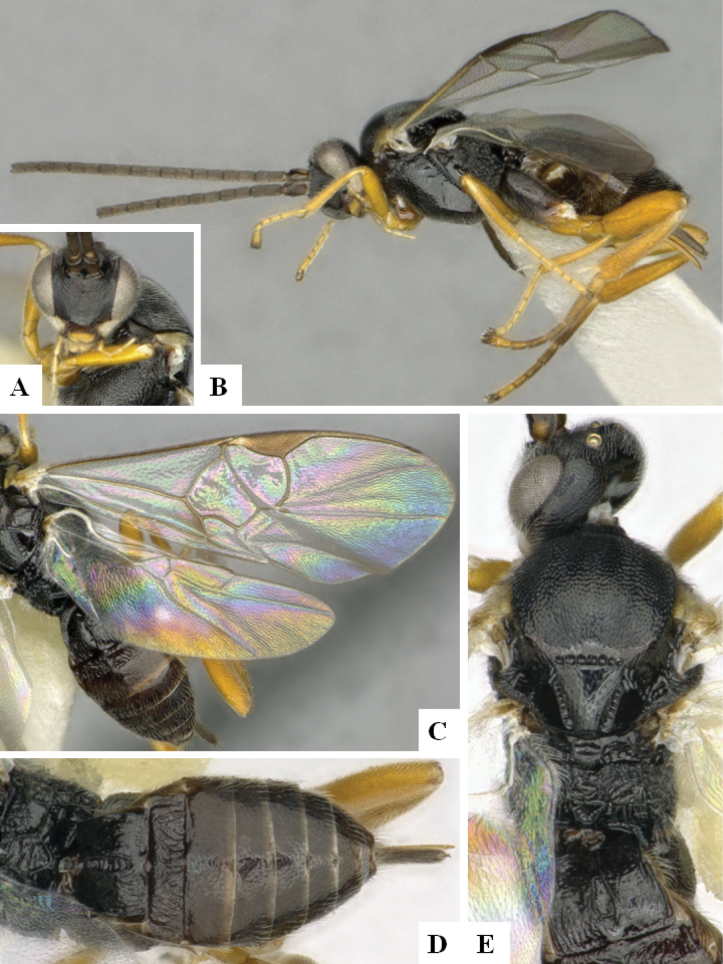
*Alphomelonwinniewertzae* Deans female CNC721098 **A** head, frontal **B** habitus, lateral **C** wings **D** metasoma, dorsal **E** mesosoma, dorsal.

**Figure 84.**
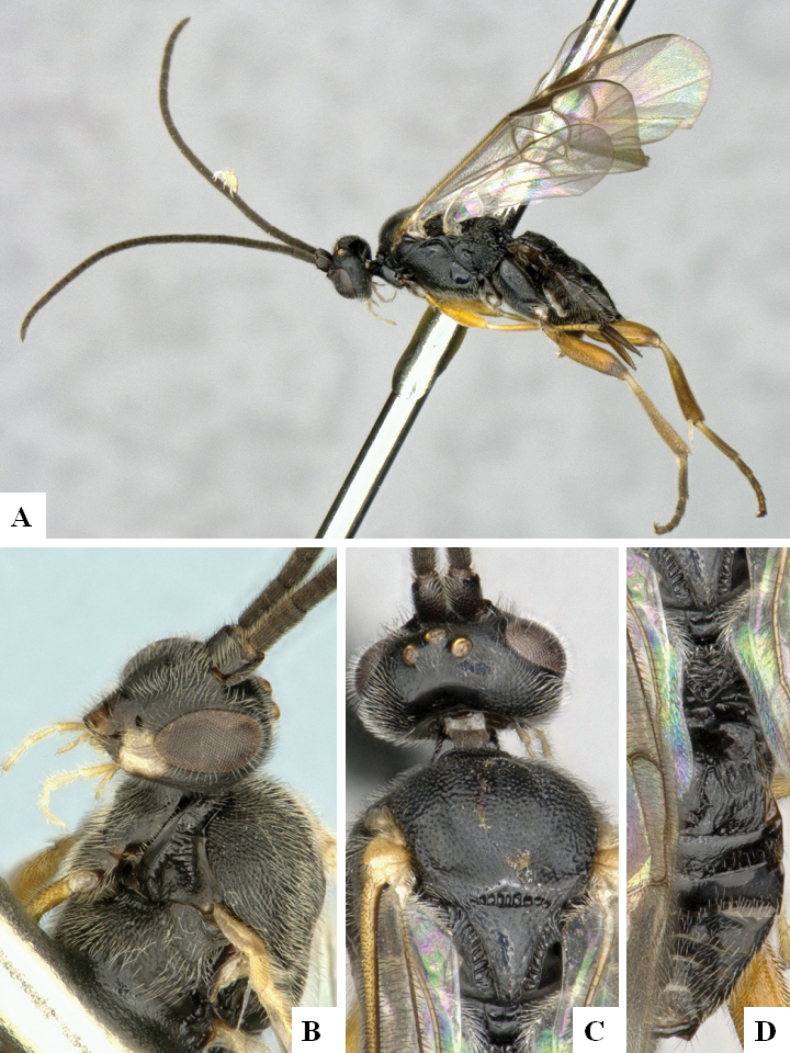
*Alphomelonwinniewertzae* Deans female CNCHYM 00025 **A** habitus, lateral **B** head, fronto-lateral **C** mesosoma, dorsal **D** metasoma, dorsal.

**Figure 85.**
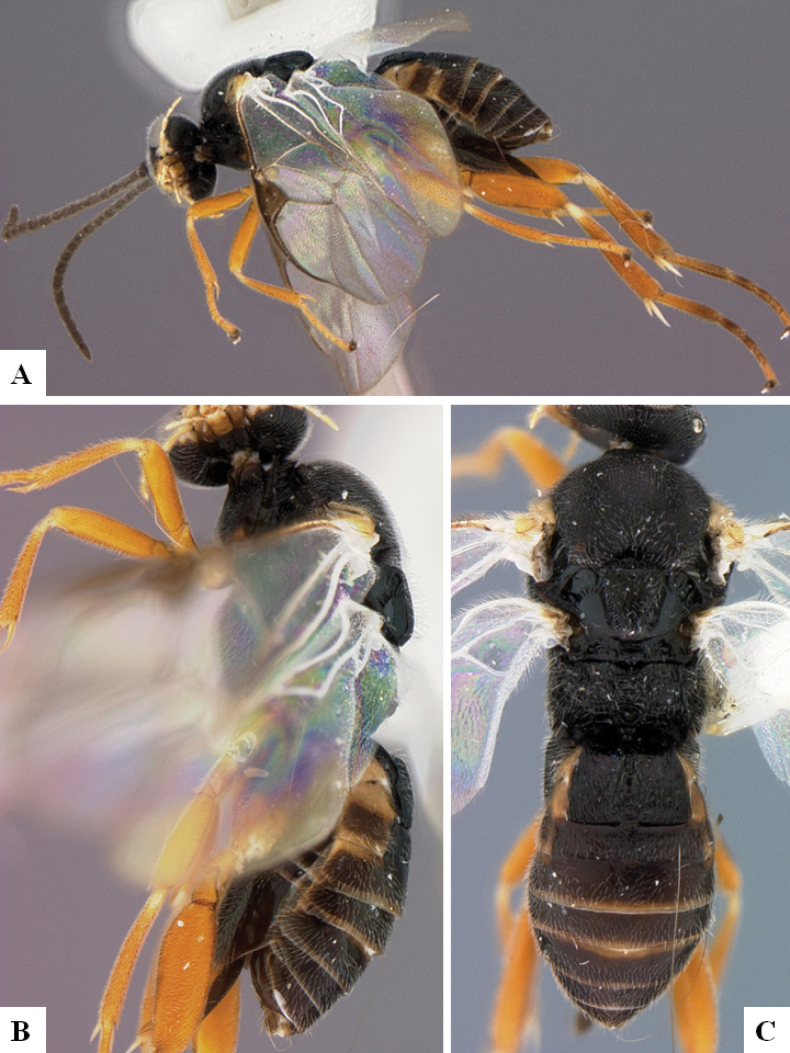
*Alphomelonwinniewertzae* Deans holotype female USNMENT00828301 **A** habitus, lateral **B** close-up of habitus, lateral **C** habitus, dorsal.

Figs 83A–E , 84A–D , 85A–C

##### Distribution.

Canada (ON, QC), Mexico, United States (AR, DC, FL, KS, MA, MI, NC, OH, TN, TX, VA); collected in variable habitats throughout its range.

##### Biology.

Solitary, reared from *Euphyes* spp. in the United States (TX).

##### DNA barcoding.

No BIN but five partial barcodes (from 103 to 382 bp) available.

##### Other specimens examined.

(8 females, 5 males, 1 sex unknown): CNC721097, CNC280524, CNC721095, CNC721100, CNCHYM 00057, CNCHYM 00060, CNCHYM 00025, CNC721098, CNCHYM 00058, CNC721093, CNC721094, CNC721096, CNC721099, CNCHYM 00059.

##### Notes.

We have not found any evidence supporting the species presence in Costa Rica, as it was previously reported in Deans et al. (2003); thus, we here remove the country record as well as associated host data. It is also possible that the two specimens recorded from Mexico (from Guadalajara and Veracruz, see Deans et al. 2003: 38) are incorrect, but we have not been able to examine those specimens to conclude. The species is therefore considered in this paper to be mostly (or perhaps entirely) Nearctic, with a relatively broad distribution in eastern North America, including Canada up to 45° N.

#### ﻿ Alphomelon xestopyga

Taxon classificationAnimaliaHymenopteraBraconidae

Deans, 2003

C1A875C9-524D-5265-8141-EAF9EDFCABDF

**Figure 86.**
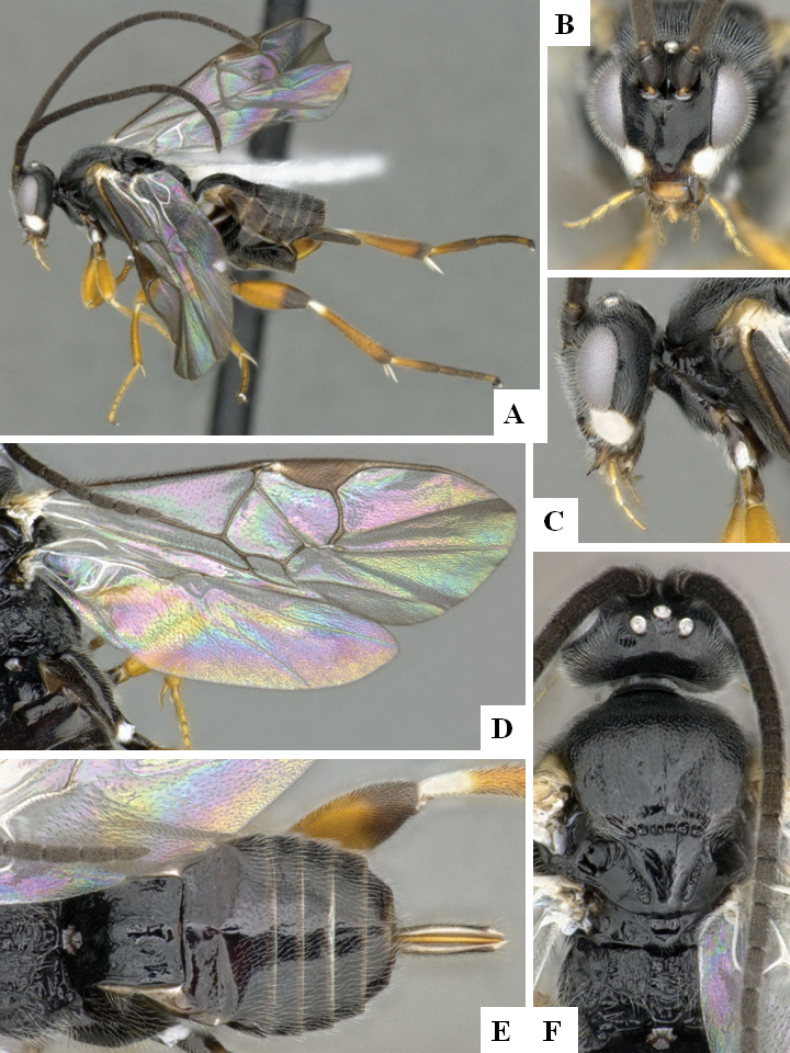
*Alphomelonxestopyga* Deans female CNC308749 **A** habitus, lateral **B** head, frontal **C** head, lateral **D** wings **E** metasoma, dorsal **F** mesosoma, dorsal.

**Figure 87.**
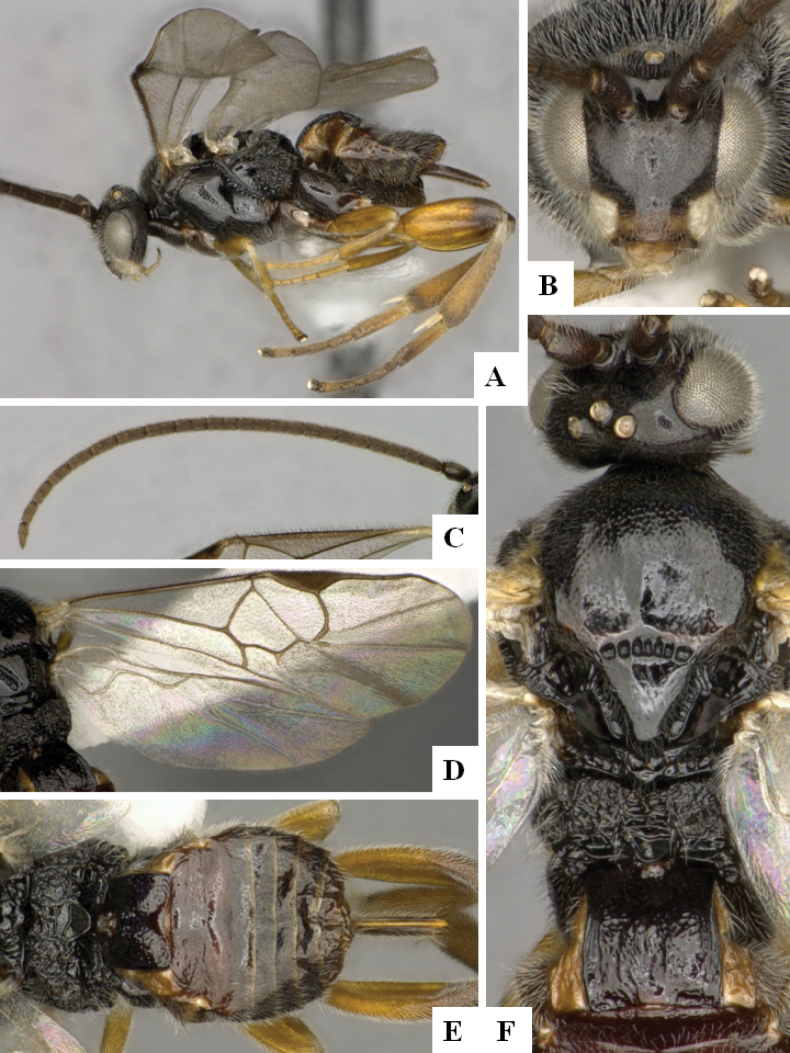
*Alphomelonxestopyga* Deans female CNC721122 **A** habitus, lateral **B** head, frontal **C** antenna, lateral **D** wings **E** propodeum and metasoma, dorsal **F** mesosoma, dorsal.

**Figure 88.**
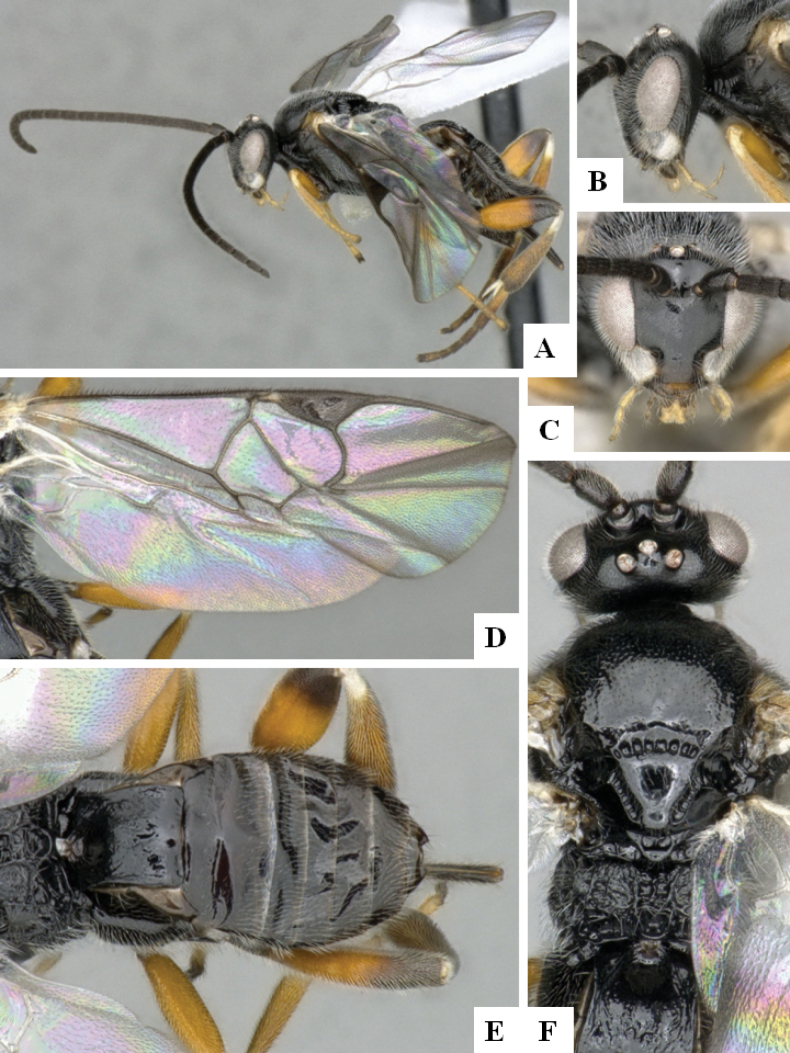
*Alphomelonxestopyga* Deans female CNC1620777 **A** habitus, lateral **B** head, lateral **C** head, frontal **D** wings **E** metasoma, dorsal **F** head and mesosoma, dorsal.

**Figure 89.**
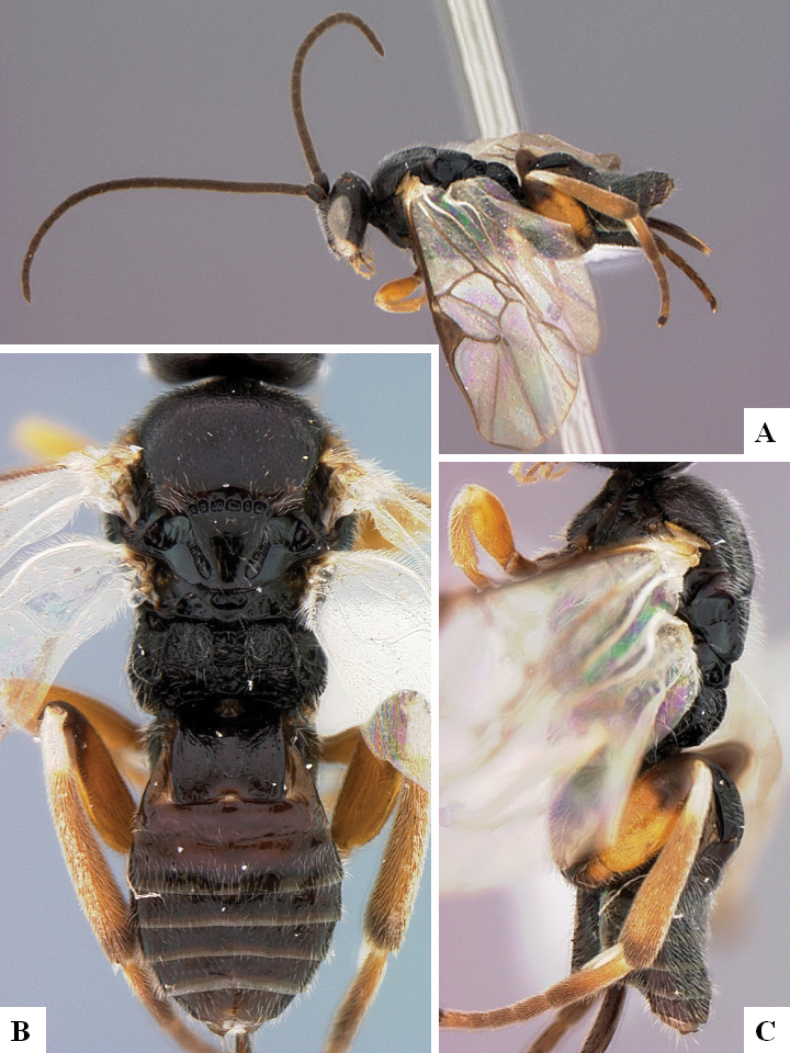
*Alphomelonxestopyga* Deans holotype female USNMENT00828302 **A** habitus, lateral **B** habitus, dorsal **C** close-up of habitus, lateral.

Figs 47A–G , 86A–F , 87A–F , 88A–F , 89A–C , 103B

##### Distribution.

Costa Rica (ACG).

##### Biology.

Gregarious, reared from *Calpodesesperi*, *C.ethlius*, *C.fusta*, *C.severus*, *Cobalopsisnero*, *Cyneairma*, *Cymaenesodiliatrebius*, *C.* Burns01, *Joannajoanna*, *Justinianorda*, *Moryslyde*, *M.micythus*, *M.valeriusvalda*, *M.* lydeDHJ01, *M.* lydeDHJ02, *Niconiadesincomptus*, *Parphorusdecora*, *Quintacannae*, *R.osca*, *Synaptesalenus*, *S.silius*, and *Vettiusaurelius*.

##### DNA barcoding.

BINBOLD: AAA1634.

##### Other specimens examined

(93 females, 32 males): CNC308749, CNC308837, CNC308838, CNC308839, CNC308840 (additional specimens in a gel capsule associated with that specimen), DHJPAR0049842, CNC1620806, CNC1620807, CNC1620808, CNC1620809, CNC1620810, CNC1620811, DHJPAR0051223, CNC1620802, CNC1620803, CNC1620804, CNC1620805, DHJPAR0031632, DHJPAR0053792, DHJPAR0053789, DHJPAR0053751, CNC1620772, CNC1620773, CNC1620774, CNC1620775, CNC1620776, DHJPAR0053767, CNC1620777, DHJPAR0020278, CNC958681, CNC958682 (additional specimens in a gel capsule associated with that specimen), DHJPAR0053760, CNC1620778, CNC1620779, CNC1620780, CNC1620781, CNC1620782 (additional specimens in a gel capsule associated with that specimen), CNC1620783, CNC1620784, CNC1620785, CNC1620786, CNC1620787, CNC1620788, CNC1620789, CNC1620790, DHJPAR0031616, DHJPAR0031735, DHJPAR0031620, DHJPAR0052957, CNC1620791, CNC1620792, CNC1620793, DHJPAR0053758, CNC1620794, CNC1620795, CNC1620796, CNC1620797, CNC1620798, CNC1620812, CNC1620813, CNC1620814, CNC1620815, CNC1620816, CNC1620817, CNC1620818, CNC1620819, CNC1620820, CNC1620821, CNC1620822, CNC1620823, CNC1620824, CNC1620825, CNC1620826, CNC1620827, CNC1620828, CNC1620829, CNC1620830, CNC1802047, CNC1802048, DHJPAR0053755, CNC1620837, CNC1620831, CNC1620832, CNC1620833, CNC1620834, CNC1620835, CNC1620836, CNC1620799, CNC1620800, CNC1620838, CNC1620839, CNC1620840, CNC1620801, CNC1620841, CNC1620842, CNC1620843, CNC1620844, CNC721102, CNC721114, CNC721118, CNC721119, CNC721122, CNC721123, CNC721125, CNCHYM 00061, CNC721103, CNC721104, CNC721106, CNC721107, CNC721108, CNC721112, CNC721101, CNC721113, CNC721115, CNC721116, CNC721117, CNC721120, CNC721121, CNC721124, CNC721126, CNC280525, CNC721105, CNC721109, CNC721110, CNC721111.

##### Notes.

There are some differences between *A.xestopyga* in the sense of Deans et al. (2003) and the ACG specimens identified as such that we have been able to examine. The species clearly comprises a complex of species, with some specimens we have seen in the CNC collection from other countries besides Costa Rica. Solving this potential complex will require obtaining more DNA barcodes, especially from areas outside of ACG.

#### ﻿ Alphomelon yanayacu

Taxon classificationAnimaliaHymenopteraBraconidae

Fernandez-Triana & Shimbori sp. nov.

E5D30FB0-8520-5C00-8026-DD50E38F82C4

https://zoobank.org/E1745F15-F7D9-435B-8C9B-F172F9B4A53D

Fig. 90A–E

##### Type material.

***Holotype*.** Ecuador • Female, CNC; Guayas, Yanayacu, Las Penas, 2°10'55.74"S, 79°52'31.98"W, 300 m; 29–30.VIII.1977; Voucher code: CNC1065901.

***Paratype*.** Ecuador • Female, CNC. Voucher code: CNC1196598.

##### Distribution.

Ecuador.

##### Biology.

No data.

##### DNA barcoding.

Not available.

##### Etymology.

Named after the holotype locality.

##### Diagnostic description.

White patch on gena: extending to occiput but not to clypeus. Tegula/humeral complex color: yellow/yellow. Mesonotum color: mostly dark brown to black. Metasoma color: mostly black or dark brown. Tarsal claws spines: 3. Pterostigma shape: comparatively less elongate, its length ≤ 2.5× its central height and usually more rounded with at least one of its lower margins curved. T1 sculpture: strongly sculptured on at least apical half or more. T1 central ridge: clearly marked by two raised carinae. T2 weakly sculptured along margins. Ovipositor sheaths length: longer than first segment of metatarsus. Body length: 3.6–3.7 mm. Fore wing length: 3.6–3.8 mm.

**Figure 90.**
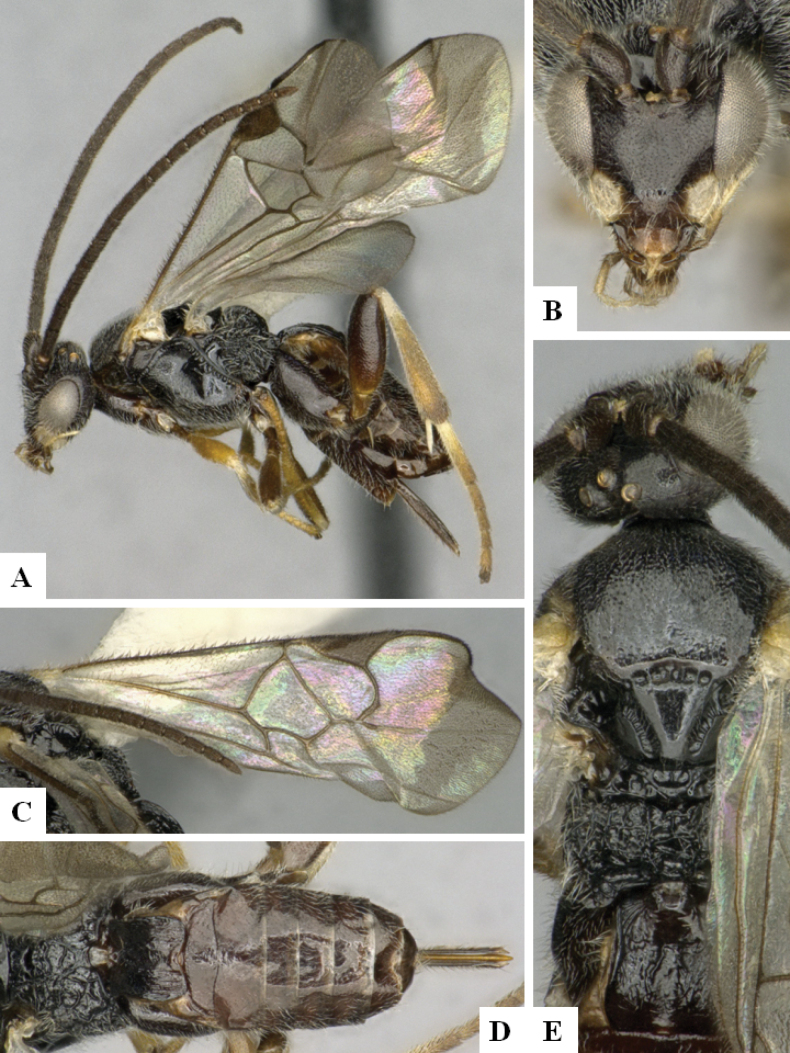
*Alphomelonyanayacu* Fernandez-Triana & Shimbori holotype female CNC1065901 **A** habitus, lateral **B** head, frontal **C** fore wing **D** propodeum and metasoma, dorsal **E** mesosoma, dorsal.

**Figure 91.**
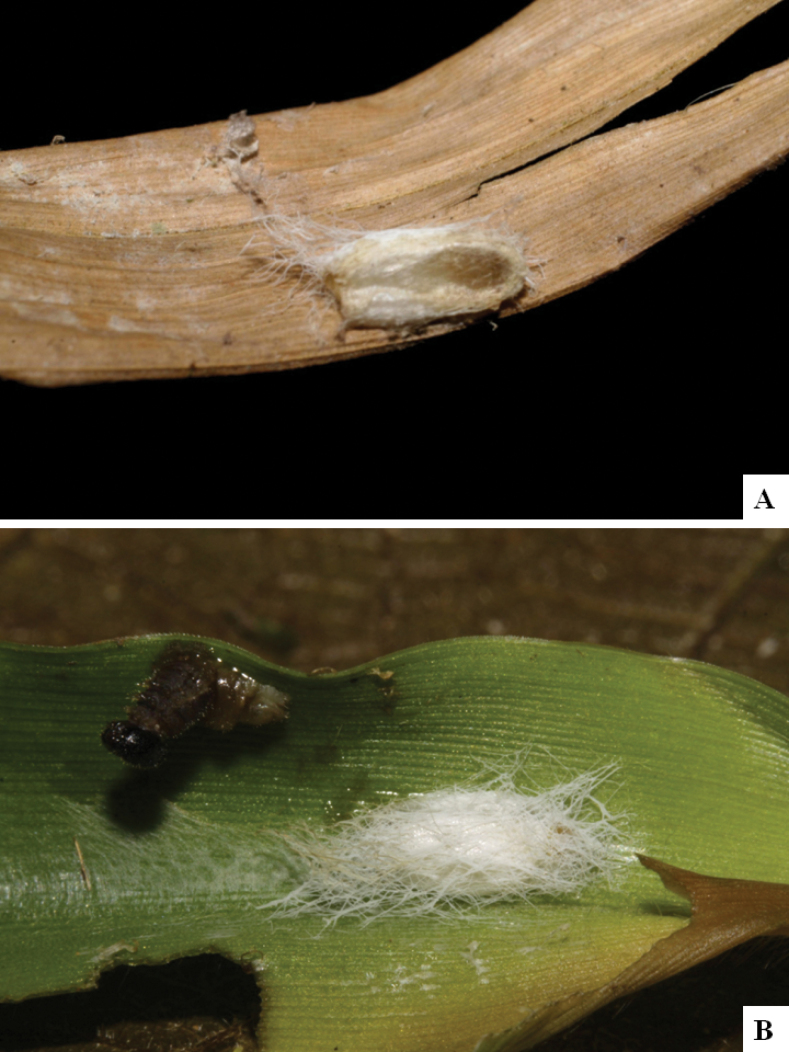
Parasite cocoons **A***Alphomelonadrianguadamuzi* 06-SRNP-34573 DHJ436594 **B***Alphomelonandydeansi* 15-SRNP-32133 DHJ729026.

**Figure 92.**
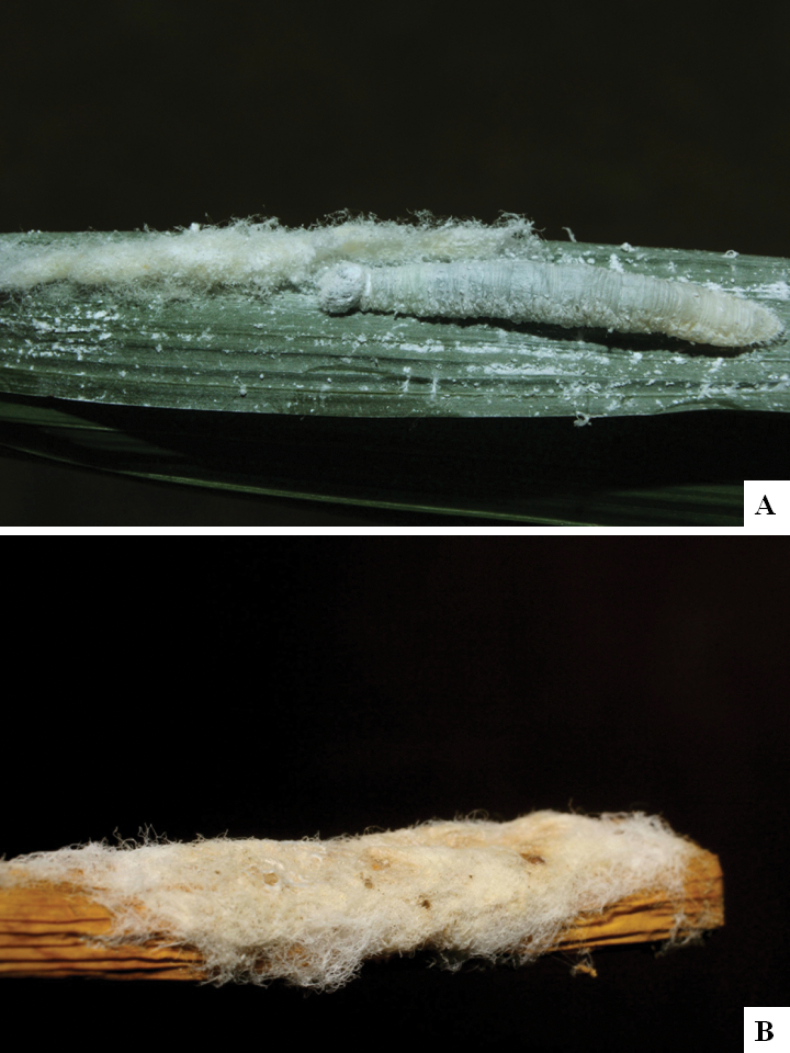
Parasite cocoons **A***Alphomelonarecaphile* 16-SRNP-70716-DHJ732874 **B***Alphomelonbromeliphile* 06-SRNP-60114-DHJ419211.

**Figure 93.**
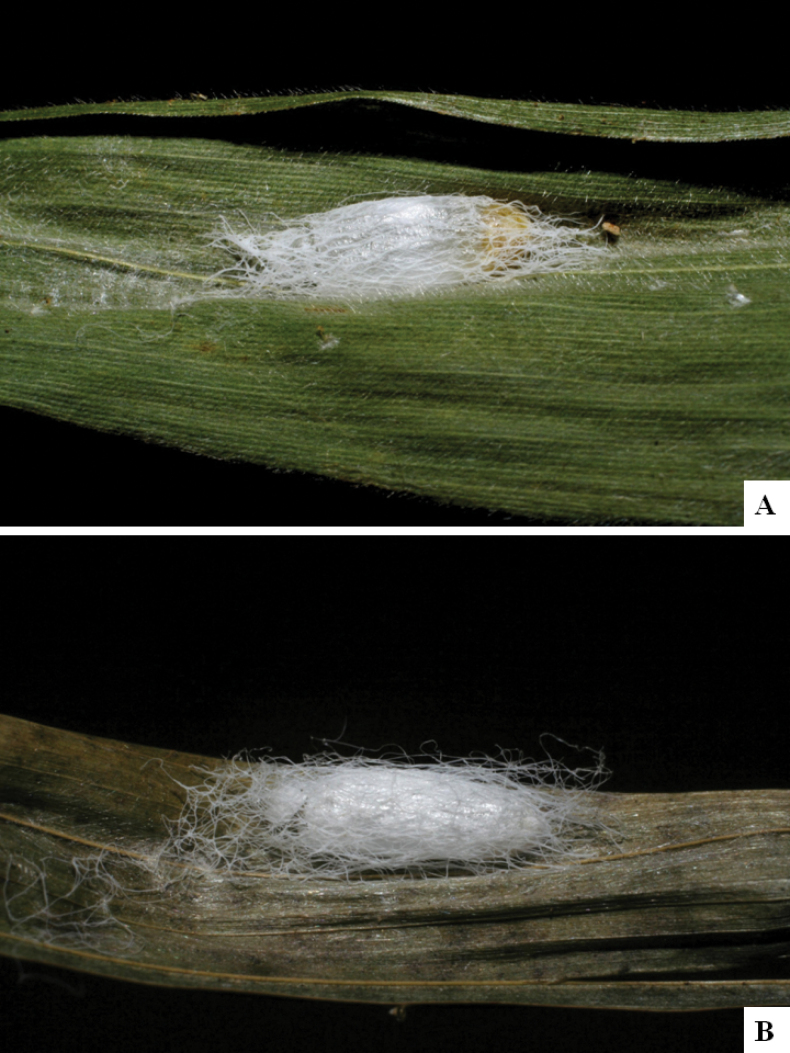
Parasite cocoons **A***Alphomeloncalixtomoragai* 08-SRNP-23629-DHJ448941 **B***Alphomeloncarolinacanoae* 06-SRNP-45140-DHJ414816.

**Figure 94.**
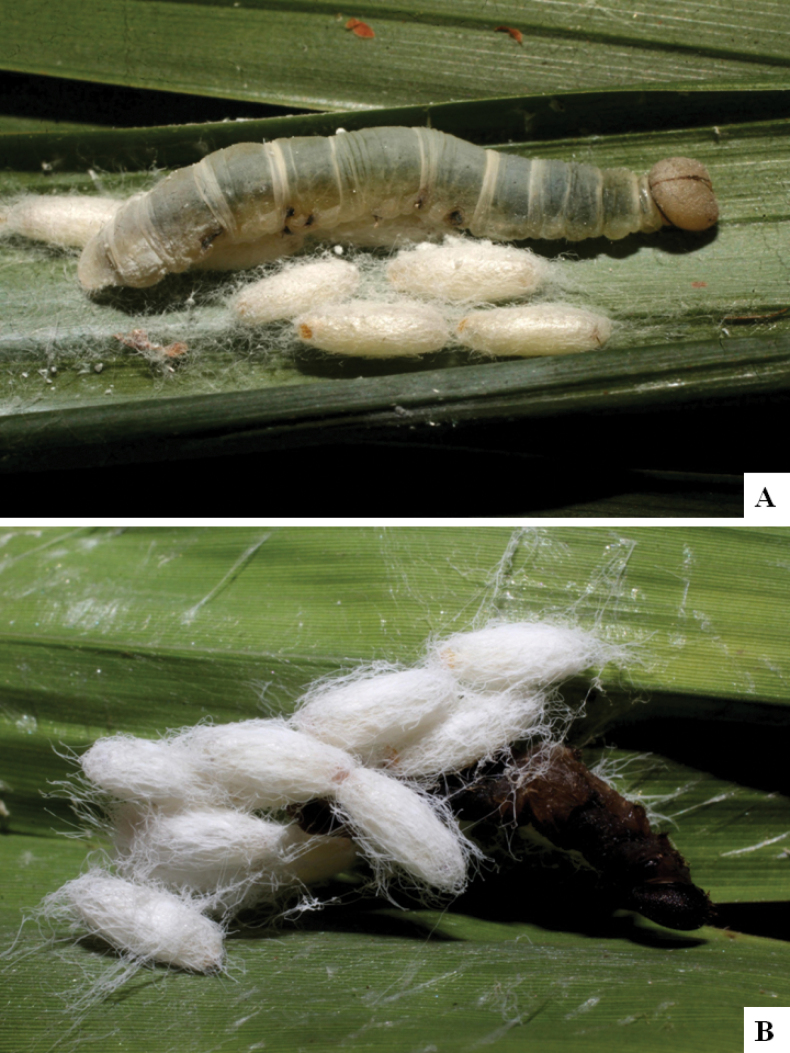
Parasite cocoons **A***Alphomelonchristerhanssoni* 16-SRNP-65052-DHJ488258 **B***Alphomelondiniamartinezae* 15-SRNP-30195-DHJ803603.

**Figure 95.**
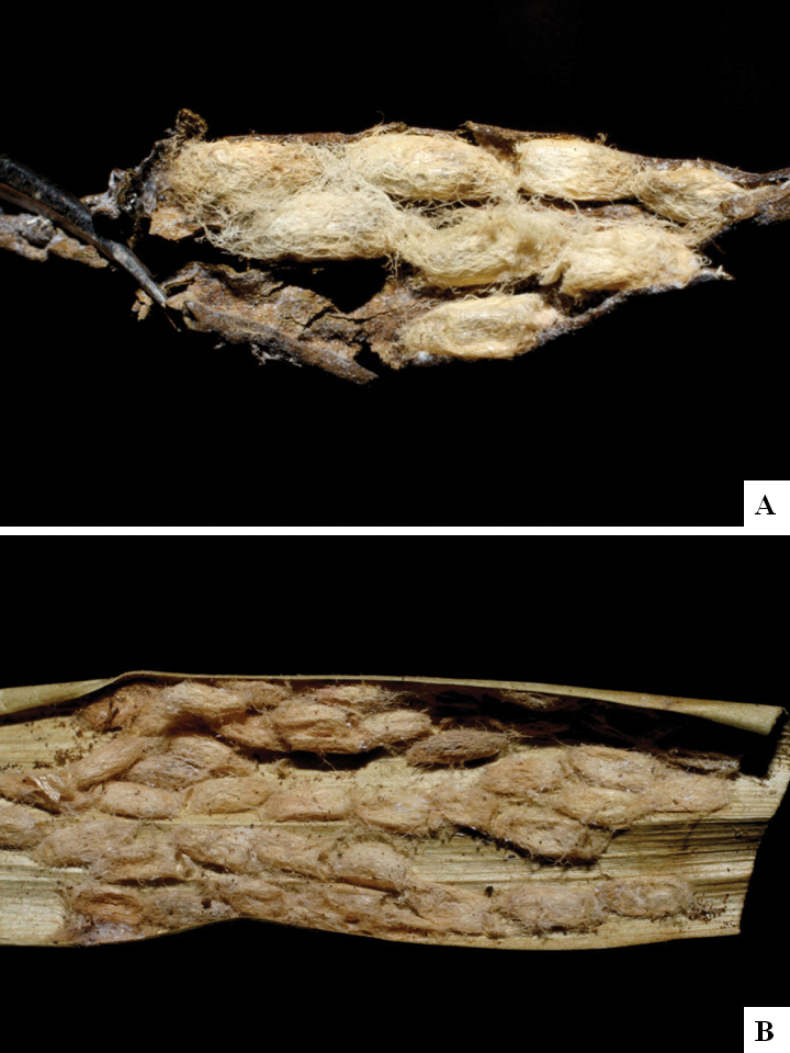
Parasite cocoons **A***Alphomelonduvalierbricenoi* 05-SRNP-24943-DHJ415451 **B***Alphomeloneldaarayae* 05-SRNP-40812-DHJ436395.

**Figure 96.**
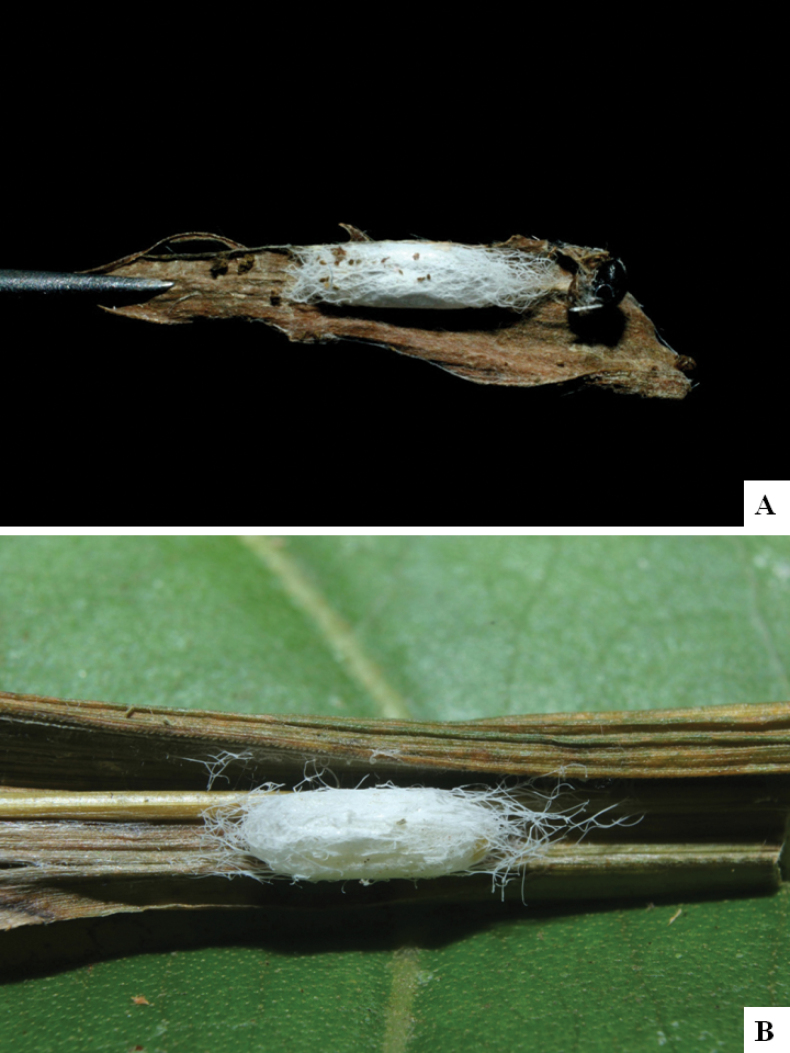
Parasite cocoons **A***Alphomelongloriasihezarae* 09-SRNP-45000-DHJ465227 **B***Alphomelonguillermopereirai* 16-SRNP-70422-DHJ732639.

**Figure 97.**
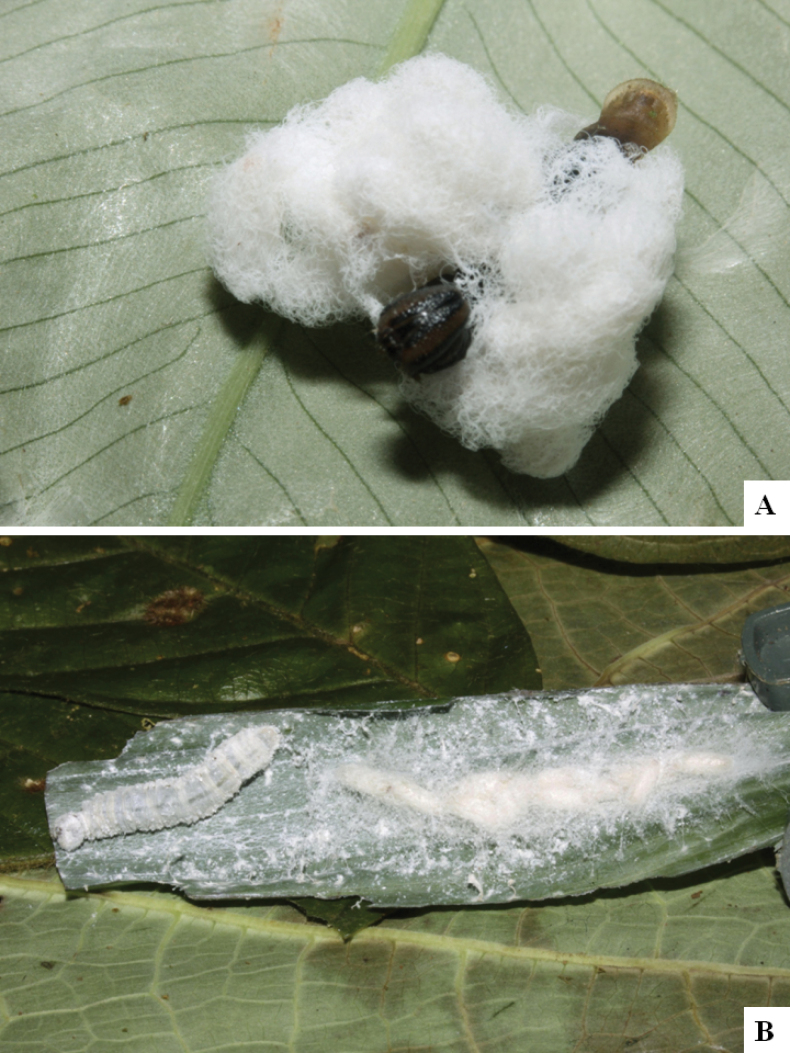
Parasite cocoons **A***Alphomelonhazelcambroneroae* 17-SRNP-27155-DHJ731343 **B***Alphomelonjosecortesi* 15-SRNP-32024-DHJ728940.

**Figure 98.**
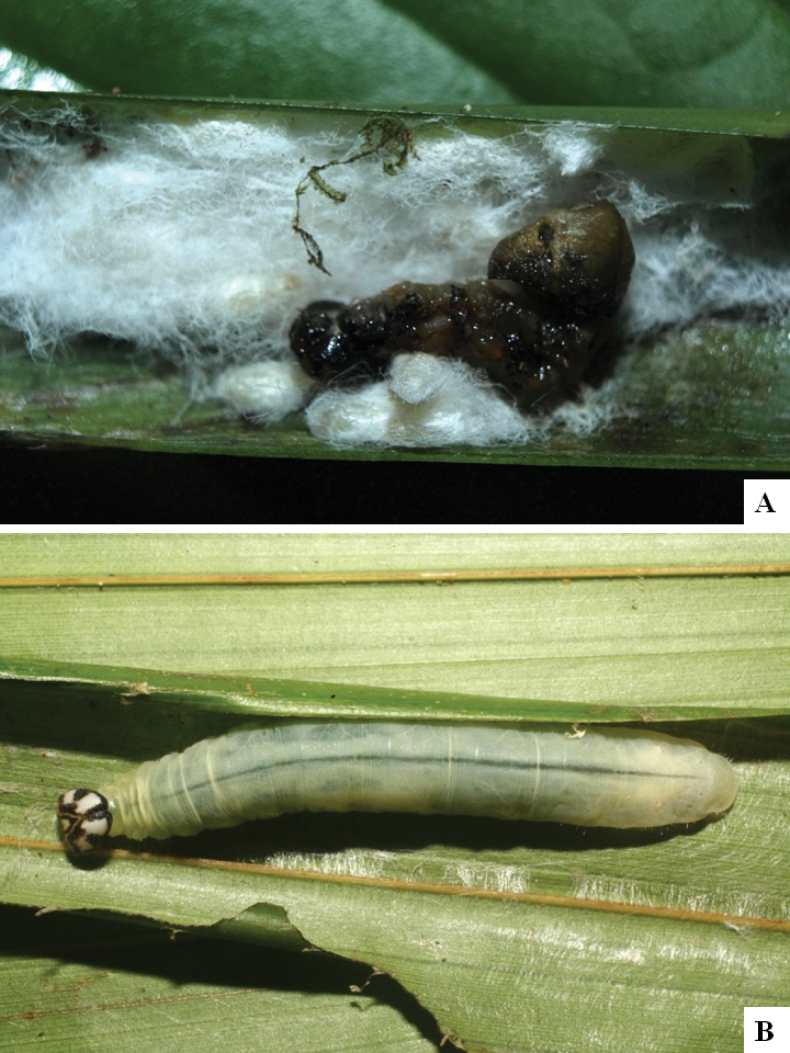
Parasite cocoons and caterpillars **A***Alphomelonkeineraragoni* 15-SRNP-71451-DHJ727801 **B***Alphomelonluciarosae* 12-SRNP-65952-DHJ487984.

**Figure 99.**
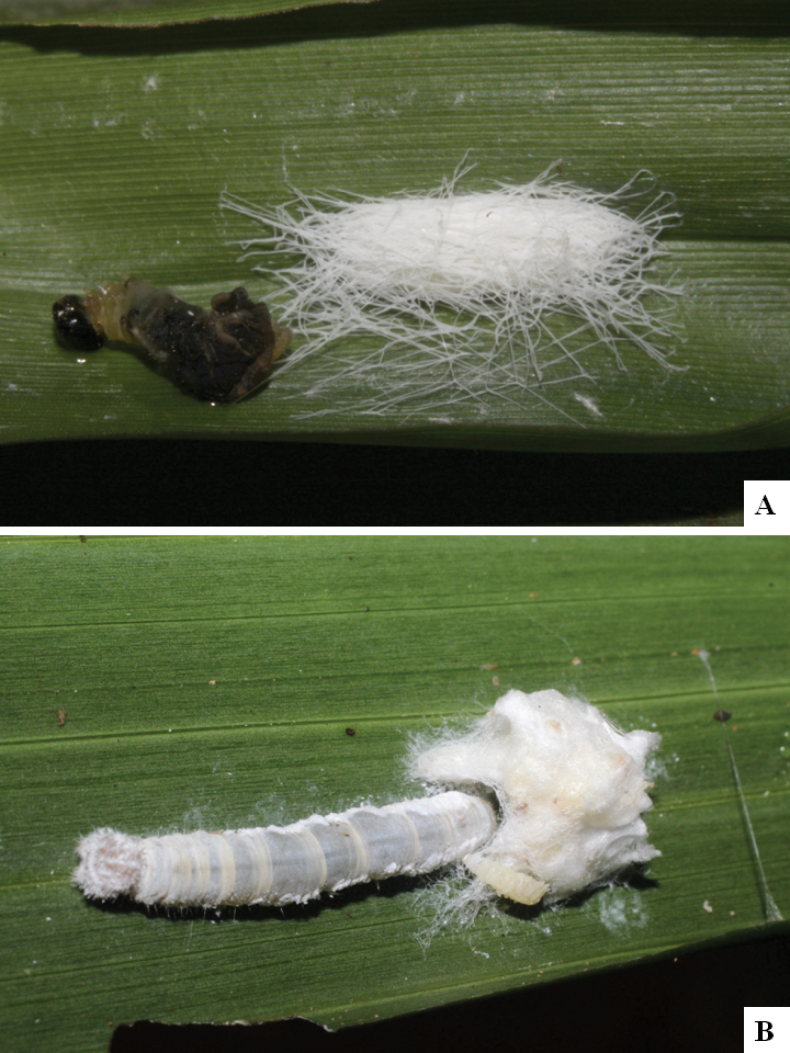
Parasite cocoons and caterpillars **A***Alphomelonmanuelriosi* 15-SRNP-31843-DHJ728686 **B***Alphomelonjosecortesi* 15-SRNP-2670-DHJ704019.

**Figure 100.**
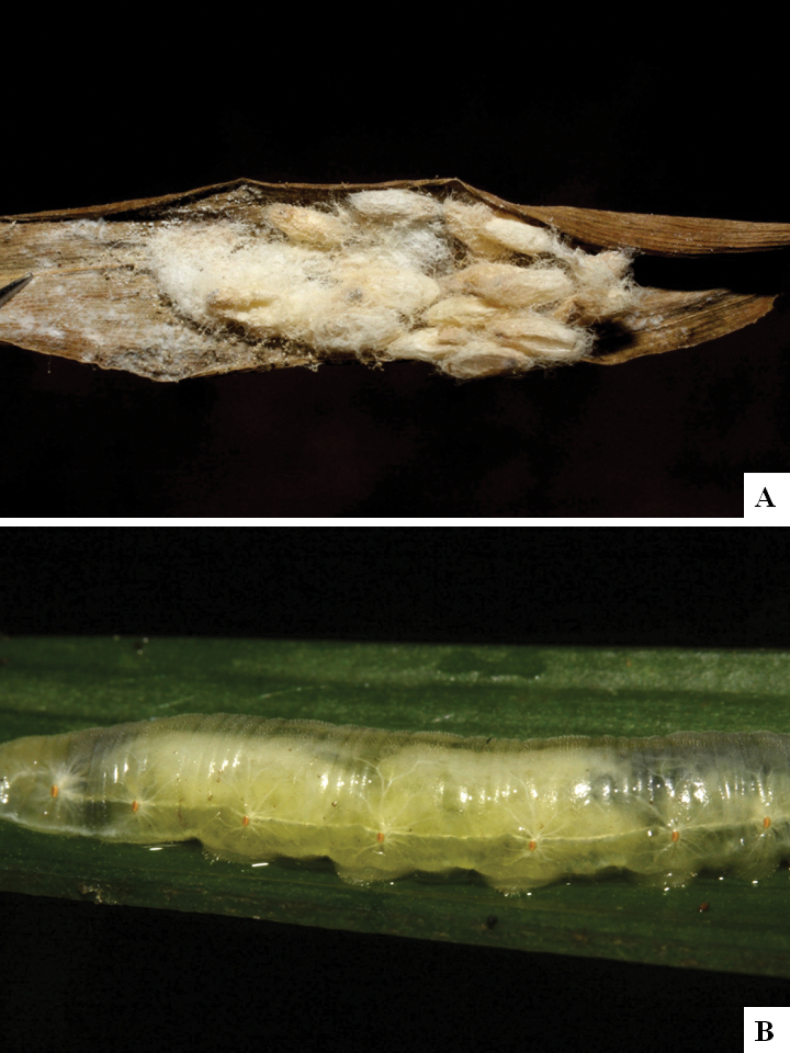
Parasite cocoons and caterpillars **A***Alphomelonmikesharkeyi* 09-SRNP-72362-DHJ474249 **B***Alphomelonnanosoma* 15-SRNP-31923-DHJ728702.

**Figure 101.**
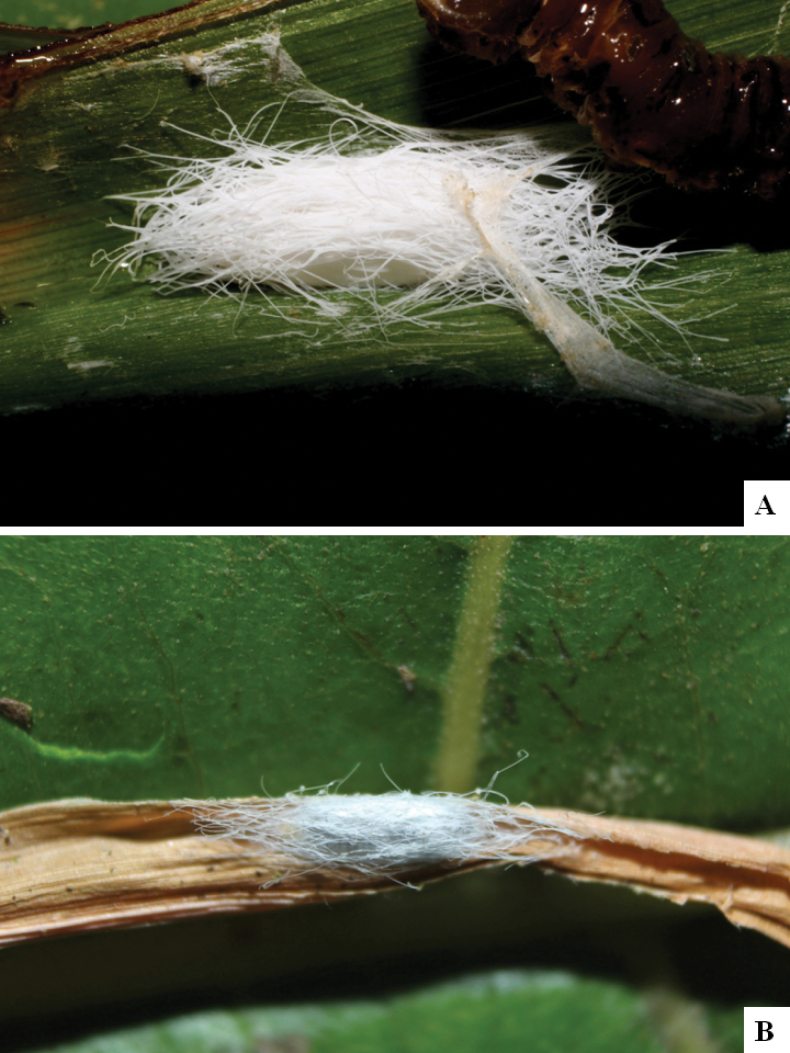
Parasite cocoons **A***Alphomelonosvaldoespinozai* 08-SRNP-72533-DHJ447339 **B***Alphomelonpetronariosae* 15-SRNP-71861-DHJ727939.

**Figure 102.**
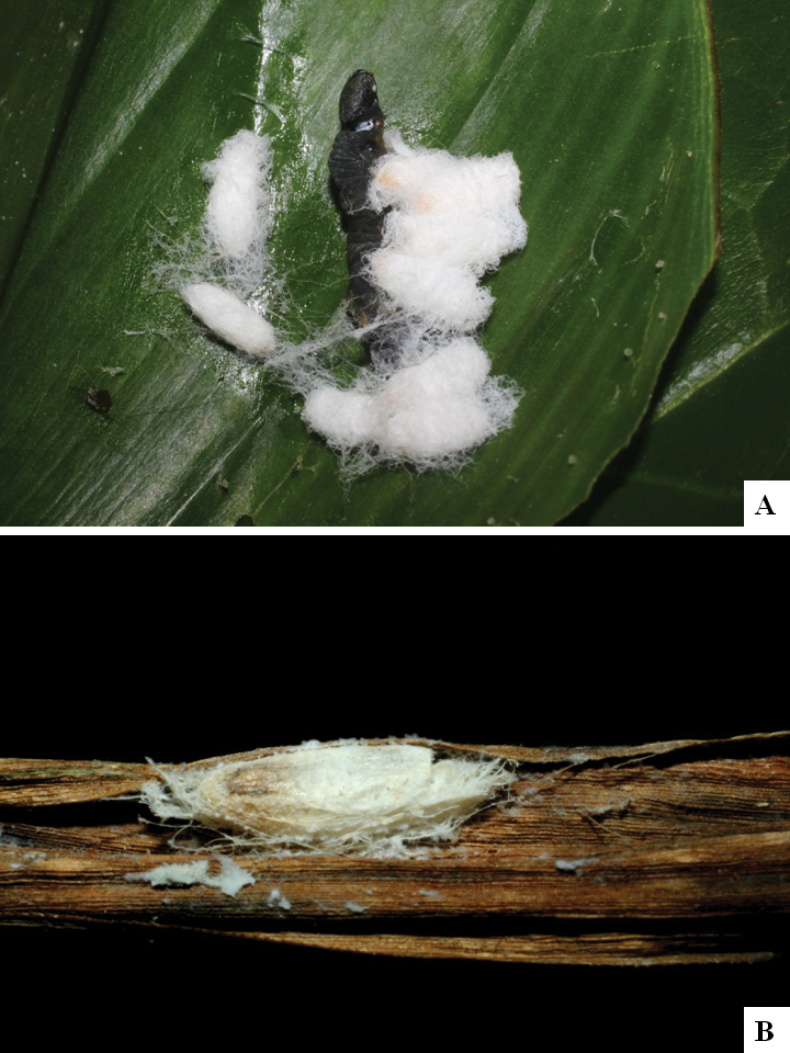
Parasite cocoons **A***Alphomelonricardocaleroi* 15-SRNP-55879-DHJ492398 **B***Alphomelonsergioriosi* 08-SRNP-32245-DHJ462411.

**Figure 103.**
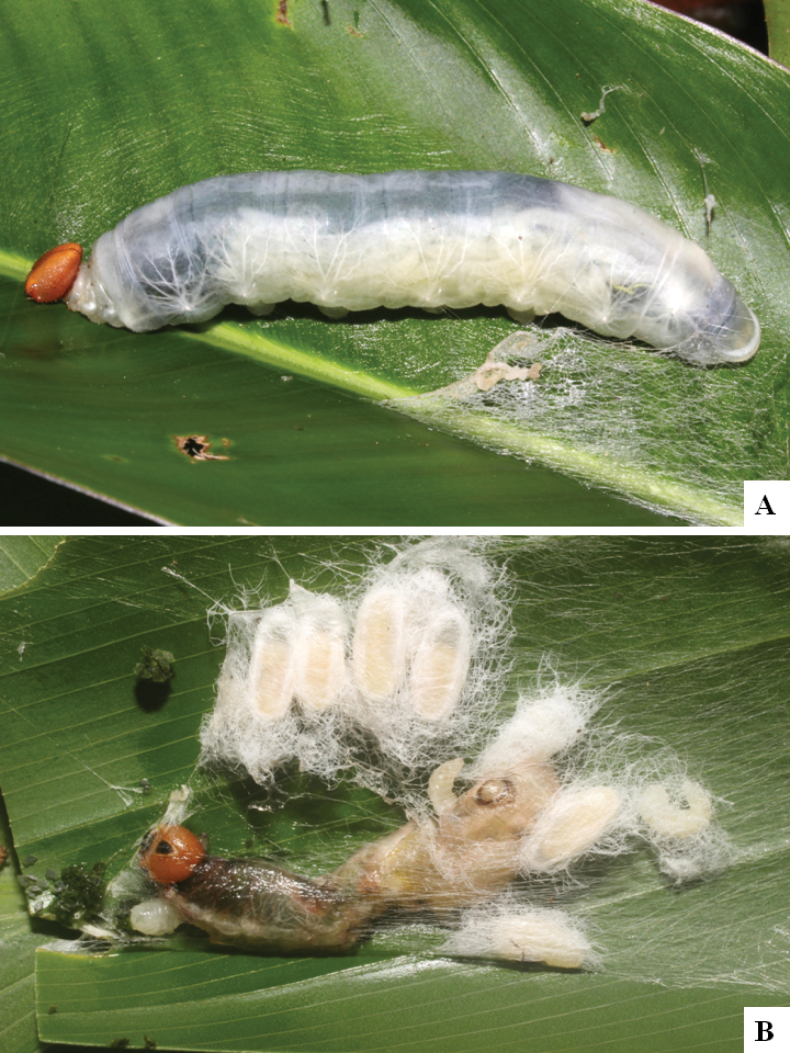
Parasite cocoons and caterpillars **A***Alphomelontalidicida* 17-SRNP-30054-DHJ735829 **B***Alphomelonxestopyga* 16-SRNP-55580-DHJ492508.

**Figure 104.**
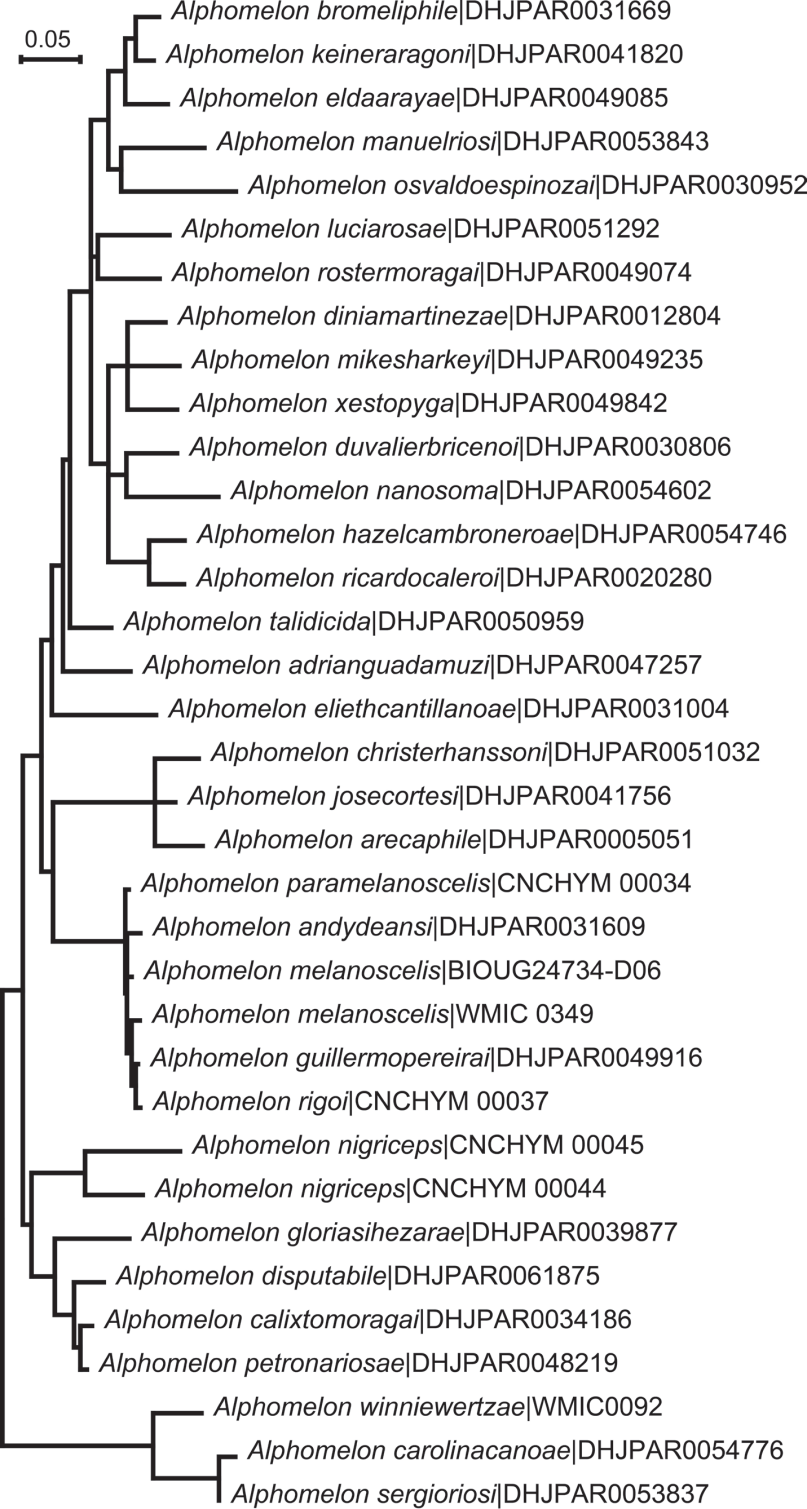
Maximum Likelihood analysis of all species of *Alphomelon* with full or almost full (> 500 base pairs) DNA barcodes. The tree was constructed based on the General Time Reversible model (Nei and Kumar 2000), made using MEGA X (Kumar et al. 2018). Tip labels include the accession for the sequence from the type series used to construct the tree.

**Figure 105.**
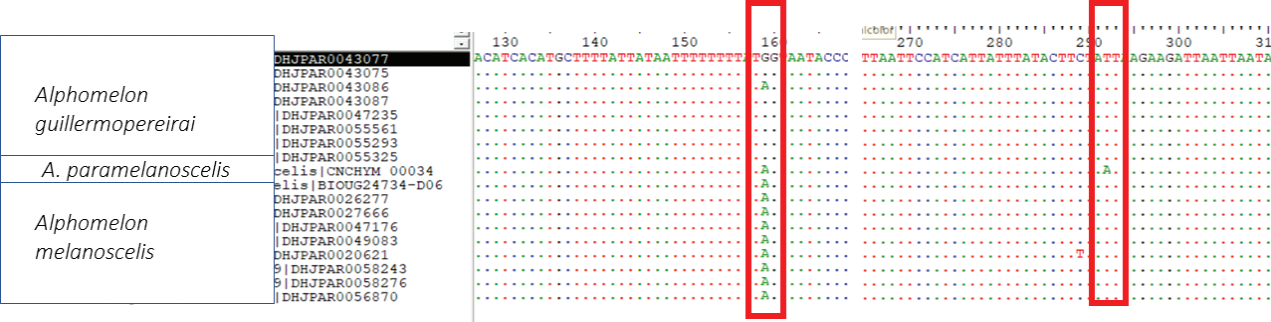
Diagnostic base pairs to differentiate species that fall within the same BIN. Red rectangles show two diagnostic base pairs (Single Nucleotide Polymorphisms) in specimens of *Alphomelonguillermopereirai*, *A.melanoscelis*, and *A.paramelanoscelis* within BINBOLD:AAB8584. Numbers were derived by aligning sequences to the cytochrome c oxidase subunit I (COI) region of the full mitochondrial reference genome of *Drosophilamelanogaster* (NC_024511.2).

## Supplementary Material

XML Treatment for Alphomelon adrianguadamuzi

XML Treatment for Alphomelon amazonas

XML Treatment for Alphomelon andydeansi

XML Treatment for Alphomelon arecaphile

XML Treatment for Alphomelon brachymacher

XML Treatment for Alphomelon brasiliensis

XML Treatment for Alphomelon bromeliphile

XML Treatment for Alphomelon calixtomoragai

XML Treatment for Alphomelon carolinacanoae

XML Treatment for Alphomelon christerhanssoni

XML Treatment for Alphomelon citroloma

XML Treatment for Alphomelon conforme

XML Treatment for Alphomelon crocostethus

XML Treatment for Alphomelon cruzi

XML Treatment for Alphomelon diniamartinezae

XML Treatment for Alphomelon disputabile

XML Treatment for Alphomelon duvalierbricenoi

XML Treatment for Alphomelon eldaarayae

XML Treatment for Alphomelon eliethcantillanoae

XML Treatment for Alphomelon gloriasihezarae

XML Treatment for Alphomelon guillermopereirai

XML Treatment for Alphomelon hazelcambroneroae

XML Treatment for Alphomelon itatiaiensis

XML Treatment for Alphomelon josecortesi

XML Treatment for Alphomelon keineraragoni

XML Treatment for Alphomelon luciarosae

XML Treatment for Alphomelon manuelriosi

XML Treatment for Alphomelon melanoscelis

XML Treatment for Alphomelon mikesharkeyi

XML Treatment for Alphomelon nanosoma

XML Treatment for Alphomelon nigriceps

XML Treatment for Alphomelon osvaldoespinozai

XML Treatment for Alphomelon palomae

XML Treatment for Alphomelon paramelanoscelis

XML Treatment for Alphomelon paranigriceps

XML Treatment for Alphomelon paurogenum

XML Treatment for Alphomelon petronariosae

XML Treatment for Alphomelon pyrrhogluteum

XML Treatment for Alphomelon rhyssocercus

XML Treatment for Alphomelon ricardocaleroi

XML Treatment for Alphomelon rigoi

XML Treatment for Alphomelon rostermoragai

XML Treatment for Alphomelon rugosus

XML Treatment for Alphomelon sergioriosi

XML Treatment for Alphomelon simpsonorum

XML Treatment for Alphomelon talidicida

XML Treatment for Alphomelon winniewertzae

XML Treatment for Alphomelon xestopyga

XML Treatment for Alphomelon yanayacu

## Footnotes

The four species in the couplets 36–39 are very similar morphologically and the characters presented here are rather subtle and subjective, but DNA barcodes discriminate among them.

## ﻿Additional information

### Author ORCIDs

Jose L. Fernandez-Triana https://orcid.org/0000-0003-0425-0309

Eduardo M. Shimbori https://orcid.org/0000-0003-4655-2591

James B. Whitfield https://orcid.org/0000-0002-3031-9106

Angelica M. Penteado-Dias https://orcid.org/0000-0002-8371-5591

Scott R. Shaw https://orcid.org/0000-0002-5024-4594

Caroline Boudreault https://orcid.org/0000-0002-4511-2626

P.D.N. Hebert https://orcid.org/0000-0002-3081-6700

M. Alex Smith https://orcid.org/0000-0002-8650-2575

Daniel H. Janzen https://orcid.org/0000-0002-7335-5107

### Data availability

All of the data that support the findings of this study are available in the main text or Supplementary Information.

## Supplementary materials

Supplementary material 1 Two NJ trees of *Alphomelon* sequences over 300 and 500 base pairs Data type DNA barcoding data https://binary.pensoft.net/file/893208This dataset is made available under the Open Database License (http://opendatacommons.org/licenses/odbl/1.0/). The Open Database License (ODbL) is a license agreement intended to allow users to freely share, modify, and use this Dataset while maintaining this same freedom for others, provided that the original source and author(s) are credited.Jose L. Fernandez-Triana, Eduardo M. Shimbori, James B. Whitfield, Angelica M. Penteado-Dias, Scott R. Shaw, Caroline Boudreault, Jayme Sones, Kate Perez, Allison Brown, Ramya Manjunath, John M. Burns, P. D. N. Hebert, M. Alex Smith, Winnifred Hallwachs, Daniel H. Janzen
