# Supplementary material for: ﻿A revision of the parasitoid wasp genus Alphomelon Mason with the description of 30 new species (Hymenoptera, Braconidae)
Source: Zookeys. 2023 Aug 16;1175:5–162. doi: 10.3897/zookeys.1175.105068 (PMC10448698; doi:10.3897/zookeys.1175.105068)
Supplement: Supplementary material 1 — Two NJ trees of Alphomelon sequences over 300 and 500 base pairs [file zookeys-1175-005_article-105068__-s001.pdf]

BOLD TaxonID Tree

Title : Tree Result - DS-ALPHOMEL (1295 records selected)
Date : 13-Apr-2023
Data Type : Nucleotide
Distance Model : Kimura 2 Parameter
Marker : COI-5P
Colourization : [blue]=Stop Codons [red]=Contamination or misidentification

Label : Sample ID
Label : Taxon
Label : Country
Label : Sequence Length
Label : Barcode Cluster (BIN)

Filter : length > 300bp only
Filter : exclude records flagged as misidentifications
Filter : exclude records with stop codons
Filter : exclude contaminants

Sequence Count : 1255
Species count : 36
Genus count : 1
Family count : 1
Unidentified : 17

BIN Count : 37

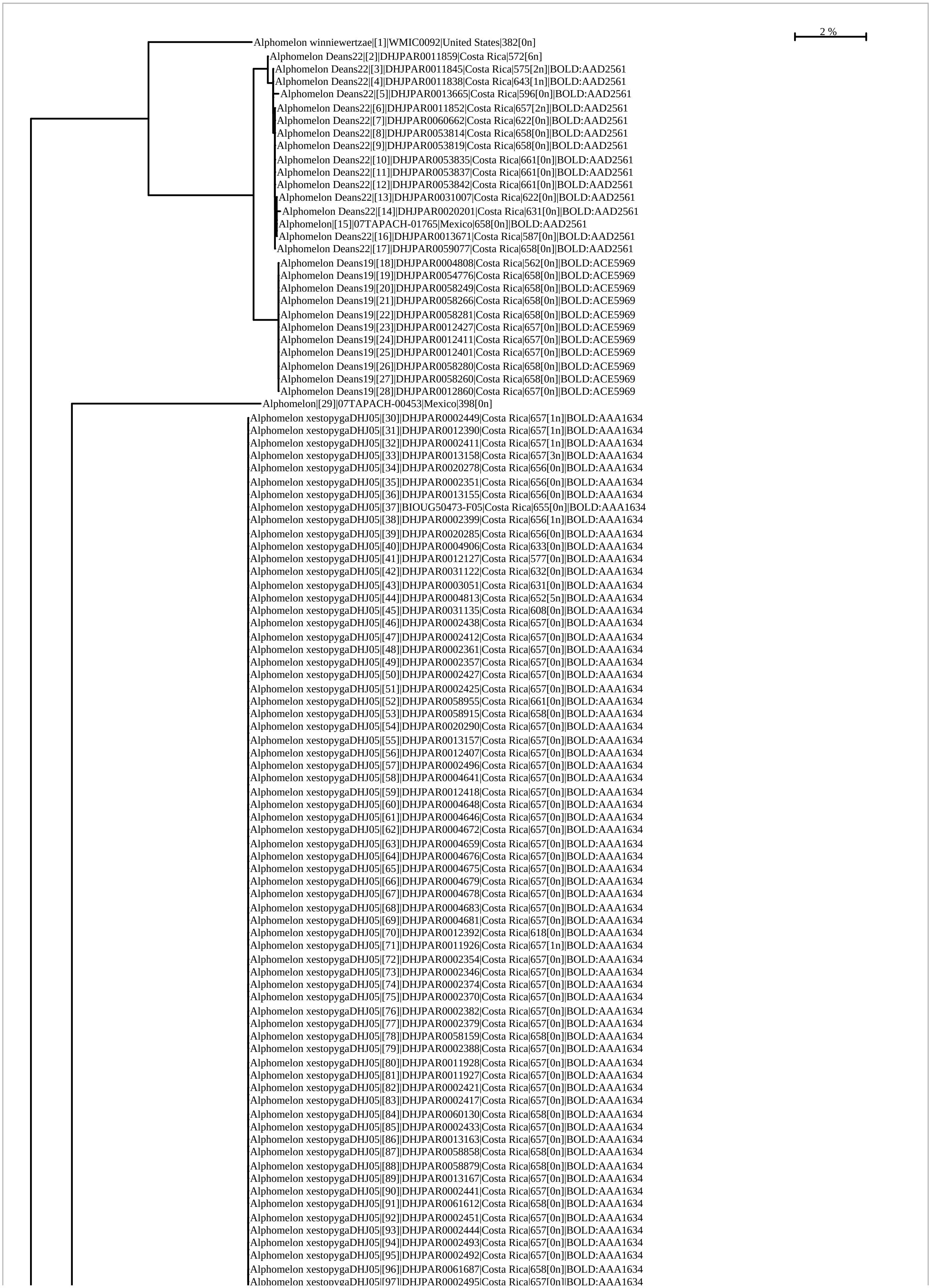
2 %
Alphomelon winniewertzae|[1]|WMIC0092|United States|382[0n]
Alphomelon Deans22|[2]|DHJPAR0011859|Costa Rica|572[6n]
Alphomelon Deans22|[3]|DHJPAR0011845|Costa Rica|575[2n]|BOLD:AAD2561
Alphomelon Deans22|[4]|DHJPAR0011838|Costa Rica|643[1n]|BOLD:AAD2561
Alphomelon Deans22|[5]|DHJPAR0013665|Costa Rica|596[0n]|BOLD:AAD2561
Alphomelon Deans22|[6]|DHJPAR0011852|Costa Rica|657[2n]|BOLD:AAD2561
Alphomelon Deans22|[7]|DHJPAR0060662|Costa Rica|622[0n]|BOLD:AAD2561
Alphomelon Deans22|[8]|DHJPAR0053814|Costa Rica|658[0n]|BOLD:AAD2561
Alphomelon Deans22|[9]|DHJPAR0053819|Costa Rica|658[0n]|BOLD:AAD2561
Alphomelon Deans22|[10]|DHJPAR0053835|Costa Rica|661[0n]|BOLD:AAD2561
Alphomelon Deans22|[11]|DHJPAR0053837|Costa Rica|661[0n]|BOLD:AAD2561
Alphomelon Deans22|[12]|DHJPAR0053842|Costa Rica|661[0n]|BOLD:AAD2561
Alphomelon Deans22|[13]|DHJPAR0031007|Costa Rica|622[0n]|BOLD:AAD2561
Alphomelon Deans22|[14]|DHJPAR0020201|Costa Rica|631[0n]|BOLD:AAD2561
Alphomelon|[15]|07TAPACH-01765|Mexico|658[0n]|BOLD:AAD2561
Alphomelon Deans22|[16]|DHJPAR0013671|Costa Rica|587[0n]|BOLD:AAD2561
Alphomelon Deans22|[17]|DHJPAR0059077|Costa Rica|658[0n]|BOLD:AAD2561
Alphomelon Deans19|[18]|DHJPAR0004808|Costa Rica|562[0n]|BOLD:ACE5969
Alphomelon Deans19|[19]|DHJPAR0054776|Costa Rica|658[0n]|BOLD:ACE5969
Alphomelon Deans19|[20]|DHJPAR0058249|Costa Rica|658[0n]|BOLD:ACE5969
Alphomelon Deans19|[21]|DHJPAR0058266|Costa Rica|658[0n]|BOLD:ACE5969
Alphomelon Deans19|[22]|DHJPAR0058281|Costa Rica|658[0n]|BOLD:ACE5969
Alphomelon Deans19|[23]|DHJPAR0012427|Costa Rica|657[0n]|BOLD:ACE5969
Alphomelon Deans19|[24]|DHJPAR0012411|Costa Rica|657[0n]|BOLD:ACE5969
Alphomelon Deans19|[25]|DHJPAR0012401|Costa Rica|657[0n]|BOLD:ACE5969
Alphomelon Deans19|[26]|DHJPAR0058280|Costa Rica|658[0n]|BOLD:ACE5969
Alphomelon Deans19|[27]|DHJPAR0058260|Costa Rica|658[0n]|BOLD:ACE5969
Alphomelon Deans19|[28]|DHJPAR0012860|Costa Rica|657[0n]|BOLD:ACE5969
Alphomelon|[29]|07TAPACH-00453|Mexico|398[0n]
Alphomelon xestopygaDHJ05|[30]|DHJPAR0002449|Costa Rica|657[1n]|BOLD:AAA1634
Alphomelon xestopygaDHJ05|[31]|DHJPAR0012390|Costa Rica|657[1n]|BOLD:AAA1634
Alphomelon xestopygaDHJ05|[32]|DHJPAR0002411|Costa Rica|657[1n]|BOLD:AAA1634
Alphomelon xestopygaDHJ05|[33]|DHJPAR0013158|Costa Rica|657[3n]|BOLD:AAA1634
Alphomelon xestopygaDHJ05|[34]|DHJPAR0020278|Costa Rica|656[0n]|BOLD:AAA1634
Alphomelon xestopygaDHJ05|[35]|DHJPAR0002351|Costa Rica|656[0n]|BOLD:AAA1634
Alphomelon xestopygaDHJ05|[36]|DHJPAR0013155|Costa Rica|656[0n]|BOLD:AAA1634
Alphomelon xestopygaDHJ05|[37]|BIOUG50473-F05|Costa Rica|655[0n]|BOLD:AAA1634
Alphomelon xestopygaDHJ05|[38]|DHJPAR0002399|Costa Rica|656[1n]|BOLD:AAA1634
Alphomelon xestopygaDHJ05|[39]|DHJPAR0020285|Costa Rica|656[0n]|BOLD:AAA1634
Alphomelon xestopygaDHJ05|[40]|DHJPAR0004906|Costa Rica|633[0n]|BOLD:AAA1634
Alphomelon xestopygaDHJ05|[41]|DHJPAR0012127|Costa Rica|577[0n]|BOLD:AAA1634
Alphomelon xestopygaDHJ05|[42]|DHJPAR0031122|Costa Rica|632[0n]|BOLD:AAA1634
Alphomelon xestopygaDHJ05|[43]|DHJPAR0003051|Costa Rica|631[0n]|BOLD:AAA1634
Alphomelon xestopygaDHJ05|[44]|DHJPAR0004813|Costa Rica|652[5n]|BOLD:AAA1634
Alphomelon xestopygaDHJ05|[45]|DHJPAR0031135|Costa Rica|608[0n]|BOLD:AAA1634
Alphomelon xestopygaDHJ05|[46]|DHJPAR0002438|Costa Rica|657[0n]|BOLD:AAA1634
Alphomelon xestopygaDHJ05|[47]|DHJPAR0002412|Costa Rica|657[0n]|BOLD:AAA1634
Alphomelon xestopygaDHJ05|[48]|DHJPAR0002361|Costa Rica|657[0n]|BOLD:AAA1634
Alphomelon xestopygaDHJ05|[49]|DHJPAR0002357|Costa Rica|657[0n]|BOLD:AAA1634
Alphomelon xestopygaDHJ05|[50]|DHJPAR0002427|Costa Rica|657[0n]|BOLD:AAA1634
Alphomelon xestopygaDHJ05|[51]|DHJPAR0002425|Costa Rica|657[0n]|BOLD:AAA1634
Alphomelon xestopygaDHJ05|[52]|DHJPAR0058955|Costa Rica|661[0n]|BOLD:AAA1634
Alphomelon xestopygaDHJ05|[53]|DHJPAR0058915|Costa Rica|658[0n]|BOLD:AAA1634
Alphomelon xestopygaDHJ05|[54]|DHJPAR0020290|Costa Rica|657[0n]|BOLD:AAA1634
Alphomelon xestopygaDHJ05|[55]|DHJPAR0013157|Costa Rica|657[0n]|BOLD:AAA1634
Alphomelon xestopygaDHJ05|[56]|DHJPAR0012407|Costa Rica|657[0n]|BOLD:AAA1634
Alphomelon xestopygaDHJ05|[57]|DHJPAR0002496|Costa Rica|657[0n]|BOLD:AAA1634
Alphomelon xestopygaDHJ05|[58]|DHJPAR0004641|Costa Rica|657[0n]|BOLD:AAA1634
Alphomelon xestopygaDHJ05|[59]|DHJPAR0012418|Costa Rica|657[0n]|BOLD:AAA1634
Alphomelon xestopygaDHJ05|[60]|DHJPAR0004648|Costa Rica|657[0n]|BOLD:AAA1634
Alphomelon xestopygaDHJ05|[61]|DHJPAR0004646|Costa Rica|657[0n]|BOLD:AAA1634
Alphomelon xestopygaDHJ05|[62]|DHJPAR0004672|Costa Rica|657[0n]|BOLD:AAA1634
Alphomelon xestopygaDHJ05|[63]|DHJPAR0004659|Costa Rica|657[0n]|BOLD:AAA1634
Alphomelon xestopygaDHJ05|[64]|DHJPAR0004676|Costa Rica|657[0n]|BOLD:AAA1634
Alphomelon xestopygaDHJ05|[65]|DHJPAR0004675|Costa Rica|657[0n]|BOLD:AAA1634
Alphomelon xestopygaDHJ05|[66]|DHJPAR0004679|Costa Rica|657[0n]|BOLD:AAA1634
Alphomelon xestopygaDHJ05|[67]|DHJPAR0004678|Costa Rica|657[0n]|BOLD:AAA1634
Alphomelon xestopygaDHJ05|[68]|DHJPAR0004683|Costa Rica|657[0n]|BOLD:AAA1634
Alphomelon xestopygaDHJ05|[69]|DHJPAR0004681|Costa Rica|657[0n]|BOLD:AAA1634
Alphomelon xestopygaDHJ05|[70]|DHJPAR0012392|Costa Rica|618[0n]|BOLD:AAA1634
Alphomelon xestopygaDHJ05|[71]|DHJPAR0011926|Costa Rica|657[1n]|BOLD:AAA1634
Alphomelon xestopygaDHJ05|[72]|DHJPAR0002354|Costa Rica|657[0n]|BOLD:AAA1634
Alphomelon xestopygaDHJ05|[73]|DHJPAR0002346|Costa Rica|657[0n]|BOLD:AAA1634
Alphomelon xestopygaDHJ05|[74]|DHJPAR0002374|Costa Rica|657[0n]|BOLD:AAA1634
Alphomelon xestopygaDHJ05|[75]|DHJPAR0002370|Costa Rica|657[0n]|BOLD:AAA1634
Alphomelon xestopygaDHJ05|[76]|DHJPAR0002382|Costa Rica|657[0n]|BOLD:AAA1634
Alphomelon xestopygaDHJ05|[77]|DHJPAR0002379|Costa Rica|657[0n]|BOLD:AAA1634
Alphomelon xestopygaDHJ05|[78]|DHJPAR0058159|Costa Rica|658[0n]|BOLD:AAA1634
Alphomelon xestopygaDHJ05|[79]|DHJPAR0002388|Costa Rica|657[0n]|BOLD:AAA1634
Alphomelon xestopygaDHJ05|[80]|DHJPAR0011928|Costa Rica|657[0n]|BOLD:AAA1634
Alphomelon xestopygaDHJ05|[81]|DHJPAR0011927|Costa Rica|657[0n]|BOLD:AAA1634
Alphomelon xestopygaDHJ05|[82]|DHJPAR0002421|Costa Rica|657[0n]|BOLD:AAA1634
Alphomelon xestopygaDHJ05|[83]|DHJPAR0002417|Costa Rica|657[0n]|BOLD:AAA1634
Alphomelon xestopygaDHJ05|[84]|DHJPAR0060130|Costa Rica|658[0n]|BOLD:AAA1634
Alphomelon xestopygaDHJ05|[85]|DHJPAR0002433|Costa Rica|657[0n]|BOLD:AAA1634
Alphomelon xestopygaDHJ05|[86]|DHJPAR0013163|Costa Rica|657[0n]|BOLD:AAA1634
Alphomelon xestopygaDHJ05|[87]|DHJPAR0058858|Costa Rica|658[0n]|BOLD:AAA1634
Alphomelon xestopygaDHJ05|[88]|DHJPAR0058879|Costa Rica|658[0n]|BOLD:AAA1634
Alphomelon xestopygaDHJ05|[89]|DHJPAR0013167|Costa Rica|657[0n]|BOLD:AAA1634
Alphomelon xestopygaDHJ05|[90]|DHJPAR0002441|Costa Rica|657[0n]|BOLD:AAA1634
Alphomelon xestopygaDHJ05|[91]|DHJPAR0061612|Costa Rica|658[0n]|BOLD:AAA1634
Alphomelon xestopygaDHJ05|[92]|DHJPAR0002451|Costa Rica|657[0n]|BOLD:AAA1634
Alphomelon xestopygaDHJ05|[93]|DHJPAR0002444|Costa Rica|657[0n]|BOLD:AAA1634
Alphomelon xestopygaDHJ05|[94]|DHJPAR0002493|Costa Rica|657[0n]|BOLD:AAA1634
Alphomelon xestopygaDHJ05|[95]|DHJPAR0002492|Costa Rica|657[0n]|BOLD:AAA1634
Alphomelon xestopygaDHJ05|[96]|DHJPAR0061687|Costa Rica|658[0n]|BOLD:AAA1634
Alphomelon xestopygaDHJ05|[97]|DHJPAR0002495|Costa Rica|657[0n]|BOLD:AAA1634

Alphomelon xestopygaDHJ05|[95]|DHJPAR0002492|Costa Rica|657[0n]|BOLD:AAA1634
Alphomelon xestopygaDHJ05|[96]|DHJPAR0061687|Costa Rica|658[0n]|BOLD:AAA1634
Alphomelon xestopygaDHJ05|[97]|DHJPAR0002495|Costa Rica|657[0n]|BOLD:AAA1634
Alphomelon xestopygaDHJ05|[98]|DHJPAR0012123|Costa Rica|657[0n]|BOLD:AAA1634
Alphomelon xestopygaDHJ05|[99]|DHJPAR0058158|Costa Rica|658[0n]|BOLD:AAA1634
Alphomelon xestopygaDHJ05|[100]|DHJPAR0058183|Costa Rica|658[0n]|BOLD:AAA1634
Alphomelon xestopygaDHJ05|[101]|DHJPAR0058176|Costa Rica|658[0n]|BOLD:AAA1634
Alphomelon xestopygaDHJ05|[102]|DHJPAR0002347|Costa Rica|657[0n]|BOLD:AAA1634
Alphomelon xestopygaDHJ05|[103]|DHJPAR0059765|Costa Rica|658[0n]|BOLD:AAA1634
Alphomelon xestopygaDHJ05|[104]|DHJPAR0002344|Costa Rica|657[0n]|BOLD:AAA1634
Alphomelon xestopygaDHJ05|[105]|DHJPAR0002343|Costa Rica|657[0n]|BOLD:AAA1634
Alphomelon xestopygaDHJ05|[106]|DHJPAR0002349|Costa Rica|657[0n]|BOLD:AAA1634
Alphomelon xestopygaDHJ05|[107]|DHJPAR0012126|Costa Rica|657[0n]|BOLD:AAA1634
Alphomelon xestopygaDHJ05|[108]|DHJPAR0058230|Costa Rica|658[0n]|BOLD:AAA1634
Alphomelon xestopygaDHJ05|[109]|DHJPAR0013168|Costa Rica|657[0n]|BOLD:AAA1634
Alphomelon xestopygaDHJ05|[110]|DHJPAR0059744|Costa Rica|658[0n]|BOLD:AAA1634
Alphomelon xestopygaDHJ05|[111]|DHJPAR0059725|Costa Rica|658[0n]|BOLD:AAA1634
Alphomelon xestopygaDHJ05|[112]|DHJPAR0002494|Costa Rica|657[0n]|BOLD:AAA1634
Alphomelon xestopygaDHJ05|[113]|DHJPAR0059756|Costa Rica|658[0n]|BOLD:AAA1634
Alphomelon xestopygaDHJ05|[114]|DHJPAR0004953|Costa Rica|657[0n]|BOLD:AAA1634
Alphomelon xestopygaDHJ05|[115]|DHJPAR0062607|Costa Rica|658[0n]|BOLD:AAA1634
Alphomelon xestopygaDHJ05|[116]|DHJPAR0031094|Costa Rica|658[0n]|BOLD:AAA1634
Alphomelon xestopygaDHJ05|[117]|DHJPAR0004964|Costa Rica|657[0n]|BOLD:AAA1634
Alphomelon xestopygaDHJ05|[118]|DHJPAR0031109|Costa Rica|658[0n]|BOLD:AAA1634
Alphomelon xestopygaDHJ05|[119]|DHJPAR0031099|Costa Rica|658[0n]|BOLD:AAA1634
Alphomelon xestopygaDHJ05|[120]|DHJPAR0005024|Costa Rica|657[0n]|BOLD:AAA1634
Alphomelon xestopygaDHJ05|[121]|DHJPAR0002359|Costa Rica|657[0n]|BOLD:AAA1634
Alphomelon xestopygaDHJ05|[122]|DHJPAR0005043|Costa Rica|657[0n]|BOLD:AAA1634
Alphomelon xestopygaDHJ05|[123]|DHJPAR0005036|Costa Rica|657[0n]|BOLD:AAA1634
Alphomelon xestopygaDHJ05|[124]|DHJPAR0012398|Costa Rica|657[0n]|BOLD:AAA1634
Alphomelon xestopygaDHJ05|[125]|DHJPAR0011923|Costa Rica|657[0n]|BOLD:AAA1634
Alphomelon xestopygaDHJ05|[126]|DHJPAR0030897|Costa Rica|657[0n]|BOLD:AAA1634
Alphomelon xestopygaDHJ05|[127]|DHJPAR0005037|Costa Rica|657[0n]|BOLD:AAA1634
Alphomelon xestopygaDHJ05|[128]|DHJPAR0056537|Costa Rica|658[0n]|BOLD:AAA1634
Alphomelon xestopygaDHJ05|[129]|DHJPAR0002408|Costa Rica|657[0n]|BOLD:AAA1634
Alphomelon xestopygaDHJ05|[130]|DHJPAR0002355|Costa Rica|657[0n]|BOLD:AAA1634
Alphomelon xestopygaDHJ05|[131]|DHJPAR0059468|Costa Rica|658[0n]|BOLD:AAA1634
Alphomelon xestopygaDHJ05|[132]|DHJPAR0002367|Costa Rica|657[1n]|BOLD:AAA1634
Alphomelon xestopygaDHJ05|[133]|DHJPAR0005031|Costa Rica|657[1n]|BOLD:AAA1634
Alphomelon xestopygaDHJ05|[134]|DHJPAR0020286|Costa Rica|657[0n]|BOLD:AAA1634
Alphomelon xestopygaDHJ05|[135]|DHJPAR0051223|Costa Rica|658[0n]|BOLD:AAA1634
Alphomelon xestopygaDHJ05|[136]|DHJPAR0031735|Costa Rica|658[0n]|BOLD:AAA1634
Alphomelon xestopygaDHJ05|[137]|DHJPAR0013165|Costa Rica|657[0n]|BOLD:AAA1634
Alphomelon xestopygaDHJ05|[138]|DHJPAR0002424|Costa Rica|657[0n]|BOLD:AAA1634
Alphomelon xestopygaDHJ05|[139]|DHJPAR0059766|Costa Rica|658[0n]|BOLD:AAA1634
Alphomelon xestopygaDHJ05|[140]|DHJPAR0002368|Costa Rica|657[2n]|BOLD:AAA1634
Alphomelon xestopygaDHJ05|[141]|DHJPAR0031620|Costa Rica|658[0n]|BOLD:AAA1634
Alphomelon xestopygaDHJ05|[142]|DHJPAR0031112|Costa Rica|658[0n]|BOLD:AAA1634
Alphomelon xestopygaDHJ05|[143]|DHJPAR0002401|Costa Rica|657[0n]|BOLD:AAA1634
Alphomelon xestopygaDHJ05|[144]|DHJPAR0002416|Costa Rica|657[0n]|BOLD:AAA1634
Alphomelon xestopygaDHJ05|[145]|DHJPAR0002363|Costa Rica|657[0n]|BOLD:AAA1634
Alphomelon xestopygaDHJ05|[146]|DHJPAR0012129|Costa Rica|621[0n]|BOLD:AAA1634
Alphomelon xestopygaDHJ05|[147]|DHJPAR0002489|Costa Rica|615[6n]|BOLD:AAA1634
Alphomelon xestopygaDHJ05|[148]|DHJPAR0012862|Costa Rica|657[0n]|BOLD:AAA1634
Alphomelon xestopygaDHJ05|[149]|DHJPAR0058881|Costa Rica|658[0n]|BOLD:AAA1634
Alphomelon xestopygaDHJ05|[150]|DHJPAR0002400|Costa Rica|657[0n]|BOLD:AAA1634
Alphomelon xestopygaDHJ05|[151]|DHJPAR0030922|Costa Rica|657[0n]|BOLD:AAA1634
Alphomelon xestopygaDHJ05|[152]|DHJPAR0002405|Costa Rica|657[0n]|BOLD:AAA1634
Alphomelon xestopygaDHJ05|[153]|DHJPAR0012403|Costa Rica|657[0n]|BOLD:AAA1634
Alphomelon xestopygaDHJ05|[154]|DHJPAR0020292|Costa Rica|656[0n]|BOLD:AAA1634
Alphomelon xestopygaDHJ05|[155]|DHJPAR0020289|Costa Rica|656[0n]|BOLD:AAA1634
Alphomelon xestopygaDHJ05|[156]|DHJPAR0012406|Costa Rica|657[0n]|BOLD:AAA1634
Alphomelon xestopygaDHJ05|[157]|DHJPAR0058184|Costa Rica|658[1n]|BOLD:AAA1634
Alphomelon xestopygaDHJ05|[158]|DHJPAR0002387|Costa Rica|657[1n]|BOLD:AAA1634
Alphomelon xestopygaDHJ05|[159]|DHJPAR0002372|Costa Rica|657[0n]|BOLD:AAA1634
Alphomelon xestopygaDHJ05|[160]|DHJPAR0002366|Costa Rica|657[0n]|BOLD:AAA1634
Alphomelon xestopygaDHJ05|[161]|DHJPAR0002380|Costa Rica|657[0n]|BOLD:AAA1634
Alphomelon xestopygaDHJ05|[162]|DHJPAR0002376|Costa Rica|657[0n]|BOLD:AAA1634
Alphomelon xestopygaDHJ05|[163]|DHJPAR0002384|Costa Rica|657[0n]|BOLD:AAA1634
Alphomelon xestopygaDHJ05|[164]|DHJPAR0002383|Costa Rica|657[0n]|BOLD:AAA1634
Alphomelon xestopygaDHJ05|[165]|DHJPAR0002389|Costa Rica|657[0n]|BOLD:AAA1634
Alphomelon xestopygaDHJ05|[166]|DHJPAR0002386|Costa Rica|657[0n]|BOLD:AAA1634
Alphomelon xestopygaDHJ05|[167]|DHJPAR0002420|Costa Rica|657[0n]|BOLD:AAA1634
Alphomelon xestopygaDHJ05|[168]|DHJPAR0002419|Costa Rica|657[0n]|BOLD:AAA1634
Alphomelon xestopygaDHJ05|[169]|DHJPAR0053789|Costa Rica|658[0n]|BOLD:AAA1634
Alphomelon xestopygaDHJ05|[170]|DHJPAR0002432|Costa Rica|657[0n]|BOLD:AAA1634
Alphomelon xestopygaDHJ05|[171]|DHJPAR0013159|Costa Rica|657[0n]|BOLD:AAA1634
Alphomelon xestopygaDHJ05|[172]|DHJPAR0013164|Costa Rica|657[0n]|BOLD:AAA1634
Alphomelon xestopygaDHJ05|[173]|DHJPAR0060190|Costa Rica|658[0n]|BOLD:AAA1634
Alphomelon xestopygaDHJ05|[174]|DHJPAR0053751|Costa Rica|658[0n]|BOLD:AAA1634
Alphomelon xestopygaDHJ05|[175]|DHJPAR0053758|Costa Rica|658[0n]|BOLD:AAA1634
Alphomelon xestopygaDHJ05|[176]|DHJPAR0013156|Costa Rica|657[0n]|BOLD:AAA1634
Alphomelon xestopygaDHJ05|[177]|DHJPAR0020283|Costa Rica|657[0n]|BOLD:AAA1634
Alphomelon xestopygaDHJ05|[178]|DHJPAR0020279|Costa Rica|657[0n]|BOLD:AAA1634
Alphomelon xestopygaDHJ05|[179]|DHJPAR0012393|Costa Rica|657[0n]|BOLD:AAA1634
Alphomelon xestopygaDHJ05|[180]|DHJPAR0012391|Costa Rica|657[0n]|BOLD:AAA1634
Alphomelon xestopygaDHJ05|[181]|DHJPAR0012415|Costa Rica|657[0n]|BOLD:AAA1634
Alphomelon xestopygaDHJ05|[182]|DHJPAR0012405|Costa Rica|657[0n]|BOLD:AAA1634
Alphomelon xestopygaDHJ05|[183]|DHJPAR0004645|Costa Rica|657[0n]|BOLD:AAA1634
Alphomelon xestopygaDHJ05|[184]|DHJPAR0004644|Costa Rica|657[0n]|BOLD:AAA1634
Alphomelon xestopygaDHJ05|[185]|DHJPAR0004652|Costa Rica|657[0n]|BOLD:AAA1634
Alphomelon xestopygaDHJ05|[186]|DHJPAR0004651|Costa Rica|657[0n]|BOLD:AAA1634
Alphomelon xestopygaDHJ05|[187]|DHJPAR0004661|Costa Rica|657[0n]|BOLD:AAA1634
Alphomelon xestopygaDHJ05|[188]|DHJPAR0004656|Costa Rica|657[0n]|BOLD:AAA1634
Alphomelon xestopygaDHJ05|[189]|DHJPAR0004663|Costa Rica|656[0n]|BOLD:AAA1634
Alphomelon xestopygaDHJ05|[190]|DHJPAR0004662|Costa Rica|657[0n]|BOLD:AAA1634
Alphomelon xestopygaDHJ05|[191]|DHJPAR0004673|Costa Rica|657[0n]|BOLD:AAA1634
Alphomelon xestopygaDHJ05|[192]|DHJPAR0004665|Costa Rica|657[0n]|BOLD:AAA1634
Alphomelon xestopygaDHJ05|[193]|DHJPAR0004682|Costa Rica|657[0n]|BOLD:AAA1634
Alphomelon xestopygaDHJ05|[194]|DHJPAR0004677|Costa Rica|657[0n]|BOLD:AAA1634

Alphomelon xestopygaDHJ05|[191]|DHJPAR000467[illegible]|Costa Rica|657[0n]|BOLD:AAA1634
Alphomelon xestopygaDHJ05|[192]|DHJPAR0004665|Costa Rica|657[0n]|BOLD:AAA1634
Alphomelon xestopygaDHJ05|[193]|DHJPAR0004682|Costa Rica|657[0n]|BOLD:AAA1634
Alphomelon xestopygaDHJ05|[194]|DHJPAR0004677|Costa Rica|657[0n]|BOLD:AAA1634
Alphomelon xestopygaDHJ05|[195]|DHJPAR0058169|Costa Rica|658[0n]|BOLD:AAA1634
Alphomelon xestopygaDHJ05|[196]|DHJPAR0002437|Costa Rica|657[0n]|BOLD:AAA1634
Alphomelon xestopygaDHJ05|[197]|DHJPAR0012122|Costa Rica|657[0n]|BOLD:AAA1634
Alphomelon xestopygaDHJ05|[198]|DHJPAR0012119|Costa Rica|657[0n]|BOLD:AAA1634
Alphomelon xestopygaDHJ05|[199]|DHJPAR0058229|Costa Rica|658[0n]|BOLD:AAA1634
Alphomelon xestopygaDHJ05|[200]|DHJPAR0049842|Costa Rica|658[0n]|BOLD:AAA1634
Alphomelon xestopygaDHJ05|[201]|DHJPAR0058210|Costa Rica|658[0n]|BOLD:AAA1634
Alphomelon xestopygaDHJ05|[202]|DHJPAR0031006|Costa Rica|658[0n]|BOLD:AAA1634
Alphomelon xestopygaDHJ05|[203]|DHJPAR0002431|Costa Rica|657[0n]|BOLD:AAA1634
Alphomelon xestopygaDHJ05|[204]|DHJPAR0002429|Costa Rica|657[0n]|BOLD:AAA1634
Alphomelon xestopygaDHJ05|[205]|DHJPAR0002439|Costa Rica|657[0n]|BOLD:AAA1634
Alphomelon xestopygaDHJ05|[206]|DHJPAR0058220|Costa Rica|658[0n]|BOLD:AAA1634
Alphomelon xestopygaDHJ05|[207]|DHJPAR0060609|Costa Rica|658[0n]|BOLD:AAA1634
Alphomelon xestopygaDHJ05|[208]|DHJPAR0005044|Costa Rica|657[0n]|BOLD:AAA1634
Alphomelon xestopygaDHJ05|[209]|DHJPAR0005059|Costa Rica|657[0n]|BOLD:AAA1634
Alphomelon xestopygaDHJ05|[210]|DHJPAR0002430|Costa Rica|657[0n]|BOLD:AAA1634
Alphomelon xestopygaDHJ05|[211]|DHJPAR0031110|Costa Rica|658[0n]|BOLD:AAA1634
Alphomelon xestopygaDHJ05|[212]|DHJPAR0031133|Costa Rica|658[0n]|BOLD:AAA1634
Alphomelon xestopygaDHJ05|[213]|DHJPAR0002364|Costa Rica|657[0n]|BOLD:AAA1634
Alphomelon xestopygaDHJ05|[214]|DHJPAR0002342|Costa Rica|657[0n]|BOLD:AAA1634
Alphomelon xestopygaDHJ05|[215]|DHJPAR0060602|Costa Rica|658[0n]|BOLD:AAA1634
Alphomelon xestopygaDHJ05|[216]|DHJPAR0002371|Costa Rica|657[0n]|BOLD:AAA1634
Alphomelon xestopygaDHJ05|[217]|DHJPAR0059740|Costa Rica|658[0n]|BOLD:AAA1634
Alphomelon xestopygaDHJ05|[218]|DHJPAR0013170|Costa Rica|657[1n]|BOLD:AAA1634
Alphomelon xestopygaDHJ05|[219]|DHJPAR0002358|Costa Rica|657[0n]|BOLD:AAA1634
Alphomelon xestopygaDHJ05|[220]|DHJPAR0002375|Costa Rica|657[0n]|BOLD:AAA1634
Alphomelon xestopygaDHJ05|[221]|DHJPAR0013153|Costa Rica|657[0n]|BOLD:AAA1634
Alphomelon xestopygaDHJ05|[222]|DHJPAR0013172|Costa Rica|657[0n]|BOLD:AAA1634
Alphomelon xestopygaDHJ05|[223]|DHJPAR0002407|Costa Rica|657[0n]|BOLD:AAA1634
Alphomelon xestopygaDHJ05|[224]|DHJPAR0002406|Costa Rica|657[0n]|BOLD:AAA1634
Alphomelon xestopygaDHJ05|[225]|DHJPAR0004933|Costa Rica|657[0n]|BOLD:AAA1634
Alphomelon xestopygaDHJ05|[226]|DHJPAR0002410|Costa Rica|657[1n]|BOLD:AAA1634
Alphomelon xestopygaDHJ05|[227]|DHJPAR0031125|Costa Rica|658[0n]|BOLD:AAA1634
Alphomelon xestopygaDHJ05|[228]|DHJPAR0031123|Costa Rica|658[0n]|BOLD:AAA1634
Alphomelon xestopygaDHJ05|[229]|DHJPAR0012703|Costa Rica|657[0n]|BOLD:AAA1634
Alphomelon xestopygaDHJ05|[230]|DHJPAR0002415|Costa Rica|657[0n]|BOLD:AAA1634
Alphomelon xestopygaDHJ05|[231]|DHJPAR0031134|Costa Rica|658[0n]|BOLD:AAA1634
Alphomelon xestopygaDHJ05|[232]|DHJPAR0031127|Costa Rica|658[0n]|BOLD:AAA1634
Alphomelon xestopygaDHJ05|[233]|DHJPAR0060596|Costa Rica|658[0n]|BOLD:AAA1634
Alphomelon xestopygaDHJ05|[234]|DHJPAR0030697|Costa Rica|658[0n]|BOLD:AAA1634
Alphomelon xestopygaDHJ05|[235]|DHJPAR0013166|Costa Rica|657[2n]|BOLD:AAA1634
Alphomelon xestopygaDHJ05|[236]|DHJPAR0060597|Costa Rica|658[0n]|BOLD:AAA1634
Alphomelon xestopygaDHJ05|[237]|DHJPAR0002428|Costa Rica|657[0n]|BOLD:AAA1634
Alphomelon xestopygaDHJ05|[238]|DHJPAR0002350|Costa Rica|657[1n]|BOLD:AAA1634
Alphomelon xestopygaDHJ05|[239]|DHJPAR0004931|Costa Rica|657[1n]|BOLD:AAA1634
Alphomelon xestopygaDHJ05|[240]|DHJPAR0002391|Costa Rica|657[1n]|BOLD:AAA1634
Alphomelon xestopygaDHJ05|[241]|DHJPAR0004897|Costa Rica|657[1n]|BOLD:AAA1634
Alphomelon xestopygaDHJ05|[242]|DHJPAR0004946|Costa Rica|657[1n]|BOLD:AAA1634
Alphomelon xestopygaDHJ05|[243]|DHJPAR0005022|Costa Rica|657[1n]|BOLD:AAA1634
Alphomelon xestopygaDHJ05|[244]|DHJPAR0002409|Costa Rica|657[1n]|BOLD:AAA1634
Alphomelon xestopygaDHJ05|[245]|DHJPAR0013162|Costa Rica|657[2n]|BOLD:AAA1634
Alphomelon xestopygaDHJ05|[246]|DHJPAR0002365|Costa Rica|657[1n]|BOLD:AAA1634
Alphomelon xestopygaDHJ05|[247]|DHJPAR0004900|Costa Rica|657[5n]|BOLD:AAA1634
Alphomelon xestopygaDHJ05|[248]|DHJPAR0013160|Costa Rica|655[3n]|BOLD:AAA1634
Alphomelon xestopygaDHJ05|[249]|DHJPAR0002418|Costa Rica|657[4n]|BOLD:AAA1634
Alphomelon xestopygaDHJ05|[250]|DHJPAR0003013|Costa Rica|627[0n]|BOLD:AAA1634
Alphomelon xestopygaDHJ05|[251]|DHJPAR0004650|Costa Rica|657[26n]|BOLD:AAA1634
Alphomelon xestopygaDHJ05|[252]|BIOUG09826-D03|Costa Rica|587[0n]|BOLD:AAA1634
Alphomelon xestopygaDHJ05|[253]|DHJPAR0002487|Costa Rica|303[0n]|BOLD:AAA1634
Alphomelon xestopygaDHJ05|[254]|DHJPAR0002436|Costa Rica|570[39n]
Alphomelon xestopygaDHJ05|[255]|DHJPAR0002352|Costa Rica|631[7n]
Alphomelon xestopygaDHJ05|[256]|DHJPAR0002373|Costa Rica|613[1n]|BOLD:AAA1634
Alphomelon xestopygaDHJ05|[257]|DHJPAR0002491|Costa Rica|561[6n]|BOLD:AAA1634
Alphomelon xestopygaDHJ05|[258]|DHJPAR0002398|Costa Rica|657[0n]|BOLD:AAA1634
Alphomelon xestopygaDHJ05|[259]|DHJPAR0002381|Costa Rica|657[0n]|BOLD:AAA1634
Alphomelon xestopygaDHJ05|[260]|DHJPAR0002356|Costa Rica|657[0n]|BOLD:AAA1634
Alphomelon xestopygaDHJ05|[261]|DHJPAR0002362|Costa Rica|657[0n]|BOLD:AAA1634
Alphomelon xestopygaDHJ05|[262]|DHJPAR0002353|Costa Rica|657[0n]|BOLD:AAA1634
Alphomelon xestopygaDHJ05|[263]|DHJPAR0011846|Costa Rica|657[0n]|BOLD:AAA1634
Alphomelon xestopygaDHJ05|[264]|DHJPAR0053760|Costa Rica|658[0n]|BOLD:AAA1634
Alphomelon xestopygaDHJ05|[265]|DHJPAR0004959|Costa Rica|657[3n]|BOLD:AAA1634
Alphomelon xestopygaDHJ05|[266]|DHJPAR0002403|Costa Rica|657[0n]|BOLD:AAA1634
Alphomelon xestopygaDHJ05|[267]|DHJPAR0002461|Costa Rica|657[0n]|BOLD:AAA1634
Alphomelon xestopygaDHJ05|[268]|DHJPAR0058880|Costa Rica|658[0n]|BOLD:AAA1634
Alphomelon xestopygaDHJ05|[269]|DHJPAR0058903|Costa Rica|658[0n]|BOLD:AAA1634
Alphomelon xestopygaDHJ05|[270]|DHJPAR0058930|Costa Rica|658[0n]|BOLD:AAA1634
Alphomelon xestopygaDHJ05|[271]|DHJPAR0058940|Costa Rica|658[0n]|BOLD:AAA1634
Alphomelon xestopygaDHJ05|[272]|DHJPAR0053767|Costa Rica|658[0n]|BOLD:AAA1634
Alphomelon xestopygaDHJ05|[273]|DHJPAR0052957|Costa Rica|658[0n]|BOLD:AAA1634
Alphomelon xestopygaDHJ05|[274]|DHJPAR0004902|Costa Rica|657[0n]|BOLD:AAA1634
Alphomelon xestopygaDHJ05|[275]|DHJPAR0012421|Costa Rica|657[0n]|BOLD:AAA1634
Alphomelon xestopygaDHJ05|[276]|DHJPAR0012423|Costa Rica|657[0n]|BOLD:AAA1634
Alphomelon xestopygaDHJ05|[277]|DHJPAR0012426|Costa Rica|657[0n]|BOLD:AAA1634
Alphomelon xestopygaDHJ05|[278]|DHJPAR0002458|Costa Rica|657[0n]|BOLD:AAA1634
Alphomelon xestopygaDHJ05|[279]|DHJPAR0002488|Costa Rica|657[0n]|BOLD:AAA1634
Alphomelon xestopygaDHJ05|[280]|DHJPAR0002490|Costa Rica|657[0n]|BOLD:AAA1634
Alphomelon xestopygaDHJ05|[281]|DHJPAR0058180|Costa Rica|658[0n]|BOLD:AAA1634
Alphomelon xestopygaDHJ05|[282]|DHJPAR0061770|Costa Rica|658[0n]|BOLD:AAA1634
Alphomelon xestopygaDHJ05|[283]|DHJPAR0058233|Costa Rica|658[0n]|BOLD:AAA1634
Alphomelon xestopygaDHJ05|[284]|DHJPAR0059720|Costa Rica|658[0n]|BOLD:AAA1634
Alphomelon xestopygaDHJ05|[285]|DHJPAR0061809|Costa Rica|658[0n]|BOLD:AAA1634
Alphomelon xestopygaDHJ05|[286]|DHJPAR0013169|Costa Rica|657[0n]|BOLD:AAA1634
Alphomelon xestopygaDHJ05|[287]|DHJPAR0059728|Costa Rica|658[0n]|BOLD:AAA1634
Alphomelon xestopygaDHJ05|[288]|DHJPAR0059729|Costa Rica|658[0n]|BOLD:AAA1634
Alphomelon xestopygaDHJ05|[289]|DHJPAR0004936|Costa Rica|657[0n]|BOLD:AAA1634
Alphomelon xestopygaDHJ05|[290]|DHJPAR0031113|Costa Rica|658[0n]|BOLD:AAA1634

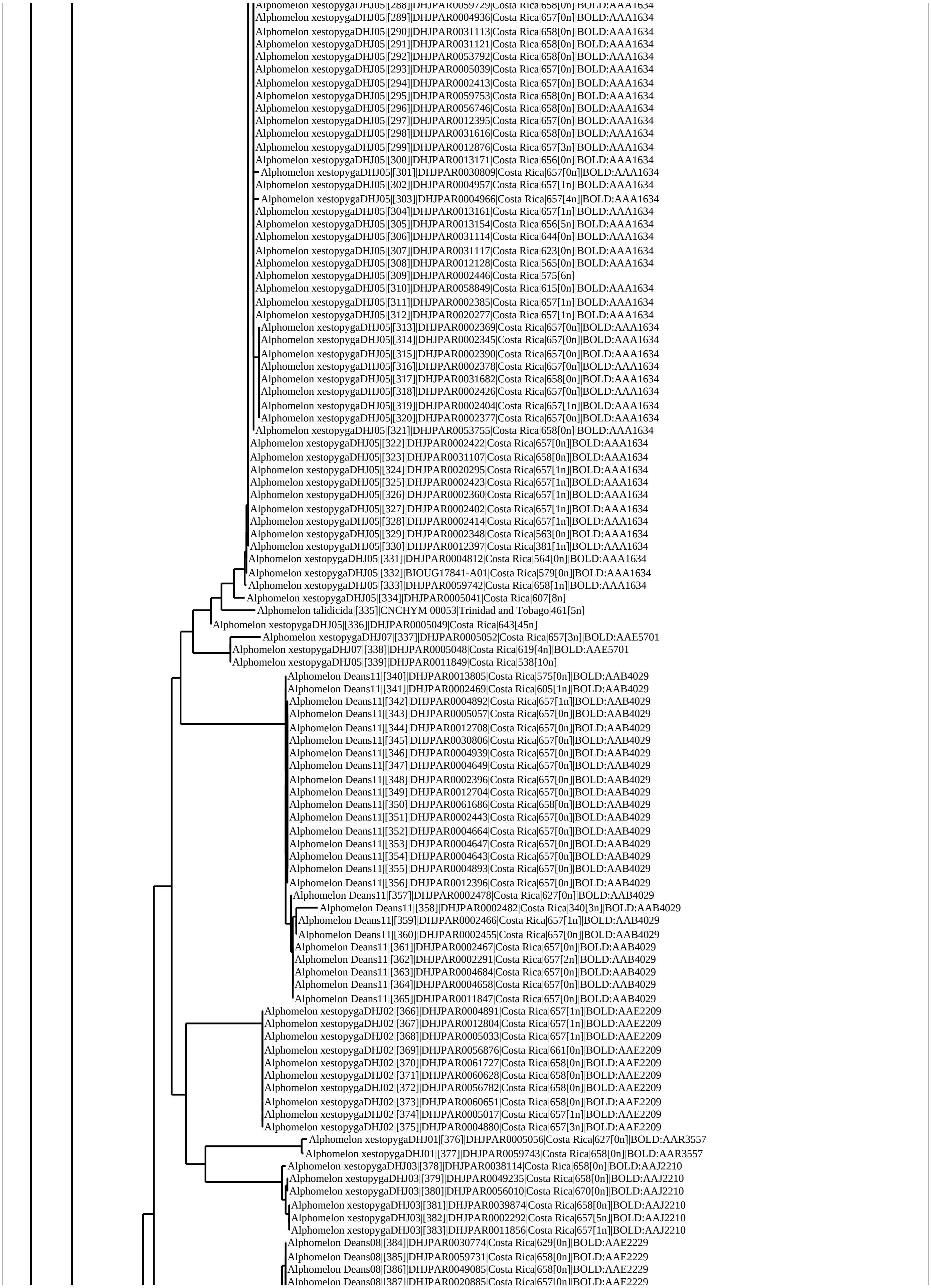
Alphomelon xestopygaDHJ05|[288]|DHJPAR0059729|Costa Rica|658[0n]|BOLD:AAA1634
Alphomelon xestopygaDHJ05|[289]|DHJPAR0004936|Costa Rica|657[0n]|BOLD:AAA1634
Alphomelon xestopygaDHJ05|[290]|DHJPAR0031113|Costa Rica|658[0n]|BOLD:AAA1634
Alphomelon xestopygaDHJ05|[291]|DHJPAR0031121|Costa Rica|658[0n]|BOLD:AAA1634
Alphomelon xestopygaDHJ05|[292]|DHJPAR0053792|Costa Rica|658[0n]|BOLD:AAA1634
Alphomelon xestopygaDHJ05|[293]|DHJPAR0005039|Costa Rica|657[0n]|BOLD:AAA1634
Alphomelon xestopygaDHJ05|[294]|DHJPAR0002413|Costa Rica|657[0n]|BOLD:AAA1634
Alphomelon xestopygaDHJ05|[295]|DHJPAR0059753|Costa Rica|658[0n]|BOLD:AAA1634
Alphomelon xestopygaDHJ05|[296]|DHJPAR0056746|Costa Rica|658[0n]|BOLD:AAA1634
Alphomelon xestopygaDHJ05|[297]|DHJPAR0012395|Costa Rica|657[0n]|BOLD:AAA1634
Alphomelon xestopygaDHJ05|[298]|DHJPAR0031616|Costa Rica|658[0n]|BOLD:AAA1634
Alphomelon xestopygaDHJ05|[299]|DHJPAR0012876|Costa Rica|657[3n]|BOLD:AAA1634
Alphomelon xestopygaDHJ05|[300]|DHJPAR0013171|Costa Rica|656[0n]|BOLD:AAA1634
Alphomelon xestopygaDHJ05|[301]|DHJPAR0030809|Costa Rica|657[0n]|BOLD:AAA1634
Alphomelon xestopygaDHJ05|[302]|DHJPAR0004957|Costa Rica|657[1n]|BOLD:AAA1634
Alphomelon xestopygaDHJ05|[303]|DHJPAR0004966|Costa Rica|657[4n]|BOLD:AAA1634
Alphomelon xestopygaDHJ05|[304]|DHJPAR0013161|Costa Rica|657[1n]|BOLD:AAA1634
Alphomelon xestopygaDHJ05|[305]|DHJPAR0013154|Costa Rica|656[5n]|BOLD:AAA1634
Alphomelon xestopygaDHJ05|[306]|DHJPAR0031114|Costa Rica|644[0n]|BOLD:AAA1634
Alphomelon xestopygaDHJ05|[307]|DHJPAR0031117|Costa Rica|623[0n]|BOLD:AAA1634
Alphomelon xestopygaDHJ05|[308]|DHJPAR0012128|Costa Rica|565[0n]|BOLD:AAA1634
Alphomelon xestopygaDHJ05|[309]|DHJPAR0002446|Costa Rica|575[6n]
Alphomelon xestopygaDHJ05|[310]|DHJPAR0058849|Costa Rica|615[0n]|BOLD:AAA1634
Alphomelon xestopygaDHJ05|[311]|DHJPAR0002385|Costa Rica|657[1n]|BOLD:AAA1634
Alphomelon xestopygaDHJ05|[312]|DHJPAR0020277|Costa Rica|657[1n]|BOLD:AAA1634
Alphomelon xestopygaDHJ05|[313]|DHJPAR0002369|Costa Rica|657[0n]|BOLD:AAA1634
Alphomelon xestopygaDHJ05|[314]|DHJPAR0002345|Costa Rica|657[0n]|BOLD:AAA1634
Alphomelon xestopygaDHJ05|[315]|DHJPAR0002390|Costa Rica|657[0n]|BOLD:AAA1634
Alphomelon xestopygaDHJ05|[316]|DHJPAR0002378|Costa Rica|657[0n]|BOLD:AAA1634
Alphomelon xestopygaDHJ05|[317]|DHJPAR0031682|Costa Rica|658[0n]|BOLD:AAA1634
Alphomelon xestopygaDHJ05|[318]|DHJPAR0002426|Costa Rica|657[0n]|BOLD:AAA1634
Alphomelon xestopygaDHJ05|[319]|DHJPAR0002404|Costa Rica|657[1n]|BOLD:AAA1634
Alphomelon xestopygaDHJ05|[320]|DHJPAR0002377|Costa Rica|657[0n]|BOLD:AAA1634
Alphomelon xestopygaDHJ05|[321]|DHJPAR0053755|Costa Rica|658[0n]|BOLD:AAA1634
Alphomelon xestopygaDHJ05|[322]|DHJPAR0002422|Costa Rica|657[0n]|BOLD:AAA1634
Alphomelon xestopygaDHJ05|[323]|DHJPAR0031107|Costa Rica|658[0n]|BOLD:AAA1634
Alphomelon xestopygaDHJ05|[324]|DHJPAR0020295|Costa Rica|657[1n]|BOLD:AAA1634
Alphomelon xestopygaDHJ05|[325]|DHJPAR0002423|Costa Rica|657[1n]|BOLD:AAA1634
Alphomelon xestopygaDHJ05|[326]|DHJPAR0002360|Costa Rica|657[1n]|BOLD:AAA1634
Alphomelon xestopygaDHJ05|[327]|DHJPAR0002402|Costa Rica|657[1n]|BOLD:AAA1634
Alphomelon xestopygaDHJ05|[328]|DHJPAR0002414|Costa Rica|657[1n]|BOLD:AAA1634
Alphomelon xestopygaDHJ05|[329]|DHJPAR0002348|Costa Rica|563[0n]|BOLD:AAA1634
Alphomelon xestopygaDHJ05|[330]|DHJPAR0012397|Costa Rica|381[1n]|BOLD:AAA1634
Alphomelon xestopygaDHJ05|[331]|DHJPAR0004812|Costa Rica|564[0n]|BOLD:AAA1634
Alphomelon xestopygaDHJ05|[332]|BIOUG17841-A01|Costa Rica|579[0n]|BOLD:AAA1634
Alphomelon xestopygaDHJ05|[333]|DHJPAR0059742|Costa Rica|658[1n]|BOLD:AAA1634
Alphomelon xestopygaDHJ05|[334]|DHJPAR0005041|Costa Rica|607[8n]
Alphomelon talidicida|[335]|CNCHYM 00053|Trinidad and Tobago|461[5n]
Alphomelon xestopygaDHJ05|[336]|DHJPAR0005049|Costa Rica|643[45n]
Alphomelon xestopygaDHJ07|[337]|DHJPAR0005052|Costa Rica|657[3n]|BOLD:AAE5701
Alphomelon xestopygaDHJ07|[338]|DHJPAR0005048|Costa Rica|619[4n]|BOLD:AAE5701
Alphomelon xestopygaDHJ05|[339]|DHJPAR0011849|Costa Rica|538[10n]
Alphomelon Deans11|[340]|DHJPAR0013805|Costa Rica|575[0n]|BOLD:AAB4029
Alphomelon Deans11|[341]|DHJPAR0002469|Costa Rica|605[1n]|BOLD:AAB4029
Alphomelon Deans11|[342]|DHJPAR0004892|Costa Rica|657[1n]|BOLD:AAB4029
Alphomelon Deans11|[343]|DHJPAR0005057|Costa Rica|657[0n]|BOLD:AAB4029
Alphomelon Deans11|[344]|DHJPAR0012708|Costa Rica|657[0n]|BOLD:AAB4029
Alphomelon Deans11|[345]|DHJPAR0030806|Costa Rica|657[0n]|BOLD:AAB4029
Alphomelon Deans11|[346]|DHJPAR0004939|Costa Rica|657[0n]|BOLD:AAB4029
Alphomelon Deans11|[347]|DHJPAR0004649|Costa Rica|657[0n]|BOLD:AAB4029
Alphomelon Deans11|[348]|DHJPAR0002396|Costa Rica|657[0n]|BOLD:AAB4029
Alphomelon Deans11|[349]|DHJPAR0012704|Costa Rica|657[0n]|BOLD:AAB4029
Alphomelon Deans11|[350]|DHJPAR0061686|Costa Rica|658[0n]|BOLD:AAB4029
Alphomelon Deans11|[351]|DHJPAR0002443|Costa Rica|657[0n]|BOLD:AAB4029
Alphomelon Deans11|[352]|DHJPAR0004664|Costa Rica|657[0n]|BOLD:AAB4029
Alphomelon Deans11|[353]|DHJPAR0004647|Costa Rica|657[0n]|BOLD:AAB4029
Alphomelon Deans11|[354]|DHJPAR0004643|Costa Rica|657[0n]|BOLD:AAB4029
Alphomelon Deans11|[355]|DHJPAR0004893|Costa Rica|657[0n]|BOLD:AAB4029
Alphomelon Deans11|[356]|DHJPAR0012396|Costa Rica|657[0n]|BOLD:AAB4029
Alphomelon Deans11|[357]|DHJPAR0002478|Costa Rica|627[0n]|BOLD:AAB4029
Alphomelon Deans11|[358]|DHJPAR0002482|Costa Rica|340[3n]|BOLD:AAB4029
Alphomelon Deans11|[359]|DHJPAR0002466|Costa Rica|657[1n]|BOLD:AAB4029
Alphomelon Deans11|[360]|DHJPAR0002455|Costa Rica|657[0n]|BOLD:AAB4029
Alphomelon Deans11|[361]|DHJPAR0002467|Costa Rica|657[0n]|BOLD:AAB4029
Alphomelon Deans11|[362]|DHJPAR0002291|Costa Rica|657[2n]|BOLD:AAB4029
Alphomelon Deans11|[363]|DHJPAR0004684|Costa Rica|657[0n]|BOLD:AAB4029
Alphomelon Deans11|[364]|DHJPAR0004658|Costa Rica|657[0n]|BOLD:AAB4029
Alphomelon Deans11|[365]|DHJPAR0011847|Costa Rica|657[0n]|BOLD:AAB4029
Alphomelon xestopygaDHJ02|[366]|DHJPAR0004891|Costa Rica|657[1n]|BOLD:AAE2209
Alphomelon xestopygaDHJ02|[367]|DHJPAR0012804|Costa Rica|657[1n]|BOLD:AAE2209
Alphomelon xestopygaDHJ02|[368]|DHJPAR0005033|Costa Rica|657[1n]|BOLD:AAE2209
Alphomelon xestopygaDHJ02|[369]|DHJPAR0056876|Costa Rica|661[0n]|BOLD:AAE2209
Alphomelon xestopygaDHJ02|[370]|DHJPAR0061727|Costa Rica|658[0n]|BOLD:AAE2209
Alphomelon xestopygaDHJ02|[371]|DHJPAR0060628|Costa Rica|658[0n]|BOLD:AAE2209
Alphomelon xestopygaDHJ02|[372]|DHJPAR0056782|Costa Rica|658[0n]|BOLD:AAE2209
Alphomelon xestopygaDHJ02|[373]|DHJPAR0060651|Costa Rica|658[0n]|BOLD:AAE2209
Alphomelon xestopygaDHJ02|[374]|DHJPAR0005017|Costa Rica|657[1n]|BOLD:AAE2209
Alphomelon xestopygaDHJ02|[375]|DHJPAR0004880|Costa Rica|657[3n]|BOLD:AAE2209
Alphomelon xestopygaDHJ01|[376]|DHJPAR0005056|Costa Rica|627[0n]|BOLD:AAR3557
Alphomelon xestopygaDHJ01|[377]|DHJPAR0059743|Costa Rica|658[0n]|BOLD:AAR3557
Alphomelon xestopygaDHJ03|[378]|DHJPAR0038114|Costa Rica|658[0n]|BOLD:AAJ2210
Alphomelon xestopygaDHJ03|[379]|DHJPAR0049235|Costa Rica|658[0n]|BOLD:AAJ2210
Alphomelon xestopygaDHJ03|[380]|DHJPAR0056010|Costa Rica|670[0n]|BOLD:AAJ2210
Alphomelon xestopygaDHJ03|[381]|DHJPAR0039874|Costa Rica|658[0n]|BOLD:AAJ2210
Alphomelon xestopygaDHJ03|[382]|DHJPAR0002292|Costa Rica|657[5n]|BOLD:AAJ2210
Alphomelon xestopygaDHJ03|[383]|DHJPAR0011856|Costa Rica|657[1n]|BOLD:AAJ2210
Alphomelon Deans08|[384]|DHJPAR0030774|Costa Rica|629[0n]|BOLD:AAE2229
Alphomelon Deans08|[385]|DHJPAR0059731|Costa Rica|658[0n]|BOLD:AAE2229
Alphomelon Deans08|[386]|DHJPAR0049085|Costa Rica|658[0n]|BOLD:AAE2229
Alphomelon Deans08|[387]|DHJPAR0020885|Costa Rica|657[0n]|BOLD:AAE2229

Alphomelon Deans08|[385]|DHJPAR0059731|Costa Rica|658[0n]|BOLD:AAE2229
Alphomelon Deans08|[386]|DHJPAR0049085|Costa Rica|658[0n]|BOLD:AAE2229
Alphomelon Deans08|[387]|DHJPAR0020885|Costa Rica|657[0n]|BOLD:AAE2229
Alphomelon Deans08|[388]|DHJPAR0004948|Costa Rica|657[1n]|BOLD:AAE2229
Alphomelon Deans08|[389]|DHJPAR0002471|Costa Rica|583[4n]|BOLD:AAE2229
Alphomelon Deans08|[390]|DHJPAR0002470|Costa Rica|657[2n]|BOLD:AAE2229
Alphomelon Deans08|[391]|DHJPAR0042940|Costa Rica|658[0n]|BOLD:AAE2229
Alphomelon Deans08|[392]|DHJPAR0042931|Costa Rica|658[0n]|BOLD:AAE2229
Alphomelon Deans08|[393]|DHJPAR0060226|Costa Rica|658[0n]|BOLD:AAE2229
Alphomelon bromeliphile|[394]|DHJPAR0004951|Costa Rica|353[4n]
Alphomelon bromeliphile|[395]|DHJPAR0005045|Costa Rica|641[0n]|BOLD:AAB5598
Alphomelon bromeliphile|[396]|DHJPAR0003979|Costa Rica|626[1n]|BOLD:AAB5598
Alphomelon bromeliphile|[397]|DHJPAR0051200|Costa Rica|626[0n]|BOLD:AAB5598
Alphomelon bromeliphile|[398]|DHJPAR0005029|Costa Rica|614[0n]|BOLD:AAB5598
Alphomelon bromeliphile|[399]|DHJPAR0004958|Costa Rica|657[12n]
Alphomelon bromeliphile|[400]|DHJPAR0004905|Costa Rica|657[0n]|BOLD:AAB5598
Alphomelon bromeliphile|[401]|DHJPAR0004674|Costa Rica|657[0n]|BOLD:AAB5598
Alphomelon bromeliphile|[402]|DHJPAR0004950|Costa Rica|657[0n]|BOLD:AAB5598
Alphomelon bromeliphile|[403]|DHJPAR0012700|Costa Rica|657[0n]|BOLD:AAB5598
Alphomelon bromeliphile|[404]|DHJPAR0012702|Costa Rica|657[0n]|BOLD:AAB5598
Alphomelon bromeliphile|[405]|DHJPAR0031657|Costa Rica|658[0n]|BOLD:AAB5598
Alphomelon bromeliphile|[406]|DHJPAR0031669|Costa Rica|658[0n]|BOLD:AAB5598
Alphomelon bromeliphile|[407]|DHJPAR0031621|Costa Rica|658[0n]|BOLD:AAB5598
Alphomelon bromeliphile|[408]|DHJPAR0012697|Costa Rica|657[0n]|BOLD:AAB5598
Alphomelon bromeliphile|[409]|DHJPAR0012698|Costa Rica|657[0n]|BOLD:AAB5598
Alphomelon bromeliphile|[410]|DHJPAR0012706|Costa Rica|657[0n]|BOLD:AAB5598
Alphomelon bromeliphile|[411]|DHJPAR0011842|Costa Rica|657[3n]|BOLD:AAB5598
Alphomelon bromeliphile|[412]|DHJPAR0005050|Costa Rica|657[1n]|BOLD:AAB5598
Alphomelon bromeliphile|[413]|DHJPAR0004972|Costa Rica|657[4n]|BOLD:AAB5598
Alphomelon bromeliphile|[414]|DHJPAR0005064|Costa Rica|655[2n]|BOLD:AAB5598
Alphomelon bromeliphile|[415]|DHJPAR0005053|Costa Rica|657[4n]|BOLD:AAB5598
Alphomelon bromeliphile|[416]|DHJPAR0005047|Costa Rica|657[3n]|BOLD:AAB5598
Alphomelon bromeliphile|[417]|DHJPAR0004955|Costa Rica|657[2n]|BOLD:AAB5598
Alphomelon bromeliphile|[418]|DHJPAR0011924|Costa Rica|657[1n]|BOLD:AAB5598
Alphomelon bromeliphile|[419]|DHJPAR0011840|Costa Rica|657[1n]|BOLD:AAB5598
Alphomelon Deans09|[420]|DHJPAR0047095|Costa Rica|627[0n]|BOLD:ABU7420
Alphomelon Deans09|[421]|DHJPAR0002445|Costa Rica|657[0n]|BOLD:ABU7420
Alphomelon Deans09|[422]|DHJPAR0047120|Costa Rica|658[0n]|BOLD:ABU7420
Alphomelon Deans09|[423]|DHJPAR0049095|Costa Rica|658[0n]|BOLD:ABU7420
Alphomelon Deans09|[424]|DHJPAR0012400|Costa Rica|657[0n]|BOLD:ABU7420
Alphomelon Deans09|[425]|DHJPAR0057788|Costa Rica|661[0n]|BOLD:ABU7420
Alphomelon Deans09|[426]|DHJPAR0057786|Costa Rica|661[0n]|BOLD:ABU7420
Alphomelon Deans09|[427]|DHJPAR0012416|Costa Rica|657[0n]|BOLD:ABU7420
Alphomelon Deans09|[428]|DHJPAR0038112|Costa Rica|658[0n]|BOLD:ABU7420
Alphomelon Deans09|[429]|DHJPAR0041758|Costa Rica|658[0n]|BOLD:ABU7420
Alphomelon Deans09|[430]|DHJPAR0041807|Costa Rica|658[0n]|BOLD:ABU7420
Alphomelon Deans09|[431]|DHJPAR0049225|Costa Rica|658[0n]|BOLD:ABU7420
Alphomelon Deans09|[432]|DHJPAR0041820|Costa Rica|658[0n]|BOLD:ABU7420
Alphomelon Deans09|[433]|DHJPAR0025653|Costa Rica|657[0n]|BOLD:ABU7420
Alphomelon Deans09|[434]|DHJPAR0057793|Costa Rica|661[0n]|BOLD:ABU7420
Alphomelon Deans09|[435]|DHJPAR0012701|Costa Rica|657[0n]|BOLD:ABU7420
Alphomelon Deans09|[436]|DHJPAR0004885|Costa Rica|657[1n]|BOLD:ABU7420
Alphomelon Deans09|[437]|DHJPAR0045224|Costa Rica|658[1n]|BOLD:ABU7420
Alphomelon Deans09|[438]|DHJPAR0004935|Costa Rica|657[1n]|BOLD:ABU7420
Alphomelon Deans09|[439]|DHJPAR0004940|Costa Rica|657[1n]|BOLD:ABU7420
Alphomelon Deans09|[440]|DHJPAR0005116|Costa Rica|657[1n]|BOLD:ABU7420
Alphomelon Deans09|[441]|DHJPAR0004882|Costa Rica|657[1n]|BOLD:ABU7420
Alphomelon Deans09|[442]|DHJPAR0012857|Costa Rica|629[0n]|BOLD:ABU7420
Alphomelon Deans09|[443]|DHJPAR0058212|Costa Rica|658[0n]|BOLD:ABU7420
Alphomelon Deans09|[444]|DHJPAR0058207|Costa Rica|658[0n]|BOLD:ABU7420
Alphomelon Deans09|[445]|DHJPAR0058201|Costa Rica|658[0n]|BOLD:ABU7420
Alphomelon Deans09|[446]|DHJPAR0058161|Costa Rica|658[0n]|BOLD:ABU7420
Alphomelon Deans09|[447]|DHJPAR0058153|Costa Rica|658[0n]|BOLD:ABU7420
Alphomelon Deans09|[448]|DHJPAR0062108|Costa Rica|658[0n]|BOLD:ABU7420
Alphomelon Deans09|[449]|DHJPAR0059482|Costa Rica|658[0n]|BOLD:ABU7420
Alphomelon Deans09|[450]|DHJPAR0038270|Costa Rica|658[0n]|BOLD:ABU7420
Alphomelon Deans09|[451]|DHJPAR0058838|Costa Rica|658[0n]|BOLD:ABU7420
Alphomelon Deans09|[452]|DHJPAR0012699|Costa Rica|657[0n]|BOLD:ABU7420
Alphomelon Deans09|[453]|DHJPAR0038962|Costa Rica|658[0n]|BOLD:ABU7420
Alphomelon Deans09|[454]|DHJPAR0038957|Costa Rica|658[0n]|BOLD:ABU7420
Alphomelon Deans09|[455]|DHJPAR0012859|Costa Rica|657[0n]|BOLD:ABU7420
Alphomelon|[456]|CCDB-07374 F11|French Guiana|658[0n]|BOLD:AAV7443
Alphomelon Deans12|[457]|DHJPAR0004805|Costa Rica|554[1n]|BOLD:AAB7535
Alphomelon Deans12|[458]|DHJPAR0031126|Costa Rica|622[0n]|BOLD:AAB7535
Alphomelon Deans12|[459]|DHJPAR0004654|Costa Rica|657[0n]|BOLD:AAB7535
Alphomelon Deans12|[460]|DHJPAR0005019|Costa Rica|654[0n]|BOLD:AAB7535
Alphomelon Deans12|[461]|DHJPAR0004917|Costa Rica|657[1n]|BOLD:AAB7535
Alphomelon Deans12|[462]|DHJPAR0012414|Costa Rica|657[0n]|BOLD:AAB7535
Alphomelon Deans12|[463]|DHJPAR0002460|Costa Rica|657[0n]|BOLD:AAB7535
Alphomelon Deans12|[464]|DHJPAR0058188|Costa Rica|658[0n]|BOLD:AAB7535
Alphomelon Deans12|[465]|DHJPAR0058190|Costa Rica|658[0n]|BOLD:AAB7535
Alphomelon Deans12|[466]|DHJPAR0058209|Costa Rica|658[0n]|BOLD:AAB7535
Alphomelon Deans12|[467]|DHJPAR0054695|Costa Rica|658[0n]|BOLD:AAB7535
Alphomelon Deans12|[468]|DHJPAR0064147|Costa Rica|658[0n]|BOLD:AAB7535
Alphomelon Deans12|[469]|DHJPAR0012120|Costa Rica|657[0n]|BOLD:AAB7535
Alphomelon Deans12|[470]|DHJPAR0030875|Costa Rica|657[0n]|BOLD:AAB7535
Alphomelon Deans12|[471]|DHJPAR0030883|Costa Rica|657[0n]|BOLD:AAB7535
Alphomelon Deans12|[472]|DHJPAR0030932|Costa Rica|657[0n]|BOLD:AAB7535
Alphomelon Deans12|[473]|DHJPAR0020276|Costa Rica|657[0n]|BOLD:AAB7535
Alphomelon Deans12|[474]|DHJPAR0012710|Costa Rica|657[0n]|BOLD:AAB7535
Alphomelon Deans12|[475]|DHJPAR0004924|Costa Rica|657[1n]|BOLD:AAB7535
Alphomelon Deans12|[476]|DHJPAR0025847|Costa Rica|656[0n]|BOLD:AAB7535
Alphomelon Deans12|[477]|BIOUG19726-B08|Costa Rica|588[0n]|BOLD:AAB7535
Alphomelon Deans12|[478]|BIOUG28011-A06|Costa Rica|600[0n]|BOLD:AAB7535
Alphomelon Deans12|[479]|DHJPAR0004888|Costa Rica|657[3n]|BOLD:AAB7535
Alphomelon Deans12|[480]|DHJPAR0020280|Costa Rica|657[0n]|BOLD:AAB7535
Alphomelon Deans12|[481]|DHJPAR0058963|Costa Rica|661[0n]|BOLD:AAB7535
Alphomelon Deans12|[482]|DHJPAR0058962|Costa Rica|661[0n]|BOLD:AAB7535
Alphomelon Deans12|[483]|DHJPAR0012863|Costa Rica|657[0n]|BOLD:AAB7535
Alphomelon Deans12|[484]|DHJPAR0060154|Costa Rica|658[0n]|BOLD:AAB7535

Alphomelon Deans12|[481]|DHJPAR[illegible]|Costa Rica|661[0n]|BOLD:AAB7535
Alphomelon Deans12|[482]|DHJPAR0058962|Costa Rica|661[0n]|BOLD:AAB7535
Alphomelon Deans12|[483]|DHJPAR0012863|Costa Rica|657[0n]|BOLD:AAB7535
Alphomelon Deans12|[484]|DHJPAR0060154|Costa Rica|658[0n]|BOLD:AAB7535
Alphomelon Deans13|[485]|DHJPAR0004802|Costa Rica|567[2n]|BOLD:AAA6775
Alphomelon Deans13|[486]|DHJPAR0057765|Costa Rica|623[0n]|BOLD:AAA6775
Alphomelon Deans13|[487]|DHJPAR0031132|Costa Rica|632[0n]|BOLD:AAA6775
Alphomelon Deans13|[488]|DHJPAR0004898|Costa Rica|595[5n]|BOLD:AAA6775
Alphomelon Deans13|[489]|DHJPAR0004949|Costa Rica|628[0n]|BOLD:AAA6775
Alphomelon Deans13|[490]|DHJPAR0005061|Costa Rica|650[0n]|BOLD:AAA6775
Alphomelon Deans13|[491]|DHJPAR0058160|Costa Rica|658[0n]|BOLD:AAA6775
Alphomelon Deans13|[492]|DHJPAR0048159|Costa Rica|658[0n]|BOLD:AAA6775
Alphomelon Deans13|[493]|DHJPAR0012707|Costa Rica|657[0n]|BOLD:AAA6775
Alphomelon Deans13|[494]|DHJPAR0057515|Costa Rica|658[0n]|BOLD:AAA6775
Alphomelon Deans13|[495]|DHJPAR0060156|Costa Rica|658[0n]|BOLD:AAA6775
Alphomelon Deans13|[496]|DHJPAR0002498|Costa Rica|657[0n]|BOLD:AAA6775
Alphomelon Deans13|[497]|DHJPAR0050981|Costa Rica|658[0n]|BOLD:AAA6775
Alphomelon Deans13|[498]|DHJPAR0061754|Costa Rica|658[0n]|BOLD:AAA6775
Alphomelon Deans13|[499]|DHJPAR0058205|Costa Rica|658[0n]|BOLD:AAA6775
Alphomelon Deans13|[500]|DHJPAR0062582|Costa Rica|658[0n]|BOLD:AAA6775
Alphomelon Deans13|[501]|DHJPAR0062590|Costa Rica|658[0n]|BOLD:AAA6775
Alphomelon Deans13|[502]|DHJPAR0062593|Costa Rica|658[0n]|BOLD:AAA6775
Alphomelon Deans13|[503]|DHJPAR0058867|Costa Rica|658[0n]|BOLD:AAA6775
Alphomelon Deans13|[504]|DHJPAR0025786|Costa Rica|655[0n]|BOLD:AAA6775
Alphomelon Deans13|[505]|DHJPAR0020596|Costa Rica|657[0n]|BOLD:AAA6775
Alphomelon Deans13|[506]|DHJPAR0004937|Costa Rica|657[0n]|BOLD:AAA6775
Alphomelon Deans13|[507]|DHJPAR0004969|Costa Rica|657[0n]|BOLD:AAA6775
Alphomelon Deans13|[508]|DHJPAR0004971|Costa Rica|657[0n]|BOLD:AAA6775
Alphomelon Deans13|[509]|DHJPAR0060613|Costa Rica|658[0n]|BOLD:AAA6775
Alphomelon Deans13|[510]|DHJPAR0005060|Costa Rica|657[0n]|BOLD:AAA6775
Alphomelon Deans13|[511]|DHJPAR0005114|Costa Rica|657[0n]|BOLD:AAA6775
Alphomelon Deans13|[512]|DHJPAR0054746|Costa Rica|658[0n]|BOLD:AAA6775
Alphomelon Deans13|[513]|DHJPAR0056706|Costa Rica|658[0n]|BOLD:AAA6775
Alphomelon Deans13|[514]|DHJPAR0061719|Costa Rica|658[0n]|BOLD:AAA6775
Alphomelon Deans13|[515]|DHJPAR0025617|Costa Rica|657[0n]|BOLD:AAA6775
Alphomelon Deans13|[516]|DHJPAR0053774|Costa Rica|658[0n]|BOLD:AAA6775
Alphomelon Deans13|[517]|DHJPAR0060621|Costa Rica|658[0n]|BOLD:AAA6775
Alphomelon Deans13|[518]|DHJPAR0060653|Costa Rica|658[0n]|BOLD:AAA6775
Alphomelon Deans13|[519]|DHJPAR0053717|Costa Rica|658[0n]|BOLD:AAA6775
Alphomelon Deans13|[520]|DHJPAR0056368|Costa Rica|661[0n]|BOLD:AAA6775
Alphomelon Deans13|[521]|DHJPAR0030714|Costa Rica|641[0n]|BOLD:AAA6775
Alphomelon Deans13|[522]|DHJPAR0002457|Costa Rica|657[0n]|BOLD:AAA6775
Alphomelon Deans13|[523]|DHJPAR0004657|Costa Rica|657[0n]|BOLD:AAA6775
Alphomelon Deans13|[524]|DHJPAR0004919|Costa Rica|657[0n]|BOLD:AAA6775
Alphomelon Deans13|[525]|DHJPAR0004894|Costa Rica|657[0n]|BOLD:AAA6775
Alphomelon Deans13|[526]|DHJPAR0012399|Costa Rica|657[0n]|BOLD:AAA6775
Alphomelon Deans13|[527]|DHJPAR0061661|Costa Rica|658[0n]|BOLD:AAA6775
Alphomelon Deans13|[528]|DHJPAR0020281|Costa Rica|657[0n]|BOLD:AAA6775
Alphomelon Deans13|[529]|DHJPAR0058938|Costa Rica|658[0n]|BOLD:AAA6775
Alphomelon Deans13|[530]|DHJPAR0058935|Costa Rica|658[0n]|BOLD:AAA6775
Alphomelon Deans13|[531]|DHJPAR0058921|Costa Rica|658[0n]|BOLD:AAA6775
Alphomelon Deans13|[532]|DHJPAR0058916|Costa Rica|658[0n]|BOLD:AAA6775
Alphomelon Deans13|[533]|DHJPAR0058893|Costa Rica|658[0n]|BOLD:AAA6775
Alphomelon Deans13|[534]|DHJPAR0058877|Costa Rica|658[0n]|BOLD:AAA6775
Alphomelon Deans13|[535]|DHJPAR0058847|Costa Rica|658[0n]|BOLD:AAA6775
Alphomelon Deans13|[536]|DHJPAR0056693|Costa Rica|658[0n]|BOLD:AAA6775
Alphomelon Deans13|[537]|DHJPAR0012855|Costa Rica|657[0n]|BOLD:AAA6775
Alphomelon Deans13|[538]|DHJPAR0046873|Costa Rica|658[0n]|BOLD:AAA6775
Alphomelon Deans13|[539]|DHJPAR0064082|Costa Rica|646[0n]|BOLD:AAA6775
Alphomelon Deans13|[540]|DHJPAR0026885|Costa Rica|597[0n]|BOLD:AAA6775
Alphomelon Deans13|[541]|DHJPAR0005113|Costa Rica|657[2n]|BOLD:AAA6775
Alphomelon Deans13|[542]|DHJPAR0025492|Costa Rica|657[0n]|BOLD:AAA6775
Alphomelon Deans13|[543]|DHJPAR0005115|Costa Rica|657[0n]|BOLD:AAA6775
Alphomelon Deans13|[544]|DHJPAR0004930|Costa Rica|657[0n]|BOLD:AAA6775
Alphomelon Deans13|[545]|DHJPAR0030830|Costa Rica|657[0n]|BOLD:AAA6775
Alphomelon Deans13|[546]|DHJPAR0030877|Costa Rica|657[0n]|BOLD:AAA6775
Alphomelon Deans13|[547]|DHJPAR0042917|Costa Rica|658[0n]|BOLD:AAA6775
Alphomelon Deans13|[548]|DHJPAR0002477|Costa Rica|657[2n]|BOLD:AAA6775
Alphomelon Deans13|[549]|DHJPAR0004884|Costa Rica|657[0n]|BOLD:AAA6775
Alphomelon Deans13|[550]|DHJPAR0020284|Costa Rica|657[0n]|BOLD:AAA6775
Alphomelon Deans13|[551]|DHJPAR0060183|Costa Rica|658[0n]|BOLD:AAA6775
Alphomelon Deans13|[552]|DHJPAR0060178|Costa Rica|658[0n]|BOLD:AAA6775
Alphomelon Deans13|[553]|DHJPAR0012874|Costa Rica|652[5n]|BOLD:AAA6775
Alphomelon Deans13|[554]|DHJPAR0002394|Costa Rica|657[0n]|BOLD:AAA6775
Alphomelon Deans13|[555]|DHJPAR0039875|Costa Rica|658[0n]|BOLD:AAA6775
Alphomelon Deans13|[556]|DHJPAR0063193|Costa Rica|658[0n]|BOLD:AAA6775
Alphomelon Deans13|[557]|DHJPAR0057761|Costa Rica|658[0n]|BOLD:AAA6775
Alphomelon Deans13|[558]|DHJPAR0025527|Costa Rica|657[0n]|BOLD:AAA6775
Alphomelon Deans13|[559]|DHJPAR0004912|Costa Rica|657[0n]|BOLD:AAA6775
Alphomelon Deans13|[560]|DHJPAR0012420|Costa Rica|657[0n]|BOLD:AAA6775
Alphomelon Deans13|[561]|DHJPAR0004642|Costa Rica|657[0n]|BOLD:AAA6775
Alphomelon Deans13|[562]|DHJPAR0002464|Costa Rica|657[0n]|BOLD:AAA6775
Alphomelon Deans13|[563]|DHJPAR0002475|Costa Rica|657[0n]|BOLD:AAA6775
Alphomelon Deans13|[564]|DHJPAR0002499|Costa Rica|657[0n]|BOLD:AAA6775
Alphomelon Deans13|[565]|DHJPAR0025376|Costa Rica|657[0n]|BOLD:AAA6775
Alphomelon Deans13|[566]|DHJPAR0062633|Costa Rica|658[0n]|BOLD:AAA6775
Alphomelon Deans13|[567]|DHJPAR0004970|Costa Rica|657[0n]|BOLD:AAA6775
Alphomelon Deans13|[568]|DHJPAR0005063|Costa Rica|657[0n]|BOLD:AAA6775
Alphomelon Deans13|[569]|DHJPAR0064063|Costa Rica|658[0n]|BOLD:AAA6775
Alphomelon Deans13|[570]|DHJPAR0002497|Costa Rica|657[0n]|BOLD:AAA6775
Alphomelon Deans13|[571]|DHJPAR0056740|Costa Rica|658[0n]|BOLD:AAA6775
Alphomelon Deans13|[572]|DHJPAR0050090|Costa Rica|658[0n]|BOLD:AAA6775
Alphomelon Deans13|[573]|DHJPAR0060610|Costa Rica|658[0n]|BOLD:AAA6775
Alphomelon Deans13|[574]|DHJPAR0011925|Costa Rica|657[1n]|BOLD:AAA6775
Alphomelon Deans13|[575]|DHJPAR0004887|Costa Rica|657[1n]|BOLD:AAA6775
Alphomelon Deans13|[576]|DHJPAR0004942|Costa Rica|657[1n]|BOLD:AAA6775
Alphomelon Deans13|[577]|DHJPAR0030769|Costa Rica|637[0n]|BOLD:AAA6775
Alphomelon Deans13|[578]|DHJPAR0030704|Costa Rica|636[0n]|BOLD:AAA6775
Alphomelon Deans13|[579]|DHJPAR0002484|Costa Rica|303[3n]
Alphomelon Deans13|[580]|DHJPAR0002393|Costa Rica|657[2n]|BOLD:AAA6775

Alphomelon Deans13|[578]|DHJPAR0030704|Costa Rica|636[0n]|BOLD:AAA6775
Alphomelon Deans13|[579]|DHJPAR0002484|Costa Rica|303[3n]
Alphomelon Deans13|[580]|DHJPAR0002393|Costa Rica|657[2n]|BOLD:AAA6775
Alphomelon Deans13|[581]|DHJPAR0025887|Costa Rica|657[1n]|BOLD:AAA6775
Alphomelon Deans13|[582]|DHJPAR0060682|Costa Rica|658[0n]|BOLD:AAA6775
Alphomelon nanosoma|[583]|DHJPAR0005058|Costa Rica|627[7n]
Alphomelon nanosoma|[584]|DHJPAR0004056|Costa Rica|627[2n]|BOLD:AAB9792
Alphomelon nanosoma|[585]|DHJPAR0005054|Costa Rica|639[1n]|BOLD:AAB9792
Alphomelon nanosoma|[586]|DHJPAR0030720|Costa Rica|655[0n]|BOLD:AAB9792
Alphomelon nanosoma|[587]|DHJPAR0005038|Costa Rica|657[0n]|BOLD:AAB9792
Alphomelon nanosoma|[588]|DHJPAR0054602|Costa Rica|658[0n]|BOLD:AAB9792
Alphomelon nanosoma|[589]|DHJPAR0061690|Costa Rica|657[0n]|BOLD:AAB9792
Alphomelon nanosoma|[590]|DHJPAR0060214|Costa Rica|658[0n]|BOLD:AAB9792
Alphomelon nanosoma|[591]|DHJPAR0059477|Costa Rica|658[0n]|BOLD:AAB9792
Alphomelon nanosoma|[592]|DHJPAR0054652|Costa Rica|658[0n]|BOLD:AAB9792
Alphomelon nanosoma|[593]|DHJPAR0035428|Costa Rica|658[0n]|BOLD:AAB9792
Alphomelon nanosoma|[594]|DHJPAR0060187|Costa Rica|658[0n]|BOLD:AAB9792
Alphomelon nanosoma|[595]|DHJPAR0035449|Costa Rica|658[0n]|BOLD:AAB9792
Alphomelon nanosoma|[596]|DHJPAR0035433|Costa Rica|658[0n]|BOLD:AAB9792
Alphomelon nanosoma|[597]|DHJPAR0058933|Costa Rica|658[0n]|BOLD:AAB9792
Alphomelon nanosoma|[598]|DHJPAR0060601|Costa Rica|658[0n]|BOLD:AAB9792
Alphomelon nanosoma|[599]|DHJPAR0060598|Costa Rica|658[0n]|BOLD:AAB9792
Alphomelon nanosoma|[600]|DHJPAR0049188|Costa Rica|658[0n]|BOLD:AAB9792
Alphomelon nanosoma|[601]|DHJPAR0058171|Costa Rica|658[0n]|BOLD:AAB9792
Alphomelon nanosoma|[602]|DHJPAR0060221|Costa Rica|658[0n]|BOLD:AAB9792
Alphomelon nanosoma|[603]|DHJPAR0053687|Costa Rica|658[0n]|BOLD:AAB9792
Alphomelon nanosoma|[604]|DHJPAR0059486|Costa Rica|658[0n]|BOLD:AAB9792
Alphomelon nanosoma|[605]|DHJPAR0051812|Costa Rica|661[0n]|BOLD:AAB9792
Alphomelon nanosoma|[606]|DHJPAR0038967|Costa Rica|658[0n]|BOLD:AAB9792
Alphomelon nanosoma|[607]|DHJPAR0058191|Costa Rica|658[0n]|BOLD:AAB9792
Alphomelon nanosoma|[608]|DHJPAR0058960|Costa Rica|661[0n]|BOLD:AAB9792
Alphomelon nanosoma|[609]|DHJPAR0054600|Costa Rica|658[0n]|BOLD:AAB9792
Alphomelon nanosoma|[610]|DHJPAR0034199|Costa Rica|655[0n]|BOLD:AAB9792
Alphomelon nanosoma|[611]|DHJPAR0004910|Costa Rica|657[0n]|BOLD:AAB9792
Alphomelon nanosoma|[612]|DHJPAR0012422|Costa Rica|657[0n]|BOLD:AAB9792
Alphomelon nanosoma|[613]|DHJPAR0012410|Costa Rica|657[0n]|BOLD:AAB9792
Alphomelon nanosoma|[614]|DHJPAR0020463|Costa Rica|657[0n]|BOLD:AAB9792
Alphomelon nanosoma|[615]|DHJPAR0020783|Costa Rica|657[0n]|BOLD:AAB9792
Alphomelon nanosoma|[616]|DHJPAR0058890|Costa Rica|658[0n]|BOLD:AAB9792
Alphomelon nanosoma|[617]|DHJPAR0047044|Costa Rica|661[0n]|BOLD:AAB9792
Alphomelon nanosoma|[618]|DHJPAR0058875|Costa Rica|658[0n]|BOLD:AAB9792
Alphomelon nanosoma|[619]|DHJPAR0060149|Costa Rica|658[0n]|BOLD:AAB9792
Alphomelon nanosoma|[620]|DHJPAR0058870|Costa Rica|658[0n]|BOLD:AAB9792
Alphomelon nanosoma|[621]|DHJPAR0060134|Costa Rica|658[0n]|BOLD:AAB9792
Alphomelon nanosoma|[622]|DHJPAR0058850|Costa Rica|658[0n]|BOLD:AAB9792
Alphomelon nanosoma|[623]|DHJPAR0012861|Costa Rica|657[0n]|BOLD:AAB9792
Alphomelon nanosoma|[624]|DHJPAR0060679|Costa Rica|658[0n]|BOLD:AAB9792
Alphomelon talidicida|[625]|DHJPAR0030709|Costa Rica|629[0n]|BOLD:AAA7259
Alphomelon talidicida|[626]|DHJPAR0048144|Costa Rica|658[0n]|BOLD:AAA7259
Alphomelon talidicida|[627]|DHJPAR0030698|Costa Rica|658[0n]|BOLD:AAA7259
Alphomelon talidicida|[628]|DHJPAR0056390|Costa Rica|661[0n]|BOLD:AAA7259
Alphomelon talidicida|[629]|DHJPAR0047253|Costa Rica|658[0n]|BOLD:AAA7259
Alphomelon talidicida|[630]|DHJPAR0002454|Costa Rica|558[14n]
Alphomelon talidicida|[631]|DHJPAR0004973|Costa Rica|627[2n]|BOLD:AAA7259
Alphomelon talidicida|[632]|DHJPAR0002453|Costa Rica|627[0n]|BOLD:AAA7259
Alphomelon talidicida|[633]|DHJPAR0060201|Costa Rica|658[0n]|BOLD:AAA7259
Alphomelon talidicida|[634]|DHJPAR0020287|Costa Rica|657[0n]|BOLD:AAA7259
Alphomelon talidicida|[635]|DHJPAR0012408|Costa Rica|657[0n]|BOLD:AAA7259
Alphomelon talidicida|[636]|DHJPAR0004886|Costa Rica|657[0n]|BOLD:AAA7259
Alphomelon talidicida|[637]|DHJPAR0063407|Costa Rica|658[0n]|BOLD:AAA7259
Alphomelon talidicida|[638]|DHJPAR0004655|Costa Rica|657[0n]|BOLD:AAA7259
Alphomelon talidicida|[639]|DHJPAR0050959|Costa Rica|658[0n]|BOLD:AAA7259
Alphomelon talidicida|[640]|DHJPAR0053777|Costa Rica|658[0n]|BOLD:AAA7259
Alphomelon talidicida|[641]|DHJPAR0004925|Costa Rica|657[0n]|BOLD:AAA7259
Alphomelon talidicida|[642]|DHJPAR0020282|Costa Rica|657[2n]|BOLD:AAA7259
Alphomelon talidicida|[643]|DHJPAR0065240|Costa Rica|638[0n]|BOLD:AAA7259
Alphomelon talidicida|[644]|DHJPAR0002435|Costa Rica|656[0n]|BOLD:AAA7259
Alphomelon talidicida|[645]|DHJPAR0011854|Costa Rica|657[0n]|BOLD:AAA7259
Alphomelon talidicida|[646]|DHJPAR0011843|Costa Rica|657[0n]|BOLD:AAA7259
Alphomelon talidicida|[647]|DHJPAR0012856|Costa Rica|657[0n]|BOLD:AAA7259
Alphomelon talidicida|[648]|DHJPAR0060683|Costa Rica|658[0n]|BOLD:AAA7259
Alphomelon talidicida|[649]|DHJPAR0056698|Costa Rica|630[0n]|BOLD:AAA7259
Alphomelon talidicida|[650]|DHJPAR0002927|Costa Rica|627[0n]|BOLD:AAA7259
Alphomelon talidicida|[651]|DHJPAR0004962|Costa Rica|627[0n]|BOLD:AAA7259
Alphomelon talidicida|[652]|DHJPAR0038166|Costa Rica|658[0n]|BOLD:AAA7259
Alphomelon talidicida|[653]|DHJPAR0061723|Costa Rica|658[0n]|BOLD:AAA7259
Alphomelon talidicida|[654]|DHJPAR0060138|Costa Rica|658[0n]|BOLD:AAA7259
Alphomelon talidicida|[655]|DHJPAR0058892|Costa Rica|658[0n]|BOLD:AAA7259
Alphomelon talidicida|[656]|DHJPAR0047138|Costa Rica|658[0n]|BOLD:AAA7259
Alphomelon talidicida|[657]|DHJPAR0056694|Costa Rica|658[0n]|BOLD:AAA7259
Alphomelon talidicida|[658]|DHJPAR0004915|Costa Rica|657[0n]|BOLD:AAA7259
Alphomelon talidicida|[659]|DHJPAR0047254|Costa Rica|658[0n]|BOLD:AAA7259
Alphomelon talidicida|[660]|DHJPAR0047259|Costa Rica|658[0n]|BOLD:AAA7259
Alphomelon talidicida|[661]|DHJPAR0004666|Costa Rica|657[0n]|BOLD:AAA7259
Alphomelon talidicida|[662]|DHJPAR0004667|Costa Rica|657[0n]|BOLD:AAA7259
Alphomelon talidicida|[663]|DHJPAR0002442|Costa Rica|657[0n]|BOLD:AAA7259
Alphomelon talidicida|[664]|DHJPAR0002450|Costa Rica|657[0n]|BOLD:AAA7259
Alphomelon talidicida|[665]|DHJPAR0061684|Costa Rica|658[0n]|BOLD:AAA7259
Alphomelon talidicida|[666]|DHJPAR0061685|Costa Rica|658[0n]|BOLD:AAA7259
Alphomelon talidicida|[667]|DHJPAR0050979|Costa Rica|658[0n]|BOLD:AAA7259
Alphomelon talidicida|[668]|DHJPAR0056697|Costa Rica|658[0n]|BOLD:AAA7259
Alphomelon talidicida|[669]|DHJPAR0058206|Costa Rica|658[0n]|BOLD:AAA7259
Alphomelon talidicida|[670]|DHJPAR0061620|Costa Rica|658[0n]|BOLD:AAA7259
Alphomelon talidicida|[671]|DHJPAR0058224|Costa Rica|658[0n]|BOLD:AAA7259
Alphomelon talidicida|[672]|DHJPAR0061790|Costa Rica|658[0n]|BOLD:AAA7259
Alphomelon talidicida|[673]|DHJPAR0058864|Costa Rica|658[0n]|BOLD:AAA7259
Alphomelon talidicida|[674]|DHJPAR0041819|Costa Rica|658[0n]|BOLD:AAA7259
Alphomelon talidicida|[675]|DHJPAR0004941|Costa Rica|657[0n]|BOLD:AAA7259
Alphomelon talidicida|[676]|DHJPAR0004961|Costa Rica|657[0n]|BOLD:AAA7259
Alphomelon talidicida|[677]|DHJPAR0004967|Costa Rica|657[0n]|BOLD:AAA7259

Alphomelon talidicida|[675]|DHJPAR0004941|Costa Rica|657[0n]|BOLD:AAA7259
Alphomelon talidicida|[676]|DHJPAR0004961|Costa Rica|657[0n]|BOLD:AAA7259
Alphomelon talidicida|[677]|DHJPAR0004967|Costa Rica|657[0n]|BOLD:AAA7259
Alphomelon talidicida|[678]|DHJPAR0062733|Costa Rica|658[0n]|BOLD:AAA7259
Alphomelon talidicida|[679]|DHJPAR0005046|Costa Rica|657[0n]|BOLD:AAA7259
Alphomelon talidicida|[680]|DHJPAR0005062|Costa Rica|657[0n]|BOLD:AAA7259
Alphomelon talidicida|[681]|DHJPAR0058910|Costa Rica|658[0n]|BOLD:AAA7259
Alphomelon talidicida|[682]|DHJPAR0054700|Costa Rica|658[0n]|BOLD:AAA7259
Alphomelon talidicida|[683]|DHJPAR0056704|Costa Rica|658[0n]|BOLD:AAA7259
Alphomelon talidicida|[684]|DHJPAR0056551|Costa Rica|658[0n]|BOLD:AAA7259
Alphomelon talidicida|[685]|DHJPAR0056780|Costa Rica|658[0n]|BOLD:AAA7259
Alphomelon talidicida|[686]|DHJPAR0056730|Costa Rica|658[0n]|BOLD:AAA7259
Alphomelon talidicida|[687]|DHJPAR0051199|Costa Rica|658[0n]|BOLD:AAA7259
Alphomelon talidicida|[688]|DHJPAR0051203|Costa Rica|658[0n]|BOLD:AAA7259
Alphomelon talidicida|[689]|DHJPAR0047139|Costa Rica|658[0n]|BOLD:AAA7259
Alphomelon talidicida|[690]|DHJPAR0049216|Costa Rica|658[0n]|BOLD:AAA7259
Alphomelon talidicida|[691]|DHJPAR0004932|Costa Rica|657[1n]|BOLD:AAA7259
Alphomelon talidicida|[692]|DHJPAR0011857|Costa Rica|657[4n]|BOLD:AAA7259
Alphomelon talidicida|[693]|DHJPAR0062109|Costa Rica|637[0n]|BOLD:AAA7259
Alphomelon talidicida|[694]|DHJPAR0047255|Costa Rica|621[0n]|BOLD:AAA7259
Alphomelon talidicida|[695]|DHJPAR0060131|Costa Rica|634[0n]|BOLD:AAA7259
Alphomelon talidicida|[696]|DHJPAR0056679|Costa Rica|633[0n]|BOLD:AAA7259
Alphomelon talidicida|[697]|DHJPAR0065190|Costa Rica|639[0n]|BOLD:AAA7259
Alphomelon talidicida|[698]|DHJPAR0055247|Costa Rica|658[0n]|BOLD:AAA7259
Alphomelon talidicida|[699]|DHJPAR0060687|Costa Rica|658[0n]|BOLD:AAA7259
Alphomelon talidicida|[700]|DHJPAR0060676|Costa Rica|658[0n]|BOLD:AAA7259
Alphomelon talidicida|[701]|DHJPAR0039876|Costa Rica|658[0n]|BOLD:AAA7259
Alphomelon talidicida|[702]|DHJPAR0058876|Costa Rica|658[0n]|BOLD:AAA7259
Alphomelon talidicida|[703]|DHJPAR0058887|Costa Rica|658[0n]|BOLD:AAA7259
Alphomelon talidicida|[704]|DHJPAR0047131|Costa Rica|658[0n]|BOLD:AAA7259
Alphomelon talidicida|[705]|DHJPAR0058973|Costa Rica|661[0n]|BOLD:AAA7259
Alphomelon talidicida|[706]|DHJPAR0004889|Costa Rica|657[0n]|BOLD:AAA7259
Alphomelon talidicida|[707]|DHJPAR0004909|Costa Rica|657[0n]|BOLD:AAA7259
Alphomelon talidicida|[708]|DHJPAR0004921|Costa Rica|657[0n]|BOLD:AAA7259
Alphomelon talidicida|[709]|DHJPAR0047256|Costa Rica|658[0n]|BOLD:AAA7259
Alphomelon talidicida|[710]|DHJPAR0021271|Costa Rica|657[0n]|BOLD:AAA7259
Alphomelon talidicida|[711]|DHJPAR0002463|Costa Rica|657[0n]|BOLD:AAA7259
Alphomelon talidicida|[712]|DHJPAR0056879|Costa Rica|661[0n]|BOLD:AAA7259
Alphomelon talidicida|[713]|DHJPAR0058181|Costa Rica|658[0n]|BOLD:AAA7259
Alphomelon talidicida|[714]|DHJPAR0058859|Costa Rica|658[0n]|BOLD:AAA7259
Alphomelon talidicida|[715]|DHJPAR0004938|Costa Rica|657[0n]|BOLD:AAA7259
Alphomelon talidicida|[716]|DHJPAR0004963|Costa Rica|657[0n]|BOLD:AAA7259
Alphomelon talidicida|[717]|DHJPAR0004965|Costa Rica|657[0n]|BOLD:AAA7259
Alphomelon talidicida|[718]|DHJPAR0004968|Costa Rica|657[0n]|BOLD:AAA7259
Alphomelon talidicida|[719]|DHJPAR0005025|Costa Rica|657[0n]|BOLD:AAA7259
Alphomelon talidicida|[720]|DHJPAR0005065|Costa Rica|657[0n]|BOLD:AAA7259
Alphomelon talidicida|[721]|DHJPAR0056506|Costa Rica|658[0n]|BOLD:AAA7259
Alphomelon talidicida|[722]|DHJPAR0056512|Costa Rica|658[0n]|BOLD:AAA7259
Alphomelon talidicida|[723]|DHJPAR0060622|Costa Rica|658[0n]|BOLD:AAA7259
Alphomelon talidicida|[724]|DHJPAR0061724|Costa Rica|658[0n]|BOLD:AAA7259
Alphomelon talidicida|[725]|DHJPAR0012711|Costa Rica|657[0n]|BOLD:AAA7259
Alphomelon talidicida|[726]|DHJPAR0004922|Costa Rica|657[2n]|BOLD:AAA7259
Alphomelon talidicida|[727]|DHJPAR0034269|Costa Rica|654[0n]|BOLD:AAA7259
Alphomelon talidicida|[728]|DHJPAR0011863|Costa Rica|657[3n]|BOLD:AAA7259
Alphomelon talidicida|[729]|DHJPAR0002500|Costa Rica|657[4n]|BOLD:AAA7259
Alphomelon talidicida|[730]|DHJPAR0011861|Costa Rica|574[4n]|BOLD:AAA7259
Alphomelon talidicida|[731]|DHJPAR0002486|Costa Rica|657[5n]|BOLD:AAA7259
Alphomelon talidicida|[732]|DHJPAR0065237|Costa Rica|638[0n]|BOLD:AAA7259
Alphomelon talidicida|[733]|DHJPAR0012875|Costa Rica|657[0n]|BOLD:AAA7259
Alphomelon talidicida|[734]|DHJPAR0039873|Costa Rica|658[0n]|BOLD:AAA7259
Alphomelon talidicida|[735]|DHJPAR0011860|Costa Rica|657[0n]|BOLD:AAA7259
Alphomelon talidicida|[736]|DHJPAR0056685|Costa Rica|658[0n]|BOLD:AAA7259
Alphomelon talidicida|[737]|DHJPAR0012853|Costa Rica|657[0n]|BOLD:AAA7259
Alphomelon talidicida|[738]|DHJPAR0058971|Costa Rica|661[0n]|BOLD:AAA7259
Alphomelon talidicida|[739]|DHJPAR0050091|Costa Rica|658[0n]|BOLD:AAA7259
Alphomelon Deans31|[740]|DHJPAR0051293|Costa Rica|658[0n]|BOLD:ACB1223
Alphomelon Deans31|[741]|DHJPAR0049074|Costa Rica|658[0n]|BOLD:ACB1223
Alphomelon Deans32|[742]|DHJPAR0051292|Costa Rica|658[0n]|BOLD:ACJ4259
Alphomelon Deans15|[743]|DHJPAR0005028|Costa Rica|657[0n]|BOLD:AAJ2171
Alphomelon Deans15|[744]|DHJPAR0012125|Costa Rica|657[0n]|BOLD:AAJ2171
Alphomelon Deans30|[745]|DHJPAR0056849|Costa Rica|657[0n]|BOLD:AAJ2207
Alphomelon Deans30|[746]|DHJPAR0038181|Costa Rica|658[0n]|BOLD:AAJ2207
Alphomelon Deans30|[747]|DHJPAR0030952|Costa Rica|657[0n]|BOLD:AAJ2207
Alphomelon Deans30|[748]|DHJPAR0031005|Costa Rica|658[0n]|BOLD:AAJ2207
Alphomelon Deans30|[749]|DHJPAR0060659|Costa Rica|658[0n]|BOLD:AAJ2207
Alphomelon Deans30|[750]|DHJPAR0064167|Costa Rica|658[0n]|BOLD:AAJ2207
Alphomelon Deans30|[751]|DHJPAR0030884|Costa Rica|360[0n]|BOLD:AAJ2207
Alphomelon Deans30|[752]|DHJPAR0059774|Costa Rica|658[0n]|BOLD:AAJ2207
Alphomelon Deans30|[753]|DHJPAR0059031|Costa Rica|660[2n]|BOLD:AAJ2207
Alphomelon Deans30|[754]|DHJPAR0059030|Costa Rica|660[0n]|BOLD:AAJ2207
Alphomelon Janzen22|[755]|DHJPAR0064007|Costa Rica|586[0n]|BOLD:ABX0806
Alphomelon Janzen22|[756]|DHJPAR0046796|Costa Rica|658[0n]|BOLD:ABX0806
Alphomelon Janzen22|[757]|DHJPAR0058263|Costa Rica|658[0n]|BOLD:ABX0806
Alphomelon Janzen22|[758]|DHJPAR0049470|Costa Rica|658[0n]|BOLD:ABX0806
Alphomelon Janzen22|[759]|DHJPAR0058256|Costa Rica|658[0n]|BOLD:ABX0806
Alphomelon Janzen22|[760]|DHJPAR0051849|Costa Rica|658[1n]|BOLD:ABX0806
Alphomelon Janzen22|[761]|DHJPAR0053843|Costa Rica|661[0n]|BOLD:ABX0806
Alphomelon Janzen22|[762]|DHJPAR0053818|Costa Rica|658[0n]|BOLD:ABX0806
Alphomelon Janzen22|[763]|DHJPAR0053809|Costa Rica|658[0n]|BOLD:ABX0806
Alphomelon Deans24|[764]|DHJPAR0047257|Costa Rica|658[0n]|BOLD:AAR3562
Alphomelon Deans24|[765]|DHJPAR0012413|Costa Rica|657[0n]|BOLD:AAR3562
Alphomelon arecaphileDHJ01|[766]|DHJPAR0064074|Costa Rica|585[0n]|BOLD:AAB1086
Alphomelon arecaphileDHJ01|[767]|DHJPAR0002473|Costa Rica|627[0n]|BOLD:AAB1086
Alphomelon arecaphileDHJ01|[768]|DHJPAR0002474|Costa Rica|657[5n]|BOLD:AAB1086
Alphomelon arecaphileDHJ01|[769]|DHJPAR0002447|Costa Rica|575[1n]|BOLD:AAB1086
Alphomelon arecaphileDHJ01|[770]|DHJPAR0058974|Costa Rica|661[1n]|BOLD:AAB1086
Alphomelon arecaphileDHJ01|[771]|DHJPAR0012858|Costa Rica|638[0n]|BOLD:AAB1086
Alphomelon arecaphileDHJ01|[772]|DHJPAR0012424|Costa Rica|657[0n]|BOLD:AAB1086
Alphomelon arecaphileDHJ01|[773]|DHJPAR0004671|Costa Rica|657[0n]|BOLD:AAB1086
[illegible]

Alphomelon arecaphileDHJ01|[771]|[illegible]|Costa Rica|[illegible]|BOLD:AAB1086
Alphomelon arecaphileDHJ01|[772]|DHJPAR0012424|Costa Rica|657[0n]|BOLD:AAB1086
Alphomelon arecaphileDHJ01|[773]|DHJPAR0004671|Costa Rica|657[0n]|BOLD:AAB1086
Alphomelon arecaphileDHJ01|[774]|DHJPAR0060133|Costa Rica|658[0n]|BOLD:AAB1086
Alphomelon arecaphileDHJ01|[775]|DHJPAR0012124|Costa Rica|657[0n]|BOLD:AAB1086
Alphomelon arecaphileDHJ01|[776]|DHJPAR0050978|Costa Rica|658[0n]|BOLD:AAB1086
Alphomelon arecaphileDHJ01|[777]|DHJPAR0058852|Costa Rica|658[0n]|BOLD:AAB1086
Alphomelon arecaphileDHJ01|[778]|DHJPAR0005026|Costa Rica|657[0n]|BOLD:AAB1086
Alphomelon arecaphileDHJ01|[779]|DHJPAR0060151|Costa Rica|658[0n]|BOLD:AAB1086
Alphomelon arecaphileDHJ01|[780]|DHJPAR0004927|Costa Rica|657[0n]|BOLD:AAB1086
Alphomelon arecaphileDHJ01|[781]|DHJPAR0004934|Costa Rica|657[0n]|BOLD:AAB1086
Alphomelon arecaphileDHJ01|[782]|DHJPAR0060611|Costa Rica|658[0n]|BOLD:AAB1086
Alphomelon arecaphileDHJ01|[783]|DHJPAR0004903|Costa Rica|657[0n]|BOLD:AAB1086
Alphomelon arecaphileDHJ01|[784]|DHJPAR0060648|Costa Rica|658[0n]|BOLD:AAB1086
Alphomelon arecaphileDHJ01|[785]|DHJPAR0026445|Costa Rica|657[0n]|BOLD:AAB1086
Alphomelon arecaphileDHJ01|[786]|DHJPAR0060647|Costa Rica|658[0n]|BOLD:AAB1086
Alphomelon arecaphileDHJ01|[787]|DHJPAR0004923|Costa Rica|657[4n]|BOLD:AAB1086
Alphomelon arecaphileDHJ01|[788]|DHJPAR0004896|Costa Rica|657[1n]|BOLD:AAB1086
Alphomelon arecaphileDHJ01|[789]|DHJPAR0060129|Costa Rica|658[0n]|BOLD:AAB1086
Alphomelon arecaphileDHJ01|[790]|DHJPAR0060128|Costa Rica|658[0n]|BOLD:AAB1086
Alphomelon arecaphileDHJ01|[791]|DHJPAR0059479|Costa Rica|658[0n]|BOLD:AAB1086
Alphomelon arecaphileDHJ01|[792]|DHJPAR0012854|Costa Rica|657[0n]|BOLD:AAB1086
Alphomelon arecaphileDHJ01|[793]|DHJPAR0004881|Costa Rica|657[1n]|BOLD:AAB1086
Alphomelon arecaphileDHJ01|[794]|DHJPAR0005055|Costa Rica|657[0n]|BOLD:AAB1086
Alphomelon arecaphileDHJ01|[795]|DHJPAR0004945|Costa Rica|657[2n]|BOLD:AAB1086
Alphomelon arecaphileDHJ01|[796]|DHJPAR0004809|Costa Rica|657[19n]
Alphomelon arecaphileDHJ01|[797]|DHJPAR0005032|Costa Rica|657[0n]|BOLD:AAB1086
Alphomelon arecaphileDHJ01|[798]|DHJPAR0047100|Costa Rica|658[0n]|BOLD:AAB1086
Alphomelon arecaphileDHJ01|[799]|DHJPAR0059489|Costa Rica|658[0n]|BOLD:AAB1086
Alphomelon arecaphileDHJ01|[800]|DHJPAR0047115|Costa Rica|658[0n]|BOLD:AAB1086
Alphomelon arecaphileDHJ01|[801]|DHJPAR0059469|Costa Rica|658[0n]|BOLD:AAB1086
Alphomelon arecaphileDHJ01|[802]|DHJPAR0005042|Costa Rica|657[1n]|BOLD:AAB1086
Alphomelon arecaphileDHJ01|[803]|DHJPAR0005023|Costa Rica|657[1n]|BOLD:AAB1086
Alphomelon arecaphileDHJ01|[804]|DHJPAR0056370|Costa Rica|661[0n]|BOLD:AAB1086
Alphomelon arecaphileDHJ01|[805]|BIOUG61902-F02|Costa Rica|654[0n]|BOLD:AAB1086
Alphomelon arecaphileDHJ01|[806]|DHJPAR0005051|Costa Rica|657[1n]|BOLD:AAB1086
Alphomelon arecaphileDHJ01|[807]|DHJPAR0005030|Costa Rica|556[0n]|BOLD:AAB1086
Alphomelon arecaphileDHJ01|[808]|DHJPAR0012709|Costa Rica|657[0n]|BOLD:AAB1086
Alphomelon arecaphileDHJ01|[809]|DHJPAR0002448|Costa Rica|657[0n]|BOLD:AAB1086
Alphomelon arecaphileDHJ01|[810]|DHJPAR0053712|Costa Rica|658[0n]|BOLD:AAB1086
Alphomelon arecaphileDHJ01|[811]|DHJPAR0020782|Costa Rica|657[0n]|BOLD:AAB1086
Alphomelon arecaphileDHJ01|[812]|DHJPAR0004901|Costa Rica|657[0n]|BOLD:AAB1086
Alphomelon arecaphileDHJ01|[813]|DHJPAR0004895|Costa Rica|657[0n]|BOLD:AAB1086
Alphomelon arecaphileDHJ01|[814]|DHJPAR0012402|Costa Rica|657[0n]|BOLD:AAB1086
Alphomelon arecaphileDHJ01|[815]|DHJPAR0011848|Costa Rica|657[0n]|BOLD:AAB1086
Alphomelon arecaphileDHJ01|[816]|DHJPAR0065170|Costa Rica|638[0n]|BOLD:AAB1086
Alphomelon arecaphileDHJ02|[817]|DHJPAR0058986|Costa Rica|661[0n]|BOLD:AAB0787
Alphomelon arecaphileDHJ02|[818]|DHJPAR0058953|Costa Rica|660[0n]|BOLD:AAB0787
Alphomelon arecaphileDHJ02|[819]|DHJPAR0053105|Costa Rica|657[0n]|BOLD:AAB0787
Alphomelon arecaphileDHJ02|[820]|DHJPAR0054626|Costa Rica|658[0n]|BOLD:AAB0787
Alphomelon arecaphileDHJ02|[821]|DHJPAR0038970|Costa Rica|658[0n]|BOLD:AAB0787
Alphomelon arecaphileDHJ02|[822]|DHJPAR0047129|Costa Rica|658[0n]|BOLD:AAB0787
Alphomelon arecaphileDHJ02|[823]|DHJPAR0052972|Costa Rica|658[0n]|BOLD:AAB0787
Alphomelon arecaphileDHJ02|[824]|DHJPAR0058917|Costa Rica|610[0n]|BOLD:AAB0787
Alphomelon arecaphileDHJ02|[825]|DHJPAR0031104|Costa Rica|617[0n]|BOLD:AAB0787
Alphomelon arecaphileDHJ02|[826]|DHJPAR0034224|Costa Rica|630[1n]|BOLD:AAB0787
Alphomelon arecaphileDHJ02|[827]|DHJPAR0012394|Costa Rica|618[0n]|BOLD:AAB0787
Alphomelon arecaphileDHJ02|[828]|DHJPAR0039872|Costa Rica|627[0n]|BOLD:AAB0787
Alphomelon arecaphileDHJ02|[829]|DHJPAR0002476|Costa Rica|627[0n]|BOLD:AAB0787
Alphomelon arecaphileDHJ02|[830]|DHJPAR0020917|Costa Rica|633[0n]|BOLD:AAB0787
Alphomelon arecaphileDHJ02|[831]|DHJPAR0034203|Costa Rica|658[0n]|BOLD:AAB0787
Alphomelon arecaphileDHJ02|[832]|DHJPAR0060177|Costa Rica|658[0n]|BOLD:AAB0787
Alphomelon arecaphileDHJ02|[833]|DHJPAR0058956|Costa Rica|660[0n]|BOLD:AAB0787
Alphomelon arecaphileDHJ02|[834]|DHJPAR0004920|Costa Rica|657[0n]|BOLD:AAB0787
Alphomelon arecaphileDHJ02|[835]|DHJPAR0004928|Costa Rica|657[0n]|BOLD:AAB0787
Alphomelon arecaphileDHJ02|[836]|DHJPAR0012409|Costa Rica|657[0n]|BOLD:AAB0787
Alphomelon arecaphileDHJ02|[837]|DHJPAR0004914|Costa Rica|657[0n]|BOLD:AAB0787
Alphomelon arecaphileDHJ02|[838]|DHJPAR0062588|Costa Rica|658[0n]|BOLD:AAB0787
Alphomelon arecaphileDHJ02|[839]|DHJPAR0004653|Costa Rica|657[0n]|BOLD:AAB0787
Alphomelon arecaphileDHJ02|[840]|DHJPAR0002485|Costa Rica|657[0n]|BOLD:AAB0787
Alphomelon arecaphileDHJ02|[841]|DHJPAR0060239|Costa Rica|658[0n]|BOLD:AAB0787
Alphomelon arecaphileDHJ02|[842]|DHJPAR0062584|Costa Rica|658[0n]|BOLD:AAB0787
Alphomelon arecaphileDHJ02|[843]|DHJPAR0005034|Costa Rica|657[0n]|BOLD:AAB0787
Alphomelon arecaphileDHJ02|[844]|DHJPAR0058972|Costa Rica|660[0n]|BOLD:AAB0787
Alphomelon arecaphileDHJ02|[845]|DHJPAR0059478|Costa Rica|658[0n]|BOLD:AAB0787
Alphomelon arecaphileDHJ02|[846]|DHJPAR0012696|Costa Rica|657[0n]|BOLD:AAB0787
Alphomelon arecaphileDHJ02|[847]|DHJPAR0012879|Costa Rica|657[2n]|BOLD:AAB0787
Alphomelon arecaphileDHJ02|[848]|DHJPAR0030711|Costa Rica|636[0n]|BOLD:AAB0787
Alphomelon arecaphileDHJ02|[849]|DHJPAR0034208|Costa Rica|621[0n]|BOLD:AAB0787
Alphomelon arecaphileDHJ02|[850]|DHJPAR0030718|Costa Rica|573[0n]|BOLD:AAB0787
Alphomelon arecaphileDHJ02|[851]|DHJPAR0041792|Costa Rica|566[0n]|BOLD:AAB0787
Alphomelon arecaphileDHJ02|[852]|DHJPAR0058218|Costa Rica|626[0n]|BOLD:AAB0787
Alphomelon arecaphileDHJ02|[853]|DHJPAR0060185|Costa Rica|634[0n]|BOLD:AAB0787
Alphomelon arecaphileDHJ02|[854]|DHJPAR0004669|Costa Rica|655[0n]|BOLD:AAB0787
Alphomelon arecaphileDHJ02|[855]|DHJPAR0004947|Costa Rica|656[3n]|BOLD:AAB0787
Alphomelon arecaphileDHJ02|[856]|DHJPAR0011844|Costa Rica|656[1n]|BOLD:AAB0787
Alphomelon arecaphileDHJ02|[857]|DHJPAR0038131|Costa Rica|658[0n]|BOLD:AAB0787
Alphomelon arecaphileDHJ02|[858]|DHJPAR0038130|Costa Rica|658[0n]|BOLD:AAB0787
Alphomelon arecaphileDHJ02|[859]|DHJPAR0038135|Costa Rica|658[0n]|BOLD:AAB0787
Alphomelon arecaphileDHJ02|[860]|DHJPAR0012870|Costa Rica|657[0n]|BOLD:AAB0787
Alphomelon arecaphileDHJ02|[861]|DHJPAR0012873|Costa Rica|657[0n]|BOLD:AAB0787
Alphomelon arecaphileDHJ02|[862]|DHJPAR0057779|Costa Rica|661[0n]|BOLD:AAB0787
Alphomelon arecaphileDHJ02|[863]|DHJPAR0058840|Costa Rica|658[0n]|BOLD:AAB0787
Alphomelon arecaphileDHJ02|[864]|DHJPAR0058843|Costa Rica|658[0n]|BOLD:AAB0787
Alphomelon arecaphileDHJ02|[865]|DHJPAR0058939|Costa Rica|658[0n]|BOLD:AAB0787
Alphomelon arecaphileDHJ02|[866]|DHJPAR0058942|Costa Rica|658[0n]|BOLD:AAB0787
Alphomelon arecaphileDHJ02|[867]|DHJPAR0058948|Costa Rica|657[0n]|BOLD:AAB0787
Alphomelon arecaphileDHJ02|[868]|DHJPAR0060234|Costa Rica|658[0n]|BOLD:AAB0787
Alphomelon arecaphileDHJ02|[869]|DHJPAR0058981|Costa Rica|661[0n]|BOLD:AAB0787
Alphomelon arecaphileDHJ02|[870]|DHJPAR0058982|Costa Rica|661[0n]|BOLD:AAB0787

Alphomelon arecaphileDHJ02|[868]|DHJPAR0060234|Costa Rica|658[0n]|BOLD:AAB0787
Alphomelon arecaphileDHJ02|[869]|DHJPAR0058981|Costa Rica|661[0n]|BOLD:AAB0787
Alphomelon arecaphileDHJ02|[870]|DHJPAR0058982|Costa Rica|661[0n]|BOLD:AAB0787
Alphomelon arecaphileDHJ02|[871]|DHJPAR0051778|Costa Rica|660[0n]|BOLD:AAB0787
Alphomelon arecaphileDHJ02|[872]|DHJPAR0004907|Costa Rica|657[0n]|BOLD:AAB0787
Alphomelon arecaphileDHJ02|[873]|DHJPAR0058957|Costa Rica|661[0n]|BOLD:AAB0787
Alphomelon arecaphileDHJ02|[874]|DHJPAR0058195|Costa Rica|658[0n]|BOLD:AAB0787
Alphomelon arecaphileDHJ02|[875]|DHJPAR0005020|Costa Rica|657[0n]|BOLD:AAB0787
Alphomelon arecaphileDHJ02|[876]|DHJPAR0039878|Costa Rica|658[0n]|BOLD:AAB0787
Alphomelon arecaphileDHJ02|[877]|DHJPAR0041775|Costa Rica|658[0n]|BOLD:AAB0787
Alphomelon arecaphileDHJ02|[878]|DHJPAR0041794|Costa Rica|658[0n]|BOLD:AAB0787
Alphomelon arecaphileDHJ02|[879]|DHJPAR0004926|Costa Rica|657[0n]|BOLD:AAB0787
Alphomelon arecaphileDHJ02|[880]|DHJPAR0058895|Costa Rica|658[0n]|BOLD:AAB0787
Alphomelon arecaphileDHJ02|[881]|DHJPAR0051032|Costa Rica|657[0n]|BOLD:AAB0787
Alphomelon arecaphileDHJ02|[882]|DHJPAR0051038|Costa Rica|658[0n]|BOLD:AAB0787
Alphomelon arecaphileDHJ02|[883]|DHJPAR0012705|Costa Rica|657[0n]|BOLD:AAB0787
Alphomelon arecaphileDHJ02|[884]|DHJPAR0020200|Costa Rica|657[1n]|BOLD:AAB0787
Alphomelon arecaphileDHJ02|[885]|DHJPAR0005066|Costa Rica|656[0n]|BOLD:AAB0787
Alphomelon arecaphileDHJ02|[886]|DHJPAR0005018|Costa Rica|656[0n]|BOLD:AAB0787
Alphomelon arecaphileDHJ02|[887]|DHJPAR0004916|Costa Rica|656[0n]|BOLD:AAB0787
Alphomelon arecaphileDHJ02|[888]|DHJPAR0004911|Costa Rica|656[0n]|BOLD:AAB0787
Alphomelon arecaphileDHJ02|[889]|DHJPAR0004883|Costa Rica|656[0n]|BOLD:AAB0787
Alphomelon arecaphileDHJ02|[890]|DHJPAR0055257|Costa Rica|657[0n]|BOLD:AAB0787
Alphomelon melanoscelis|[891]|DHJPAR0051219|Costa Rica|618[1n]|BOLD:AAB6733
Alphomelon melanoscelis|[892]|DHJPAR0004904|Costa Rica|627[0n]|BOLD:AAB6733
Alphomelon melanoscelis|[893]|DHJPAR0057763|Costa Rica|623[0n]|BOLD:AAB6733
Alphomelon melanoscelis|[894]|DHJPAR0004890|Costa Rica|623[0n]|BOLD:AAB6733
Alphomelon melanoscelis|[895]|DHJPAR0002452|Costa Rica|419[0n]|BOLD:AAB6733
Alphomelon melanoscelis|[896]|DHJPAR0060618|Costa Rica|658[0n]|BOLD:AAB6733
Alphomelon melanoscelis|[897]|DHJPAR0034243|Costa Rica|658[0n]|BOLD:AAB6733
Alphomelon melanoscelis|[898]|DHJPAR0055274|Costa Rica|658[0n]|BOLD:AAB6733
Alphomelon melanoscelis|[899]|DHJPAR0002395|Costa Rica|657[0n]|BOLD:AAB6733
Alphomelon melanoscelis|[900]|DHJPAR0058834|Costa Rica|658[0n]|BOLD:AAB6733
Alphomelon melanoscelis|[901]|DHJPAR0058841|Costa Rica|658[0n]|BOLD:AAB6733
Alphomelon melanoscelis|[902]|DHJPAR0058842|Costa Rica|658[0n]|BOLD:AAB6733
Alphomelon melanoscelis|[903]|DHJPAR0058844|Costa Rica|658[0n]|BOLD:AAB6733
Alphomelon melanoscelis|[904]|DHJPAR0058848|Costa Rica|658[0n]|BOLD:AAB6733
Alphomelon melanoscelis|[905]|DHJPAR0058866|Costa Rica|658[0n]|BOLD:AAB6733
Alphomelon melanoscelis|[906]|DHJPAR0058888|Costa Rica|658[0n]|BOLD:AAB6733
Alphomelon melanoscelis|[907]|DHJPAR0060625|Costa Rica|658[0n]|BOLD:AAB6733
Alphomelon melanoscelis|[908]|DHJPAR0058941|Costa Rica|658[0n]|BOLD:AAB6733
Alphomelon melanoscelis|[909]|DHJPAR0058946|Costa Rica|658[0n]|BOLD:AAB6733
Alphomelon melanoscelis|[910]|DHJPAR0058954|Costa Rica|661[0n]|BOLD:AAB6733
Alphomelon melanoscelis|[911]|DHJPAR0058964|Costa Rica|661[0n]|BOLD:AAB6733
Alphomelon melanoscelis|[912]|DHJPAR0058980|Costa Rica|661[0n]|BOLD:AAB6733
Alphomelon melanoscelis|[913]|DHJPAR0058983|Costa Rica|661[0n]|BOLD:AAB6733
Alphomelon melanoscelis|[914]|DHJPAR0004899|Costa Rica|657[0n]|BOLD:AAB6733
Alphomelon melanoscelis|[915]|DHJPAR0004908|Costa Rica|657[0n]|BOLD:AAB6733
Alphomelon melanoscelis|[916]|DHJPAR0004913|Costa Rica|657[0n]|BOLD:AAB6733
Alphomelon melanoscelis|[917]|DHJPAR0004918|Costa Rica|657[0n]|BOLD:AAB6733
Alphomelon melanoscelis|[918]|DHJPAR0012412|Costa Rica|657[0n]|BOLD:AAB6733
Alphomelon melanoscelis|[919]|DHJPAR0012419|Costa Rica|657[0n]|BOLD:AAB6733
Alphomelon melanoscelis|[920]|DHJPAR0012425|Costa Rica|657[0n]|BOLD:AAB6733
Alphomelon melanoscelis|[921]|DHJPAR0060630|Costa Rica|658[0n]|BOLD:AAB6733
Alphomelon melanoscelis|[922]|DHJPAR0020291|Costa Rica|657[0n]|BOLD:AAB6733
Alphomelon melanoscelis|[923]|DHJPAR0061666|Costa Rica|658[0n]|BOLD:AAB6733
Alphomelon melanoscelis|[924]|DHJPAR0058177|Costa Rica|658[0n]|BOLD:AAB6733
Alphomelon melanoscelis|[925]|DHJPAR0058156|Costa Rica|658[0n]|BOLD:AAB6733
Alphomelon melanoscelis|[926]|DHJPAR0060232|Costa Rica|658[0n]|BOLD:AAB6733
Alphomelon melanoscelis|[927]|DHJPAR0057502|Costa Rica|658[0n]|BOLD:AAB6733
Alphomelon melanoscelis|[928]|DHJPAR0041766|Costa Rica|658[0n]|BOLD:AAB6733
Alphomelon melanoscelis|[929]|DHJPAR0057514|Costa Rica|658[0n]|BOLD:AAB6733
Alphomelon melanoscelis|[930]|DHJPAR0041692|Costa Rica|658[0n]|BOLD:AAB6733
Alphomelon melanoscelis|[931]|DHJPAR0058213|Costa Rica|658[0n]|BOLD:AAB6733
Alphomelon melanoscelis|[932]|DHJPAR0041714|Costa Rica|658[0n]|BOLD:AAB6733
Alphomelon melanoscelis|[933]|DHJPAR0041739|Costa Rica|658[0n]|BOLD:AAB6733
Alphomelon melanoscelis|[934]|DHJPAR0058851|Costa Rica|658[0n]|BOLD:AAB6733
Alphomelon melanoscelis|[935]|DHJPAR0041756|Costa Rica|658[0n]|BOLD:AAB6733
Alphomelon melanoscelis|[936]|DHJPAR0058861|Costa Rica|658[0n]|BOLD:AAB6733
Alphomelon melanoscelis|[937]|DHJPAR0061623|Costa Rica|658[0n]|BOLD:AAB6733
Alphomelon melanoscelis|[938]|DHJPAR0058168|Costa Rica|658[0n]|BOLD:AAB6733
Alphomelon melanoscelis|[939]|DHJPAR0058219|Costa Rica|658[0n]|BOLD:AAB6733
Alphomelon melanoscelis|[940]|DHJPAR0060617|Costa Rica|658[0n]|BOLD:AAB6733
Alphomelon melanoscelis|[941]|DHJPAR0059480|Costa Rica|658[0n]|BOLD:AAB6733
Alphomelon melanoscelis|[942]|DHJPAR0058936|Costa Rica|658[0n]|BOLD:AAB6733
Alphomelon melanoscelis|[943]|DHJPAR0061628|Costa Rica|658[0n]|BOLD:AAB6733
Alphomelon melanoscelis|[944]|DHJPAR0055445|Costa Rica|658[0n]|BOLD:AAB6733
Alphomelon melanoscelis|[945]|DHJPAR0057770|Costa Rica|658[0n]|BOLD:AAB6733
Alphomelon melanoscelis|[946]|DHJPAR0012404|Costa Rica|657[0n]|BOLD:AAB6733
Alphomelon melanoscelis|[947]|DHJPAR0020288|Costa Rica|657[1n]|BOLD:AAB6733
Alphomelon melanoscelis|[948]|DHJPAR0012872|Costa Rica|656[2n]|BOLD:AAB6733
Alphomelon melanoscelis|[949]|DHJPAR0004929|Costa Rica|657[1n]|BOLD:AAB6733
Alphomelon melanoscelis|[950]|DHJPAR0034227|Costa Rica|612[0n]|BOLD:AAB6733
Alphomelon melanoscelis|[951]|DHJPAR0005035|Costa Rica|656[2n]|BOLD:AAB6733
Alphomelon melanoscelis|[952]|DHJPAR0034230|Costa Rica|651[0n]|BOLD:AAB6733
Alphomelon citroloma|[953]|BIOUG92282-B07|Costa Rica|656[0n]|BOLD:AEN2538
Hymenoptera|[954]|BIOUG92355-E04|Costa Rica|696[0n]
Alphomelon citroloma|[955]|BIOUG94211-E06|Costa Rica|654[0n]|BOLD:AEN2538
Alphomelon citroloma|[956]|BIOUG92281-F02|Costa Rica|656[0n]|BOLD:AEN2538
Hymenoptera|[957]|BIOUG96059-B09|Costa Rica|652[0n]|BOLD:AEN2538
Hymenoptera|[958]|BIOUG92367-F10|Costa Rica|654[0n]|BOLD:AEN2538
Alphomelon citroloma|[959]|BIOUG95212-F10|Costa Rica|653[0n]|BOLD:AEN2538
Alphomelon Deans29|[960]|DHJPAR0039480|Costa Rica|658[0n]|BOLD:AAB8584
Alphomelon Deans29|[961]|DHJPAR0027684|Costa Rica|627[0n]|BOLD:AAB8584
Alphomelon Deans29|[962]|DHJPAR0060266|Costa Rica|658[0n]|BOLD:AAB8584
Alphomelon Deans29|[963]|DHJPAR0002459|Costa Rica|657[0n]|BOLD:AAB8584
Alphomelon Deans29|[964]|DHJPAR0027666|Costa Rica|627[0n]|BOLD:AAB8584
Alphomelon Deans29|[965]|DHJPAR0013793|Costa Rica|600[0n]|BOLD:AAB8584
Alphomelon Deans29|[966]|DHJPAR0013794|Costa Rica|596[0n]|BOLD:AAB8584
Alphomelon Deans29|[967]|DHJPAR0042479|Costa Rica|658[0n]|BOLD:AAB8584

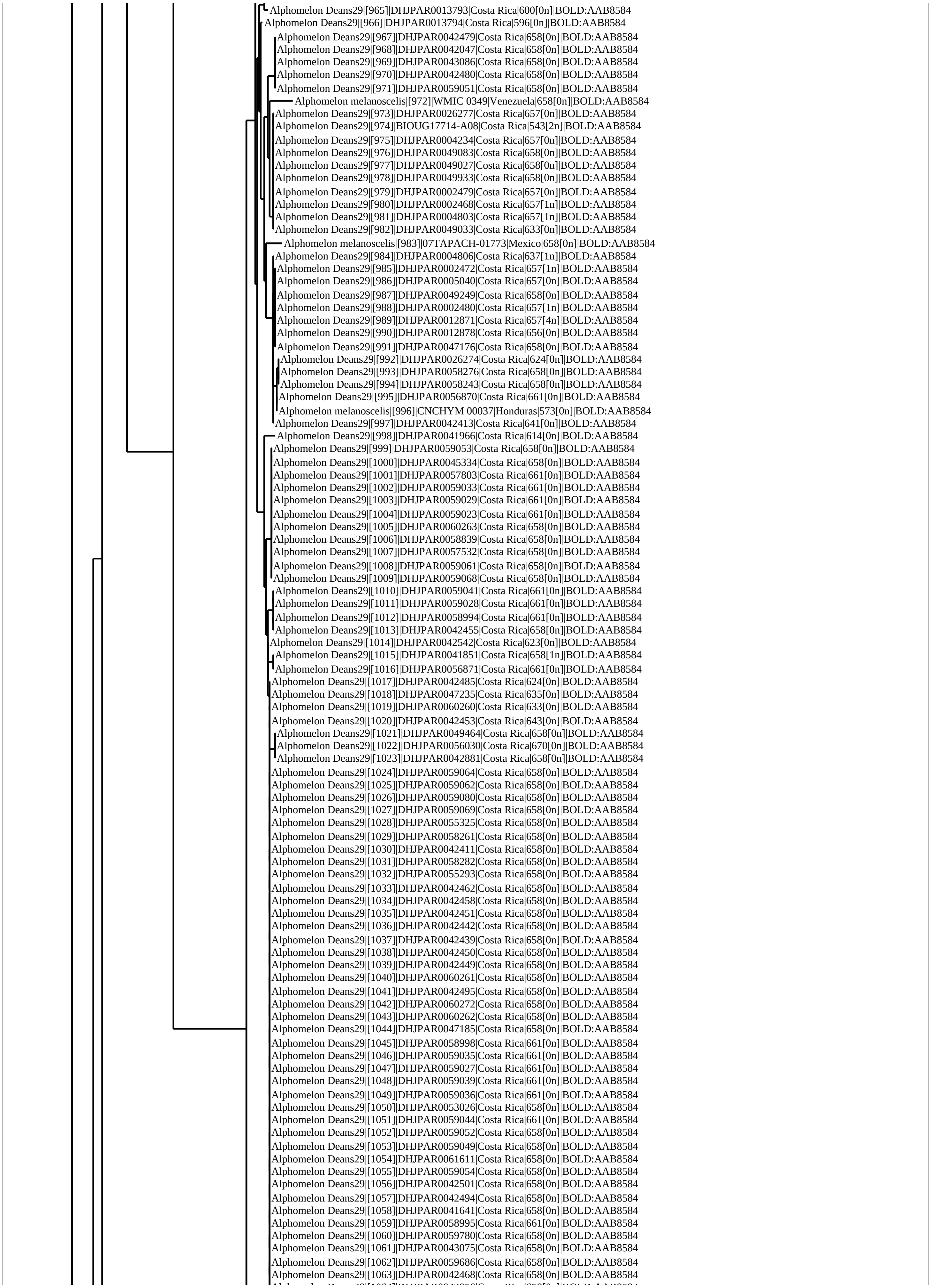
Alphomelon Deans29|[965]|DHJPAR0013793|Costa Rica|600[0n]|BOLD:AAB8584
Alphomelon Deans29|[966]|DHJPAR0013794|Costa Rica|596[0n]|BOLD:AAB8584
Alphomelon Deans29|[967]|DHJPAR0042479|Costa Rica|658[0n]|BOLD:AAB8584
Alphomelon Deans29|[968]|DHJPAR0042047|Costa Rica|658[0n]|BOLD:AAB8584
Alphomelon Deans29|[969]|DHJPAR0043086|Costa Rica|658[0n]|BOLD:AAB8584
Alphomelon Deans29|[970]|DHJPAR0042480|Costa Rica|658[0n]|BOLD:AAB8584
Alphomelon Deans29|[971]|DHJPAR0059051|Costa Rica|658[0n]|BOLD:AAB8584
Alphomelon melanoscelis|[972]|WMIC 0349|Venezuela|658[0n]|BOLD:AAB8584
Alphomelon Deans29|[973]|DHJPAR0026277|Costa Rica|657[0n]|BOLD:AAB8584
Alphomelon Deans29|[974]|BIOUG17714-A08|Costa Rica|543[2n]|BOLD:AAB8584
Alphomelon Deans29|[975]|DHJPAR0004234|Costa Rica|657[0n]|BOLD:AAB8584
Alphomelon Deans29|[976]|DHJPAR0049083|Costa Rica|658[0n]|BOLD:AAB8584
Alphomelon Deans29|[977]|DHJPAR0049027|Costa Rica|658[0n]|BOLD:AAB8584
Alphomelon Deans29|[978]|DHJPAR0049933|Costa Rica|658[0n]|BOLD:AAB8584
Alphomelon Deans29|[979]|DHJPAR0002479|Costa Rica|657[0n]|BOLD:AAB8584
Alphomelon Deans29|[980]|DHJPAR0002468|Costa Rica|657[1n]|BOLD:AAB8584
Alphomelon Deans29|[981]|DHJPAR0004803|Costa Rica|657[1n]|BOLD:AAB8584
Alphomelon Deans29|[982]|DHJPAR0049033|Costa Rica|633[0n]|BOLD:AAB8584
Alphomelon melanoscelis|[983]|07TAPACH-01773|Mexico|658[0n]|BOLD:AAB8584
Alphomelon Deans29|[984]|DHJPAR0004806|Costa Rica|637[1n]|BOLD:AAB8584
Alphomelon Deans29|[985]|DHJPAR0002472|Costa Rica|657[1n]|BOLD:AAB8584
Alphomelon Deans29|[986]|DHJPAR0005040|Costa Rica|657[0n]|BOLD:AAB8584
Alphomelon Deans29|[987]|DHJPAR0049249|Costa Rica|658[0n]|BOLD:AAB8584
Alphomelon Deans29|[988]|DHJPAR0002480|Costa Rica|657[1n]|BOLD:AAB8584
Alphomelon Deans29|[989]|DHJPAR0012871|Costa Rica|657[4n]|BOLD:AAB8584
Alphomelon Deans29|[990]|DHJPAR0012878|Costa Rica|656[0n]|BOLD:AAB8584
Alphomelon Deans29|[991]|DHJPAR0047176|Costa Rica|658[0n]|BOLD:AAB8584
Alphomelon Deans29|[992]|DHJPAR0026274|Costa Rica|624[0n]|BOLD:AAB8584
Alphomelon Deans29|[993]|DHJPAR0058276|Costa Rica|658[0n]|BOLD:AAB8584
Alphomelon Deans29|[994]|DHJPAR0058243|Costa Rica|658[0n]|BOLD:AAB8584
Alphomelon Deans29|[995]|DHJPAR0056870|Costa Rica|661[0n]|BOLD:AAB8584
Alphomelon melanoscelis|[996]|CNCHYM 00037|Honduras|573[0n]|BOLD:AAB8584
Alphomelon Deans29|[997]|DHJPAR0042413|Costa Rica|641[0n]|BOLD:AAB8584
Alphomelon Deans29|[998]|DHJPAR0041966|Costa Rica|614[0n]|BOLD:AAB8584
Alphomelon Deans29|[999]|DHJPAR0059053|Costa Rica|658[0n]|BOLD:AAB8584
Alphomelon Deans29|[1000]|DHJPAR0045334|Costa Rica|658[0n]|BOLD:AAB8584
Alphomelon Deans29|[1001]|DHJPAR0057803|Costa Rica|661[0n]|BOLD:AAB8584
Alphomelon Deans29|[1002]|DHJPAR0059033|Costa Rica|661[0n]|BOLD:AAB8584
Alphomelon Deans29|[1003]|DHJPAR0059029|Costa Rica|661[0n]|BOLD:AAB8584
Alphomelon Deans29|[1004]|DHJPAR0059023|Costa Rica|661[0n]|BOLD:AAB8584
Alphomelon Deans29|[1005]|DHJPAR0060263|Costa Rica|658[0n]|BOLD:AAB8584
Alphomelon Deans29|[1006]|DHJPAR0058839|Costa Rica|658[0n]|BOLD:AAB8584
Alphomelon Deans29|[1007]|DHJPAR0057532|Costa Rica|658[0n]|BOLD:AAB8584
Alphomelon Deans29|[1008]|DHJPAR0059061|Costa Rica|658[0n]|BOLD:AAB8584
Alphomelon Deans29|[1009]|DHJPAR0059068|Costa Rica|658[0n]|BOLD:AAB8584
Alphomelon Deans29|[1010]|DHJPAR0059041|Costa Rica|661[0n]|BOLD:AAB8584
Alphomelon Deans29|[1011]|DHJPAR0059028|Costa Rica|661[0n]|BOLD:AAB8584
Alphomelon Deans29|[1012]|DHJPAR0058994|Costa Rica|661[0n]|BOLD:AAB8584
Alphomelon Deans29|[1013]|DHJPAR0042455|Costa Rica|658[0n]|BOLD:AAB8584
Alphomelon Deans29|[1014]|DHJPAR0042542|Costa Rica|623[0n]|BOLD:AAB8584
Alphomelon Deans29|[1015]|DHJPAR0041851|Costa Rica|658[1n]|BOLD:AAB8584
Alphomelon Deans29|[1016]|DHJPAR0056871|Costa Rica|661[0n]|BOLD:AAB8584
Alphomelon Deans29|[1017]|DHJPAR0042485|Costa Rica|624[0n]|BOLD:AAB8584
Alphomelon Deans29|[1018]|DHJPAR0047235|Costa Rica|635[0n]|BOLD:AAB8584
Alphomelon Deans29|[1019]|DHJPAR0060260|Costa Rica|633[0n]|BOLD:AAB8584
Alphomelon Deans29|[1020]|DHJPAR0042453|Costa Rica|643[0n]|BOLD:AAB8584
Alphomelon Deans29|[1021]|DHJPAR0049464|Costa Rica|658[0n]|BOLD:AAB8584
Alphomelon Deans29|[1022]|DHJPAR0056030|Costa Rica|670[0n]|BOLD:AAB8584
Alphomelon Deans29|[1023]|DHJPAR0042881|Costa Rica|658[0n]|BOLD:AAB8584
Alphomelon Deans29|[1024]|DHJPAR0059064|Costa Rica|658[0n]|BOLD:AAB8584
Alphomelon Deans29|[1025]|DHJPAR0059062|Costa Rica|658[0n]|BOLD:AAB8584
Alphomelon Deans29|[1026]|DHJPAR0059080|Costa Rica|658[0n]|BOLD:AAB8584
Alphomelon Deans29|[1027]|DHJPAR0059069|Costa Rica|658[0n]|BOLD:AAB8584
Alphomelon Deans29|[1028]|DHJPAR0055325|Costa Rica|658[0n]|BOLD:AAB8584
Alphomelon Deans29|[1029]|DHJPAR0058261|Costa Rica|658[0n]|BOLD:AAB8584
Alphomelon Deans29|[1030]|DHJPAR0042411|Costa Rica|658[0n]|BOLD:AAB8584
Alphomelon Deans29|[1031]|DHJPAR0058282|Costa Rica|658[0n]|BOLD:AAB8584
Alphomelon Deans29|[1032]|DHJPAR0055293|Costa Rica|658[0n]|BOLD:AAB8584
Alphomelon Deans29|[1033]|DHJPAR0042462|Costa Rica|658[0n]|BOLD:AAB8584
Alphomelon Deans29|[1034]|DHJPAR0042458|Costa Rica|658[0n]|BOLD:AAB8584
Alphomelon Deans29|[1035]|DHJPAR0042451|Costa Rica|658[0n]|BOLD:AAB8584
Alphomelon Deans29|[1036]|DHJPAR0042442|Costa Rica|658[0n]|BOLD:AAB8584
Alphomelon Deans29|[1037]|DHJPAR0042439|Costa Rica|658[0n]|BOLD:AAB8584
Alphomelon Deans29|[1038]|DHJPAR0042450|Costa Rica|658[0n]|BOLD:AAB8584
Alphomelon Deans29|[1039]|DHJPAR0042449|Costa Rica|658[0n]|BOLD:AAB8584
Alphomelon Deans29|[1040]|DHJPAR0060261|Costa Rica|658[0n]|BOLD:AAB8584
Alphomelon Deans29|[1041]|DHJPAR0042495|Costa Rica|658[0n]|BOLD:AAB8584
Alphomelon Deans29|[1042]|DHJPAR0060272|Costa Rica|658[0n]|BOLD:AAB8584
Alphomelon Deans29|[1043]|DHJPAR0060262|Costa Rica|658[0n]|BOLD:AAB8584
Alphomelon Deans29|[1044]|DHJPAR0047185|Costa Rica|658[0n]|BOLD:AAB8584
Alphomelon Deans29|[1045]|DHJPAR0058998|Costa Rica|661[0n]|BOLD:AAB8584
Alphomelon Deans29|[1046]|DHJPAR0059035|Costa Rica|661[0n]|BOLD:AAB8584
Alphomelon Deans29|[1047]|DHJPAR0059027|Costa Rica|661[0n]|BOLD:AAB8584
Alphomelon Deans29|[1048]|DHJPAR0059039|Costa Rica|661[0n]|BOLD:AAB8584
Alphomelon Deans29|[1049]|DHJPAR0059036|Costa Rica|661[0n]|BOLD:AAB8584
Alphomelon Deans29|[1050]|DHJPAR0053026|Costa Rica|658[0n]|BOLD:AAB8584
Alphomelon Deans29|[1051]|DHJPAR0059044|Costa Rica|661[0n]|BOLD:AAB8584
Alphomelon Deans29|[1052]|DHJPAR0059052|Costa Rica|658[0n]|BOLD:AAB8584
Alphomelon Deans29|[1053]|DHJPAR0059049|Costa Rica|658[0n]|BOLD:AAB8584
Alphomelon Deans29|[1054]|DHJPAR0061611|Costa Rica|658[0n]|BOLD:AAB8584
Alphomelon Deans29|[1055]|DHJPAR0059054|Costa Rica|658[0n]|BOLD:AAB8584
Alphomelon Deans29|[1056]|DHJPAR0042501|Costa Rica|658[0n]|BOLD:AAB8584
Alphomelon Deans29|[1057]|DHJPAR0042494|Costa Rica|658[0n]|BOLD:AAB8584
Alphomelon Deans29|[1058]|DHJPAR0041641|Costa Rica|658[0n]|BOLD:AAB8584
Alphomelon Deans29|[1059]|DHJPAR0058995|Costa Rica|661[0n]|BOLD:AAB8584
Alphomelon Deans29|[1060]|DHJPAR0059780|Costa Rica|658[0n]|BOLD:AAB8584
Alphomelon Deans29|[1061]|DHJPAR0043075|Costa Rica|658[0n]|BOLD:AAB8584
Alphomelon Deans29|[1062]|DHJPAR0059686|Costa Rica|658[0n]|BOLD:AAB8584
Alphomelon Deans29|[1063]|DHJPAR0042468|Costa Rica|658[0n]|BOLD:AAB8584

Alphomelon Deans29|[1061]|DHJPAR004307[illegible]|Costa Rica|658[0n]|BOLD:AAB8584
Alphomelon Deans29|[1062]|DHJPAR0059686|Costa Rica|658[0n]|BOLD:AAB8584
Alphomelon Deans29|[1063]|DHJPAR0042468|Costa Rica|658[0n]|BOLD:AAB8584
Alphomelon Deans29|[1064]|DHJPAR0042056|Costa Rica|658[0n]|BOLD:AAB8584
Alphomelon Deans29|[1065]|DHJPAR0042441|Costa Rica|658[0n]|BOLD:AAB8584
Alphomelon Deans29|[1066]|DHJPAR0043094|Costa Rica|658[0n]|BOLD:AAB8584
Alphomelon Deans29|[1067]|DHJPAR0043092|Costa Rica|658[0n]|BOLD:AAB8584
Alphomelon Deans29|[1068]|DHJPAR0043077|Costa Rica|658[0n]|BOLD:AAB8584
Alphomelon Deans29|[1069]|DHJPAR0043076|Costa Rica|658[0n]|BOLD:AAB8584
Alphomelon Deans29|[1070]|DHJPAR0043130|Costa Rica|658[0n]|BOLD:AAB8584
Alphomelon Deans29|[1071]|DHJPAR0043127|Costa Rica|658[0n]|BOLD:AAB8584
Alphomelon Deans29|[1072]|DHJPAR0043122|Costa Rica|658[0n]|BOLD:AAB8584
Alphomelon Deans29|[1073]|DHJPAR0043110|Costa Rica|658[0n]|BOLD:AAB8584
Alphomelon Deans29|[1074]|DHJPAR0043004|Costa Rica|658[0n]|BOLD:AAB8584
Alphomelon Deans29|[1075]|DHJPAR0041858|Costa Rica|658[0n]|BOLD:AAB8584
Alphomelon Deans29|[1076]|DHJPAR0042052|Costa Rica|658[0n]|BOLD:AAB8584
Alphomelon Deans29|[1077]|DHJPAR0043009|Costa Rica|658[0n]|BOLD:AAB8584
Alphomelon Deans29|[1078]|DHJPAR0042412|Costa Rica|658[0n]|BOLD:AAB8584
Alphomelon Deans29|[1079]|DHJPAR0043087|Costa Rica|658[0n]|BOLD:AAB8584
Alphomelon Deans29|[1080]|DHJPAR0043005|Costa Rica|658[0n]|BOLD:AAB8584
Alphomelon Deans29|[1081]|DHJPAR0042481|Costa Rica|658[0n]|BOLD:AAB8584
Alphomelon Deans29|[1082]|DHJPAR0042434|Costa Rica|658[0n]|BOLD:AAB8584
Alphomelon Deans29|[1083]|DHJPAR0060274|Costa Rica|658[0n]|BOLD:AAB8584
Alphomelon Deans29|[1084]|DHJPAR0060273|Costa Rica|658[0n]|BOLD:AAB8584
Alphomelon Deans29|[1085]|DHJPAR0060265|Costa Rica|658[0n]|BOLD:AAB8584
Alphomelon Deans29|[1086]|DHJPAR0049465|Costa Rica|658[0n]|BOLD:AAB8584
Alphomelon Deans29|[1087]|DHJPAR0042471|Costa Rica|658[0n]|BOLD:AAB8584
Alphomelon Deans29|[1088]|DHJPAR0042456|Costa Rica|658[0n]|BOLD:AAB8584
Alphomelon Deans29|[1089]|DHJPAR0059007|Costa Rica|661[0n]|BOLD:AAB8584
Alphomelon Deans29|[1090]|DHJPAR0042049|Costa Rica|658[0n]|BOLD:AAB8584
Alphomelon Deans29|[1091]|DHJPAR0061836|Costa Rica|658[0n]|BOLD:AAB8584
Alphomelon Deans29|[1092]|DHJPAR0043126|Costa Rica|658[0n]|BOLD:AAB8584
Alphomelon Deans29|[1093]|DHJPAR0061850|Costa Rica|658[0n]|BOLD:AAB8584
Alphomelon Deans29|[1094]|DHJPAR0042444|Costa Rica|658[0n]|BOLD:AAB8584
Alphomelon Deans29|[1095]|DHJPAR0042445|Costa Rica|658[0n]|BOLD:AAB8584
Alphomelon Deans29|[1096]|DHJPAR0055526|Costa Rica|658[0n]|BOLD:AAB8584
Alphomelon Deans29|[1097]|DHJPAR0042051|Costa Rica|658[0n]|BOLD:AAB8584
Alphomelon Deans29|[1098]|DHJPAR0042447|Costa Rica|658[0n]|BOLD:AAB8584
Alphomelon Deans29|[1099]|DHJPAR0055561|Costa Rica|661[0n]|BOLD:AAB8584
Alphomelon Deans29|[1100]|DHJPAR0042457|Costa Rica|658[0n]|BOLD:AAB8584
Alphomelon Deans29|[1101]|DHJPAR0059034|Costa Rica|661[0n]|BOLD:AAB8584
Alphomelon Deans29|[1102]|DHJPAR0042459|Costa Rica|658[0n]|BOLD:AAB8584
Alphomelon Deans29|[1103]|DHJPAR0042467|Costa Rica|658[0n]|BOLD:AAB8584
Alphomelon Deans29|[1104]|DHJPAR0059040|Costa Rica|661[0n]|BOLD:AAB8584
Alphomelon Deans29|[1105]|DHJPAR0042436|Costa Rica|658[0n]|BOLD:AAB8584
Alphomelon Deans29|[1106]|DHJPAR0041852|Costa Rica|329[3n]
Alphomelon Deans29|[1107]|DHJPAR0041845|Costa Rica|530[0n]|BOLD:AAB8584
Alphomelon Deans29|[1108]|DHJPAR0059776|Costa Rica|658[0n]|BOLD:AAB8584
Alphomelon Deans29|[1109]|DHJPAR0049916|Costa Rica|658[0n]|BOLD:AAB8584
Alphomelon Deans29|[1110]|DHJPAR0042486|Costa Rica|658[0n]|BOLD:AAB8584
Alphomelon melanoscelis|[1111]|BIOUG24734-D06|Argentina|585[0n]|BOLD:AAB8584
Alphomelon melanoscelis|[1112]|CNCHYM 00034|Colombia|442[0n]|BOLD:AAB8584
Alphomelon Deans03|[1113]|DHJPAR0013792|Costa Rica|596[0n]|BOLD:ADJ6568
Alphomelon Deans03|[1114]|DHJPAR0058247|Costa Rica|658[0n]|BOLD:ADJ6568
Alphomelon Deans03|[1115]|DHJPAR0058277|Costa Rica|621[0n]|BOLD:ADJ6568
Alphomelon Deans03|[1116]|DHJPAR0058259|Costa Rica|658[0n]|BOLD:ADJ6568
Alphomelon Deans03|[1117]|DHJPAR0004804|Costa Rica|554[0n]|BOLD:ADJ6568
Alphomelon Deans03|[1118]|DHJPAR0047189|Costa Rica|658[0n]|BOLD:ADJ6568
Alphomelon Deans03|[1119]|DHJPAR0058258|Costa Rica|658[0n]|BOLD:ADJ6568
Alphomelon Deans03|[1120]|DHJPAR0059048|Costa Rica|658[0n]|BOLD:ADJ6568
Alphomelon Deans03|[1121]|DHJPAR0058274|Costa Rica|658[0n]|BOLD:ADJ6568
Alphomelon Deans03|[1122]|DHJPAR0058271|Costa Rica|658[0n]|BOLD:ADJ6568
Alphomelon Deans03|[1123]|DHJPAR0058269|Costa Rica|658[0n]|BOLD:ADJ6568
Alphomelon Deans03|[1124]|DHJPAR0020103|Costa Rica|578[1n]|BOLD:ADJ6568
Alphomelon Deans03|[1125]|DHJPAR0002481|Costa Rica|614[0n]|BOLD:ADJ6568
Alphomelon Deans03|[1126]|DHJPAR0004807|Costa Rica|577[2n]|BOLD:ADJ6568
Alphomelon Deans03|[1127]|DHJPAR0025344|Costa Rica|645[0n]|BOLD:ADJ6568
Alphomelon Deans03|[1128]|DHJPAR0025843|Costa Rica|657[0n]|BOLD:ADJ6568
Alphomelon Deans03|[1129]|DHJPAR0049260|Costa Rica|658[0n]|BOLD:ADJ6568
Alphomelon Deans03|[1130]|DHJPAR0055291|Costa Rica|658[0n]|BOLD:ADJ6568
Alphomelon Deans03|[1131]|DHJPAR0049212|Costa Rica|658[0n]|BOLD:ADJ6568
Alphomelon Deans03|[1132]|DHJPAR0049254|Costa Rica|658[0n]|BOLD:ADJ6568
Alphomelon Deans03|[1133]|DHJPAR0049250|Costa Rica|658[0n]|BOLD:ADJ6568
Alphomelon Deans03|[1134]|DHJPAR0049244|Costa Rica|658[0n]|BOLD:ADJ6568
Alphomelon Deans03|[1135]|DHJPAR0058244|Costa Rica|658[0n]|BOLD:ADJ6568
Alphomelon Deans03|[1136]|DHJPAR0058238|Costa Rica|658[0n]|BOLD:ADJ6568
Alphomelon Deans03|[1137]|DHJPAR0048182|Costa Rica|658[0n]|BOLD:ADJ6568
Alphomelon Deans03|[1138]|DHJPAR0012877|Costa Rica|657[0n]|BOLD:ADJ6568
Alphomelon Deans03|[1139]|DHJPAR0058262|Costa Rica|658[0n]|BOLD:ADJ6568
Alphomelon Deans03|[1140]|DHJPAR0058252|Costa Rica|658[0n]|BOLD:ADJ6568
Alphomelon Deans03|[1141]|DHJPAR0031673|Costa Rica|658[0n]|BOLD:ADJ6568
Alphomelon Deans03|[1142]|DHJPAR0031677|Costa Rica|658[0n]|BOLD:ADJ6568
Alphomelon Deans03|[1143]|DHJPAR0031675|Costa Rica|658[0n]|BOLD:ADJ6568
Alphomelon Deans03|[1144]|DHJPAR0031615|Costa Rica|658[0n]|BOLD:ADJ6568
Alphomelon Deans03|[1145]|DHJPAR0031609|Costa Rica|658[0n]|BOLD:ADJ6568
Alphomelon Deans03|[1146]|DHJPAR0030810|Costa Rica|657[0n]|BOLD:ADJ6568
Alphomelon Deans03|[1147]|DHJPAR0005027|Costa Rica|657[0n]|BOLD:ADJ6568
Alphomelon Deans03|[1148]|DHJPAR0048880|Costa Rica|658[0n]|BOLD:ADJ6568
Alphomelon Deans03|[1149]|DHJPAR0047217|Costa Rica|658[0n]|BOLD:ADJ6568
Alphomelon Deans03|[1150]|DHJPAR0031661|Costa Rica|629[0n]|BOLD:ADJ6568
Alphomelon Deans03|[1151]|DHJPAR0043085|Costa Rica|658[0n]|BOLD:ADJ6568
Alphomelon Deans03|[1152]|DHJPAR0031647|Costa Rica|658[0n]|BOLD:ADJ6568
Alphomelon Deans03|[1153]|BIOUG29282-E11|Costa Rica|585[0n]|BOLD:ADJ6568
Alphomelon Deans03|[1154]|BIOUG05082-G11|Costa Rica|633[0n]|BOLD:ADJ6568
Alphomelon Deans03|[1155]|BIOUG64900-A06|Costa Rica|640[0n]|BOLD:ADJ6568
Alphomelon Deans03|[1156]|DHJPAR0031680|Costa Rica|658[0n]|BOLD:ADJ6568
Alphomelon Deans03|[1157]|DHJPAR0031676|Costa Rica|658[0n]|BOLD:ADJ6568
Alphomelon Deans03|[1158]|DHJPAR0031670|Costa Rica|658[0n]|BOLD:ADJ6568
Alphomelon Deans03|[1159]|DHJPAR0031612|Costa Rica|658[0n]|BOLD:ADJ6568
Alphomelon Deans03|[1160]|DHJPAR0049243|Costa Rica|658[0n]|BOLD:ADJ6568

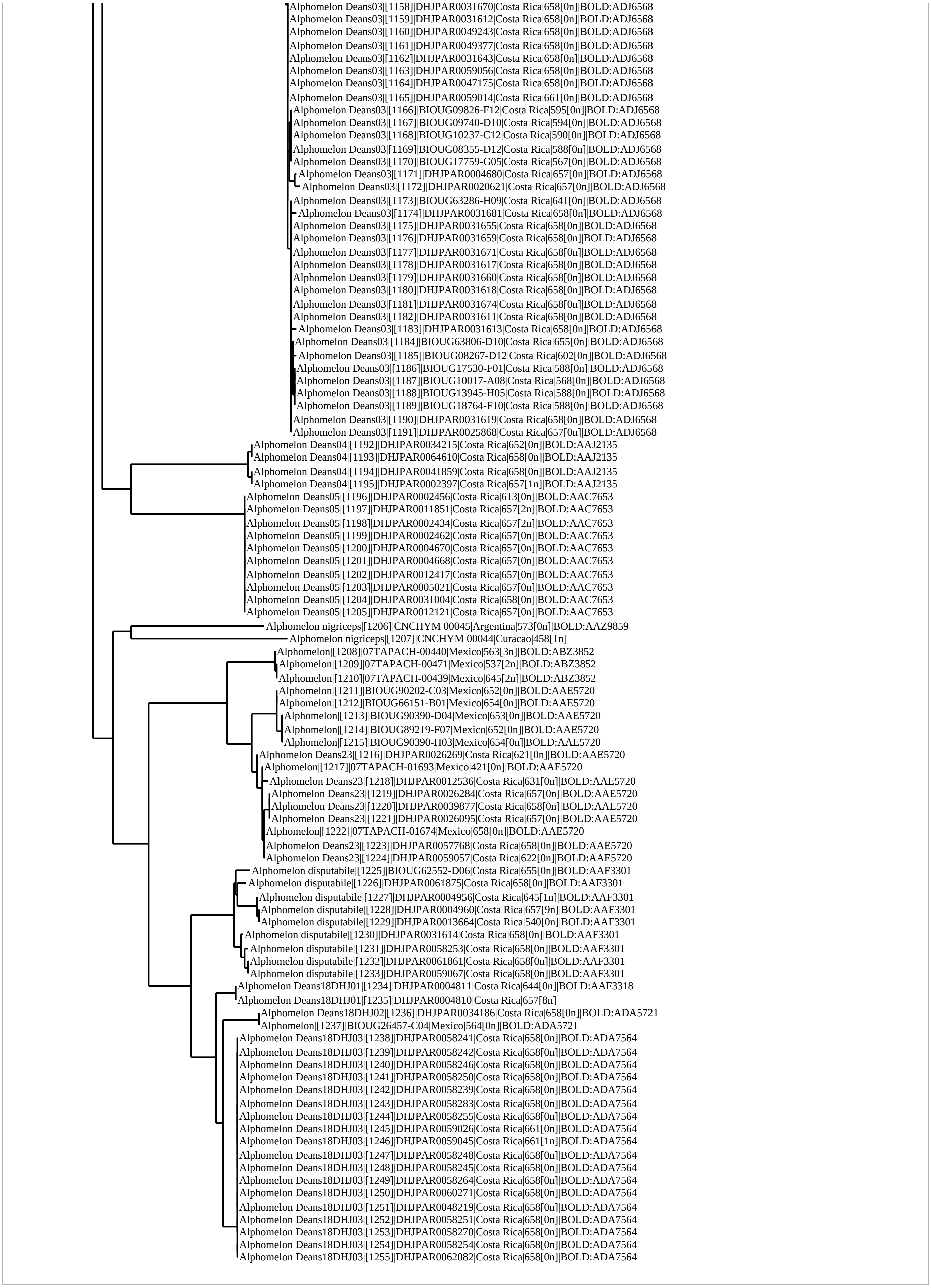
Alphomelon Deans03|[1158]|DHJPAR0031670|Costa Rica|658[0n]|BOLD:ADJ6568
Alphomelon Deans03|[1159]|DHJPAR0031612|Costa Rica|658[0n]|BOLD:ADJ6568
Alphomelon Deans03|[1160]|DHJPAR0049243|Costa Rica|658[0n]|BOLD:ADJ6568
Alphomelon Deans03|[1161]|DHJPAR0049377|Costa Rica|658[0n]|BOLD:ADJ6568
Alphomelon Deans03|[1162]|DHJPAR0031643|Costa Rica|658[0n]|BOLD:ADJ6568
Alphomelon Deans03|[1163]|DHJPAR0059056|Costa Rica|658[0n]|BOLD:ADJ6568
Alphomelon Deans03|[1164]|DHJPAR0047175|Costa Rica|658[0n]|BOLD:ADJ6568
Alphomelon Deans03|[1165]|DHJPAR0059014|Costa Rica|661[0n]|BOLD:ADJ6568
Alphomelon Deans03|[1166]|BIOUG09826-F12|Costa Rica|595[0n]|BOLD:ADJ6568
Alphomelon Deans03|[1167]|BIOUG09740-D10|Costa Rica|594[0n]|BOLD:ADJ6568
Alphomelon Deans03|[1168]|BIOUG10237-C12|Costa Rica|590[0n]|BOLD:ADJ6568
Alphomelon Deans03|[1169]|BIOUG08355-D12|Costa Rica|588[0n]|BOLD:ADJ6568
Alphomelon Deans03|[1170]|BIOUG17759-G05|Costa Rica|567[0n]|BOLD:ADJ6568
Alphomelon Deans03|[1171]|DHJPAR0004680|Costa Rica|657[0n]|BOLD:ADJ6568
Alphomelon Deans03|[1172]|DHJPAR0020621|Costa Rica|657[0n]|BOLD:ADJ6568
Alphomelon Deans03|[1173]|BIOUG63286-H09|Costa Rica|641[0n]|BOLD:ADJ6568
Alphomelon Deans03|[1174]|DHJPAR0031681|Costa Rica|658[0n]|BOLD:ADJ6568
Alphomelon Deans03|[1175]|DHJPAR0031655|Costa Rica|658[0n]|BOLD:ADJ6568
Alphomelon Deans03|[1176]|DHJPAR0031659|Costa Rica|658[0n]|BOLD:ADJ6568
Alphomelon Deans03|[1177]|DHJPAR0031671|Costa Rica|658[0n]|BOLD:ADJ6568
Alphomelon Deans03|[1178]|DHJPAR0031617|Costa Rica|658[0n]|BOLD:ADJ6568
Alphomelon Deans03|[1179]|DHJPAR0031660|Costa Rica|658[0n]|BOLD:ADJ6568
Alphomelon Deans03|[1180]|DHJPAR0031618|Costa Rica|658[0n]|BOLD:ADJ6568
Alphomelon Deans03|[1181]|DHJPAR0031674|Costa Rica|658[0n]|BOLD:ADJ6568
Alphomelon Deans03|[1182]|DHJPAR0031611|Costa Rica|658[0n]|BOLD:ADJ6568
Alphomelon Deans03|[1183]|DHJPAR0031613|Costa Rica|658[0n]|BOLD:ADJ6568
Alphomelon Deans03|[1184]|BIOUG63806-D10|Costa Rica|655[0n]|BOLD:ADJ6568
Alphomelon Deans03|[1185]|BIOUG08267-D12|Costa Rica|602[0n]|BOLD:ADJ6568
Alphomelon Deans03|[1186]|BIOUG17530-F01|Costa Rica|588[0n]|BOLD:ADJ6568
Alphomelon Deans03|[1187]|BIOUG10017-A08|Costa Rica|568[0n]|BOLD:ADJ6568
Alphomelon Deans03|[1188]|BIOUG13945-H05|Costa Rica|588[0n]|BOLD:ADJ6568
Alphomelon Deans03|[1189]|BIOUG18764-F10|Costa Rica|588[0n]|BOLD:ADJ6568
Alphomelon Deans03|[1190]|DHJPAR0031619|Costa Rica|658[0n]|BOLD:ADJ6568
Alphomelon Deans03|[1191]|DHJPAR0025868|Costa Rica|657[0n]|BOLD:ADJ6568
Alphomelon Deans04|[1192]|DHJPAR0034215|Costa Rica|652[0n]|BOLD:AAJ2135
Alphomelon Deans04|[1193]|DHJPAR0064610|Costa Rica|658[0n]|BOLD:AAJ2135
Alphomelon Deans04|[1194]|DHJPAR0041859|Costa Rica|658[0n]|BOLD:AAJ2135
Alphomelon Deans04|[1195]|DHJPAR0002397|Costa Rica|657[1n]|BOLD:AAJ2135
Alphomelon Deans05|[1196]|DHJPAR0002456|Costa Rica|613[0n]|BOLD:AAC7653
Alphomelon Deans05|[1197]|DHJPAR0011851|Costa Rica|657[2n]|BOLD:AAC7653
Alphomelon Deans05|[1198]|DHJPAR0002434|Costa Rica|657[2n]|BOLD:AAC7653
Alphomelon Deans05|[1199]|DHJPAR0002462|Costa Rica|657[0n]|BOLD:AAC7653
Alphomelon Deans05|[1200]|DHJPAR0004670|Costa Rica|657[0n]|BOLD:AAC7653
Alphomelon Deans05|[1201]|DHJPAR0004668|Costa Rica|657[0n]|BOLD:AAC7653
Alphomelon Deans05|[1202]|DHJPAR0012417|Costa Rica|657[0n]|BOLD:AAC7653
Alphomelon Deans05|[1203]|DHJPAR0005021|Costa Rica|657[0n]|BOLD:AAC7653
Alphomelon Deans05|[1204]|DHJPAR0031004|Costa Rica|658[0n]|BOLD:AAC7653
Alphomelon Deans05|[1205]|DHJPAR0012121|Costa Rica|657[0n]|BOLD:AAC7653
Alphomelon nigriceps|[1206]|CNCHYM 00045|Argentina|573[0n]|BOLD:AAZ9859
Alphomelon nigriceps|[1207]|CNCHYM 00044|Curacao|458[1n]
Alphomelon|[1208]|07TAPACH-00440|Mexico|563[3n]|BOLD:ABZ3852
Alphomelon|[1209]|07TAPACH-00471|Mexico|537[2n]|BOLD:ABZ3852
Alphomelon|[1210]|07TAPACH-00439|Mexico|645[2n]|BOLD:ABZ3852
Alphomelon|[1211]|BIOUG90202-C03|Mexico|652[0n]|BOLD:AAE5720
Alphomelon|[1212]|BIOUG66151-B01|Mexico|654[0n]|BOLD:AAE5720
Alphomelon|[1213]|BIOUG90390-D04|Mexico|653[0n]|BOLD:AAE5720
Alphomelon|[1214]|BIOUG89219-F07|Mexico|652[0n]|BOLD:AAE5720
Alphomelon|[1215]|BIOUG90390-H03|Mexico|654[0n]|BOLD:AAE5720
Alphomelon Deans23|[1216]|DHJPAR0026269|Costa Rica|621[0n]|BOLD:AAE5720
Alphomelon|[1217]|07TAPACH-01693|Mexico|421[0n]|BOLD:AAE5720
Alphomelon Deans23|[1218]|DHJPAR0012536|Costa Rica|631[0n]|BOLD:AAE5720
Alphomelon Deans23|[1219]|DHJPAR0026284|Costa Rica|657[0n]|BOLD:AAE5720
Alphomelon Deans23|[1220]|DHJPAR0039877|Costa Rica|658[0n]|BOLD:AAE5720
Alphomelon Deans23|[1221]|DHJPAR0026095|Costa Rica|657[0n]|BOLD:AAE5720
Alphomelon|[1222]|07TAPACH-01674|Mexico|658[0n]|BOLD:AAE5720
Alphomelon Deans23|[1223]|DHJPAR0057768|Costa Rica|658[0n]|BOLD:AAE5720
Alphomelon Deans23|[1224]|DHJPAR0059057|Costa Rica|622[0n]|BOLD:AAE5720
Alphomelon disputabile|[1225]|BIOUG62552-D06|Costa Rica|655[0n]|BOLD:AAF3301
Alphomelon disputabile|[1226]|DHJPAR0061875|Costa Rica|658[0n]|BOLD:AAF3301
Alphomelon disputabile|[1227]|DHJPAR0004956|Costa Rica|645[1n]|BOLD:AAF3301
Alphomelon disputabile|[1228]|DHJPAR0004960|Costa Rica|657[9n]|BOLD:AAF3301
Alphomelon disputabile|[1229]|DHJPAR0013664|Costa Rica|540[0n]|BOLD:AAF3301
Alphomelon disputabile|[1230]|DHJPAR0031614|Costa Rica|658[0n]|BOLD:AAF3301
Alphomelon disputabile|[1231]|DHJPAR0058253|Costa Rica|658[0n]|BOLD:AAF3301
Alphomelon disputabile|[1232]|DHJPAR0061861|Costa Rica|658[0n]|BOLD:AAF3301
Alphomelon disputabile|[1233]|DHJPAR0059067|Costa Rica|658[0n]|BOLD:AAF3301
Alphomelon Deans18DHJ01|[1234]|DHJPAR0004811|Costa Rica|644[0n]|BOLD:AAF3318
Alphomelon Deans18DHJ01|[1235]|DHJPAR0004810|Costa Rica|657[8n]
Alphomelon Deans18DHJ02|[1236]|DHJPAR0034186|Costa Rica|658[0n]|BOLD:ADA5721
Alphomelon|[1237]|BIOUG26457-C04|Mexico|564[0n]|BOLD:ADA5721
Alphomelon Deans18DHJ03|[1238]|DHJPAR0058241|Costa Rica|658[0n]|BOLD:ADA7564
Alphomelon Deans18DHJ03|[1239]|DHJPAR0058242|Costa Rica|658[0n]|BOLD:ADA7564
Alphomelon Deans18DHJ03|[1240]|DHJPAR0058246|Costa Rica|658[0n]|BOLD:ADA7564
Alphomelon Deans18DHJ03|[1241]|DHJPAR0058250|Costa Rica|658[0n]|BOLD:ADA7564
Alphomelon Deans18DHJ03|[1242]|DHJPAR0058239|Costa Rica|658[0n]|BOLD:ADA7564
Alphomelon Deans18DHJ03|[1243]|DHJPAR0058283|Costa Rica|658[0n]|BOLD:ADA7564
Alphomelon Deans18DHJ03|[1244]|DHJPAR0058255|Costa Rica|658[0n]|BOLD:ADA7564
Alphomelon Deans18DHJ03|[1245]|DHJPAR0059026|Costa Rica|661[0n]|BOLD:ADA7564
Alphomelon Deans18DHJ03|[1246]|DHJPAR0059045|Costa Rica|661[1n]|BOLD:ADA7564
Alphomelon Deans18DHJ03|[1247]|DHJPAR0058248|Costa Rica|658[0n]|BOLD:ADA7564
Alphomelon Deans18DHJ03|[1248]|DHJPAR0058245|Costa Rica|658[0n]|BOLD:ADA7564
Alphomelon Deans18DHJ03|[1249]|DHJPAR0058264|Costa Rica|658[0n]|BOLD:ADA7564
Alphomelon Deans18DHJ03|[1250]|DHJPAR0060271|Costa Rica|658[0n]|BOLD:ADA7564
Alphomelon Deans18DHJ03|[1251]|DHJPAR0048219|Costa Rica|658[0n]|BOLD:ADA7564
Alphomelon Deans18DHJ03|[1252]|DHJPAR0058251|Costa Rica|658[0n]|BOLD:ADA7564
Alphomelon Deans18DHJ03|[1253]|DHJPAR0058270|Costa Rica|658[0n]|BOLD:ADA7564
Alphomelon Deans18DHJ03|[1254]|DHJPAR0058254|Costa Rica|658[0n]|BOLD:ADA7564
Alphomelon Deans18DHJ03|[1255]|DHJPAR0062082|Costa Rica|658[0n]|BOLD:ADA7564

BOLD TaxonID Tree

Title : Tree Result - DS-ALPHOMEL (1295 records selected)
Date : 14-Apr-2023
Data Type : Nucleotide
Distance Model : Kimura 2 Parameter
Marker : COI-5P
Colourization : [blue]=Stop Codons [red]=Contamination or misidentification

Label : Sample ID
Label : Taxon
Label : Country
Label : Barcode Cluster (BIN)

Filter : length > 500bp only
Filter : exclude records flagged as misidentifications
Filter : exclude records with stop codons
Filter : exclude contaminants

Sequence Count : 1241
Species count : 35
Genus count : 1
Family count : 1
Unidentified : 15

BIN Count : 37

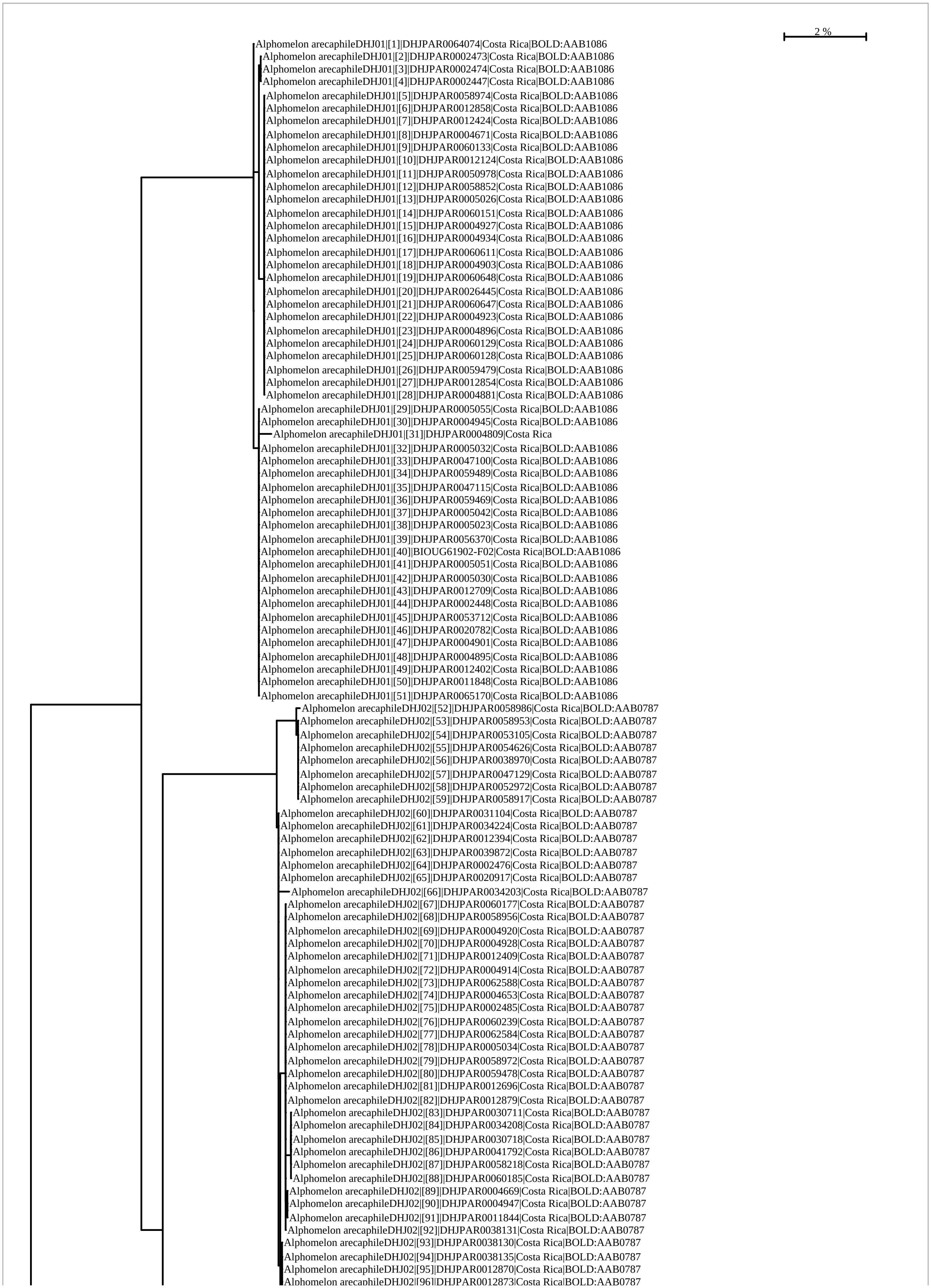
2 %
Alphomelon arecaphileDHJ01|[1]|DHJPAR0064074|Costa Rica|BOLD:AAB1086
Alphomelon arecaphileDHJ01|[2]|DHJPAR0002473|Costa Rica|BOLD:AAB1086
Alphomelon arecaphileDHJ01|[3]|DHJPAR0002474|Costa Rica|BOLD:AAB1086
Alphomelon arecaphileDHJ01|[4]|DHJPAR0002447|Costa Rica|BOLD:AAB1086
Alphomelon arecaphileDHJ01|[5]|DHJPAR0058974|Costa Rica|BOLD:AAB1086
Alphomelon arecaphileDHJ01|[6]|DHJPAR0012858|Costa Rica|BOLD:AAB1086
Alphomelon arecaphileDHJ01|[7]|DHJPAR0012424|Costa Rica|BOLD:AAB1086
Alphomelon arecaphileDHJ01|[8]|DHJPAR0004671|Costa Rica|BOLD:AAB1086
Alphomelon arecaphileDHJ01|[9]|DHJPAR0060133|Costa Rica|BOLD:AAB1086
Alphomelon arecaphileDHJ01|[10]|DHJPAR0012124|Costa Rica|BOLD:AAB1086
Alphomelon arecaphileDHJ01|[11]|DHJPAR0050978|Costa Rica|BOLD:AAB1086
Alphomelon arecaphileDHJ01|[12]|DHJPAR0058852|Costa Rica|BOLD:AAB1086
Alphomelon arecaphileDHJ01|[13]|DHJPAR0005026|Costa Rica|BOLD:AAB1086
Alphomelon arecaphileDHJ01|[14]|DHJPAR0060151|Costa Rica|BOLD:AAB1086
Alphomelon arecaphileDHJ01|[15]|DHJPAR0004927|Costa Rica|BOLD:AAB1086
Alphomelon arecaphileDHJ01|[16]|DHJPAR0004934|Costa Rica|BOLD:AAB1086
Alphomelon arecaphileDHJ01|[17]|DHJPAR0060611|Costa Rica|BOLD:AAB1086
Alphomelon arecaphileDHJ01|[18]|DHJPAR0004903|Costa Rica|BOLD:AAB1086
Alphomelon arecaphileDHJ01|[19]|DHJPAR0060648|Costa Rica|BOLD:AAB1086
Alphomelon arecaphileDHJ01|[20]|DHJPAR0026445|Costa Rica|BOLD:AAB1086
Alphomelon arecaphileDHJ01|[21]|DHJPAR0060647|Costa Rica|BOLD:AAB1086
Alphomelon arecaphileDHJ01|[22]|DHJPAR0004923|Costa Rica|BOLD:AAB1086
Alphomelon arecaphileDHJ01|[23]|DHJPAR0004896|Costa Rica|BOLD:AAB1086
Alphomelon arecaphileDHJ01|[24]|DHJPAR0060129|Costa Rica|BOLD:AAB1086
Alphomelon arecaphileDHJ01|[25]|DHJPAR0060128|Costa Rica|BOLD:AAB1086
Alphomelon arecaphileDHJ01|[26]|DHJPAR0059479|Costa Rica|BOLD:AAB1086
Alphomelon arecaphileDHJ01|[27]|DHJPAR0012854|Costa Rica|BOLD:AAB1086
Alphomelon arecaphileDHJ01|[28]|DHJPAR0004881|Costa Rica|BOLD:AAB1086
Alphomelon arecaphileDHJ01|[29]|DHJPAR0005055|Costa Rica|BOLD:AAB1086
Alphomelon arecaphileDHJ01|[30]|DHJPAR0004945|Costa Rica|BOLD:AAB1086
Alphomelon arecaphileDHJ01|[31]|DHJPAR0004809|Costa Rica
Alphomelon arecaphileDHJ01|[32]|DHJPAR0005032|Costa Rica|BOLD:AAB1086
Alphomelon arecaphileDHJ01|[33]|DHJPAR0047100|Costa Rica|BOLD:AAB1086
Alphomelon arecaphileDHJ01|[34]|DHJPAR0059489|Costa Rica|BOLD:AAB1086
Alphomelon arecaphileDHJ01|[35]|DHJPAR0047115|Costa Rica|BOLD:AAB1086
Alphomelon arecaphileDHJ01|[36]|DHJPAR0059469|Costa Rica|BOLD:AAB1086
Alphomelon arecaphileDHJ01|[37]|DHJPAR0005042|Costa Rica|BOLD:AAB1086
Alphomelon arecaphileDHJ01|[38]|DHJPAR0005023|Costa Rica|BOLD:AAB1086
Alphomelon arecaphileDHJ01|[39]|DHJPAR0056370|Costa Rica|BOLD:AAB1086
Alphomelon arecaphileDHJ01|[40]|BIOUG61902-F02|Costa Rica|BOLD:AAB1086
Alphomelon arecaphileDHJ01|[41]|DHJPAR0005051|Costa Rica|BOLD:AAB1086
Alphomelon arecaphileDHJ01|[42]|DHJPAR0005030|Costa Rica|BOLD:AAB1086
Alphomelon arecaphileDHJ01|[43]|DHJPAR0012709|Costa Rica|BOLD:AAB1086
Alphomelon arecaphileDHJ01|[44]|DHJPAR0002448|Costa Rica|BOLD:AAB1086
Alphomelon arecaphileDHJ01|[45]|DHJPAR0053712|Costa Rica|BOLD:AAB1086
Alphomelon arecaphileDHJ01|[46]|DHJPAR0020782|Costa Rica|BOLD:AAB1086
Alphomelon arecaphileDHJ01|[47]|DHJPAR0004901|Costa Rica|BOLD:AAB1086
Alphomelon arecaphileDHJ01|[48]|DHJPAR0004895|Costa Rica|BOLD:AAB1086
Alphomelon arecaphileDHJ01|[49]|DHJPAR0012402|Costa Rica|BOLD:AAB1086
Alphomelon arecaphileDHJ01|[50]|DHJPAR0011848|Costa Rica|BOLD:AAB1086
Alphomelon arecaphileDHJ01|[51]|DHJPAR0065170|Costa Rica|BOLD:AAB1086
Alphomelon arecaphileDHJ02|[52]|DHJPAR0058986|Costa Rica|BOLD:AAB0787
Alphomelon arecaphileDHJ02|[53]|DHJPAR0058953|Costa Rica|BOLD:AAB0787
Alphomelon arecaphileDHJ02|[54]|DHJPAR0053105|Costa Rica|BOLD:AAB0787
Alphomelon arecaphileDHJ02|[55]|DHJPAR0054626|Costa Rica|BOLD:AAB0787
Alphomelon arecaphileDHJ02|[56]|DHJPAR0038970|Costa Rica|BOLD:AAB0787
Alphomelon arecaphileDHJ02|[57]|DHJPAR0047129|Costa Rica|BOLD:AAB0787
Alphomelon arecaphileDHJ02|[58]|DHJPAR0052972|Costa Rica|BOLD:AAB0787
Alphomelon arecaphileDHJ02|[59]|DHJPAR0058917|Costa Rica|BOLD:AAB0787
Alphomelon arecaphileDHJ02|[60]|DHJPAR0031104|Costa Rica|BOLD:AAB0787
Alphomelon arecaphileDHJ02|[61]|DHJPAR0034224|Costa Rica|BOLD:AAB0787
Alphomelon arecaphileDHJ02|[62]|DHJPAR0012394|Costa Rica|BOLD:AAB0787
Alphomelon arecaphileDHJ02|[63]|DHJPAR0039872|Costa Rica|BOLD:AAB0787
Alphomelon arecaphileDHJ02|[64]|DHJPAR0002476|Costa Rica|BOLD:AAB0787
Alphomelon arecaphileDHJ02|[65]|DHJPAR0020917|Costa Rica|BOLD:AAB0787
Alphomelon arecaphileDHJ02|[66]|DHJPAR0034203|Costa Rica|BOLD:AAB0787
Alphomelon arecaphileDHJ02|[67]|DHJPAR0060177|Costa Rica|BOLD:AAB0787
Alphomelon arecaphileDHJ02|[68]|DHJPAR0058956|Costa Rica|BOLD:AAB0787
Alphomelon arecaphileDHJ02|[69]|DHJPAR0004920|Costa Rica|BOLD:AAB0787
Alphomelon arecaphileDHJ02|[70]|DHJPAR0004928|Costa Rica|BOLD:AAB0787
Alphomelon arecaphileDHJ02|[71]|DHJPAR0012409|Costa Rica|BOLD:AAB0787
Alphomelon arecaphileDHJ02|[72]|DHJPAR0004914|Costa Rica|BOLD:AAB0787
Alphomelon arecaphileDHJ02|[73]|DHJPAR0062588|Costa Rica|BOLD:AAB0787
Alphomelon arecaphileDHJ02|[74]|DHJPAR0004653|Costa Rica|BOLD:AAB0787
Alphomelon arecaphileDHJ02|[75]|DHJPAR0002485|Costa Rica|BOLD:AAB0787
Alphomelon arecaphileDHJ02|[76]|DHJPAR0060239|Costa Rica|BOLD:AAB0787
Alphomelon arecaphileDHJ02|[77]|DHJPAR0062584|Costa Rica|BOLD:AAB0787
Alphomelon arecaphileDHJ02|[78]|DHJPAR0005034|Costa Rica|BOLD:AAB0787
Alphomelon arecaphileDHJ02|[79]|DHJPAR0058972|Costa Rica|BOLD:AAB0787
Alphomelon arecaphileDHJ02|[80]|DHJPAR0059478|Costa Rica|BOLD:AAB0787
Alphomelon arecaphileDHJ02|[81]|DHJPAR0012696|Costa Rica|BOLD:AAB0787
Alphomelon arecaphileDHJ02|[82]|DHJPAR0012879|Costa Rica|BOLD:AAB0787
Alphomelon arecaphileDHJ02|[83]|DHJPAR0030711|Costa Rica|BOLD:AAB0787
Alphomelon arecaphileDHJ02|[84]|DHJPAR0034208|Costa Rica|BOLD:AAB0787
Alphomelon arecaphileDHJ02|[85]|DHJPAR0030718|Costa Rica|BOLD:AAB0787
Alphomelon arecaphileDHJ02|[86]|DHJPAR0041792|Costa Rica|BOLD:AAB0787
Alphomelon arecaphileDHJ02|[87]|DHJPAR0058218|Costa Rica|BOLD:AAB0787
Alphomelon arecaphileDHJ02|[88]|DHJPAR0060185|Costa Rica|BOLD:AAB0787
Alphomelon arecaphileDHJ02|[89]|DHJPAR0004669|Costa Rica|BOLD:AAB0787
Alphomelon arecaphileDHJ02|[90]|DHJPAR0004947|Costa Rica|BOLD:AAB0787
Alphomelon arecaphileDHJ02|[91]|DHJPAR0011844|Costa Rica|BOLD:AAB0787
Alphomelon arecaphileDHJ02|[92]|DHJPAR0038131|Costa Rica|BOLD:AAB0787
Alphomelon arecaphileDHJ02|[93]|DHJPAR0038130|Costa Rica|BOLD:AAB0787
Alphomelon arecaphileDHJ02|[94]|DHJPAR0038135|Costa Rica|BOLD:AAB0787
Alphomelon arecaphileDHJ02|[95]|DHJPAR0012870|Costa Rica|BOLD:AAB0787
Alphomelon arecaphileDHJ02|[96]|DHJPAR0012873|Costa Rica|BOLD:AAB0787

Alphomelon arecaphileDHJ02|[94]|DHJPAR0038135|Costa Rica|BOLD:AAB0787
Alphomelon arecaphileDHJ02|[95]|DHJPAR0012870|Costa Rica|BOLD:AAB0787
Alphomelon arecaphileDHJ02|[96]|DHJPAR0012873|Costa Rica|BOLD:AAB0787
Alphomelon arecaphileDHJ02|[97]|DHJPAR0057779|Costa Rica|BOLD:AAB0787
Alphomelon arecaphileDHJ02|[98]|DHJPAR0058840|Costa Rica|BOLD:AAB0787
Alphomelon arecaphileDHJ02|[99]|DHJPAR0058843|Costa Rica|BOLD:AAB0787
Alphomelon arecaphileDHJ02|[100]|DHJPAR0058939|Costa Rica|BOLD:AAB0787
Alphomelon arecaphileDHJ02|[101]|DHJPAR0058942|Costa Rica|BOLD:AAB0787
Alphomelon arecaphileDHJ02|[102]|DHJPAR0058948|Costa Rica|BOLD:AAB0787
Alphomelon arecaphileDHJ02|[103]|DHJPAR0060234|Costa Rica|BOLD:AAB0787
Alphomelon arecaphileDHJ02|[104]|DHJPAR0058981|Costa Rica|BOLD:AAB0787
Alphomelon arecaphileDHJ02|[105]|DHJPAR0058982|Costa Rica|BOLD:AAB0787
Alphomelon arecaphileDHJ02|[106]|DHJPAR0051778|Costa Rica|BOLD:AAB0787
Alphomelon arecaphileDHJ02|[107]|DHJPAR0004907|Costa Rica|BOLD:AAB0787
Alphomelon arecaphileDHJ02|[108]|DHJPAR0058957|Costa Rica|BOLD:AAB0787
Alphomelon arecaphileDHJ02|[109]|DHJPAR0058195|Costa Rica|BOLD:AAB0787
Alphomelon arecaphileDHJ02|[110]|DHJPAR0005020|Costa Rica|BOLD:AAB0787
Alphomelon arecaphileDHJ02|[111]|DHJPAR0039878|Costa Rica|BOLD:AAB0787
Alphomelon arecaphileDHJ02|[112]|DHJPAR0041775|Costa Rica|BOLD:AAB0787
Alphomelon arecaphileDHJ02|[113]|DHJPAR0041794|Costa Rica|BOLD:AAB0787
Alphomelon arecaphileDHJ02|[114]|DHJPAR0004926|Costa Rica|BOLD:AAB0787
Alphomelon arecaphileDHJ02|[115]|DHJPAR0058895|Costa Rica|BOLD:AAB0787
Alphomelon arecaphileDHJ02|[116]|DHJPAR0051032|Costa Rica|BOLD:AAB0787
Alphomelon arecaphileDHJ02|[117]|DHJPAR0051038|Costa Rica|BOLD:AAB0787
Alphomelon arecaphileDHJ02|[118]|DHJPAR0012705|Costa Rica|BOLD:AAB0787
Alphomelon arecaphileDHJ02|[119]|DHJPAR0020200|Costa Rica|BOLD:AAB0787
Alphomelon arecaphileDHJ02|[120]|DHJPAR0005066|Costa Rica|BOLD:AAB0787
Alphomelon arecaphileDHJ02|[121]|DHJPAR0005018|Costa Rica|BOLD:AAB0787
Alphomelon arecaphileDHJ02|[122]|DHJPAR0004916|Costa Rica|BOLD:AAB0787
Alphomelon arecaphileDHJ02|[123]|DHJPAR0004911|Costa Rica|BOLD:AAB0787
Alphomelon arecaphileDHJ02|[124]|DHJPAR0004883|Costa Rica|BOLD:AAB0787
Alphomelon arecaphileDHJ02|[125]|DHJPAR0055257|Costa Rica|BOLD:AAB0787
Alphomelon melanoscelis|[126]|DHJPAR0051219|Costa Rica|BOLD:AAB6733
Alphomelon melanoscelis|[127]|DHJPAR0004904|Costa Rica|BOLD:AAB6733
Alphomelon melanoscelis|[128]|DHJPAR0057763|Costa Rica|BOLD:AAB6733
Alphomelon melanoscelis|[129]|DHJPAR0004890|Costa Rica|BOLD:AAB6733
Alphomelon melanoscelis|[130]|DHJPAR0060618|Costa Rica|BOLD:AAB6733
Alphomelon melanoscelis|[131]|DHJPAR0034243|Costa Rica|BOLD:AAB6733
Alphomelon melanoscelis|[132]|DHJPAR0055274|Costa Rica|BOLD:AAB6733
Alphomelon melanoscelis|[133]|DHJPAR0002395|Costa Rica|BOLD:AAB6733
Alphomelon melanoscelis|[134]|DHJPAR0058834|Costa Rica|BOLD:AAB6733
Alphomelon melanoscelis|[135]|DHJPAR0058841|Costa Rica|BOLD:AAB6733
Alphomelon melanoscelis|[136]|DHJPAR0058842|Costa Rica|BOLD:AAB6733
Alphomelon melanoscelis|[137]|DHJPAR0058844|Costa Rica|BOLD:AAB6733
Alphomelon melanoscelis|[138]|DHJPAR0058848|Costa Rica|BOLD:AAB6733
Alphomelon melanoscelis|[139]|DHJPAR0058866|Costa Rica|BOLD:AAB6733
Alphomelon melanoscelis|[140]|DHJPAR0058888|Costa Rica|BOLD:AAB6733
Alphomelon melanoscelis|[141]|DHJPAR0060625|Costa Rica|BOLD:AAB6733
Alphomelon melanoscelis|[142]|DHJPAR0058941|Costa Rica|BOLD:AAB6733
Alphomelon melanoscelis|[143]|DHJPAR0058946|Costa Rica|BOLD:AAB6733
Alphomelon melanoscelis|[144]|DHJPAR0058954|Costa Rica|BOLD:AAB6733
Alphomelon melanoscelis|[145]|DHJPAR0058964|Costa Rica|BOLD:AAB6733
Alphomelon melanoscelis|[146]|DHJPAR0058980|Costa Rica|BOLD:AAB6733
Alphomelon melanoscelis|[147]|DHJPAR0058983|Costa Rica|BOLD:AAB6733
Alphomelon melanoscelis|[148]|DHJPAR0004899|Costa Rica|BOLD:AAB6733
Alphomelon melanoscelis|[149]|DHJPAR0004908|Costa Rica|BOLD:AAB6733
Alphomelon melanoscelis|[150]|DHJPAR0004913|Costa Rica|BOLD:AAB6733
Alphomelon melanoscelis|[151]|DHJPAR0004918|Costa Rica|BOLD:AAB6733
Alphomelon melanoscelis|[152]|DHJPAR0012412|Costa Rica|BOLD:AAB6733
Alphomelon melanoscelis|[153]|DHJPAR0012419|Costa Rica|BOLD:AAB6733
Alphomelon melanoscelis|[154]|DHJPAR0012425|Costa Rica|BOLD:AAB6733
Alphomelon melanoscelis|[155]|DHJPAR0060630|Costa Rica|BOLD:AAB6733
Alphomelon melanoscelis|[156]|DHJPAR0020291|Costa Rica|BOLD:AAB6733
Alphomelon melanoscelis|[157]|DHJPAR0061666|Costa Rica|BOLD:AAB6733
Alphomelon melanoscelis|[158]|DHJPAR0058177|Costa Rica|BOLD:AAB6733
Alphomelon melanoscelis|[159]|DHJPAR0058156|Costa Rica|BOLD:AAB6733
Alphomelon melanoscelis|[160]|DHJPAR0060232|Costa Rica|BOLD:AAB6733
Alphomelon melanoscelis|[161]|DHJPAR0057502|Costa Rica|BOLD:AAB6733
Alphomelon melanoscelis|[162]|DHJPAR0041766|Costa Rica|BOLD:AAB6733
Alphomelon melanoscelis|[163]|DHJPAR0057514|Costa Rica|BOLD:AAB6733
Alphomelon melanoscelis|[164]|DHJPAR0041692|Costa Rica|BOLD:AAB6733
Alphomelon melanoscelis|[165]|DHJPAR0058213|Costa Rica|BOLD:AAB6733
Alphomelon melanoscelis|[166]|DHJPAR0041714|Costa Rica|BOLD:AAB6733
Alphomelon melanoscelis|[167]|DHJPAR0041739|Costa Rica|BOLD:AAB6733
Alphomelon melanoscelis|[168]|DHJPAR0058851|Costa Rica|BOLD:AAB6733
Alphomelon melanoscelis|[169]|DHJPAR0041756|Costa Rica|BOLD:AAB6733
Alphomelon melanoscelis|[170]|DHJPAR0058861|Costa Rica|BOLD:AAB6733
Alphomelon melanoscelis|[171]|DHJPAR0061623|Costa Rica|BOLD:AAB6733
Alphomelon melanoscelis|[172]|DHJPAR0058168|Costa Rica|BOLD:AAB6733
Alphomelon melanoscelis|[173]|DHJPAR0058219|Costa Rica|BOLD:AAB6733
Alphomelon melanoscelis|[174]|DHJPAR0060617|Costa Rica|BOLD:AAB6733
Alphomelon melanoscelis|[175]|DHJPAR0059480|Costa Rica|BOLD:AAB6733
Alphomelon melanoscelis|[176]|DHJPAR0058936|Costa Rica|BOLD:AAB6733
Alphomelon melanoscelis|[177]|DHJPAR0061628|Costa Rica|BOLD:AAB6733
Alphomelon melanoscelis|[178]|DHJPAR0055445|Costa Rica|BOLD:AAB6733
Alphomelon melanoscelis|[179]|DHJPAR0057770|Costa Rica|BOLD:AAB6733
Alphomelon melanoscelis|[180]|DHJPAR0012404|Costa Rica|BOLD:AAB6733
Alphomelon melanoscelis|[181]|DHJPAR0020288|Costa Rica|BOLD:AAB6733
Alphomelon melanoscelis|[182]|DHJPAR0012872|Costa Rica|BOLD:AAB6733
Alphomelon melanoscelis|[183]|DHJPAR0004929|Costa Rica|BOLD:AAB6733
Alphomelon melanoscelis|[184]|DHJPAR0034227|Costa Rica|BOLD:AAB6733
Alphomelon melanoscelis|[185]|DHJPAR0005035|Costa Rica|BOLD:AAB6733
Alphomelon melanoscelis|[186]|DHJPAR0034230|Costa Rica|BOLD:AAB6733
Alphomelon xestopygaDHJ05|[187]|DHJPAR0002449|Costa Rica|BOLD:AAA1634
Alphomelon xestopygaDHJ05|[188]|DHJPAR0012390|Costa Rica|BOLD:AAA1634
Alphomelon xestopygaDHJ05|[189]|DHJPAR0002411|Costa Rica|BOLD:AAA1634
Alphomelon xestopygaDHJ05|[190]|DHJPAR0002367|Costa Rica|BOLD:AAA1634
Alphomelon xestopygaDHJ05|[191]|DHJPAR0011926|Costa Rica|BOLD:AAA1634
[illegible]

Alphomelon xestopygaDHJ05|[189]|DHJPAR0002411|Costa Rica|BOLD:AAA1634
Alphomelon xestopygaDHJ05|[190]|DHJPAR0002367|Costa Rica|BOLD:AAA1634
Alphomelon xestopygaDHJ05|[191]|DHJPAR0011926|Costa Rica|BOLD:AAA1634
Alphomelon xestopygaDHJ05|[192]|DHJPAR0020295|Costa Rica|BOLD:AAA1634
Alphomelon xestopygaDHJ05|[193]|DHJPAR0002423|Costa Rica|BOLD:AAA1634
Alphomelon xestopygaDHJ05|[194]|DHJPAR0013158|Costa Rica|BOLD:AAA1634
Alphomelon xestopygaDHJ05|[195]|DHJPAR0002417|Costa Rica|BOLD:AAA1634
Alphomelon xestopygaDHJ05|[196]|DHJPAR0011928|Costa Rica|BOLD:AAA1634
Alphomelon xestopygaDHJ05|[197]|DHJPAR0002425|Costa Rica|BOLD:AAA1634
Alphomelon xestopygaDHJ05|[198]|DHJPAR0002421|Costa Rica|BOLD:AAA1634
Alphomelon xestopygaDHJ05|[199]|DHJPAR0013167|Costa Rica|BOLD:AAA1634
Alphomelon xestopygaDHJ05|[200]|DHJPAR0013163|Costa Rica|BOLD:AAA1634
Alphomelon xestopygaDHJ05|[201]|DHJPAR0058858|Costa Rica|BOLD:AAA1634
Alphomelon xestopygaDHJ05|[202]|DHJPAR0060130|Costa Rica|BOLD:AAA1634
Alphomelon xestopygaDHJ05|[203]|DHJPAR0002433|Costa Rica|BOLD:AAA1634
Alphomelon xestopygaDHJ05|[204]|DHJPAR0002427|Costa Rica|BOLD:AAA1634
Alphomelon xestopygaDHJ05|[205]|DHJPAR0058915|Costa Rica|BOLD:AAA1634
Alphomelon xestopygaDHJ05|[206]|DHJPAR0058879|Costa Rica|BOLD:AAA1634
Alphomelon xestopygaDHJ05|[207]|DHJPAR0013157|Costa Rica|BOLD:AAA1634
Alphomelon xestopygaDHJ05|[208]|DHJPAR0058955|Costa Rica|BOLD:AAA1634
Alphomelon xestopygaDHJ05|[209]|DHJPAR0002496|Costa Rica|BOLD:AAA1634
Alphomelon xestopygaDHJ05|[210]|DHJPAR0020290|Costa Rica|BOLD:AAA1634
Alphomelon xestopygaDHJ05|[211]|DHJPAR0012418|Costa Rica|BOLD:AAA1634
Alphomelon xestopygaDHJ05|[212]|DHJPAR0012407|Costa Rica|BOLD:AAA1634
Alphomelon xestopygaDHJ05|[213]|DHJPAR0004646|Costa Rica|BOLD:AAA1634
Alphomelon xestopygaDHJ05|[214]|DHJPAR0004641|Costa Rica|BOLD:AAA1634
Alphomelon xestopygaDHJ05|[215]|DHJPAR0004659|Costa Rica|BOLD:AAA1634
Alphomelon xestopygaDHJ05|[216]|DHJPAR0004648|Costa Rica|BOLD:AAA1634
Alphomelon xestopygaDHJ05|[217]|DHJPAR0004675|Costa Rica|BOLD:AAA1634
Alphomelon xestopygaDHJ05|[218]|DHJPAR0004672|Costa Rica|BOLD:AAA1634
Alphomelon xestopygaDHJ05|[219]|DHJPAR0004678|Costa Rica|BOLD:AAA1634
Alphomelon xestopygaDHJ05|[220]|DHJPAR0004676|Costa Rica|BOLD:AAA1634
Alphomelon xestopygaDHJ05|[221]|DHJPAR0004681|Costa Rica|BOLD:AAA1634
Alphomelon xestopygaDHJ05|[222]|DHJPAR0004679|Costa Rica|BOLD:AAA1634
Alphomelon xestopygaDHJ05|[223]|DHJPAR0002412|Costa Rica|BOLD:AAA1634
Alphomelon xestopygaDHJ05|[224]|DHJPAR0004683|Costa Rica|BOLD:AAA1634
Alphomelon xestopygaDHJ05|[225]|DHJPAR0061612|Costa Rica|BOLD:AAA1634
Alphomelon xestopygaDHJ05|[226]|DHJPAR0002438|Costa Rica|BOLD:AAA1634
Alphomelon xestopygaDHJ05|[227]|DHJPAR0002444|Costa Rica|BOLD:AAA1634
Alphomelon xestopygaDHJ05|[228]|DHJPAR0002441|Costa Rica|BOLD:AAA1634
Alphomelon xestopygaDHJ05|[229]|DHJPAR0002492|Costa Rica|BOLD:AAA1634
Alphomelon xestopygaDHJ05|[230]|DHJPAR0002451|Costa Rica|BOLD:AAA1634
Alphomelon xestopygaDHJ05|[231]|DHJPAR0002388|Costa Rica|BOLD:AAA1634
Alphomelon xestopygaDHJ05|[232]|DHJPAR0002382|Costa Rica|BOLD:AAA1634
Alphomelon xestopygaDHJ05|[233]|DHJPAR0011927|Costa Rica|BOLD:AAA1634
Alphomelon xestopygaDHJ05|[234]|DHJPAR0058159|Costa Rica|BOLD:AAA1634
Alphomelon xestopygaDHJ05|[235]|DHJPAR0002495|Costa Rica|BOLD:AAA1634
Alphomelon xestopygaDHJ05|[236]|DHJPAR0002493|Costa Rica|BOLD:AAA1634
Alphomelon xestopygaDHJ05|[237]|DHJPAR0058158|Costa Rica|BOLD:AAA1634
Alphomelon xestopygaDHJ05|[238]|DHJPAR0061687|Costa Rica|BOLD:AAA1634
Alphomelon xestopygaDHJ05|[239]|DHJPAR0058176|Costa Rica|BOLD:AAA1634
Alphomelon xestopygaDHJ05|[240]|DHJPAR0012123|Costa Rica|BOLD:AAA1634
Alphomelon xestopygaDHJ05|[241]|DHJPAR0059765|Costa Rica|BOLD:AAA1634
Alphomelon xestopygaDHJ05|[242]|DHJPAR0058183|Costa Rica|BOLD:AAA1634
Alphomelon xestopygaDHJ05|[243]|DHJPAR0002343|Costa Rica|BOLD:AAA1634
Alphomelon xestopygaDHJ05|[244]|DHJPAR0002347|Costa Rica|BOLD:AAA1634
Alphomelon xestopygaDHJ05|[245]|DHJPAR0012126|Costa Rica|BOLD:AAA1634
Alphomelon xestopygaDHJ05|[246]|DHJPAR0002344|Costa Rica|BOLD:AAA1634
Alphomelon xestopygaDHJ05|[247]|DHJPAR0013168|Costa Rica|BOLD:AAA1634
Alphomelon xestopygaDHJ05|[248]|DHJPAR0002349|Costa Rica|BOLD:AAA1634
Alphomelon xestopygaDHJ05|[249]|DHJPAR0059725|Costa Rica|BOLD:AAA1634
Alphomelon xestopygaDHJ05|[250]|DHJPAR0058230|Costa Rica|BOLD:AAA1634
Alphomelon xestopygaDHJ05|[251]|DHJPAR0062607|Costa Rica|BOLD:AAA1634
Alphomelon xestopygaDHJ05|[252]|DHJPAR0002494|Costa Rica|BOLD:AAA1634
Alphomelon xestopygaDHJ05|[253]|DHJPAR0031099|Costa Rica|BOLD:AAA1634
Alphomelon xestopygaDHJ05|[254]|DHJPAR0031094|Costa Rica|BOLD:AAA1634
Alphomelon xestopygaDHJ05|[255]|DHJPAR0011923|Costa Rica|BOLD:AAA1634
Alphomelon xestopygaDHJ05|[256]|DHJPAR0005043|Costa Rica|BOLD:AAA1634
Alphomelon xestopygaDHJ05|[257]|DHJPAR0031107|Costa Rica|BOLD:AAA1634
Alphomelon xestopygaDHJ05|[258]|DHJPAR0056537|Costa Rica|BOLD:AAA1634
Alphomelon xestopygaDHJ05|[259]|DHJPAR0002401|Costa Rica|BOLD:AAA1634
Alphomelon xestopygaDHJ05|[260]|DHJPAR0002400|Costa Rica|BOLD:AAA1634
Alphomelon xestopygaDHJ05|[261]|BIOUG50473-F05|Costa Rica|BOLD:AAA1634
Alphomelon xestopygaDHJ05|[262]|DHJPAR0004813|Costa Rica|BOLD:AAA1634
Alphomelon xestopygaDHJ05|[263]|DHJPAR0031122|Costa Rica|BOLD:AAA1634
Alphomelon xestopygaDHJ05|[264]|DHJPAR0003051|Costa Rica|BOLD:AAA1634
Alphomelon xestopygaDHJ05|[265]|DHJPAR0012392|Costa Rica|BOLD:AAA1634
Alphomelon xestopygaDHJ05|[266]|DHJPAR0012127|Costa Rica|BOLD:AAA1634
Alphomelon xestopygaDHJ05|[267]|DHJPAR0031135|Costa Rica|BOLD:AAA1634
Alphomelon xestopygaDHJ05|[268]|DHJPAR0004906|Costa Rica|BOLD:AAA1634
Alphomelon xestopygaDHJ05|[269]|DHJPAR0020278|Costa Rica|BOLD:AAA1634
Alphomelon xestopygaDHJ05|[270]|DHJPAR0059756|Costa Rica|BOLD:AAA1634
Alphomelon xestopygaDHJ05|[271]|DHJPAR0059744|Costa Rica|BOLD:AAA1634
Alphomelon xestopygaDHJ05|[272]|DHJPAR0002359|Costa Rica|BOLD:AAA1634
Alphomelon xestopygaDHJ05|[273]|DHJPAR0031109|Costa Rica|BOLD:AAA1634
Alphomelon xestopygaDHJ05|[274]|DHJPAR0002379|Costa Rica|BOLD:AAA1634
Alphomelon xestopygaDHJ05|[275]|DHJPAR0002374|Costa Rica|BOLD:AAA1634
Alphomelon xestopygaDHJ05|[276]|DHJPAR0058881|Costa Rica|BOLD:AAA1634
Alphomelon xestopygaDHJ05|[277]|DHJPAR0020286|Costa Rica|BOLD:AAA1634
Alphomelon xestopygaDHJ05|[278]|DHJPAR0013165|Costa Rica|BOLD:AAA1634
Alphomelon xestopygaDHJ05|[279]|DHJPAR0002416|Costa Rica|BOLD:AAA1634
Alphomelon xestopygaDHJ05|[280]|DHJPAR0002351|Costa Rica|BOLD:AAA1634
Alphomelon xestopygaDHJ05|[281]|DHJPAR0020285|Costa Rica|BOLD:AAA1634
Alphomelon xestopygaDHJ05|[282]|DHJPAR0059766|Costa Rica|BOLD:AAA1634
Alphomelon xestopygaDHJ05|[283]|DHJPAR0002368|Costa Rica|BOLD:AAA1634
Alphomelon xestopygaDHJ05|[284]|DHJPAR0002370|Costa Rica|BOLD:AAA1634
Alphomelon xestopygaDHJ05|[285]|DHJPAR0002361|Costa Rica|BOLD:AAA1634
Alphomelon xestopygaDHJ05|[286]|DHJPAR0005036|Costa Rica|BOLD:AAA1634
Alphomelon xestopygaDHJ05|[287]|DHJPAR0005024|Costa Rica|BOLD:AAA1634

Alphomelon xestopygaDHJ05|[285]|DHJPAR0002361|Costa Rica|BOLD:AAA1634
Alphomelon xestopygaDHJ05|[286]|DHJPAR0005036|Costa Rica|BOLD:AAA1634
Alphomelon xestopygaDHJ05|[287]|DHJPAR0005024|Costa Rica|BOLD:AAA1634
Alphomelon xestopygaDHJ05|[288]|DHJPAR0002408|Costa Rica|BOLD:AAA1634
Alphomelon xestopygaDHJ05|[289]|DHJPAR0030897|Costa Rica|BOLD:AAA1634
Alphomelon xestopygaDHJ05|[290]|DHJPAR0031620|Costa Rica|BOLD:AAA1634
Alphomelon xestopygaDHJ05|[291]|DHJPAR0002355|Costa Rica|BOLD:AAA1634
Alphomelon xestopygaDHJ05|[292]|DHJPAR0030922|Costa Rica|BOLD:AAA1634
Alphomelon xestopygaDHJ05|[293]|DHJPAR0031735|Costa Rica|BOLD:AAA1634
Alphomelon xestopygaDHJ05|[294]|DHJPAR0005031|Costa Rica|BOLD:AAA1634
Alphomelon xestopygaDHJ05|[295]|DHJPAR0013155|Costa Rica|BOLD:AAA1634
Alphomelon xestopygaDHJ05|[296]|DHJPAR0002399|Costa Rica|BOLD:AAA1634
Alphomelon xestopygaDHJ05|[297]|DHJPAR0012129|Costa Rica|BOLD:AAA1634
Alphomelon xestopygaDHJ05|[298]|DHJPAR0002489|Costa Rica|BOLD:AAA1634
Alphomelon xestopygaDHJ05|[299]|DHJPAR0002346|Costa Rica|BOLD:AAA1634
Alphomelon xestopygaDHJ05|[300]|DHJPAR0012862|Costa Rica|BOLD:AAA1634
Alphomelon xestopygaDHJ05|[301]|DHJPAR0004964|Costa Rica|BOLD:AAA1634
Alphomelon xestopygaDHJ05|[302]|DHJPAR0004953|Costa Rica|BOLD:AAA1634
Alphomelon xestopygaDHJ05|[303]|DHJPAR0002357|Costa Rica|BOLD:AAA1634
Alphomelon xestopygaDHJ05|[304]|DHJPAR0002354|Costa Rica|BOLD:AAA1634
Alphomelon xestopygaDHJ05|[305]|DHJPAR0005037|Costa Rica|BOLD:AAA1634
Alphomelon xestopygaDHJ05|[306]|DHJPAR0012398|Costa Rica|BOLD:AAA1634
Alphomelon xestopygaDHJ05|[307]|DHJPAR0059468|Costa Rica|BOLD:AAA1634
Alphomelon xestopygaDHJ05|[308]|DHJPAR0002422|Costa Rica|BOLD:AAA1634
Alphomelon xestopygaDHJ05|[309]|DHJPAR0051223|Costa Rica|BOLD:AAA1634
Alphomelon xestopygaDHJ05|[310]|DHJPAR0031112|Costa Rica|BOLD:AAA1634
Alphomelon xestopygaDHJ05|[311]|DHJPAR0002363|Costa Rica|BOLD:AAA1634
Alphomelon xestopygaDHJ05|[312]|DHJPAR0002405|Costa Rica|BOLD:AAA1634
Alphomelon xestopygaDHJ05|[313]|DHJPAR0002424|Costa Rica|BOLD:AAA1634
Alphomelon xestopygaDHJ05|[314]|DHJPAR0012403|Costa Rica|BOLD:AAA1634
Alphomelon xestopygaDHJ05|[315]|DHJPAR0020292|Costa Rica|BOLD:AAA1634
Alphomelon xestopygaDHJ05|[316]|DHJPAR0020289|Costa Rica|BOLD:AAA1634
Alphomelon xestopygaDHJ05|[317]|DHJPAR0012406|Costa Rica|BOLD:AAA1634
Alphomelon xestopygaDHJ05|[318]|DHJPAR0002352|Costa Rica
Alphomelon xestopygaDHJ05|[319]|DHJPAR0002373|Costa Rica|BOLD:AAA1634
Alphomelon xestopygaDHJ05|[320]|DHJPAR0002491|Costa Rica|BOLD:AAA1634
Alphomelon xestopygaDHJ05|[321]|DHJPAR0002398|Costa Rica|BOLD:AAA1634
Alphomelon xestopygaDHJ05|[322]|DHJPAR0002381|Costa Rica|BOLD:AAA1634
Alphomelon xestopygaDHJ05|[323]|DHJPAR0002356|Costa Rica|BOLD:AAA1634
Alphomelon xestopygaDHJ05|[324]|DHJPAR0013166|Costa Rica|BOLD:AAA1634
Alphomelon xestopygaDHJ05|[325]|DHJPAR0058184|Costa Rica|BOLD:AAA1634
Alphomelon xestopygaDHJ05|[326]|DHJPAR0002372|Costa Rica|BOLD:AAA1634
Alphomelon xestopygaDHJ05|[327]|DHJPAR0002366|Costa Rica|BOLD:AAA1634
Alphomelon xestopygaDHJ05|[328]|DHJPAR0002380|Costa Rica|BOLD:AAA1634
Alphomelon xestopygaDHJ05|[329]|DHJPAR0002376|Costa Rica|BOLD:AAA1634
Alphomelon xestopygaDHJ05|[330]|DHJPAR0002384|Costa Rica|BOLD:AAA1634
Alphomelon xestopygaDHJ05|[331]|DHJPAR0002383|Costa Rica|BOLD:AAA1634
Alphomelon xestopygaDHJ05|[332]|DHJPAR0002389|Costa Rica|BOLD:AAA1634
Alphomelon xestopygaDHJ05|[333]|DHJPAR0002386|Costa Rica|BOLD:AAA1634
Alphomelon xestopygaDHJ05|[334]|DHJPAR0002431|Costa Rica|BOLD:AAA1634
Alphomelon xestopygaDHJ05|[335]|DHJPAR0002429|Costa Rica|BOLD:AAA1634
Alphomelon xestopygaDHJ05|[336]|DHJPAR0002420|Costa Rica|BOLD:AAA1634
Alphomelon xestopygaDHJ05|[337]|DHJPAR0002419|Costa Rica|BOLD:AAA1634
Alphomelon xestopygaDHJ05|[338]|DHJPAR0053789|Costa Rica|BOLD:AAA1634
Alphomelon xestopygaDHJ05|[339]|DHJPAR0002432|Costa Rica|BOLD:AAA1634
Alphomelon xestopygaDHJ05|[340]|DHJPAR0013159|Costa Rica|BOLD:AAA1634
Alphomelon xestopygaDHJ05|[341]|DHJPAR0013164|Costa Rica|BOLD:AAA1634
Alphomelon xestopygaDHJ05|[342]|DHJPAR0060190|Costa Rica|BOLD:AAA1634
Alphomelon xestopygaDHJ05|[343]|DHJPAR0053751|Costa Rica|BOLD:AAA1634
Alphomelon xestopygaDHJ05|[344]|DHJPAR0053758|Costa Rica|BOLD:AAA1634
Alphomelon xestopygaDHJ05|[345]|DHJPAR0013156|Costa Rica|BOLD:AAA1634
Alphomelon xestopygaDHJ05|[346]|DHJPAR0020283|Costa Rica|BOLD:AAA1634
Alphomelon xestopygaDHJ05|[347]|DHJPAR0020279|Costa Rica|BOLD:AAA1634
Alphomelon xestopygaDHJ05|[348]|DHJPAR0012393|Costa Rica|BOLD:AAA1634
Alphomelon xestopygaDHJ05|[349]|DHJPAR0012391|Costa Rica|BOLD:AAA1634
Alphomelon xestopygaDHJ05|[350]|DHJPAR0012415|Costa Rica|BOLD:AAA1634
Alphomelon xestopygaDHJ05|[351]|DHJPAR0012405|Costa Rica|BOLD:AAA1634
Alphomelon xestopygaDHJ05|[352]|DHJPAR0004661|Costa Rica|BOLD:AAA1634
Alphomelon xestopygaDHJ05|[353]|DHJPAR0004656|Costa Rica|BOLD:AAA1634
Alphomelon xestopygaDHJ05|[354]|DHJPAR0004663|Costa Rica|BOLD:AAA1634
Alphomelon xestopygaDHJ05|[355]|DHJPAR0004662|Costa Rica|BOLD:AAA1634
Alphomelon xestopygaDHJ05|[356]|DHJPAR0004645|Costa Rica|BOLD:AAA1634
Alphomelon xestopygaDHJ05|[357]|DHJPAR0004644|Costa Rica|BOLD:AAA1634
Alphomelon xestopygaDHJ05|[358]|DHJPAR0004652|Costa Rica|BOLD:AAA1634
Alphomelon xestopygaDHJ05|[359]|DHJPAR0004651|Costa Rica|BOLD:AAA1634
Alphomelon xestopygaDHJ05|[360]|DHJPAR0004673|Costa Rica|BOLD:AAA1634
Alphomelon xestopygaDHJ05|[361]|DHJPAR0004665|Costa Rica|BOLD:AAA1634
Alphomelon xestopygaDHJ05|[362]|DHJPAR0004682|Costa Rica|BOLD:AAA1634
Alphomelon xestopygaDHJ05|[363]|DHJPAR0004677|Costa Rica|BOLD:AAA1634
Alphomelon xestopygaDHJ05|[364]|DHJPAR0058169|Costa Rica|BOLD:AAA1634
Alphomelon xestopygaDHJ05|[365]|DHJPAR0002437|Costa Rica|BOLD:AAA1634
Alphomelon xestopygaDHJ05|[366]|DHJPAR0012122|Costa Rica|BOLD:AAA1634
Alphomelon xestopygaDHJ05|[367]|DHJPAR0012119|Costa Rica|BOLD:AAA1634
Alphomelon xestopygaDHJ05|[368]|DHJPAR0058229|Costa Rica|BOLD:AAA1634
Alphomelon xestopygaDHJ05|[369]|DHJPAR0049842|Costa Rica|BOLD:AAA1634
Alphomelon xestopygaDHJ05|[370]|DHJPAR0058210|Costa Rica|BOLD:AAA1634
Alphomelon xestopygaDHJ05|[371]|DHJPAR0031006|Costa Rica|BOLD:AAA1634
Alphomelon xestopygaDHJ05|[372]|DHJPAR0002439|Costa Rica|BOLD:AAA1634
Alphomelon xestopygaDHJ05|[373]|DHJPAR0058220|Costa Rica|BOLD:AAA1634
Alphomelon xestopygaDHJ05|[374]|DHJPAR0059740|Costa Rica|BOLD:AAA1634
Alphomelon xestopygaDHJ05|[375]|DHJPAR0013170|Costa Rica|BOLD:AAA1634
Alphomelon xestopygaDHJ05|[376]|DHJPAR0013153|Costa Rica|BOLD:AAA1634
Alphomelon xestopygaDHJ05|[377]|DHJPAR0013172|Costa Rica|BOLD:AAA1634
Alphomelon xestopygaDHJ05|[378]|DHJPAR0004933|Costa Rica|BOLD:AAA1634
Alphomelon xestopygaDHJ05|[379]|DHJPAR0002410|Costa Rica|BOLD:AAA1634
Alphomelon xestopygaDHJ05|[380]|DHJPAR0031125|Costa Rica|BOLD:AAA1634
Alphomelon xestopygaDHJ05|[381]|DHJPAR0031123|Costa Rica|BOLD:AAA1634
Alphomelon xestopygaDHJ05|[382]|DHJPAR0031134|Costa Rica|BOLD:AAA1634
Alphomelon xestopygaDHJ05|[383]|DHJPAR0031127|Costa Rica|BOLD:AAA1634

[illegible]
Alphomelon xestopygaDHJ05|[381]|DHJPAR0031123|Costa Rica|BOLD:AAA1634
Alphomelon xestopygaDHJ05|[382]|DHJPAR0031134|Costa Rica|BOLD:AAA1634
Alphomelon xestopygaDHJ05|[383]|DHJPAR0031127|Costa Rica|BOLD:AAA1634
Alphomelon xestopygaDHJ05|[384]|DHJPAR0060609|Costa Rica|BOLD:AAA1634
Alphomelon xestopygaDHJ05|[385]|DHJPAR0005044|Costa Rica|BOLD:AAA1634
Alphomelon xestopygaDHJ05|[386]|DHJPAR0005059|Costa Rica|BOLD:AAA1634
Alphomelon xestopygaDHJ05|[387]|DHJPAR0002430|Costa Rica|BOLD:AAA1634
Alphomelon xestopygaDHJ05|[388]|DHJPAR0031110|Costa Rica|BOLD:AAA1634
Alphomelon xestopygaDHJ05|[389]|DHJPAR0031133|Costa Rica|BOLD:AAA1634
Alphomelon xestopygaDHJ05|[390]|DHJPAR0002364|Costa Rica|BOLD:AAA1634
Alphomelon xestopygaDHJ05|[391]|DHJPAR0002342|Costa Rica|BOLD:AAA1634
Alphomelon xestopygaDHJ05|[392]|DHJPAR0060602|Costa Rica|BOLD:AAA1634
Alphomelon xestopygaDHJ05|[393]|DHJPAR0002371|Costa Rica|BOLD:AAA1634
Alphomelon xestopygaDHJ05|[394]|DHJPAR0002358|Costa Rica|BOLD:AAA1634
Alphomelon xestopygaDHJ05|[395]|DHJPAR0002375|Costa Rica|BOLD:AAA1634
Alphomelon xestopygaDHJ05|[396]|DHJPAR0002407|Costa Rica|BOLD:AAA1634
Alphomelon xestopygaDHJ05|[397]|DHJPAR0002406|Costa Rica|BOLD:AAA1634
Alphomelon xestopygaDHJ05|[398]|DHJPAR0012703|Costa Rica|BOLD:AAA1634
Alphomelon xestopygaDHJ05|[399]|DHJPAR0002415|Costa Rica|BOLD:AAA1634
Alphomelon xestopygaDHJ05|[400]|DHJPAR0060596|Costa Rica|BOLD:AAA1634
Alphomelon xestopygaDHJ05|[401]|DHJPAR0030697|Costa Rica|BOLD:AAA1634
Alphomelon xestopygaDHJ05|[402]|DHJPAR0060597|Costa Rica|BOLD:AAA1634
Alphomelon xestopygaDHJ05|[403]|DHJPAR0002428|Costa Rica|BOLD:AAA1634
Alphomelon xestopygaDHJ05|[404]|DHJPAR0004931|Costa Rica|BOLD:AAA1634
Alphomelon xestopygaDHJ05|[405]|DHJPAR0002387|Costa Rica|BOLD:AAA1634
Alphomelon xestopygaDHJ05|[406]|DHJPAR0002391|Costa Rica|BOLD:AAA1634
Alphomelon xestopygaDHJ05|[407]|DHJPAR0004897|Costa Rica|BOLD:AAA1634
Alphomelon xestopygaDHJ05|[408]|DHJPAR0004946|Costa Rica|BOLD:AAA1634
Alphomelon xestopygaDHJ05|[409]|DHJPAR0005022|Costa Rica|BOLD:AAA1634
Alphomelon xestopygaDHJ05|[410]|DHJPAR0002409|Costa Rica|BOLD:AAA1634
Alphomelon xestopygaDHJ05|[411]|DHJPAR0013162|Costa Rica|BOLD:AAA1634
Alphomelon xestopygaDHJ05|[412]|DHJPAR0002350|Costa Rica|BOLD:AAA1634
Alphomelon xestopygaDHJ05|[413]|DHJPAR0002418|Costa Rica|BOLD:AAA1634
Alphomelon xestopygaDHJ05|[414]|DHJPAR0003013|Costa Rica|BOLD:AAA1634
Alphomelon xestopygaDHJ05|[415]|BIOUG09826-D03|Costa Rica|BOLD:AAA1634
Alphomelon xestopygaDHJ05|[416]|DHJPAR0002436|Costa Rica
Alphomelon xestopygaDHJ05|[417]|DHJPAR0004650|Costa Rica|BOLD:AAA1634
Alphomelon xestopygaDHJ05|[418]|DHJPAR0004900|Costa Rica|BOLD:AAA1634
Alphomelon xestopygaDHJ05|[419]|DHJPAR0013160|Costa Rica|BOLD:AAA1634
Alphomelon xestopygaDHJ05|[420]|DHJPAR0002365|Costa Rica|BOLD:AAA1634
Alphomelon xestopygaDHJ05|[421]|DHJPAR0002362|Costa Rica|BOLD:AAA1634
Alphomelon xestopygaDHJ05|[422]|DHJPAR0002353|Costa Rica|BOLD:AAA1634
Alphomelon xestopygaDHJ05|[423]|DHJPAR0011846|Costa Rica|BOLD:AAA1634
Alphomelon xestopygaDHJ05|[424]|DHJPAR0053760|Costa Rica|BOLD:AAA1634
Alphomelon xestopygaDHJ05|[425]|DHJPAR0004959|Costa Rica|BOLD:AAA1634
Alphomelon xestopygaDHJ05|[426]|DHJPAR0020277|Costa Rica|BOLD:AAA1634
Alphomelon xestopygaDHJ05|[427]|DHJPAR0058880|Costa Rica|BOLD:AAA1634
Alphomelon xestopygaDHJ05|[428]|DHJPAR0002461|Costa Rica|BOLD:AAA1634
Alphomelon xestopygaDHJ05|[429]|DHJPAR0053767|Costa Rica|BOLD:AAA1634
Alphomelon xestopygaDHJ05|[430]|DHJPAR0058940|Costa Rica|BOLD:AAA1634
Alphomelon xestopygaDHJ05|[431]|DHJPAR0058930|Costa Rica|BOLD:AAA1634
Alphomelon xestopygaDHJ05|[432]|DHJPAR0058903|Costa Rica|BOLD:AAA1634
Alphomelon xestopygaDHJ05|[433]|DHJPAR0004902|Costa Rica|BOLD:AAA1634
Alphomelon xestopygaDHJ05|[434]|DHJPAR0052957|Costa Rica|BOLD:AAA1634
Alphomelon xestopygaDHJ05|[435]|DHJPAR0012423|Costa Rica|BOLD:AAA1634
Alphomelon xestopygaDHJ05|[436]|DHJPAR0012421|Costa Rica|BOLD:AAA1634
Alphomelon xestopygaDHJ05|[437]|DHJPAR0002458|Costa Rica|BOLD:AAA1634
Alphomelon xestopygaDHJ05|[438]|DHJPAR0012426|Costa Rica|BOLD:AAA1634
Alphomelon xestopygaDHJ05|[439]|DHJPAR0002490|Costa Rica|BOLD:AAA1634
Alphomelon xestopygaDHJ05|[440]|DHJPAR0002488|Costa Rica|BOLD:AAA1634
Alphomelon xestopygaDHJ05|[441]|DHJPAR0061770|Costa Rica|BOLD:AAA1634
Alphomelon xestopygaDHJ05|[442]|DHJPAR0058180|Costa Rica|BOLD:AAA1634
Alphomelon xestopygaDHJ05|[443]|DHJPAR0013169|Costa Rica|BOLD:AAA1634
Alphomelon xestopygaDHJ05|[444]|DHJPAR0061809|Costa Rica|BOLD:AAA1634
Alphomelon xestopygaDHJ05|[445]|DHJPAR0059720|Costa Rica|BOLD:AAA1634
Alphomelon xestopygaDHJ05|[446]|DHJPAR0058233|Costa Rica|BOLD:AAA1634
Alphomelon xestopygaDHJ05|[447]|DHJPAR0002385|Costa Rica|BOLD:AAA1634
Alphomelon xestopygaDHJ05|[448]|DHJPAR0004957|Costa Rica|BOLD:AAA1634
Alphomelon xestopygaDHJ05|[449]|DHJPAR0013171|Costa Rica|BOLD:AAA1634
Alphomelon xestopygaDHJ05|[450]|DHJPAR0012876|Costa Rica|BOLD:AAA1634
Alphomelon xestopygaDHJ05|[451]|DHJPAR0013161|Costa Rica|BOLD:AAA1634
Alphomelon xestopygaDHJ05|[452]|DHJPAR0059728|Costa Rica|BOLD:AAA1634
Alphomelon xestopygaDHJ05|[453]|DHJPAR0059729|Costa Rica|BOLD:AAA1634
Alphomelon xestopygaDHJ05|[454]|DHJPAR0004936|Costa Rica|BOLD:AAA1634
Alphomelon xestopygaDHJ05|[455]|DHJPAR0031113|Costa Rica|BOLD:AAA1634
Alphomelon xestopygaDHJ05|[456]|DHJPAR0031121|Costa Rica|BOLD:AAA1634
Alphomelon xestopygaDHJ05|[457]|DHJPAR0053792|Costa Rica|BOLD:AAA1634
Alphomelon xestopygaDHJ05|[458]|DHJPAR0005039|Costa Rica|BOLD:AAA1634
Alphomelon xestopygaDHJ05|[459]|DHJPAR0002413|Costa Rica|BOLD:AAA1634
Alphomelon xestopygaDHJ05|[460]|DHJPAR0031616|Costa Rica|BOLD:AAA1634
Alphomelon xestopygaDHJ05|[461]|DHJPAR0013154|Costa Rica|BOLD:AAA1634
Alphomelon xestopygaDHJ05|[462]|DHJPAR0031114|Costa Rica|BOLD:AAA1634
Alphomelon xestopygaDHJ05|[463]|DHJPAR0031117|Costa Rica|BOLD:AAA1634
Alphomelon xestopygaDHJ05|[464]|DHJPAR0012128|Costa Rica|BOLD:AAA1634
Alphomelon xestopygaDHJ05|[465]|DHJPAR0002446|Costa Rica
Alphomelon xestopygaDHJ05|[466]|DHJPAR0058849|Costa Rica|BOLD:AAA1634
Alphomelon xestopygaDHJ05|[467]|DHJPAR0056746|Costa Rica|BOLD:AAA1634
Alphomelon xestopygaDHJ05|[468]|DHJPAR0030809|Costa Rica|BOLD:AAA1634
Alphomelon xestopygaDHJ05|[469]|DHJPAR0012395|Costa Rica|BOLD:AAA1634
Alphomelon xestopygaDHJ05|[470]|DHJPAR0004966|Costa Rica|BOLD:AAA1634
Alphomelon xestopygaDHJ05|[471]|DHJPAR0059753|Costa Rica|BOLD:AAA1634
Alphomelon xestopygaDHJ05|[472]|DHJPAR0002369|Costa Rica|BOLD:AAA1634
Alphomelon xestopygaDHJ05|[473]|DHJPAR0031682|Costa Rica|BOLD:AAA1634
Alphomelon xestopygaDHJ05|[474]|DHJPAR0002426|Costa Rica|BOLD:AAA1634
Alphomelon xestopygaDHJ05|[475]|DHJPAR0002404|Costa Rica|BOLD:AAA1634
Alphomelon xestopygaDHJ05|[476]|DHJPAR0002345|Costa Rica|BOLD:AAA1634
Alphomelon xestopygaDHJ05|[477]|DHJPAR0002390|Costa Rica|BOLD:AAA1634
Alphomelon xestopygaDHJ05|[478]|DHJPAR0002378|Costa Rica|BOLD:AAA1634

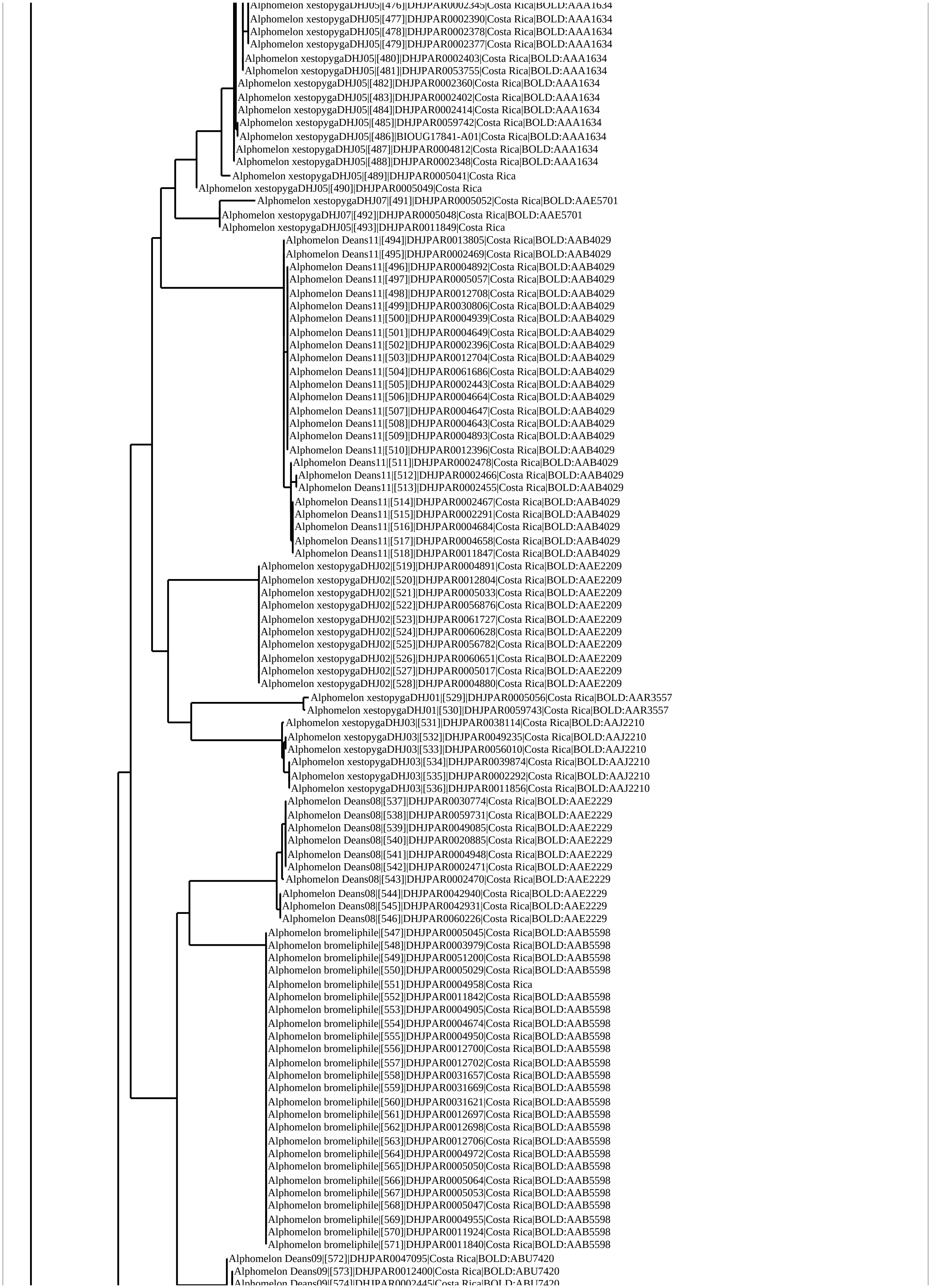
Alphomelon xestopygaDHJ05|[476]|DHJPAR0002345|Costa Rica|BOLD:AAA1634
Alphomelon xestopygaDHJ05|[477]|DHJPAR0002390|Costa Rica|BOLD:AAA1634
Alphomelon xestopygaDHJ05|[478]|DHJPAR0002378|Costa Rica|BOLD:AAA1634
Alphomelon xestopygaDHJ05|[479]|DHJPAR0002377|Costa Rica|BOLD:AAA1634
Alphomelon xestopygaDHJ05|[480]|DHJPAR0002403|Costa Rica|BOLD:AAA1634
Alphomelon xestopygaDHJ05|[481]|DHJPAR0053755|Costa Rica|BOLD:AAA1634
Alphomelon xestopygaDHJ05|[482]|DHJPAR0002360|Costa Rica|BOLD:AAA1634
Alphomelon xestopygaDHJ05|[483]|DHJPAR0002402|Costa Rica|BOLD:AAA1634
Alphomelon xestopygaDHJ05|[484]|DHJPAR0002414|Costa Rica|BOLD:AAA1634
Alphomelon xestopygaDHJ05|[485]|DHJPAR0059742|Costa Rica|BOLD:AAA1634
Alphomelon xestopygaDHJ05|[486]|BIOUG17841-A01|Costa Rica|BOLD:AAA1634
Alphomelon xestopygaDHJ05|[487]|DHJPAR0004812|Costa Rica|BOLD:AAA1634
Alphomelon xestopygaDHJ05|[488]|DHJPAR0002348|Costa Rica|BOLD:AAA1634
Alphomelon xestopygaDHJ05|[489]|DHJPAR0005041|Costa Rica
Alphomelon xestopygaDHJ05|[490]|DHJPAR0005049|Costa Rica
Alphomelon xestopygaDHJ07|[491]|DHJPAR0005052|Costa Rica|BOLD:AAE5701
Alphomelon xestopygaDHJ07|[492]|DHJPAR0005048|Costa Rica|BOLD:AAE5701
Alphomelon xestopygaDHJ05|[493]|DHJPAR0011849|Costa Rica
Alphomelon Deans11|[494]|DHJPAR0013805|Costa Rica|BOLD:AAB4029
Alphomelon Deans11|[495]|DHJPAR0002469|Costa Rica|BOLD:AAB4029
Alphomelon Deans11|[496]|DHJPAR0004892|Costa Rica|BOLD:AAB4029
Alphomelon Deans11|[497]|DHJPAR0005057|Costa Rica|BOLD:AAB4029
Alphomelon Deans11|[498]|DHJPAR0012708|Costa Rica|BOLD:AAB4029
Alphomelon Deans11|[499]|DHJPAR0030806|Costa Rica|BOLD:AAB4029
Alphomelon Deans11|[500]|DHJPAR0004939|Costa Rica|BOLD:AAB4029
Alphomelon Deans11|[501]|DHJPAR0004649|Costa Rica|BOLD:AAB4029
Alphomelon Deans11|[502]|DHJPAR0002396|Costa Rica|BOLD:AAB4029
Alphomelon Deans11|[503]|DHJPAR0012704|Costa Rica|BOLD:AAB4029
Alphomelon Deans11|[504]|DHJPAR0061686|Costa Rica|BOLD:AAB4029
Alphomelon Deans11|[505]|DHJPAR0002443|Costa Rica|BOLD:AAB4029
Alphomelon Deans11|[506]|DHJPAR0004664|Costa Rica|BOLD:AAB4029
Alphomelon Deans11|[507]|DHJPAR0004647|Costa Rica|BOLD:AAB4029
Alphomelon Deans11|[508]|DHJPAR0004643|Costa Rica|BOLD:AAB4029
Alphomelon Deans11|[509]|DHJPAR0004893|Costa Rica|BOLD:AAB4029
Alphomelon Deans11|[510]|DHJPAR0012396|Costa Rica|BOLD:AAB4029
Alphomelon Deans11|[511]|DHJPAR0002478|Costa Rica|BOLD:AAB4029
Alphomelon Deans11|[512]|DHJPAR0002466|Costa Rica|BOLD:AAB4029
Alphomelon Deans11|[513]|DHJPAR0002455|Costa Rica|BOLD:AAB4029
Alphomelon Deans11|[514]|DHJPAR0002467|Costa Rica|BOLD:AAB4029
Alphomelon Deans11|[515]|DHJPAR0002291|Costa Rica|BOLD:AAB4029
Alphomelon Deans11|[516]|DHJPAR0004684|Costa Rica|BOLD:AAB4029
Alphomelon Deans11|[517]|DHJPAR0004658|Costa Rica|BOLD:AAB4029
Alphomelon Deans11|[518]|DHJPAR0011847|Costa Rica|BOLD:AAB4029
Alphomelon xestopygaDHJ02|[519]|DHJPAR0004891|Costa Rica|BOLD:AAE2209
Alphomelon xestopygaDHJ02|[520]|DHJPAR0012804|Costa Rica|BOLD:AAE2209
Alphomelon xestopygaDHJ02|[521]|DHJPAR0005033|Costa Rica|BOLD:AAE2209
Alphomelon xestopygaDHJ02|[522]|DHJPAR0056876|Costa Rica|BOLD:AAE2209
Alphomelon xestopygaDHJ02|[523]|DHJPAR0061727|Costa Rica|BOLD:AAE2209
Alphomelon xestopygaDHJ02|[524]|DHJPAR0060628|Costa Rica|BOLD:AAE2209
Alphomelon xestopygaDHJ02|[525]|DHJPAR0056782|Costa Rica|BOLD:AAE2209
Alphomelon xestopygaDHJ02|[526]|DHJPAR0060651|Costa Rica|BOLD:AAE2209
Alphomelon xestopygaDHJ02|[527]|DHJPAR0005017|Costa Rica|BOLD:AAE2209
Alphomelon xestopygaDHJ02|[528]|DHJPAR0004880|Costa Rica|BOLD:AAE2209
Alphomelon xestopygaDHJ01|[529]|DHJPAR0005056|Costa Rica|BOLD:AAR3557
Alphomelon xestopygaDHJ01|[530]|DHJPAR0059743|Costa Rica|BOLD:AAR3557
Alphomelon xestopygaDHJ03|[531]|DHJPAR0038114|Costa Rica|BOLD:AAJ2210
Alphomelon xestopygaDHJ03|[532]|DHJPAR0049235|Costa Rica|BOLD:AAJ2210
Alphomelon xestopygaDHJ03|[533]|DHJPAR0056010|Costa Rica|BOLD:AAJ2210
Alphomelon xestopygaDHJ03|[534]|DHJPAR0039874|Costa Rica|BOLD:AAJ2210
Alphomelon xestopygaDHJ03|[535]|DHJPAR0002292|Costa Rica|BOLD:AAJ2210
Alphomelon xestopygaDHJ03|[536]|DHJPAR0011856|Costa Rica|BOLD:AAJ2210
Alphomelon Deans08|[537]|DHJPAR0030774|Costa Rica|BOLD:AAE2229
Alphomelon Deans08|[538]|DHJPAR0059731|Costa Rica|BOLD:AAE2229
Alphomelon Deans08|[539]|DHJPAR0049085|Costa Rica|BOLD:AAE2229
Alphomelon Deans08|[540]|DHJPAR0020885|Costa Rica|BOLD:AAE2229
Alphomelon Deans08|[541]|DHJPAR0004948|Costa Rica|BOLD:AAE2229
Alphomelon Deans08|[542]|DHJPAR0002471|Costa Rica|BOLD:AAE2229
Alphomelon Deans08|[543]|DHJPAR0002470|Costa Rica|BOLD:AAE2229
Alphomelon Deans08|[544]|DHJPAR0042940|Costa Rica|BOLD:AAE2229
Alphomelon Deans08|[545]|DHJPAR0042931|Costa Rica|BOLD:AAE2229
Alphomelon Deans08|[546]|DHJPAR0060226|Costa Rica|BOLD:AAE2229
Alphomelon bromeliphile|[547]|DHJPAR0005045|Costa Rica|BOLD:AAB5598
Alphomelon bromeliphile|[548]|DHJPAR0003979|Costa Rica|BOLD:AAB5598
Alphomelon bromeliphile|[549]|DHJPAR0051200|Costa Rica|BOLD:AAB5598
Alphomelon bromeliphile|[550]|DHJPAR0005029|Costa Rica|BOLD:AAB5598
Alphomelon bromeliphile|[551]|DHJPAR0004958|Costa Rica
Alphomelon bromeliphile|[552]|DHJPAR0011842|Costa Rica|BOLD:AAB5598
Alphomelon bromeliphile|[553]|DHJPAR0004905|Costa Rica|BOLD:AAB5598
Alphomelon bromeliphile|[554]|DHJPAR0004674|Costa Rica|BOLD:AAB5598
Alphomelon bromeliphile|[555]|DHJPAR0004950|Costa Rica|BOLD:AAB5598
Alphomelon bromeliphile|[556]|DHJPAR0012700|Costa Rica|BOLD:AAB5598
Alphomelon bromeliphile|[557]|DHJPAR0012702|Costa Rica|BOLD:AAB5598
Alphomelon bromeliphile|[558]|DHJPAR0031657|Costa Rica|BOLD:AAB5598
Alphomelon bromeliphile|[559]|DHJPAR0031669|Costa Rica|BOLD:AAB5598
Alphomelon bromeliphile|[560]|DHJPAR0031621|Costa Rica|BOLD:AAB5598
Alphomelon bromeliphile|[561]|DHJPAR0012697|Costa Rica|BOLD:AAB5598
Alphomelon bromeliphile|[562]|DHJPAR0012698|Costa Rica|BOLD:AAB5598
Alphomelon bromeliphile|[563]|DHJPAR0012706|Costa Rica|BOLD:AAB5598
Alphomelon bromeliphile|[564]|DHJPAR0004972|Costa Rica|BOLD:AAB5598
Alphomelon bromeliphile|[565]|DHJPAR0005050|Costa Rica|BOLD:AAB5598
Alphomelon bromeliphile|[566]|DHJPAR0005064|Costa Rica|BOLD:AAB5598
Alphomelon bromeliphile|[567]|DHJPAR0005053|Costa Rica|BOLD:AAB5598
Alphomelon bromeliphile|[568]|DHJPAR0005047|Costa Rica|BOLD:AAB5598
Alphomelon bromeliphile|[569]|DHJPAR0004955|Costa Rica|BOLD:AAB5598
Alphomelon bromeliphile|[570]|DHJPAR0011924|Costa Rica|BOLD:AAB5598
Alphomelon bromeliphile|[571]|DHJPAR0011840|Costa Rica|BOLD:AAB5598
Alphomelon Deans09|[572]|DHJPAR0047095|Costa Rica|BOLD:ABU7420
Alphomelon Deans09|[573]|DHJPAR0012400|Costa Rica|BOLD:ABU7420
Alphomelon Deans09|[574]|DHJPAR0002445|Costa Rica|BOLD:ABU7420

Alphomelon Deans09|[572]|DHJPAR0047095|Costa Rica|BOLD:ABU7420
Alphomelon Deans09|[573]|DHJPAR0012400|Costa Rica|BOLD:ABU7420
Alphomelon Deans09|[574]|DHJPAR0002445|Costa Rica|BOLD:ABU7420
Alphomelon Deans09|[575]|DHJPAR0047120|Costa Rica|BOLD:ABU7420
Alphomelon Deans09|[576]|DHJPAR0057788|Costa Rica|BOLD:ABU7420
Alphomelon Deans09|[577]|DHJPAR0049095|Costa Rica|BOLD:ABU7420
Alphomelon Deans09|[578]|DHJPAR0057786|Costa Rica|BOLD:ABU7420
Alphomelon Deans09|[579]|DHJPAR0012416|Costa Rica|BOLD:ABU7420
Alphomelon Deans09|[580]|DHJPAR0038112|Costa Rica|BOLD:ABU7420
Alphomelon Deans09|[581]|DHJPAR0041758|Costa Rica|BOLD:ABU7420
Alphomelon Deans09|[582]|DHJPAR0041807|Costa Rica|BOLD:ABU7420
Alphomelon Deans09|[583]|DHJPAR0049225|Costa Rica|BOLD:ABU7420
Alphomelon Deans09|[584]|DHJPAR0041820|Costa Rica|BOLD:ABU7420
Alphomelon Deans09|[585]|DHJPAR0025653|Costa Rica|BOLD:ABU7420
Alphomelon Deans09|[586]|DHJPAR0057793|Costa Rica|BOLD:ABU7420
Alphomelon Deans09|[587]|DHJPAR0012701|Costa Rica|BOLD:ABU7420
Alphomelon Deans09|[588]|DHJPAR0004885|Costa Rica|BOLD:ABU7420
Alphomelon Deans09|[589]|DHJPAR0045224|Costa Rica|BOLD:ABU7420
Alphomelon Deans09|[590]|DHJPAR0004935|Costa Rica|BOLD:ABU7420
Alphomelon Deans09|[591]|DHJPAR0004940|Costa Rica|BOLD:ABU7420
Alphomelon Deans09|[592]|DHJPAR0005116|Costa Rica|BOLD:ABU7420
Alphomelon Deans09|[593]|DHJPAR0004882|Costa Rica|BOLD:ABU7420
Alphomelon Deans09|[594]|DHJPAR0012857|Costa Rica|BOLD:ABU7420
Alphomelon Deans09|[595]|DHJPAR0059482|Costa Rica|BOLD:ABU7420
Alphomelon Deans09|[596]|DHJPAR0038270|Costa Rica|BOLD:ABU7420
Alphomelon Deans09|[597]|DHJPAR0058212|Costa Rica|BOLD:ABU7420
Alphomelon Deans09|[598]|DHJPAR0058207|Costa Rica|BOLD:ABU7420
Alphomelon Deans09|[599]|DHJPAR0058201|Costa Rica|BOLD:ABU7420
Alphomelon Deans09|[600]|DHJPAR0058161|Costa Rica|BOLD:ABU7420
Alphomelon Deans09|[601]|DHJPAR0058153|Costa Rica|BOLD:ABU7420
Alphomelon Deans09|[602]|DHJPAR0062108|Costa Rica|BOLD:ABU7420
Alphomelon Deans09|[603]|DHJPAR0058838|Costa Rica|BOLD:ABU7420
Alphomelon Deans09|[604]|DHJPAR0012699|Costa Rica|BOLD:ABU7420
Alphomelon Deans09|[605]|DHJPAR0038962|Costa Rica|BOLD:ABU7420
Alphomelon Deans09|[606]|DHJPAR0038957|Costa Rica|BOLD:ABU7420
Alphomelon Deans09|[607]|DHJPAR0012859|Costa Rica|BOLD:ABU7420
Alphomelon|[608]|CCDB-07374 F11|French Guiana|BOLD:AAV7443
Alphomelon Deans12|[609]|DHJPAR0004805|Costa Rica|BOLD:AAB7535
Alphomelon Deans12|[610]|DHJPAR0031126|Costa Rica|BOLD:AAB7535
Alphomelon Deans12|[611]|DHJPAR0004654|Costa Rica|BOLD:AAB7535
Alphomelon Deans12|[612]|DHJPAR0005019|Costa Rica|BOLD:AAB7535
Alphomelon Deans12|[613]|DHJPAR0004917|Costa Rica|BOLD:AAB7535
Alphomelon Deans12|[614]|DHJPAR0012414|Costa Rica|BOLD:AAB7535
Alphomelon Deans12|[615]|DHJPAR0002460|Costa Rica|BOLD:AAB7535
Alphomelon Deans12|[616]|DHJPAR0058188|Costa Rica|BOLD:AAB7535
Alphomelon Deans12|[617]|DHJPAR0058190|Costa Rica|BOLD:AAB7535
Alphomelon Deans12|[618]|DHJPAR0058209|Costa Rica|BOLD:AAB7535
Alphomelon Deans12|[619]|DHJPAR0054695|Costa Rica|BOLD:AAB7535
Alphomelon Deans12|[620]|DHJPAR0064147|Costa Rica|BOLD:AAB7535
Alphomelon Deans12|[621]|DHJPAR0012120|Costa Rica|BOLD:AAB7535
Alphomelon Deans12|[622]|DHJPAR0030875|Costa Rica|BOLD:AAB7535
Alphomelon Deans12|[623]|DHJPAR0030883|Costa Rica|BOLD:AAB7535
Alphomelon Deans12|[624]|DHJPAR0030932|Costa Rica|BOLD:AAB7535
Alphomelon Deans12|[625]|DHJPAR0020276|Costa Rica|BOLD:AAB7535
Alphomelon Deans12|[626]|DHJPAR0012710|Costa Rica|BOLD:AAB7535
Alphomelon Deans12|[627]|DHJPAR0004924|Costa Rica|BOLD:AAB7535
Alphomelon Deans12|[628]|DHJPAR0025847|Costa Rica|BOLD:AAB7535
Alphomelon Deans12|[629]|BIOUG19726-B08|Costa Rica|BOLD:AAB7535
Alphomelon Deans12|[630]|BIOUG28011-A06|Costa Rica|BOLD:AAB7535
Alphomelon Deans12|[631]|DHJPAR0004888|Costa Rica|BOLD:AAB7535
Alphomelon Deans12|[632]|DHJPAR0020280|Costa Rica|BOLD:AAB7535
Alphomelon Deans12|[633]|DHJPAR0058963|Costa Rica|BOLD:AAB7535
Alphomelon Deans12|[634]|DHJPAR0058962|Costa Rica|BOLD:AAB7535
Alphomelon Deans12|[635]|DHJPAR0012863|Costa Rica|BOLD:AAB7535
Alphomelon Deans12|[636]|DHJPAR0060154|Costa Rica|BOLD:AAB7535
Alphomelon Deans13|[637]|DHJPAR0004802|Costa Rica|BOLD:AAA6775
Alphomelon Deans13|[638]|DHJPAR0057765|Costa Rica|BOLD:AAA6775
Alphomelon Deans13|[639]|DHJPAR0031132|Costa Rica|BOLD:AAA6775
Alphomelon Deans13|[640]|DHJPAR0004898|Costa Rica|BOLD:AAA6775
Alphomelon Deans13|[641]|DHJPAR0004949|Costa Rica|BOLD:AAA6775
Alphomelon Deans13|[642]|DHJPAR0005061|Costa Rica|BOLD:AAA6775
Alphomelon Deans13|[643]|DHJPAR0058160|Costa Rica|BOLD:AAA6775
Alphomelon Deans13|[644]|DHJPAR0048159|Costa Rica|BOLD:AAA6775
Alphomelon Deans13|[645]|DHJPAR0012707|Costa Rica|BOLD:AAA6775
Alphomelon Deans13|[646]|DHJPAR0057515|Costa Rica|BOLD:AAA6775
Alphomelon Deans13|[647]|DHJPAR0060156|Costa Rica|BOLD:AAA6775
Alphomelon Deans13|[648]|DHJPAR0002498|Costa Rica|BOLD:AAA6775
Alphomelon Deans13|[649]|DHJPAR0050981|Costa Rica|BOLD:AAA6775
Alphomelon Deans13|[650]|DHJPAR0061754|Costa Rica|BOLD:AAA6775
Alphomelon Deans13|[651]|DHJPAR0058205|Costa Rica|BOLD:AAA6775
Alphomelon Deans13|[652]|DHJPAR0062582|Costa Rica|BOLD:AAA6775
Alphomelon Deans13|[653]|DHJPAR0062590|Costa Rica|BOLD:AAA6775
Alphomelon Deans13|[654]|DHJPAR0062593|Costa Rica|BOLD:AAA6775
Alphomelon Deans13|[655]|DHJPAR0058867|Costa Rica|BOLD:AAA6775
Alphomelon Deans13|[656]|DHJPAR0025786|Costa Rica|BOLD:AAA6775
Alphomelon Deans13|[657]|DHJPAR0020596|Costa Rica|BOLD:AAA6775
Alphomelon Deans13|[658]|DHJPAR0004937|Costa Rica|BOLD:AAA6775
Alphomelon Deans13|[659]|DHJPAR0004969|Costa Rica|BOLD:AAA6775
Alphomelon Deans13|[660]|DHJPAR0004971|Costa Rica|BOLD:AAA6775
Alphomelon Deans13|[661]|DHJPAR0060613|Costa Rica|BOLD:AAA6775
Alphomelon Deans13|[662]|DHJPAR0005060|Costa Rica|BOLD:AAA6775
Alphomelon Deans13|[663]|DHJPAR0005114|Costa Rica|BOLD:AAA6775
Alphomelon Deans13|[664]|DHJPAR0054746|Costa Rica|BOLD:AAA6775
Alphomelon Deans13|[665]|DHJPAR0056706|Costa Rica|BOLD:AAA6775
Alphomelon Deans13|[666]|DHJPAR0061719|Costa Rica|BOLD:AAA6775
Alphomelon Deans13|[667]|DHJPAR0025617|Costa Rica|BOLD:AAA6775
Alphomelon Deans13|[668]|DHJPAR0053774|Costa Rica|BOLD:AAA6775
Alphomelon Deans13|[669]|DHJPAR0060621|Costa Rica|BOLD:AAA6775

Alphomelon Deans13|[667]|DHJPAR0025617|Costa Rica|BOLD:AAA6775
Alphomelon Deans13|[668]|DHJPAR0053774|Costa Rica|BOLD:AAA6775
Alphomelon Deans13|[669]|DHJPAR0060621|Costa Rica|BOLD:AAA6775
Alphomelon Deans13|[670]|DHJPAR0060653|Costa Rica|BOLD:AAA6775
Alphomelon Deans13|[671]|DHJPAR0053717|Costa Rica|BOLD:AAA6775
Alphomelon Deans13|[672]|DHJPAR0056368|Costa Rica|BOLD:AAA6775
Alphomelon Deans13|[673]|DHJPAR0030714|Costa Rica|BOLD:AAA6775
Alphomelon Deans13|[674]|DHJPAR0002457|Costa Rica|BOLD:AAA6775
Alphomelon Deans13|[675]|DHJPAR0004657|Costa Rica|BOLD:AAA6775
Alphomelon Deans13|[676]|DHJPAR0004919|Costa Rica|BOLD:AAA6775
Alphomelon Deans13|[677]|DHJPAR0004894|Costa Rica|BOLD:AAA6775
Alphomelon Deans13|[678]|DHJPAR0012399|Costa Rica|BOLD:AAA6775
Alphomelon Deans13|[679]|DHJPAR0061661|Costa Rica|BOLD:AAA6775
Alphomelon Deans13|[680]|DHJPAR0020281|Costa Rica|BOLD:AAA6775
Alphomelon Deans13|[681]|DHJPAR0058938|Costa Rica|BOLD:AAA6775
Alphomelon Deans13|[682]|DHJPAR0058935|Costa Rica|BOLD:AAA6775
Alphomelon Deans13|[683]|DHJPAR0058921|Costa Rica|BOLD:AAA6775
Alphomelon Deans13|[684]|DHJPAR0058916|Costa Rica|BOLD:AAA6775
Alphomelon Deans13|[685]|DHJPAR0058893|Costa Rica|BOLD:AAA6775
Alphomelon Deans13|[686]|DHJPAR0058877|Costa Rica|BOLD:AAA6775
Alphomelon Deans13|[687]|DHJPAR0058847|Costa Rica|BOLD:AAA6775
Alphomelon Deans13|[688]|DHJPAR0056693|Costa Rica|BOLD:AAA6775
Alphomelon Deans13|[689]|DHJPAR0012855|Costa Rica|BOLD:AAA6775
Alphomelon Deans13|[690]|DHJPAR0046873|Costa Rica|BOLD:AAA6775
Alphomelon Deans13|[691]|DHJPAR0064082|Costa Rica|BOLD:AAA6775
Alphomelon Deans13|[692]|DHJPAR0026885|Costa Rica|BOLD:AAA6775
Alphomelon Deans13|[693]|DHJPAR0005113|Costa Rica|BOLD:AAA6775
Alphomelon Deans13|[694]|DHJPAR0025492|Costa Rica|BOLD:AAA6775
Alphomelon Deans13|[695]|DHJPAR0005115|Costa Rica|BOLD:AAA6775
Alphomelon Deans13|[696]|DHJPAR0004930|Costa Rica|BOLD:AAA6775
Alphomelon Deans13|[697]|DHJPAR0030830|Costa Rica|BOLD:AAA6775
Alphomelon Deans13|[698]|DHJPAR0030877|Costa Rica|BOLD:AAA6775
Alphomelon Deans13|[699]|DHJPAR0042917|Costa Rica|BOLD:AAA6775
Alphomelon Deans13|[700]|DHJPAR0002477|Costa Rica|BOLD:AAA6775
Alphomelon Deans13|[701]|DHJPAR0004884|Costa Rica|BOLD:AAA6775
Alphomelon Deans13|[702]|DHJPAR0020284|Costa Rica|BOLD:AAA6775
Alphomelon Deans13|[703]|DHJPAR0060183|Costa Rica|BOLD:AAA6775
Alphomelon Deans13|[704]|DHJPAR0060178|Costa Rica|BOLD:AAA6775
Alphomelon Deans13|[705]|DHJPAR0012874|Costa Rica|BOLD:AAA6775
Alphomelon Deans13|[706]|DHJPAR0063193|Costa Rica|BOLD:AAA6775
Alphomelon Deans13|[707]|DHJPAR0039875|Costa Rica|BOLD:AAA6775
Alphomelon Deans13|[708]|DHJPAR0002464|Costa Rica|BOLD:AAA6775
Alphomelon Deans13|[709]|DHJPAR0004642|Costa Rica|BOLD:AAA6775
Alphomelon Deans13|[710]|DHJPAR0025527|Costa Rica|BOLD:AAA6775
Alphomelon Deans13|[711]|DHJPAR0057761|Costa Rica|BOLD:AAA6775
Alphomelon Deans13|[712]|DHJPAR0012420|Costa Rica|BOLD:AAA6775
Alphomelon Deans13|[713]|DHJPAR0004912|Costa Rica|BOLD:AAA6775
Alphomelon Deans13|[714]|DHJPAR0002394|Costa Rica|BOLD:AAA6775
Alphomelon Deans13|[715]|DHJPAR0002475|Costa Rica|BOLD:AAA6775
Alphomelon Deans13|[716]|DHJPAR0002499|Costa Rica|BOLD:AAA6775
Alphomelon Deans13|[717]|DHJPAR0025376|Costa Rica|BOLD:AAA6775
Alphomelon Deans13|[718]|DHJPAR0062633|Costa Rica|BOLD:AAA6775
Alphomelon Deans13|[719]|DHJPAR0004970|Costa Rica|BOLD:AAA6775
Alphomelon Deans13|[720]|DHJPAR0005063|Costa Rica|BOLD:AAA6775
Alphomelon Deans13|[721]|DHJPAR0064063|Costa Rica|BOLD:AAA6775
Alphomelon Deans13|[722]|DHJPAR0002497|Costa Rica|BOLD:AAA6775
Alphomelon Deans13|[723]|DHJPAR0056740|Costa Rica|BOLD:AAA6775
Alphomelon Deans13|[724]|DHJPAR0050090|Costa Rica|BOLD:AAA6775
Alphomelon Deans13|[725]|DHJPAR0060610|Costa Rica|BOLD:AAA6775
Alphomelon Deans13|[726]|DHJPAR0011925|Costa Rica|BOLD:AAA6775
Alphomelon Deans13|[727]|DHJPAR0004887|Costa Rica|BOLD:AAA6775
Alphomelon Deans13|[728]|DHJPAR0004942|Costa Rica|BOLD:AAA6775
Alphomelon Deans13|[729]|DHJPAR0030704|Costa Rica|BOLD:AAA6775
Alphomelon Deans13|[730]|DHJPAR0030769|Costa Rica|BOLD:AAA6775
Alphomelon Deans13|[731]|DHJPAR0002393|Costa Rica|BOLD:AAA6775
Alphomelon Deans13|[732]|DHJPAR0025887|Costa Rica|BOLD:AAA6775
Alphomelon Deans13|[733]|DHJPAR0060682|Costa Rica|BOLD:AAA6775
Alphomelon nanosoma|[734]|DHJPAR0005058|Costa Rica
Alphomelon nanosoma|[735]|DHJPAR0004056|Costa Rica|BOLD:AAB9792
Alphomelon nanosoma|[736]|DHJPAR0005054|Costa Rica|BOLD:AAB9792
Alphomelon nanosoma|[737]|DHJPAR0030720|Costa Rica|BOLD:AAB9792
Alphomelon nanosoma|[738]|DHJPAR0053687|Costa Rica|BOLD:AAB9792
Alphomelon nanosoma|[739]|DHJPAR0059486|Costa Rica|BOLD:AAB9792
Alphomelon nanosoma|[740]|DHJPAR0051812|Costa Rica|BOLD:AAB9792
Alphomelon nanosoma|[741]|DHJPAR0060601|Costa Rica|BOLD:AAB9792
Alphomelon nanosoma|[742]|DHJPAR0060598|Costa Rica|BOLD:AAB9792
Alphomelon nanosoma|[743]|DHJPAR0038967|Costa Rica|BOLD:AAB9792
Alphomelon nanosoma|[744]|DHJPAR0049188|Costa Rica|BOLD:AAB9792
Alphomelon nanosoma|[745]|DHJPAR0059477|Costa Rica|BOLD:AAB9792
Alphomelon nanosoma|[746]|DHJPAR0054652|Costa Rica|BOLD:AAB9792
Alphomelon nanosoma|[747]|DHJPAR0005038|Costa Rica|BOLD:AAB9792
Alphomelon nanosoma|[748]|DHJPAR0054602|Costa Rica|BOLD:AAB9792
Alphomelon nanosoma|[749]|DHJPAR0054600|Costa Rica|BOLD:AAB9792
Alphomelon nanosoma|[750]|DHJPAR0034199|Costa Rica|BOLD:AAB9792
Alphomelon nanosoma|[751]|DHJPAR0058191|Costa Rica|BOLD:AAB9792
Alphomelon nanosoma|[752]|DHJPAR0058171|Costa Rica|BOLD:AAB9792
Alphomelon nanosoma|[753]|DHJPAR0061690|Costa Rica|BOLD:AAB9792
Alphomelon nanosoma|[754]|DHJPAR0035449|Costa Rica|BOLD:AAB9792
Alphomelon nanosoma|[755]|DHJPAR0035433|Costa Rica|BOLD:AAB9792
Alphomelon nanosoma|[756]|DHJPAR0020463|Costa Rica|BOLD:AAB9792
Alphomelon nanosoma|[757]|DHJPAR0020783|Costa Rica|BOLD:AAB9792
Alphomelon nanosoma|[758]|DHJPAR0012422|Costa Rica|BOLD:AAB9792
Alphomelon nanosoma|[759]|DHJPAR0012410|Costa Rica|BOLD:AAB9792
Alphomelon nanosoma|[760]|DHJPAR0004910|Costa Rica|BOLD:AAB9792
Alphomelon nanosoma|[761]|DHJPAR0058960|Costa Rica|BOLD:AAB9792
Alphomelon nanosoma|[762]|DHJPAR0060221|Costa Rica|BOLD:AAB9792
Alphomelon nanosoma|[763]|DHJPAR0060214|Costa Rica|BOLD:AAB9792
Alphomelon nanosoma|[764]|DHJPAR0058933|Costa Rica|BOLD:AAB9792
Alphomelon nanosoma|[765]|DHJPAR0035428|Costa Rica|BOLD:AAB9792

Alphomelon nanosoma|[763]|DHJPAR0060214|Costa Rica|BOLD:AAB9792
Alphomelon nanosoma|[764]|DHJPAR0058933|Costa Rica|BOLD:AAB9792
Alphomelon nanosoma|[765]|DHJPAR0035428|Costa Rica|BOLD:AAB9792
Alphomelon nanosoma|[766]|DHJPAR0060187|Costa Rica|BOLD:AAB9792
Alphomelon nanosoma|[767]|DHJPAR0058890|Costa Rica|BOLD:AAB9792
Alphomelon nanosoma|[768]|DHJPAR0047044|Costa Rica|BOLD:AAB9792
Alphomelon nanosoma|[769]|DHJPAR0058875|Costa Rica|BOLD:AAB9792
Alphomelon nanosoma|[770]|DHJPAR0060149|Costa Rica|BOLD:AAB9792
Alphomelon nanosoma|[771]|DHJPAR0058870|Costa Rica|BOLD:AAB9792
Alphomelon nanosoma|[772]|DHJPAR0060134|Costa Rica|BOLD:AAB9792
Alphomelon nanosoma|[773]|DHJPAR0058850|Costa Rica|BOLD:AAB9792
Alphomelon nanosoma|[774]|DHJPAR0012861|Costa Rica|BOLD:AAB9792
Alphomelon nanosoma|[775]|DHJPAR0060679|Costa Rica|BOLD:AAB9792
Alphomelon Deans31|[776]|DHJPAR0051293|Costa Rica|BOLD:ACB1223
Alphomelon Deans31|[777]|DHJPAR0049074|Costa Rica|BOLD:ACB1223
Alphomelon Deans32|[778]|DHJPAR0051292|Costa Rica|BOLD:ACJ4259
Alphomelon Deans15|[779]|DHJPAR0005028|Costa Rica|BOLD:AAJ2171
Alphomelon Deans15|[780]|DHJPAR0012125|Costa Rica|BOLD:AAJ2171
Alphomelon talidicida|[781]|DHJPAR0030709|Costa Rica|BOLD:AAA7259
Alphomelon talidicida|[782]|DHJPAR0048144|Costa Rica|BOLD:AAA7259
Alphomelon talidicida|[783]|DHJPAR0030698|Costa Rica|BOLD:AAA7259
Alphomelon talidicida|[784]|DHJPAR0056390|Costa Rica|BOLD:AAA7259
Alphomelon talidicida|[785]|DHJPAR0047253|Costa Rica|BOLD:AAA7259
Alphomelon talidicida|[786]|DHJPAR0002454|Costa Rica
Alphomelon talidicida|[787]|DHJPAR0004973|Costa Rica|BOLD:AAA7259
Alphomelon talidicida|[788]|DHJPAR0002453|Costa Rica|BOLD:AAA7259
Alphomelon talidicida|[789]|DHJPAR0060201|Costa Rica|BOLD:AAA7259
Alphomelon talidicida|[790]|DHJPAR0020287|Costa Rica|BOLD:AAA7259
Alphomelon talidicida|[791]|DHJPAR0012408|Costa Rica|BOLD:AAA7259
Alphomelon talidicida|[792]|DHJPAR0004886|Costa Rica|BOLD:AAA7259
Alphomelon talidicida|[793]|DHJPAR0063407|Costa Rica|BOLD:AAA7259
Alphomelon talidicida|[794]|DHJPAR0004655|Costa Rica|BOLD:AAA7259
Alphomelon talidicida|[795]|DHJPAR0050959|Costa Rica|BOLD:AAA7259
Alphomelon talidicida|[796]|DHJPAR0053777|Costa Rica|BOLD:AAA7259
Alphomelon talidicida|[797]|DHJPAR0004925|Costa Rica|BOLD:AAA7259
Alphomelon talidicida|[798]|DHJPAR0020282|Costa Rica|BOLD:AAA7259
Alphomelon talidicida|[799]|DHJPAR0065240|Costa Rica|BOLD:AAA7259
Alphomelon talidicida|[800]|DHJPAR0002435|Costa Rica|BOLD:AAA7259
Alphomelon talidicida|[801]|DHJPAR0011854|Costa Rica|BOLD:AAA7259
Alphomelon talidicida|[802]|DHJPAR0011843|Costa Rica|BOLD:AAA7259
Alphomelon talidicida|[803]|DHJPAR0012856|Costa Rica|BOLD:AAA7259
Alphomelon talidicida|[804]|DHJPAR0060683|Costa Rica|BOLD:AAA7259
Alphomelon talidicida|[805]|DHJPAR0056698|Costa Rica|BOLD:AAA7259
Alphomelon talidicida|[806]|DHJPAR0002927|Costa Rica|BOLD:AAA7259
Alphomelon talidicida|[807]|DHJPAR0004962|Costa Rica|BOLD:AAA7259
Alphomelon talidicida|[808]|DHJPAR0038166|Costa Rica|BOLD:AAA7259
Alphomelon talidicida|[809]|DHJPAR0061723|Costa Rica|BOLD:AAA7259
Alphomelon talidicida|[810]|DHJPAR0060138|Costa Rica|BOLD:AAA7259
Alphomelon talidicida|[811]|DHJPAR0058892|Costa Rica|BOLD:AAA7259
Alphomelon talidicida|[812]|DHJPAR0047138|Costa Rica|BOLD:AAA7259
Alphomelon talidicida|[813]|DHJPAR0056694|Costa Rica|BOLD:AAA7259
Alphomelon talidicida|[814]|DHJPAR0004915|Costa Rica|BOLD:AAA7259
Alphomelon talidicida|[815]|DHJPAR0047254|Costa Rica|BOLD:AAA7259
Alphomelon talidicida|[816]|DHJPAR0047259|Costa Rica|BOLD:AAA7259
Alphomelon talidicida|[817]|DHJPAR0004666|Costa Rica|BOLD:AAA7259
Alphomelon talidicida|[818]|DHJPAR0004667|Costa Rica|BOLD:AAA7259
Alphomelon talidicida|[819]|DHJPAR0002442|Costa Rica|BOLD:AAA7259
Alphomelon talidicida|[820]|DHJPAR0002450|Costa Rica|BOLD:AAA7259
Alphomelon talidicida|[821]|DHJPAR0061684|Costa Rica|BOLD:AAA7259
Alphomelon talidicida|[822]|DHJPAR0061685|Costa Rica|BOLD:AAA7259
Alphomelon talidicida|[823]|DHJPAR0050979|Costa Rica|BOLD:AAA7259
Alphomelon talidicida|[824]|DHJPAR0056697|Costa Rica|BOLD:AAA7259
Alphomelon talidicida|[825]|DHJPAR0058206|Costa Rica|BOLD:AAA7259
Alphomelon talidicida|[826]|DHJPAR0061620|Costa Rica|BOLD:AAA7259
Alphomelon talidicida|[827]|DHJPAR0058224|Costa Rica|BOLD:AAA7259
Alphomelon talidicida|[828]|DHJPAR0061790|Costa Rica|BOLD:AAA7259
Alphomelon talidicida|[829]|DHJPAR0058864|Costa Rica|BOLD:AAA7259
Alphomelon talidicida|[830]|DHJPAR0041819|Costa Rica|BOLD:AAA7259
Alphomelon talidicida|[831]|DHJPAR0004941|Costa Rica|BOLD:AAA7259
Alphomelon talidicida|[832]|DHJPAR0004961|Costa Rica|BOLD:AAA7259
Alphomelon talidicida|[833]|DHJPAR0004967|Costa Rica|BOLD:AAA7259
Alphomelon talidicida|[834]|DHJPAR0062733|Costa Rica|BOLD:AAA7259
Alphomelon talidicida|[835]|DHJPAR0005046|Costa Rica|BOLD:AAA7259
Alphomelon talidicida|[836]|DHJPAR0005062|Costa Rica|BOLD:AAA7259
Alphomelon talidicida|[837]|DHJPAR0058910|Costa Rica|BOLD:AAA7259
Alphomelon talidicida|[838]|DHJPAR0054700|Costa Rica|BOLD:AAA7259
Alphomelon talidicida|[839]|DHJPAR0056704|Costa Rica|BOLD:AAA7259
Alphomelon talidicida|[840]|DHJPAR0056551|Costa Rica|BOLD:AAA7259
Alphomelon talidicida|[841]|DHJPAR0056780|Costa Rica|BOLD:AAA7259
Alphomelon talidicida|[842]|DHJPAR0056730|Costa Rica|BOLD:AAA7259
Alphomelon talidicida|[843]|DHJPAR0051199|Costa Rica|BOLD:AAA7259
Alphomelon talidicida|[844]|DHJPAR0051203|Costa Rica|BOLD:AAA7259
Alphomelon talidicida|[845]|DHJPAR0047139|Costa Rica|BOLD:AAA7259
Alphomelon talidicida|[846]|DHJPAR0049216|Costa Rica|BOLD:AAA7259
Alphomelon talidicida|[847]|DHJPAR0004932|Costa Rica|BOLD:AAA7259
Alphomelon talidicida|[848]|DHJPAR0011857|Costa Rica|BOLD:AAA7259
Alphomelon talidicida|[849]|DHJPAR0062109|Costa Rica|BOLD:AAA7259
Alphomelon talidicida|[850]|DHJPAR0047255|Costa Rica|BOLD:AAA7259
Alphomelon talidicida|[851]|DHJPAR0060131|Costa Rica|BOLD:AAA7259
Alphomelon talidicida|[852]|DHJPAR0056679|Costa Rica|BOLD:AAA7259
Alphomelon talidicida|[853]|DHJPAR0065190|Costa Rica|BOLD:AAA7259
Alphomelon talidicida|[854]|DHJPAR0055247|Costa Rica|BOLD:AAA7259
Alphomelon talidicida|[855]|DHJPAR0060687|Costa Rica|BOLD:AAA7259
Alphomelon talidicida|[856]|DHJPAR0060676|Costa Rica|BOLD:AAA7259
Alphomelon talidicida|[857]|DHJPAR0012875|Costa Rica|BOLD:AAA7259
Alphomelon talidicida|[858]|DHJPAR0039873|Costa Rica|BOLD:AAA7259
Alphomelon talidicida|[859]|DHJPAR0058876|Costa Rica|BOLD:AAA7259
Alphomelon talidicida|[860]|DHJPAR0039876|Costa Rica|BOLD:AAA7259
[illegible]

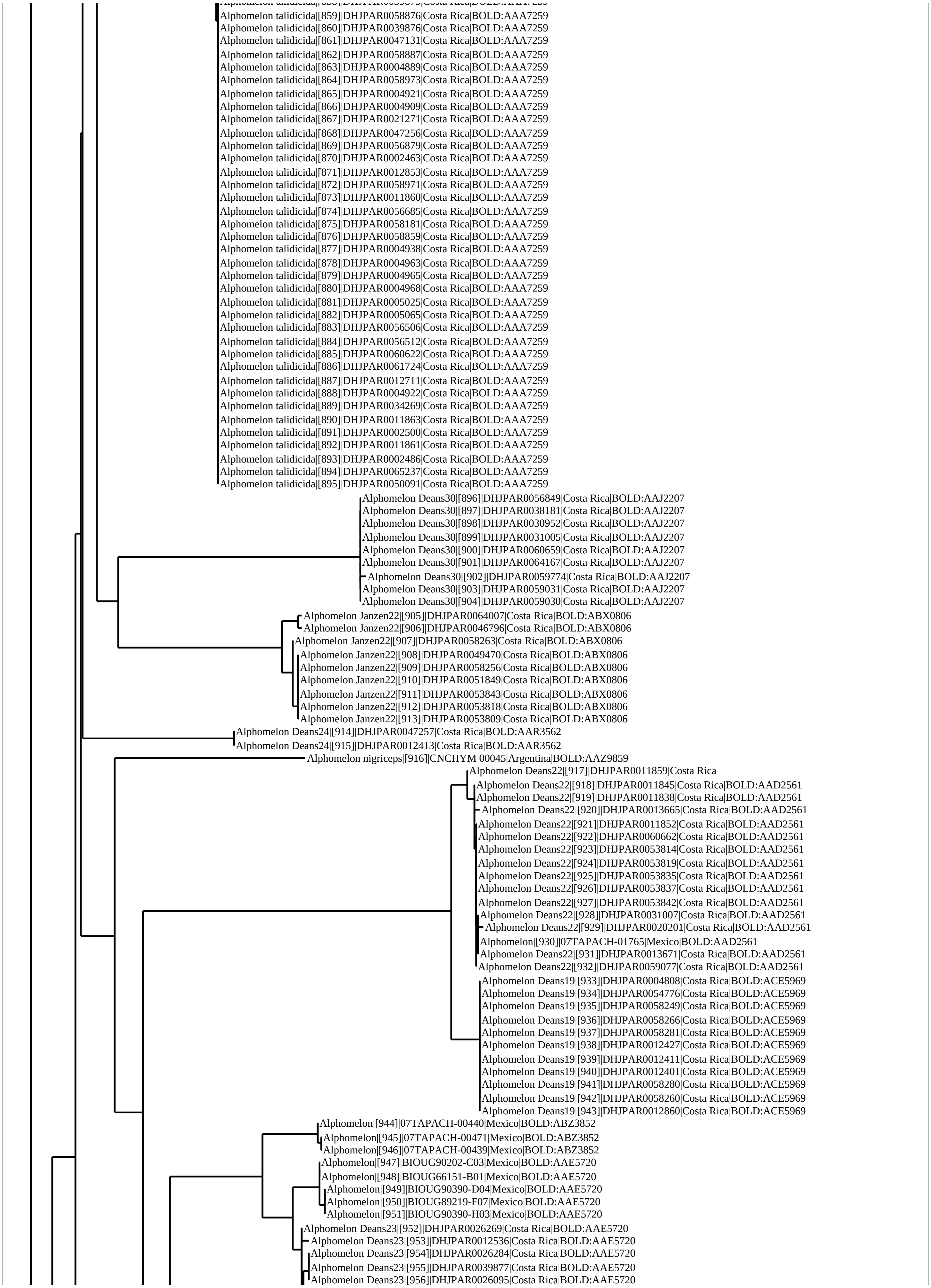
Alphomelon talidicida|[859]|DHJPAR0058876|Costa Rica|BOLD:AAA7259
Alphomelon talidicida|[860]|DHJPAR0039876|Costa Rica|BOLD:AAA7259
Alphomelon talidicida|[861]|DHJPAR0047131|Costa Rica|BOLD:AAA7259
Alphomelon talidicida|[862]|DHJPAR0058887|Costa Rica|BOLD:AAA7259
Alphomelon talidicida|[863]|DHJPAR0004889|Costa Rica|BOLD:AAA7259
Alphomelon talidicida|[864]|DHJPAR0058973|Costa Rica|BOLD:AAA7259
Alphomelon talidicida|[865]|DHJPAR0004921|Costa Rica|BOLD:AAA7259
Alphomelon talidicida|[866]|DHJPAR0004909|Costa Rica|BOLD:AAA7259
Alphomelon talidicida|[867]|DHJPAR0021271|Costa Rica|BOLD:AAA7259
Alphomelon talidicida|[868]|DHJPAR0047256|Costa Rica|BOLD:AAA7259
Alphomelon talidicida|[869]|DHJPAR0056879|Costa Rica|BOLD:AAA7259
Alphomelon talidicida|[870]|DHJPAR0002463|Costa Rica|BOLD:AAA7259
Alphomelon talidicida|[871]|DHJPAR0012853|Costa Rica|BOLD:AAA7259
Alphomelon talidicida|[872]|DHJPAR0058971|Costa Rica|BOLD:AAA7259
Alphomelon talidicida|[873]|DHJPAR0011860|Costa Rica|BOLD:AAA7259
Alphomelon talidicida|[874]|DHJPAR0056685|Costa Rica|BOLD:AAA7259
Alphomelon talidicida|[875]|DHJPAR0058181|Costa Rica|BOLD:AAA7259
Alphomelon talidicida|[876]|DHJPAR0058859|Costa Rica|BOLD:AAA7259
Alphomelon talidicida|[877]|DHJPAR0004938|Costa Rica|BOLD:AAA7259
Alphomelon talidicida|[878]|DHJPAR0004963|Costa Rica|BOLD:AAA7259
Alphomelon talidicida|[879]|DHJPAR0004965|Costa Rica|BOLD:AAA7259
Alphomelon talidicida|[880]|DHJPAR0004968|Costa Rica|BOLD:AAA7259
Alphomelon talidicida|[881]|DHJPAR0005025|Costa Rica|BOLD:AAA7259
Alphomelon talidicida|[882]|DHJPAR0005065|Costa Rica|BOLD:AAA7259
Alphomelon talidicida|[883]|DHJPAR0056506|Costa Rica|BOLD:AAA7259
Alphomelon talidicida|[884]|DHJPAR0056512|Costa Rica|BOLD:AAA7259
Alphomelon talidicida|[885]|DHJPAR0060622|Costa Rica|BOLD:AAA7259
Alphomelon talidicida|[886]|DHJPAR0061724|Costa Rica|BOLD:AAA7259
Alphomelon talidicida|[887]|DHJPAR0012711|Costa Rica|BOLD:AAA7259
Alphomelon talidicida|[888]|DHJPAR0004922|Costa Rica|BOLD:AAA7259
Alphomelon talidicida|[889]|DHJPAR0034269|Costa Rica|BOLD:AAA7259
Alphomelon talidicida|[890]|DHJPAR0011863|Costa Rica|BOLD:AAA7259
Alphomelon talidicida|[891]|DHJPAR0002500|Costa Rica|BOLD:AAA7259
Alphomelon talidicida|[892]|DHJPAR0011861|Costa Rica|BOLD:AAA7259
Alphomelon talidicida|[893]|DHJPAR0002486|Costa Rica|BOLD:AAA7259
Alphomelon talidicida|[894]|DHJPAR0065237|Costa Rica|BOLD:AAA7259
Alphomelon talidicida|[895]|DHJPAR0050091|Costa Rica|BOLD:AAA7259
Alphomelon Deans30|[896]|DHJPAR0056849|Costa Rica|BOLD:AAJ2207
Alphomelon Deans30|[897]|DHJPAR0038181|Costa Rica|BOLD:AAJ2207
Alphomelon Deans30|[898]|DHJPAR0030952|Costa Rica|BOLD:AAJ2207
Alphomelon Deans30|[899]|DHJPAR0031005|Costa Rica|BOLD:AAJ2207
Alphomelon Deans30|[900]|DHJPAR0060659|Costa Rica|BOLD:AAJ2207
Alphomelon Deans30|[901]|DHJPAR0064167|Costa Rica|BOLD:AAJ2207
Alphomelon Deans30|[902]|DHJPAR0059774|Costa Rica|BOLD:AAJ2207
Alphomelon Deans30|[903]|DHJPAR0059031|Costa Rica|BOLD:AAJ2207
Alphomelon Deans30|[904]|DHJPAR0059030|Costa Rica|BOLD:AAJ2207
Alphomelon Janzen22|[905]|DHJPAR0064007|Costa Rica|BOLD:ABX0806
Alphomelon Janzen22|[906]|DHJPAR0046796|Costa Rica|BOLD:ABX0806
Alphomelon Janzen22|[907]|DHJPAR0058263|Costa Rica|BOLD:ABX0806
Alphomelon Janzen22|[908]|DHJPAR0049470|Costa Rica|BOLD:ABX0806
Alphomelon Janzen22|[909]|DHJPAR0058256|Costa Rica|BOLD:ABX0806
Alphomelon Janzen22|[910]|DHJPAR0051849|Costa Rica|BOLD:ABX0806
Alphomelon Janzen22|[911]|DHJPAR0053843|Costa Rica|BOLD:ABX0806
Alphomelon Janzen22|[912]|DHJPAR0053818|Costa Rica|BOLD:ABX0806
Alphomelon Janzen22|[913]|DHJPAR0053809|Costa Rica|BOLD:ABX0806
Alphomelon Deans24|[914]|DHJPAR0047257|Costa Rica|BOLD:AAR3562
Alphomelon Deans24|[915]|DHJPAR0012413|Costa Rica|BOLD:AAR3562
Alphomelon nigriceps|[916]|CNCHYM 00045|Argentina|BOLD:AAZ9859
Alphomelon Deans22|[917]|DHJPAR0011859|Costa Rica
Alphomelon Deans22|[918]|DHJPAR0011845|Costa Rica|BOLD:AAD2561
Alphomelon Deans22|[919]|DHJPAR0011838|Costa Rica|BOLD:AAD2561
Alphomelon Deans22|[920]|DHJPAR0013665|Costa Rica|BOLD:AAD2561
Alphomelon Deans22|[921]|DHJPAR0011852|Costa Rica|BOLD:AAD2561
Alphomelon Deans22|[922]|DHJPAR0060662|Costa Rica|BOLD:AAD2561
Alphomelon Deans22|[923]|DHJPAR0053814|Costa Rica|BOLD:AAD2561
Alphomelon Deans22|[924]|DHJPAR0053819|Costa Rica|BOLD:AAD2561
Alphomelon Deans22|[925]|DHJPAR0053835|Costa Rica|BOLD:AAD2561
Alphomelon Deans22|[926]|DHJPAR0053837|Costa Rica|BOLD:AAD2561
Alphomelon Deans22|[927]|DHJPAR0053842|Costa Rica|BOLD:AAD2561
Alphomelon Deans22|[928]|DHJPAR0031007|Costa Rica|BOLD:AAD2561
Alphomelon Deans22|[929]|DHJPAR0020201|Costa Rica|BOLD:AAD2561
Alphomelon|[930]|07TAPACH-01765|Mexico|BOLD:AAD2561
Alphomelon Deans22|[931]|DHJPAR0013671|Costa Rica|BOLD:AAD2561
Alphomelon Deans22|[932]|DHJPAR0059077|Costa Rica|BOLD:AAD2561
Alphomelon Deans19|[933]|DHJPAR0004808|Costa Rica|BOLD:ACE5969
Alphomelon Deans19|[934]|DHJPAR0054776|Costa Rica|BOLD:ACE5969
Alphomelon Deans19|[935]|DHJPAR0058249|Costa Rica|BOLD:ACE5969
Alphomelon Deans19|[936]|DHJPAR0058266|Costa Rica|BOLD:ACE5969
Alphomelon Deans19|[937]|DHJPAR0058281|Costa Rica|BOLD:ACE5969
Alphomelon Deans19|[938]|DHJPAR0012427|Costa Rica|BOLD:ACE5969
Alphomelon Deans19|[939]|DHJPAR0012411|Costa Rica|BOLD:ACE5969
Alphomelon Deans19|[940]|DHJPAR0012401|Costa Rica|BOLD:ACE5969
Alphomelon Deans19|[941]|DHJPAR0058280|Costa Rica|BOLD:ACE5969
Alphomelon Deans19|[942]|DHJPAR0058260|Costa Rica|BOLD:ACE5969
Alphomelon Deans19|[943]|DHJPAR0012860|Costa Rica|BOLD:ACE5969
Alphomelon|[944]|07TAPACH-00440|Mexico|BOLD:ABZ3852
Alphomelon|[945]|07TAPACH-00471|Mexico|BOLD:ABZ3852
Alphomelon|[946]|07TAPACH-00439|Mexico|BOLD:ABZ3852
Alphomelon|[947]|BIOUG90202-C03|Mexico|BOLD:AAE5720
Alphomelon|[948]|BIOUG66151-B01|Mexico|BOLD:AAE5720
Alphomelon|[949]|BIOUG90390-D04|Mexico|BOLD:AAE5720
Alphomelon|[950]|BIOUG89219-F07|Mexico|BOLD:AAE5720
Alphomelon|[951]|BIOUG90390-H03|Mexico|BOLD:AAE5720
Alphomelon Deans23|[952]|DHJPAR0026269|Costa Rica|BOLD:AAE5720
Alphomelon Deans23|[953]|DHJPAR0012536|Costa Rica|BOLD:AAE5720
Alphomelon Deans23|[954]|DHJPAR0026284|Costa Rica|BOLD:AAE5720
Alphomelon Deans23|[955]|DHJPAR0039877|Costa Rica|BOLD:AAE5720
Alphomelon Deans23|[956]|DHJPAR0026095|Costa Rica|BOLD:AAE5720

Alphomelon Deans23|[954]|DHJPAR0026284|Costa Rica|BOLD:AAE5720
Alphomelon Deans23|[955]|DHJPAR0039877|Costa Rica|BOLD:AAE5720
Alphomelon Deans23|[956]|DHJPAR0026095|Costa Rica|BOLD:AAE5720
Alphomelon|[957]|07TAPACH-01674|Mexico|BOLD:AAE5720
Alphomelon Deans23|[958]|DHJPAR0057768|Costa Rica|BOLD:AAE5720
Alphomelon Deans23|[959]|DHJPAR0059057|Costa Rica|BOLD:AAE5720
Alphomelon disputabile|[960]|BIOUG62552-D06|Costa Rica|BOLD:AAF3301
Alphomelon disputabile|[961]|DHJPAR0061875|Costa Rica|BOLD:AAF3301
Alphomelon disputabile|[962]|DHJPAR0004956|Costa Rica|BOLD:AAF3301
Alphomelon disputabile|[963]|DHJPAR0004960|Costa Rica|BOLD:AAF3301
Alphomelon disputabile|[964]|DHJPAR0013664|Costa Rica|BOLD:AAF3301
Alphomelon disputabile|[965]|DHJPAR0031614|Costa Rica|BOLD:AAF3301
Alphomelon disputabile|[966]|DHJPAR0058253|Costa Rica|BOLD:AAF3301
Alphomelon disputabile|[967]|DHJPAR0061861|Costa Rica|BOLD:AAF3301
Alphomelon disputabile|[968]|DHJPAR0059067|Costa Rica|BOLD:AAF3301
Alphomelon Deans18DHJ01|[969]|DHJPAR0004811|Costa Rica|BOLD:AAF3318
Alphomelon Deans18DHJ01|[970]|DHJPAR0004810|Costa Rica
Alphomelon Deans18DHJ02|[971]|DHJPAR0034186|Costa Rica|BOLD:ADA5721
Alphomelon|[972]|BIOUG26457-C04|Mexico|BOLD:ADA5721
Alphomelon Deans18DHJ03|[973]|DHJPAR0058241|Costa Rica|BOLD:ADA7564
Alphomelon Deans18DHJ03|[974]|DHJPAR0058242|Costa Rica|BOLD:ADA7564
Alphomelon Deans18DHJ03|[975]|DHJPAR0058246|Costa Rica|BOLD:ADA7564
Alphomelon Deans18DHJ03|[976]|DHJPAR0058250|Costa Rica|BOLD:ADA7564
Alphomelon Deans18DHJ03|[977]|DHJPAR0058239|Costa Rica|BOLD:ADA7564
Alphomelon Deans18DHJ03|[978]|DHJPAR0058283|Costa Rica|BOLD:ADA7564
Alphomelon Deans18DHJ03|[979]|DHJPAR0058255|Costa Rica|BOLD:ADA7564
Alphomelon Deans18DHJ03|[980]|DHJPAR0059026|Costa Rica|BOLD:ADA7564
Alphomelon Deans18DHJ03|[981]|DHJPAR0059045|Costa Rica|BOLD:ADA7564
Alphomelon Deans18DHJ03|[982]|DHJPAR0058248|Costa Rica|BOLD:ADA7564
Alphomelon Deans18DHJ03|[983]|DHJPAR0058245|Costa Rica|BOLD:ADA7564
Alphomelon Deans18DHJ03|[984]|DHJPAR0058264|Costa Rica|BOLD:ADA7564
Alphomelon Deans18DHJ03|[985]|DHJPAR0060271|Costa Rica|BOLD:ADA7564
Alphomelon Deans18DHJ03|[986]|DHJPAR0048219|Costa Rica|BOLD:ADA7564
Alphomelon Deans18DHJ03|[987]|DHJPAR0058251|Costa Rica|BOLD:ADA7564
Alphomelon Deans18DHJ03|[988]|DHJPAR0058270|Costa Rica|BOLD:ADA7564
Alphomelon Deans18DHJ03|[989]|DHJPAR0058254|Costa Rica|BOLD:ADA7564
Alphomelon Deans18DHJ03|[990]|DHJPAR0062082|Costa Rica|BOLD:ADA7564
Alphomelon Deans04|[991]|DHJPAR0034215|Costa Rica|BOLD:AAJ2135
Alphomelon Deans04|[992]|DHJPAR0064610|Costa Rica|BOLD:AAJ2135
Alphomelon Deans04|[993]|DHJPAR0041859|Costa Rica|BOLD:AAJ2135
Alphomelon Deans04|[994]|DHJPAR0002397|Costa Rica|BOLD:AAJ2135
Alphomelon Deans05|[995]|DHJPAR0002456|Costa Rica|BOLD:AAC7653
Alphomelon Deans05|[996]|DHJPAR0011851|Costa Rica|BOLD:AAC7653
Alphomelon Deans05|[997]|DHJPAR0002434|Costa Rica|BOLD:AAC7653
Alphomelon Deans05|[998]|DHJPAR0002462|Costa Rica|BOLD:AAC7653
Alphomelon Deans05|[999]|DHJPAR0004670|Costa Rica|BOLD:AAC7653
Alphomelon Deans05|[1000]|DHJPAR0004668|Costa Rica|BOLD:AAC7653
Alphomelon Deans05|[1001]|DHJPAR0012417|Costa Rica|BOLD:AAC7653
Alphomelon Deans05|[1002]|DHJPAR0005021|Costa Rica|BOLD:AAC7653
Alphomelon Deans05|[1003]|DHJPAR0031004|Costa Rica|BOLD:AAC7653
Alphomelon Deans05|[1004]|DHJPAR0012121|Costa Rica|BOLD:AAC7653
Alphomelon citroloma|[1005]|BIOUG92282-B07|Costa Rica|BOLD:AEN2538
Hymenoptera|[1006]|BIOUG92355-E04|Costa Rica
Alphomelon citroloma|[1007]|BIOUG94211-E06|Costa Rica|BOLD:AEN2538
Alphomelon citroloma|[1008]|BIOUG92281-F02|Costa Rica|BOLD:AEN2538
Hymenoptera|[1009]|BIOUG96059-B09|Costa Rica|BOLD:AEN2538
Hymenoptera|[1010]|BIOUG92367-F10|Costa Rica|BOLD:AEN2538
Alphomelon citroloma|[1011]|BIOUG95212-F10|Costa Rica|BOLD:AEN2538
Alphomelon Deans29|[1012]|DHJPAR0039480|Costa Rica|BOLD:AAB8584
Alphomelon Deans29|[1013]|DHJPAR0027684|Costa Rica|BOLD:AAB8584
Alphomelon Deans29|[1014]|DHJPAR0060266|Costa Rica|BOLD:AAB8584
Alphomelon Deans29|[1015]|DHJPAR0002459|Costa Rica|BOLD:AAB8584
Alphomelon Deans29|[1016]|DHJPAR0027666|Costa Rica|BOLD:AAB8584
Alphomelon Deans29|[1017]|DHJPAR0013793|Costa Rica|BOLD:AAB8584
Alphomelon Deans29|[1018]|DHJPAR0013794|Costa Rica|BOLD:AAB8584
Alphomelon Deans29|[1019]|DHJPAR0042479|Costa Rica|BOLD:AAB8584
Alphomelon Deans29|[1020]|DHJPAR0042047|Costa Rica|BOLD:AAB8584
Alphomelon Deans29|[1021]|DHJPAR0043086|Costa Rica|BOLD:AAB8584
Alphomelon Deans29|[1022]|DHJPAR0042480|Costa Rica|BOLD:AAB8584
Alphomelon Deans29|[1023]|DHJPAR0059051|Costa Rica|BOLD:AAB8584
Alphomelon melanoscelis|[1024]|WMIC 0349|Venezuela|BOLD:AAB8584
Alphomelon Deans29|[1025]|DHJPAR0026277|Costa Rica|BOLD:AAB8584
Alphomelon Deans29|[1026]|BIOUG17714-A08|Costa Rica|BOLD:AAB8584
Alphomelon Deans29|[1027]|DHJPAR0004234|Costa Rica|BOLD:AAB8584
Alphomelon Deans29|[1028]|DHJPAR0049083|Costa Rica|BOLD:AAB8584
Alphomelon Deans29|[1029]|DHJPAR0049027|Costa Rica|BOLD:AAB8584
Alphomelon Deans29|[1030]|DHJPAR0049933|Costa Rica|BOLD:AAB8584
Alphomelon Deans29|[1031]|DHJPAR0002479|Costa Rica|BOLD:AAB8584
Alphomelon Deans29|[1032]|DHJPAR0002468|Costa Rica|BOLD:AAB8584
Alphomelon Deans29|[1033]|DHJPAR0004803|Costa Rica|BOLD:AAB8584
Alphomelon Deans29|[1034]|DHJPAR0049033|Costa Rica|BOLD:AAB8584
Alphomelon melanoscelis|[1035]|07TAPACH-01773|Mexico|BOLD:AAB8584
Alphomelon Deans29|[1036]|DHJPAR0004806|Costa Rica|BOLD:AAB8584
Alphomelon Deans29|[1037]|DHJPAR0002472|Costa Rica|BOLD:AAB8584
Alphomelon Deans29|[1038]|DHJPAR0005040|Costa Rica|BOLD:AAB8584
Alphomelon Deans29|[1039]|DHJPAR0049249|Costa Rica|BOLD:AAB8584
Alphomelon Deans29|[1040]|DHJPAR0002480|Costa Rica|BOLD:AAB8584
Alphomelon Deans29|[1041]|DHJPAR0012871|Costa Rica|BOLD:AAB8584
Alphomelon Deans29|[1042]|DHJPAR0012878|Costa Rica|BOLD:AAB8584
Alphomelon Deans29|[1043]|DHJPAR0047176|Costa Rica|BOLD:AAB8584
Alphomelon Deans29|[1044]|DHJPAR0026274|Costa Rica|BOLD:AAB8584
Alphomelon Deans29|[1045]|DHJPAR0058276|Costa Rica|BOLD:AAB8584
Alphomelon Deans29|[1046]|DHJPAR0058243|Costa Rica|BOLD:AAB8584
Alphomelon Deans29|[1047]|DHJPAR0056870|Costa Rica|BOLD:AAB8584
Alphomelon melanoscelis|[1048]|CNCHYM 00037|Honduras|BOLD:AAB8584
Alphomelon Deans29|[1049]|DHJPAR0042413|Costa Rica|BOLD:AAB8584
Alphomelon Deans29|[1050]|DHJPAR0041966|Costa Rica|BOLD:AAB8584
Alphomelon Deans29|[1051]|DHJPAR0059053|Costa Rica|BOLD:AAB8584
Alphomelon Deans29|[1052]|DHJPAR0045334|Costa Rica|BOLD:AAB8584

Alphomelon Deans29|[1050]|DHJPAR0041966|Costa Rica|BOLD:AAB8584
Alphomelon Deans29|[1051]|DHJPAR0059053|Costa Rica|BOLD:AAB8584
Alphomelon Deans29|[1052]|DHJPAR0045334|Costa Rica|BOLD:AAB8584
Alphomelon Deans29|[1053]|DHJPAR0057803|Costa Rica|BOLD:AAB8584
Alphomelon Deans29|[1054]|DHJPAR0059033|Costa Rica|BOLD:AAB8584
Alphomelon Deans29|[1055]|DHJPAR0059029|Costa Rica|BOLD:AAB8584
Alphomelon Deans29|[1056]|DHJPAR0059023|Costa Rica|BOLD:AAB8584
Alphomelon Deans29|[1057]|DHJPAR0060263|Costa Rica|BOLD:AAB8584
Alphomelon Deans29|[1058]|DHJPAR0058839|Costa Rica|BOLD:AAB8584
Alphomelon Deans29|[1059]|DHJPAR0057532|Costa Rica|BOLD:AAB8584
Alphomelon Deans29|[1060]|DHJPAR0059061|Costa Rica|BOLD:AAB8584
Alphomelon Deans29|[1061]|DHJPAR0059068|Costa Rica|BOLD:AAB8584
Alphomelon Deans29|[1062]|DHJPAR0059041|Costa Rica|BOLD:AAB8584
Alphomelon Deans29|[1063]|DHJPAR0059028|Costa Rica|BOLD:AAB8584
Alphomelon Deans29|[1064]|DHJPAR0058994|Costa Rica|BOLD:AAB8584
Alphomelon Deans29|[1065]|DHJPAR0042455|Costa Rica|BOLD:AAB8584
Alphomelon Deans29|[1066]|DHJPAR0042542|Costa Rica|BOLD:AAB8584
Alphomelon Deans29|[1067]|DHJPAR0041851|Costa Rica|BOLD:AAB8584
Alphomelon Deans29|[1068]|DHJPAR0056871|Costa Rica|BOLD:AAB8584
Alphomelon Deans29|[1069]|DHJPAR0042485|Costa Rica|BOLD:AAB8584
Alphomelon Deans29|[1070]|DHJPAR0047235|Costa Rica|BOLD:AAB8584
Alphomelon Deans29|[1071]|DHJPAR0060260|Costa Rica|BOLD:AAB8584
Alphomelon Deans29|[1072]|DHJPAR0042453|Costa Rica|BOLD:AAB8584
Alphomelon Deans29|[1073]|DHJPAR0049464|Costa Rica|BOLD:AAB8584
Alphomelon Deans29|[1074]|DHJPAR0056030|Costa Rica|BOLD:AAB8584
Alphomelon Deans29|[1075]|DHJPAR0042881|Costa Rica|BOLD:AAB8584
Alphomelon Deans29|[1076]|DHJPAR0059776|Costa Rica|BOLD:AAB8584
Alphomelon Deans29|[1077]|DHJPAR0041858|Costa Rica|BOLD:AAB8584
Alphomelon Deans29|[1078]|DHJPAR0043004|Costa Rica|BOLD:AAB8584
Alphomelon Deans29|[1079]|DHJPAR0043009|Costa Rica|BOLD:AAB8584
Alphomelon Deans29|[1080]|DHJPAR0042052|Costa Rica|BOLD:AAB8584
Alphomelon Deans29|[1081]|DHJPAR0042471|Costa Rica|BOLD:AAB8584
Alphomelon Deans29|[1082]|DHJPAR0049465|Costa Rica|BOLD:AAB8584
Alphomelon Deans29|[1083]|DHJPAR0043076|Costa Rica|BOLD:AAB8584
Alphomelon Deans29|[1084]|DHJPAR0043077|Costa Rica|BOLD:AAB8584
Alphomelon Deans29|[1085]|DHJPAR0043092|Costa Rica|BOLD:AAB8584
Alphomelon Deans29|[1086]|DHJPAR0043094|Costa Rica|BOLD:AAB8584
Alphomelon Deans29|[1087]|DHJPAR0043110|Costa Rica|BOLD:AAB8584
Alphomelon Deans29|[1088]|DHJPAR0043122|Costa Rica|BOLD:AAB8584
Alphomelon Deans29|[1089]|DHJPAR0043127|Costa Rica|BOLD:AAB8584
Alphomelon Deans29|[1090]|DHJPAR0043130|Costa Rica|BOLD:AAB8584
Alphomelon Deans29|[1091]|DHJPAR0042481|Costa Rica|BOLD:AAB8584
Alphomelon Deans29|[1092]|DHJPAR0043005|Costa Rica|BOLD:AAB8584
Alphomelon Deans29|[1093]|DHJPAR0043087|Costa Rica|BOLD:AAB8584
Alphomelon Deans29|[1094]|DHJPAR0042412|Costa Rica|BOLD:AAB8584
Alphomelon Deans29|[1095]|DHJPAR0060265|Costa Rica|BOLD:AAB8584
Alphomelon Deans29|[1096]|DHJPAR0060273|Costa Rica|BOLD:AAB8584
Alphomelon Deans29|[1097]|DHJPAR0060274|Costa Rica|BOLD:AAB8584
Alphomelon Deans29|[1098]|DHJPAR0042434|Costa Rica|BOLD:AAB8584
Alphomelon Deans29|[1099]|DHJPAR0042456|Costa Rica|BOLD:AAB8584
Alphomelon Deans29|[1100]|DHJPAR0059007|Costa Rica|BOLD:AAB8584
Alphomelon Deans29|[1101]|DHJPAR0042049|Costa Rica|BOLD:AAB8584
Alphomelon Deans29|[1102]|DHJPAR0061836|Costa Rica|BOLD:AAB8584
Alphomelon Deans29|[1103]|DHJPAR0043126|Costa Rica|BOLD:AAB8584
Alphomelon Deans29|[1104]|DHJPAR0061850|Costa Rica|BOLD:AAB8584
Alphomelon Deans29|[1105]|DHJPAR0042444|Costa Rica|BOLD:AAB8584
Alphomelon Deans29|[1106]|DHJPAR0042445|Costa Rica|BOLD:AAB8584
Alphomelon Deans29|[1107]|DHJPAR0055526|Costa Rica|BOLD:AAB8584
Alphomelon Deans29|[1108]|DHJPAR0042051|Costa Rica|BOLD:AAB8584
Alphomelon Deans29|[1109]|DHJPAR0042447|Costa Rica|BOLD:AAB8584
Alphomelon Deans29|[1110]|DHJPAR0055561|Costa Rica|BOLD:AAB8584
Alphomelon Deans29|[1111]|DHJPAR0042457|Costa Rica|BOLD:AAB8584
Alphomelon Deans29|[1112]|DHJPAR0059034|Costa Rica|BOLD:AAB8584
Alphomelon Deans29|[1113]|DHJPAR0042459|Costa Rica|BOLD:AAB8584
Alphomelon Deans29|[1114]|DHJPAR0042467|Costa Rica|BOLD:AAB8584
Alphomelon Deans29|[1115]|DHJPAR0059040|Costa Rica|BOLD:AAB8584
Alphomelon Deans29|[1116]|DHJPAR0042436|Costa Rica|BOLD:AAB8584
Alphomelon Deans29|[1117]|DHJPAR0041845|Costa Rica|BOLD:AAB8584
Alphomelon Deans29|[1118]|DHJPAR0042056|Costa Rica|BOLD:AAB8584
Alphomelon Deans29|[1119]|DHJPAR0042441|Costa Rica|BOLD:AAB8584
Alphomelon Deans29|[1120]|DHJPAR0059686|Costa Rica|BOLD:AAB8584
Alphomelon Deans29|[1121]|DHJPAR0042468|Costa Rica|BOLD:AAB8584
Alphomelon Deans29|[1122]|DHJPAR0049916|Costa Rica|BOLD:AAB8584
Alphomelon Deans29|[1123]|DHJPAR0059780|Costa Rica|BOLD:AAB8584
Alphomelon Deans29|[1124]|DHJPAR0043075|Costa Rica|BOLD:AAB8584
Alphomelon Deans29|[1125]|DHJPAR0041641|Costa Rica|BOLD:AAB8584
Alphomelon Deans29|[1126]|DHJPAR0058995|Costa Rica|BOLD:AAB8584
Alphomelon Deans29|[1127]|DHJPAR0042501|Costa Rica|BOLD:AAB8584
Alphomelon Deans29|[1128]|DHJPAR0042494|Costa Rica|BOLD:AAB8584
Alphomelon Deans29|[1129]|DHJPAR0061611|Costa Rica|BOLD:AAB8584
Alphomelon Deans29|[1130]|DHJPAR0059054|Costa Rica|BOLD:AAB8584
Alphomelon Deans29|[1131]|DHJPAR0059052|Costa Rica|BOLD:AAB8584
Alphomelon Deans29|[1132]|DHJPAR0059049|Costa Rica|BOLD:AAB8584
Alphomelon Deans29|[1133]|DHJPAR0053026|Costa Rica|BOLD:AAB8584
Alphomelon Deans29|[1134]|DHJPAR0059044|Costa Rica|BOLD:AAB8584
Alphomelon Deans29|[1135]|DHJPAR0059039|Costa Rica|BOLD:AAB8584
Alphomelon Deans29|[1136]|DHJPAR0059036|Costa Rica|BOLD:AAB8584
Alphomelon Deans29|[1137]|DHJPAR0059035|Costa Rica|BOLD:AAB8584
Alphomelon Deans29|[1138]|DHJPAR0059027|Costa Rica|BOLD:AAB8584
Alphomelon Deans29|[1139]|DHJPAR0047185|Costa Rica|BOLD:AAB8584
Alphomelon Deans29|[1140]|DHJPAR0058998|Costa Rica|BOLD:AAB8584
Alphomelon Deans29|[1141]|DHJPAR0060272|Costa Rica|BOLD:AAB8584
Alphomelon Deans29|[1142]|DHJPAR0060262|Costa Rica|BOLD:AAB8584
Alphomelon Deans29|[1143]|DHJPAR0060261|Costa Rica|BOLD:AAB8584
Alphomelon Deans29|[1144]|DHJPAR0042495|Costa Rica|BOLD:AAB8584
Alphomelon Deans29|[1145]|DHJPAR0055293|Costa Rica|BOLD:AAB8584
Alphomelon Deans29|[1146]|DHJPAR0042462|Costa Rica|BOLD:AAB8584
Alphomelon Deans29|[1147]|DHJPAR0042458|Costa Rica|BOLD:AAB8584

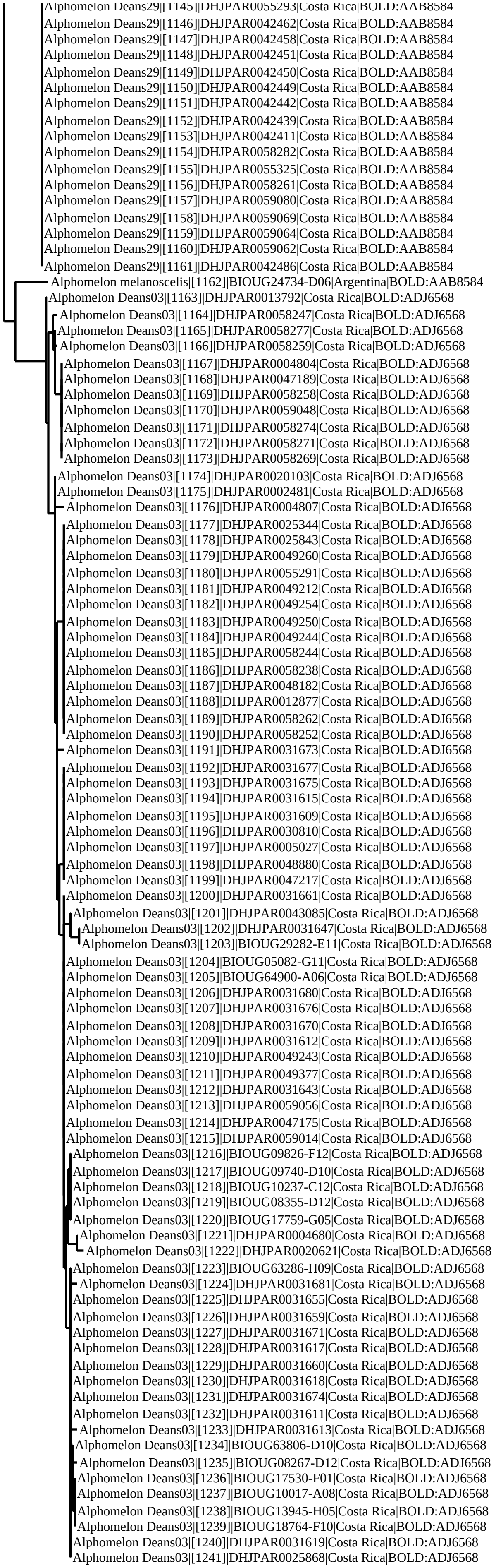
Alphomelon Deans29|[1145]|DHJPAR0055293|Costa Rica|BOLD:AAB8584
Alphomelon Deans29|[1146]|DHJPAR0042462|Costa Rica|BOLD:AAB8584
Alphomelon Deans29|[1147]|DHJPAR0042458|Costa Rica|BOLD:AAB8584
Alphomelon Deans29|[1148]|DHJPAR0042451|Costa Rica|BOLD:AAB8584
Alphomelon Deans29|[1149]|DHJPAR0042450|Costa Rica|BOLD:AAB8584
Alphomelon Deans29|[1150]|DHJPAR0042449|Costa Rica|BOLD:AAB8584
Alphomelon Deans29|[1151]|DHJPAR0042442|Costa Rica|BOLD:AAB8584
Alphomelon Deans29|[1152]|DHJPAR0042439|Costa Rica|BOLD:AAB8584
Alphomelon Deans29|[1153]|DHJPAR0042411|Costa Rica|BOLD:AAB8584
Alphomelon Deans29|[1154]|DHJPAR0058282|Costa Rica|BOLD:AAB8584
Alphomelon Deans29|[1155]|DHJPAR0055325|Costa Rica|BOLD:AAB8584
Alphomelon Deans29|[1156]|DHJPAR0058261|Costa Rica|BOLD:AAB8584
Alphomelon Deans29|[1157]|DHJPAR0059080|Costa Rica|BOLD:AAB8584
Alphomelon Deans29|[1158]|DHJPAR0059069|Costa Rica|BOLD:AAB8584
Alphomelon Deans29|[1159]|DHJPAR0059064|Costa Rica|BOLD:AAB8584
Alphomelon Deans29|[1160]|DHJPAR0059062|Costa Rica|BOLD:AAB8584
Alphomelon Deans29|[1161]|DHJPAR0042486|Costa Rica|BOLD:AAB8584
Alphomelon melanoscelis|[1162]|BIOUG24734-D06|Argentina|BOLD:AAB8584
Alphomelon Deans03|[1163]|DHJPAR0013792|Costa Rica|BOLD:ADJ6568
Alphomelon Deans03|[1164]|DHJPAR0058247|Costa Rica|BOLD:ADJ6568
Alphomelon Deans03|[1165]|DHJPAR0058277|Costa Rica|BOLD:ADJ6568
Alphomelon Deans03|[1166]|DHJPAR0058259|Costa Rica|BOLD:ADJ6568
Alphomelon Deans03|[1167]|DHJPAR0004804|Costa Rica|BOLD:ADJ6568
Alphomelon Deans03|[1168]|DHJPAR0047189|Costa Rica|BOLD:ADJ6568
Alphomelon Deans03|[1169]|DHJPAR0058258|Costa Rica|BOLD:ADJ6568
Alphomelon Deans03|[1170]|DHJPAR0059048|Costa Rica|BOLD:ADJ6568
Alphomelon Deans03|[1171]|DHJPAR0058274|Costa Rica|BOLD:ADJ6568
Alphomelon Deans03|[1172]|DHJPAR0058271|Costa Rica|BOLD:ADJ6568
Alphomelon Deans03|[1173]|DHJPAR0058269|Costa Rica|BOLD:ADJ6568
Alphomelon Deans03|[1174]|DHJPAR0020103|Costa Rica|BOLD:ADJ6568
Alphomelon Deans03|[1175]|DHJPAR0002481|Costa Rica|BOLD:ADJ6568
Alphomelon Deans03|[1176]|DHJPAR0004807|Costa Rica|BOLD:ADJ6568
Alphomelon Deans03|[1177]|DHJPAR0025344|Costa Rica|BOLD:ADJ6568
Alphomelon Deans03|[1178]|DHJPAR0025843|Costa Rica|BOLD:ADJ6568
Alphomelon Deans03|[1179]|DHJPAR0049260|Costa Rica|BOLD:ADJ6568
Alphomelon Deans03|[1180]|DHJPAR0055291|Costa Rica|BOLD:ADJ6568
Alphomelon Deans03|[1181]|DHJPAR0049212|Costa Rica|BOLD:ADJ6568
Alphomelon Deans03|[1182]|DHJPAR0049254|Costa Rica|BOLD:ADJ6568
Alphomelon Deans03|[1183]|DHJPAR0049250|Costa Rica|BOLD:ADJ6568
Alphomelon Deans03|[1184]|DHJPAR0049244|Costa Rica|BOLD:ADJ6568
Alphomelon Deans03|[1185]|DHJPAR0058244|Costa Rica|BOLD:ADJ6568
Alphomelon Deans03|[1186]|DHJPAR0058238|Costa Rica|BOLD:ADJ6568
Alphomelon Deans03|[1187]|DHJPAR0048182|Costa Rica|BOLD:ADJ6568
Alphomelon Deans03|[1188]|DHJPAR0012877|Costa Rica|BOLD:ADJ6568
Alphomelon Deans03|[1189]|DHJPAR0058262|Costa Rica|BOLD:ADJ6568
Alphomelon Deans03|[1190]|DHJPAR0058252|Costa Rica|BOLD:ADJ6568
Alphomelon Deans03|[1191]|DHJPAR0031673|Costa Rica|BOLD:ADJ6568
Alphomelon Deans03|[1192]|DHJPAR0031677|Costa Rica|BOLD:ADJ6568
Alphomelon Deans03|[1193]|DHJPAR0031675|Costa Rica|BOLD:ADJ6568
Alphomelon Deans03|[1194]|DHJPAR0031615|Costa Rica|BOLD:ADJ6568
Alphomelon Deans03|[1195]|DHJPAR0031609|Costa Rica|BOLD:ADJ6568
Alphomelon Deans03|[1196]|DHJPAR0030810|Costa Rica|BOLD:ADJ6568
Alphomelon Deans03|[1197]|DHJPAR0005027|Costa Rica|BOLD:ADJ6568
Alphomelon Deans03|[1198]|DHJPAR0048880|Costa Rica|BOLD:ADJ6568
Alphomelon Deans03|[1199]|DHJPAR0047217|Costa Rica|BOLD:ADJ6568
Alphomelon Deans03|[1200]|DHJPAR0031661|Costa Rica|BOLD:ADJ6568
Alphomelon Deans03|[1201]|DHJPAR0043085|Costa Rica|BOLD:ADJ6568
Alphomelon Deans03|[1202]|DHJPAR0031647|Costa Rica|BOLD:ADJ6568
Alphomelon Deans03|[1203]|BIOUG29282-E11|Costa Rica|BOLD:ADJ6568
Alphomelon Deans03|[1204]|BIOUG05082-G11|Costa Rica|BOLD:ADJ6568
Alphomelon Deans03|[1205]|BIOUG64900-A06|Costa Rica|BOLD:ADJ6568
Alphomelon Deans03|[1206]|DHJPAR0031680|Costa Rica|BOLD:ADJ6568
Alphomelon Deans03|[1207]|DHJPAR0031676|Costa Rica|BOLD:ADJ6568
Alphomelon Deans03|[1208]|DHJPAR0031670|Costa Rica|BOLD:ADJ6568
Alphomelon Deans03|[1209]|DHJPAR0031612|Costa Rica|BOLD:ADJ6568
Alphomelon Deans03|[1210]|DHJPAR0049243|Costa Rica|BOLD:ADJ6568
Alphomelon Deans03|[1211]|DHJPAR0049377|Costa Rica|BOLD:ADJ6568
Alphomelon Deans03|[1212]|DHJPAR0031643|Costa Rica|BOLD:ADJ6568
Alphomelon Deans03|[1213]|DHJPAR0059056|Costa Rica|BOLD:ADJ6568
Alphomelon Deans03|[1214]|DHJPAR0047175|Costa Rica|BOLD:ADJ6568
Alphomelon Deans03|[1215]|DHJPAR0059014|Costa Rica|BOLD:ADJ6568
Alphomelon Deans03|[1216]|BIOUG09826-F12|Costa Rica|BOLD:ADJ6568
Alphomelon Deans03|[1217]|BIOUG09740-D10|Costa Rica|BOLD:ADJ6568
Alphomelon Deans03|[1218]|BIOUG10237-C12|Costa Rica|BOLD:ADJ6568
Alphomelon Deans03|[1219]|BIOUG08355-D12|Costa Rica|BOLD:ADJ6568
Alphomelon Deans03|[1220]|BIOUG17759-G05|Costa Rica|BOLD:ADJ6568
Alphomelon Deans03|[1221]|DHJPAR0004680|Costa Rica|BOLD:ADJ6568
Alphomelon Deans03|[1222]|DHJPAR0020621|Costa Rica|BOLD:ADJ6568
Alphomelon Deans03|[1223]|BIOUG63286-H09|Costa Rica|BOLD:ADJ6568
Alphomelon Deans03|[1224]|DHJPAR0031681|Costa Rica|BOLD:ADJ6568
Alphomelon Deans03|[1225]|DHJPAR0031655|Costa Rica|BOLD:ADJ6568
Alphomelon Deans03|[1226]|DHJPAR0031659|Costa Rica|BOLD:ADJ6568
Alphomelon Deans03|[1227]|DHJPAR0031671|Costa Rica|BOLD:ADJ6568
Alphomelon Deans03|[1228]|DHJPAR0031617|Costa Rica|BOLD:ADJ6568
Alphomelon Deans03|[1229]|DHJPAR0031660|Costa Rica|BOLD:ADJ6568
Alphomelon Deans03|[1230]|DHJPAR0031618|Costa Rica|BOLD:ADJ6568
Alphomelon Deans03|[1231]|DHJPAR0031674|Costa Rica|BOLD:ADJ6568
Alphomelon Deans03|[1232]|DHJPAR0031611|Costa Rica|BOLD:ADJ6568
Alphomelon Deans03|[1233]|DHJPAR0031613|Costa Rica|BOLD:ADJ6568
Alphomelon Deans03|[1234]|BIOUG63806-D10|Costa Rica|BOLD:ADJ6568
Alphomelon Deans03|[1235]|BIOUG08267-D12|Costa Rica|BOLD:ADJ6568
Alphomelon Deans03|[1236]|BIOUG17530-F01|Costa Rica|BOLD:ADJ6568
Alphomelon Deans03|[1237]|BIOUG10017-A08|Costa Rica|BOLD:ADJ6568
Alphomelon Deans03|[1238]|BIOUG13945-H05|Costa Rica|BOLD:ADJ6568
Alphomelon Deans03|[1239]|BIOUG18764-F10|Costa Rica|BOLD:ADJ6568
Alphomelon Deans03|[1240]|DHJPAR0031619|Costa Rica|BOLD:ADJ6568
Alphomelon Deans03|[1241]|DHJPAR0025868|Costa Rica|BOLD:ADJ6568
